# Status and Challenges of Blue OLEDs: A Review

**DOI:** 10.3390/nano13182521

**Published:** 2023-09-08

**Authors:** Iram Siddiqui, Sudhir Kumar, Yi-Fang Tsai, Prakalp Gautam, Shahnawaz, Kiran Kesavan, Jin-Ting Lin, Luke Khai, Kuo-Hsien Chou, Abhijeet Choudhury, Saulius Grigalevicius, Jwo-Huei Jou

**Affiliations:** 1 Department of Materials Science and Engineering, National Tsing Hua University, Hsinchu 30013, Taiwan; 2 Institute for Chemical and Bioengineering, ETH Zürich, 8093 Zürich, Switzerland; 3 Department of Polymer Chemistry and Technology, Kaunas University of Technology, LT-50254 Kaunas, Lithuania

**Keywords:** organic light-emitting diode (OLED), blue and deep-blue OLEDs, high-efficiency, lifetime, chemical structure

## Abstract

Organic light-emitting diodes (OLEDs) have outperformed conventional display technologies in smartphones, smartwatches, tablets, and televisions while gradually growing to cover a sizable fraction of the solid-state lighting industry. Blue emission is a crucial chromatic component for realizing high-quality red, green, blue, and yellow (RGBY) and RGB white display technologies and solid-state lighting sources. For consumer products with desirable lifetimes and efficiency, deep blue emissions with much higher power efficiency and operation time are necessary prerequisites. This article reviews over 700 papers covering various factors, namely, the crucial role of blue emission for full-color displays and solid-state lighting, the performance status of blue OLEDs, and the systematic development of fluorescent, phosphorescent, and thermally activated delayed fluorescence blue emitters. In addition, various challenges concerning deep blue efficiency, lifetime, and approaches to realizing deeper blue emission and higher efficacy for blue OLED devices are also described.

## 1. Introduction

### 1.1. World Revenues and Forecasts for Lighting and Display

Organic light-emitting diodes (OLEDs) have attracted great attention due to their applications in high-contrast, innately thin, easily adaptable, full-color, flat-panel displays and solid-state lighting [1,2,3,4,5,6,7,8]. Nowadays, OLEDs are almost unanimously leading the portable display market and strongly contesting the market for large-area displays with reasonably sound efficiency and operational stability. Particularly, all leading smartphone and smartwatch companies, including Samsung, Apple, LG, Sony, Nokia, Huawei, Xiaomi, OPPO, OnePlus, and Vivo, are marketing their flagship devices with high-quality OLED displays. OLED revenues are currently driven by high-quality display applications. In addition, the lighting market is also gradually emerging. Due to their numerous inherently disruptive advantages, including a plane light form, a very high color rendering index, tunable color temperature, diffused emission, flexibility, transparency, being fully dimmable, human-friendliness, a wide viewing angle, being made with mercury-free, sustainable raw materials, being ultra-thin and light-weight, an ultimate high contrast, fast response time, and energy-saving, etc., flat panel displays and solid-state lighting panels will, sooner or later, take up a major portion of revenue and eventually disrupt conventional technologies [5,9,10,11,12,13,14].

**Figure 1:**
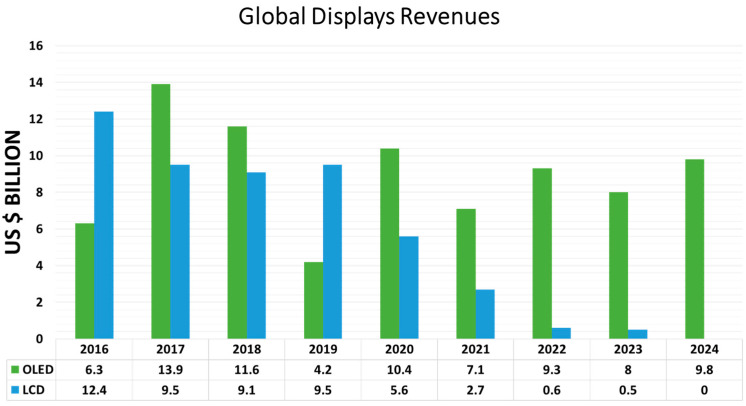
Global revenues of LCD- and OLED-based displays from 2016 to 2024. Keyword: Global revenue OLED display 2020. Source: DSCC’s Quarterly Display Capex and Equipment Market Share Report [15].

Figure 1 compares the global revenue of OLED displays with that of LCD displays reported by DSCC’s Quarterly Display Capex and Equipment Market Share Report [15]. Overall, the revenue of LCDs experienced a catastrophic fall, as it dropped from 12.4 billion in 2016 to 5.6 billion in 2020. It is expected to drop further to less than 1 billion in 2024. In contrast, the revenue of OLED displays peaked at 13.9 billion in 2017. Since then, it has slightly oscillated around 10 billion and is projected continue to do so going into 2024. OLED is a disruptive technology in the display market, especially since its revenue surpassed that of LCD displays. Undoubtedly, OLED is and will remain the mainstream display technology for a few more decades.

**Figure 2:**
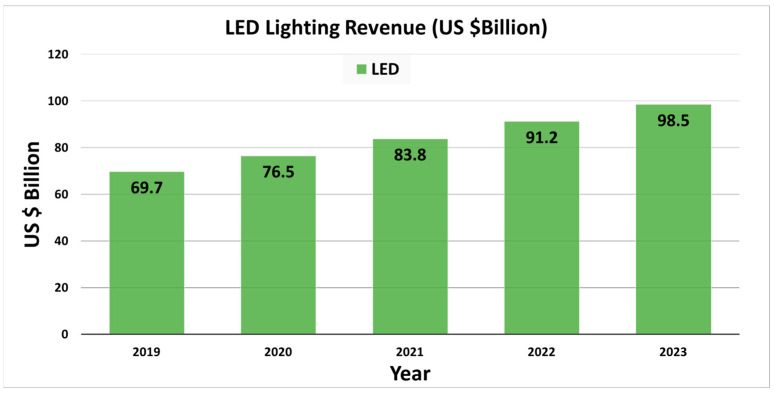
Global LED Lighting Revenues and Forecast by Statista Research Development. Keyword: Global revenue LED lighting. Source: https://www.statista.com/statistics/753939/global-led-luminaire-market-size [16].

A recent report from Statista depicted global annual revenue for LED lighting and estimated it to be 70 billion in 2019. An increment of 41% is expected until 2023, with a forecasted amount of 99 billion annually. However, on closer analysis, there was an approximate annual increase in value of less than 10 billion from 2019–2023 (Figure 2) [16]. This reduction in market size can be attributed to the rise of OLEDs, which are expected to overcome the LED market.

**Figure 3:**
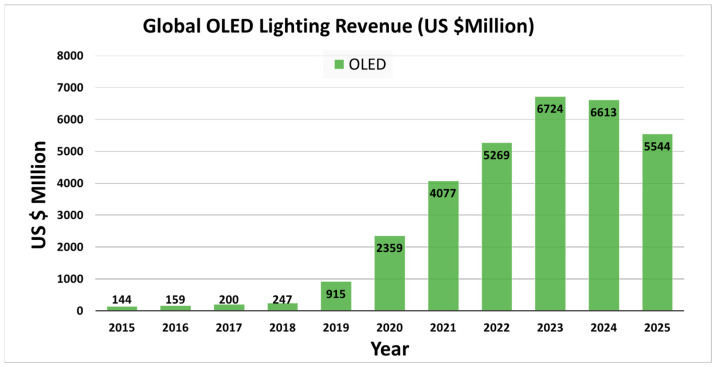
Global OLED Lighting Revenues and Forecast by ElectroniCast. Keyword: Global revenue OLED lighting. Source: https://www.oled-info.com/electronicast-sees-fast-growing-oled-lighting-market-starting-2015 [17].

On the other hand, ElectroniCast, a US-based company, says that the estimated global revenue for the OLED lightings is set to grow speedily from 0.14 billion in markets in 2015 to nearly 7 billion by 2023, representing a compound annual growth rate (CAGR) of 7% in the next ten years (Figure 3). According to the above statics, OLED lighting revenue is expected to grow rapidly from 2015–2025. The forecast also displays a slight decline in the annual revenue around 2025, with an estimated value of 5.5 billion [17]; however, on studying the market size, OLEDs for lighting applications show a significant rise and are expected to hold the entire global market. 

### 1.2. OLED—The Ultimate Display Technology

Twenty-five years ago, the first OLED product, a monochromatic display in car stereos, was launched by Pioneer [18]. Since then, researchers have made tremendous efforts in developing high-quality OLED displays. Nowadays, OLEDs are extensively applied in portable display devices, such as laptops, smartphones, mp3/mp4 players, digital cameras, smart watches, electric razors, and PDAs [19]. After years of struggle, large-size OLED displays are reaching the verge of a commercial breakthrough. In 2007, Sony developed the world’s first 11-inch OLED TV (XEL-1); in 2019, Sony developed the largest screen available on the market, i.e., the 77-inch A9G [20]. Other large companies, such as LG, Samsung, Panasonic, Sharp, AU Optronics, and BOE, have also launched ultra-high definition (UHD) full-color OLED TVs with various display sizes. A few years back, AU Optronics was set to demonstrate a 17.3-inch display with a 4K (225 PPI) resolution fabricated using inkjet technology. Additionally, AUO will be showcasing a 5.6-inch foldable active-matrix organic light-emitting diode (AMOLED) [21]. Meanwhile, BOE also fabricated an inkjet printed 55-inch OLED display TV with an 8K resolution and a claimed maximum brightness of 400 nits with a color gamut of 95% [22]. The global enterprise trends of OLED displays are rapidly growing, and it is clear that OLED displays will dominate the flat panel display industry in the coming years. In 2016, LG began producing their flat panel display units after long use of curved displays. This panel design has been adopted by some other major companies for applications in TVs, laptops, and desktops [23]. 

### 1.3. OLED—The Best Lighting Technology

There is a strong demand for energy-efficient and optimal next-generation lighting because approximately 20% of total global electricity is consumed for general lighting [24]. Researchers have extensively accepted that the OLEDs must become a mainstream technology for next-generation illumination sources within a few years because of their numerous distinctive features, e.g., lightweight, uniform, cold-light, human-eye friendly, and soft surface emission, feasible to form on conformable substrates, free from ultraviolet (UV) and near-infrared (NIR) emission, glare-free, environmentally friendly, high CRI, and extremely energy efficient [10,13,14,25,26]. Conventional light sources cannot provide these advantages, such as incandescent bulbs, fluorescent tubes, and light-emitting diodes (LEDs). Therefore, due to high demand for a human-friendly light source, intensive research is ongoing for better OLED lighting. 

Kido et al. discovered the first white OLED in 1993 [27]. Since then, there has been rapid progress in both academic and industrial OLED research and development. In 2009, Leo et al. reported a white OLED structure showing a fluorescent tube efficacy of 90 lm/W with a CRI of 80 at 1000 cd/m^2^ [28]. Panasonic introduced a 4 mm^2^ OLED with 128 lm/W and 1 cm^2^ white OLED panels with an efficacy of 114 lm/W at 1000 cd/m^2^ [29]. Panasonic also developed a 25 cm^2^ white OLED panel with 110 lm/W efficacy and a lifetime of over 100,000 h (L_50_) at 1000 cd/m^2^ by employing all phosphorescent materials [30]. In 2014, Konica Minolta demonstrated an OLED with a record high efficacy of 139 lm/W at 1000 cd/m^2^ using all phosphorescent materials [31].

Commercially, Osram introduced the first OLED-based functional table lamp in 2008 [32,33]. In 2009, Philips started selling its OLED lighting panel under the trade name of Lumiblade [34,35]. Lumiotech introduced a high CRI of 90 for natural white lighting panels with an efficacy of 45 lm/W and a lifetime of 40,000 h (L_50_) at 3000 cd/m^2^ [36]. LG Chem. developed white OLED panels with an efficacy of 60 lm/W and a lifetime of 40,000 h (L_70_) at 3000 cd/m^2^ [34] (Table 1). Other companies such as UDC, Idemitsu, Mitsubishi Chemical, Verbatim, Fraunhofer COMEDD, and Konica Minolta have followed the same path to enhance product viability in the consumer market [37].

Furthermore, high-efficiency, tunable color-temperature, high-CRI, and low-MSI OLED panels are being fabricated, and few are exclusively available in the market. The time is not far when OLED panels will light every household, workplace, library, and even hospital, and human health will no longer be at risk.

## 2. The Crucial Roles of Blue Emitters

To realize high-quality OLED displays and solid-state lighting, extensively energy-efficient and long-lifetime red, green, and blue (RGB) primary emitters are indispensable. Many high-potential red and green emitters have been developed, and they are extensively accepted for commercial OLED products [41,42]. High-quality blue emitters are still a major challenge, and the development of blue-light emitting materials exhibiting high efficiency and long lifetimes is an issue of current interest.

### 2.1. Importance of Blue Emission

Red and green lights have been used for over a century. The need for blue light has risen to fabricate energy-efficient white light to revolutionize the lighting industry. Red, green, and blue light can provide a continuous spectrum, emitting white light. Blue emission is the color of light between violet and green, which can be classified in four different chromaticity variables representing different CIE_y_ value ranges: ultra-deep blue (CIE_y_ < 0.05), deep blue (CIE_y_ < 0.10), blue (CIE_y_ < 0.25), and sky-blue (CIE_y_ < 0.35). The development of a deep blue emitter is an essential requirement to realize high-quality displays and solid-state lighting. In addition, promising deep-blue emitters must also match the National Television System Committee (NTSC) standard with Commission Internationale de L’Eclairage (CIE) coordinates of (0.14, 0.08) for full-color display applications [41].

In the 1990s, Shuji Nakamura and his co-workers, Akasaki and Anamo, invented the blue light-emitting diode (LED) using high-quality crystals of GaN. The main purpose of this invention was to produce energy- and resource-efficient lighting that consumes comparatively less power for illumination and is cheap enough to light every household. They were awarded a Nobel prize for this esteemed invention that consumes much less energy than other available lighting resources. These LEDs hold a long operating lifetime of over 100,000 h, which is 110 times higher than incandescent bulbs and white filament lamps, respectively [43,44,45].

### 2.2. To Enable a White Light

Typically, white electroluminescence in OLED devices has been produced through three major strategies: (i) mixing of primary red, green, and blue (RGB) emissions, (ii) development of a white emitter that consists of multiple spectral peaks covering the entire visible range, and (iii) utilization of complementary emitters, such as orange and blue or yellow and blue emitters. Consequently, blue emitters are crucial primary emission components for producing white emission chromaticity. 

### 2.3. To Enable a High Color Gamut

The color gamut refers to the range of colors that any display can potentially display. There are two types of color gamut, additive and subtractive. An additive color gamut refers to color generated by mixing two or more color lights to generate a desired color. Subtractive color is generated by mixing more than one dye that absorbs certain wavelengths of light and reflects others to produce a color; it is used in color printing [46,47,48]. Considering a display’s high color gamut range, the organic emitters used in an OLED display should exhibit color purity in RGB emission [49,50]. Researchers have broadly accepted that extremely deep-blue emitters can play a considerable role in developing an incredibly high color gamut. The color gamut of NTSC can be exceptionally enhanced by replacing the sky-blue emitter with an extremely deep blue counterpart in displays [51,52,53].

### 2.4. To Enable a High Color Rendering Index

The color rendering index (CRI) is a significant indicator representing the quality of illumination. High CRI is a noteworthy indicator of light quality for applications in art galleries and exhibition centers because it can reveal the true color of paintings and artworks faithfully under artificial lighting. Additionally, it has been extensively confirmed that deep-blue emission plays a crucial role in achieving a high CRI. Leo’s group reported a hybrid white OLED that used phosphorescent red and green emitters with fluorescent deep-blue counterparts, which exhibited a CRI of 86 with an efficacy of 22 lm/W at 1000 cd/m^2^ [54]. Yang et al. developed a CRI of 91 using a triple emissive layer device architecture [55]. In 2011, Jou et al. fabricated a double emissive layer white OLED with a record high CRI of 96 and 5.2 lm/W efficacy [56]. In the industrial sector, LG Chem. and Lumiotech achieved over 90 CRI for OLED lighting panels with a power efficacy of 60 lm/W and 28 lm/W, respectively, at 3000 cd/m^2^ [57,58].

## 3. Papers

For nearly a decade, the research communities and industry have worked on high-performance long-lifetime deep blue-emitting OLED devices. OLEDs are divided into three groups based on their luminescent organic materials: fluorescent (Generation 1, Gen-1), phosphorescent (Generation 2, Gen-2), and thermally activated delayed fluorescence (TADF) (Generation 3, Gen-3). Blue fluorescent OLEDs have a good operating lifespan and emit a deep-blue light, but they are less effective at emitting light per unit area than phosphorescent OLEDs. Phosphorescent blue emitters are commonly used to achieve high performance. Nevertheless, owing to their short operating life and low color coordination, the extensive use of the phosphorescent emitters in OLEDs is limited. The trends and the developments of previously recorded blue OLED journals have been listed in this section.

### Trends and Development

**Figure 4:**
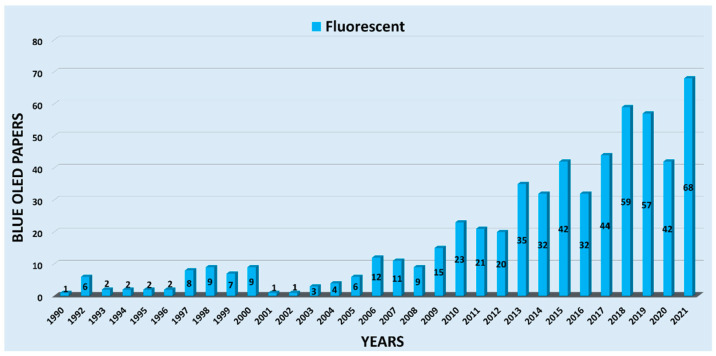
Research papers on blue fluorescent OLEDs per year since 1990. Keywords: Fluorescent blue organic light-emitting device. Source: Web of science.

The trend and development of the number of journal papers on fluorescent-based blue OLEDs are illustrated in the bar graph in Figure 4. Adachi and his team reported the first blue OLED device in 1990 in Applied Physics Letters [59]. In 1992, around six fluorescent material-based blue OLED papers were reported. From 1993 to 1996, there were only two papers reported per year. However, the average number of papers per year was recorded as eight from 1997–2000. In 2001 and 2002, the number of publications decreased to one paper per year, but gradually increased in the following years. The average number of papers per year was determined to be around sixteen from 2006–2012. Furthermore, after 2012, we found a drastic enhancement in the publications, and more than 30 publications were counted per year. The highest number of journal papers per year was counted as 68; these were published over 2021.

**Figure 5:**
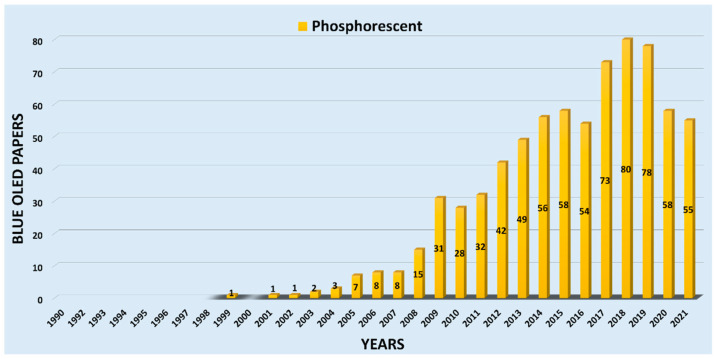
Research papers on blue phosphorescent OLEDs per year; the first paper appeared in 1999 and rapidly peaked to 31 papers in 2009. Keywords: Phosphorescent blue organic light-emitting device. Source: Web of science.

Figure 5 displays the number of publications on phosphorescent-based blue OLEDs (PHOLED) per year. In 2001, Adachi et al. exhibited a phosphorescent blue emitter, FIrpic, and published an article in Applied Physics Letters [60]. In the succeeding year, Kawamura et al. published an article in the same journal on blue PHOLED devices fabricated using PVK as a host and Ir (III) complex as a dopant [61]. Moreover, we noticed that from 2002, the number of journal papers gradually increased, with around ten papers published each year till 2007. Furthermore, a rise in publication was observed till 2015, however, there still were fewer than fifteen papers per year. In contrast, there was a drastic increment from 2015–2019, with an average of 36 papers per year. The highest count reached eighty in 2018. On average, 66 articles were published on blue PHOLED from 2016 to 2021.

**Figure 6:**
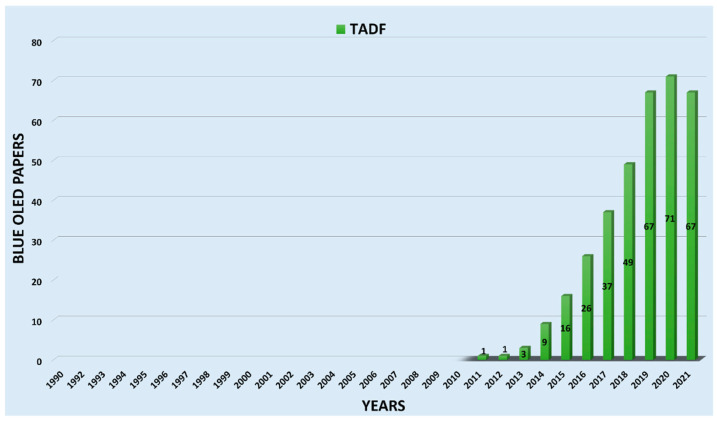
Research papers on blue TADF OLEDs per year since 2015; the first paper appeared in 2012 and rapidly peaked to 71 papers in 8 years. Keywords: TADF blue organic light-emitting device. Source: Web of science.

Figure 6 illustrates yearly publication on thermally activated delayed fluorescent (TADF)-based blue OLEDs. The bar graph shows that the number of publications rapidly increased since the first TADF-based blue OLED paper was reported and peaked to 71 in 8 years. In 2012, Lee et al. reported a blue TADF emitter CC2TA comprising a bicarbazole donor and phenyltriazine acceptor units in the Applied Physics Letter [62]. In 2013, Adachi and his team published three papers [63,64,65]. In 2015, a report from Mallesham et al. demonstrated pyrene excimer-based blue emission [66]. Subsequently, in the same year, Liu et al. also published an article based on designing and synthesizing blue TADF emitters for OLED devices [67]. In addition, Adachi et al. reported a sky-blue OLED device in the same year and published an article. They studied a sky-blue OLED based on a TADF molecule, 1,2-bis(carbazol-9-yl)-4,5-dicyanobenzene (2CzPN) [68]. A total of 16 articles were published in 2015. For the following years, the number of reports continuously rose, with the publication peaking in 2020 and slightly decreasing in 2021.

**Figure 7:**
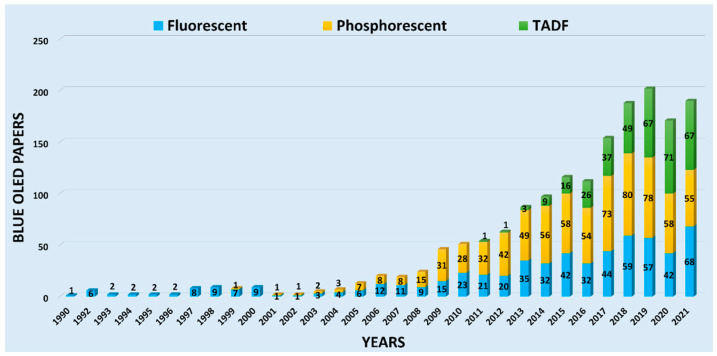
An overall chart depicting the rise of blue OLED papers using fluorescent (Gen-1), phosphorescent (Gen-2), and TADF (Gen-3) emitters. The yearly paper amount for Gen-2 emitter-composed devices surpassed that of Gen-1 counterparts in 2008 (after the first paper was published in 2001), while the yearly paper amount for Gen-3-based devices surpassed that of Gen-2 in 2020 (after the first paper was published in 2012). Keywords: Blue organic light-emitting device. Source: Web of science.

Figure 7 depicts the rise of blue OLED papers using fluorescent (Gen-1), phosphorescent (Gen-2) and TADF (Gen-3) emitters. The yearly paper amount for Gen-2 emitter-composed devices surpassed that of Gen-1 counterparts in 2008 (after the first paper was published in 2001), while the yearly paper amount of Gen-3-based devices surpassed that of Gen-2 in 2021 (after the first paper was published in 2012). 

## 4. Patent

**Figure 8:**
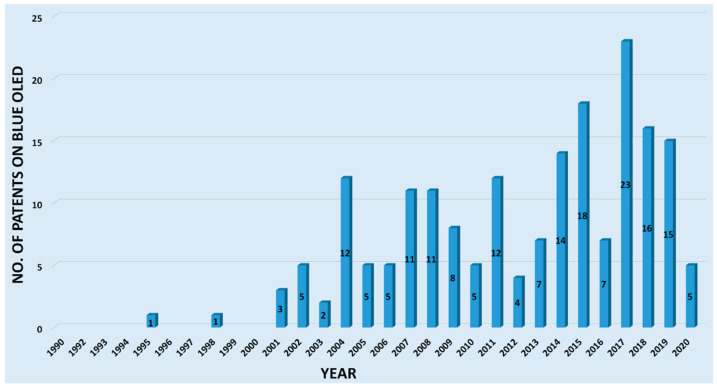
Blue OLEDs were seemingly quite challenging to devise since there was only one patent filed in 1998 after the first patent appeared in 1995. There were no blue OLED patents issued in 1999 or 2000. After that, blue OLED patents were continuously granted, peaking to 23 in the year 2017. The overall increasing trend over the past 20 and some years indicates that blue OLEDs are critically crucial. Keyword: Blue organic light-emitting diodes. Source: patentscope.wipo.int [71].

Figure 8 depicts the number of granted patents for blue OLEDs from 1995–2020. Blue OLEDs were seemingly quite challenging to devise, since there was just one patent in 1998, after the first patent in 1995. The first patent was filed by Hosokawa et al. from Idemitsu Kosan Co. Ltd. (Tokyo, Japan) [69], demonstrating the feasibility of fabricating a blue OLED on the basis of a polycarbonate containing a styryl-amine or diarylvinylene arylene skeleton. 

In 1998, the first small molecule-based blue OLED patent was granted to Antoniadis et al. from Agilent Technologies Inc., wherein oxadiazole-, thiadiazole-, or triazole-based blue emitters were used [70]. There were no blue OLED patents issued in 1999 or 2000. 

In 2001, Adachi et al. from Princeton University received a patent based on transition metals, wherein zinc-based derivatives exhibited both blue fluorescence and phosphorescence, the beginning of the phosphorescent mechanism [71].

After that, blue OLED patents were continuously granted, peaking to 23 in 2017. The overall increasing trend over the past 20 and some years indicates that blue OLEDs are critically crucial.

## 5. Performance Status

Nearly two decades ago, both academia and industries started making efforts to develop high-efficiency and long-lifetime OLED devices with deep-blue emission. Based on luminescent organic materials, OLEDs are generally classified into three main categories: fluorescent, phosphorescent, and thermally activated delayed fluorescence (TADF).

Blue fluorescent OLEDs possess a fair operational lifetime and deep-blue emission, but they are typically less efficient at emitting light per unit area than their phosphorescent counterparts. 

To realize high efficiency, phosphorescent blue emitters are extensively used. However, the widespread use of phosphorescent emitters is limited due to the absence of deep-blue emission. Further, to resolve the limitation, TADF materials were developed and many more are under development. In this section, we describe the efficiency and lifetime records for previously reported blue OLEDs.

### 5.1. Fluorescent Blue OLEDs

#### 5.1.1. EQE vs. CIE_y_

**Figure 9:**
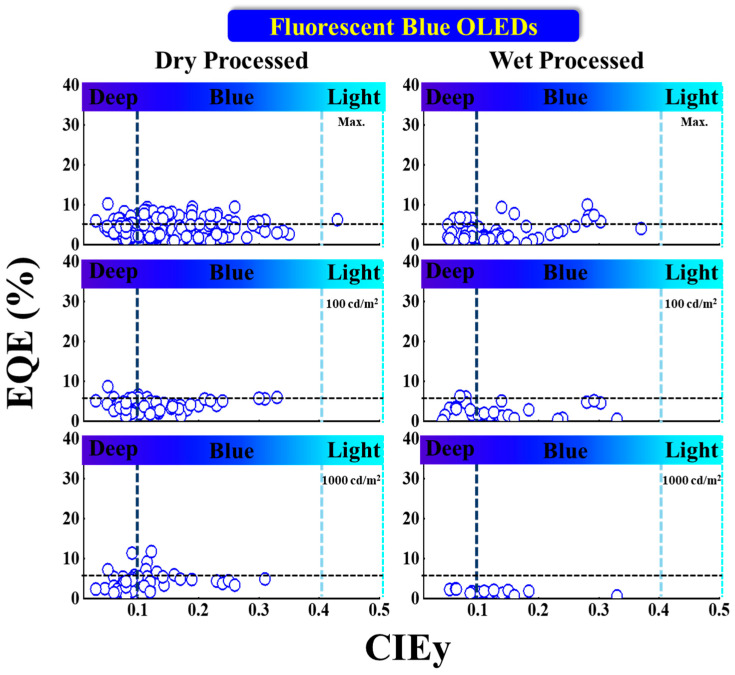
EQE performance vs. CIE_y_ of fluorescent blue OLEDs with dry and wet processes. The horizontal black dashed lines represent the theoretical 5% upper limit. The vertical dark and light blue dashed lines represent the region for deep blue and greenish blue, respectively. Some deep-blue devices were reported with a greater than 5% EQE at 1000 cd/m^2^ with a CIE_y_ less than 0.1 by using the dry process.

Figure 9 shows the EQE performance vs. CIE_y_ of fluorescent blue OLEDs with dry and wet processes. The horizontal black dashed lines represent the theoretical 5% upper limit. The vertical dark and light blue dashed lines represent the region for deep blue and greenish blue, respectively. 

Some deep-blue device efficiencies were reported with a greater than 5% EQE at 1000 cd/m^2^ with a CIE_y_ less than 0.1 by using the dry process. Among these, Yamazaki et al. reported a highest EQE of 11% at 1000 cd/m^2^ with CIE coordinates of (0.14, 0.09) using a BD-06 deep-blue emitter. The same report published a 12% EQE blue emission with coordinates of (0.14, 0.12) using a BD-05 emitter [72]. Liu et al. reported, at 100 cd/m^2^, a deep-blue emitter BBPA (at 5 wt%) with an 8.7% EQE at (0.15, 0.05) [73].

For wet-processed deep-blue fluorescent OLEDs, Wu et al. reported a 6.8% EQE at 100 cd/m^2^ with CIE coordinates of (0.16, 0.08) by using a host-free emitter T3 [74], while at 1000 cd/m^2^, Ma et al. reported a 6.2% EQE at (0.15, 0.08) by using a host-free emitter (G0) [75].

For emission with a greater than 0.1 CIE_y_, the maximum reported EQE is 10% with coordinates of (0.15, 0.28), which displays a bluish-green emission using DSA-Ph emitter doped in an MADN (host) [76]. At 100 cd/m^2^, the EQE achieved is 5.1% at (0.14, 0.19) by employing a host-free Blu2 blue emitter [77]. 

#### 5.1.2. Power Efficacy vs. CIE_y_

**Figure 10:**
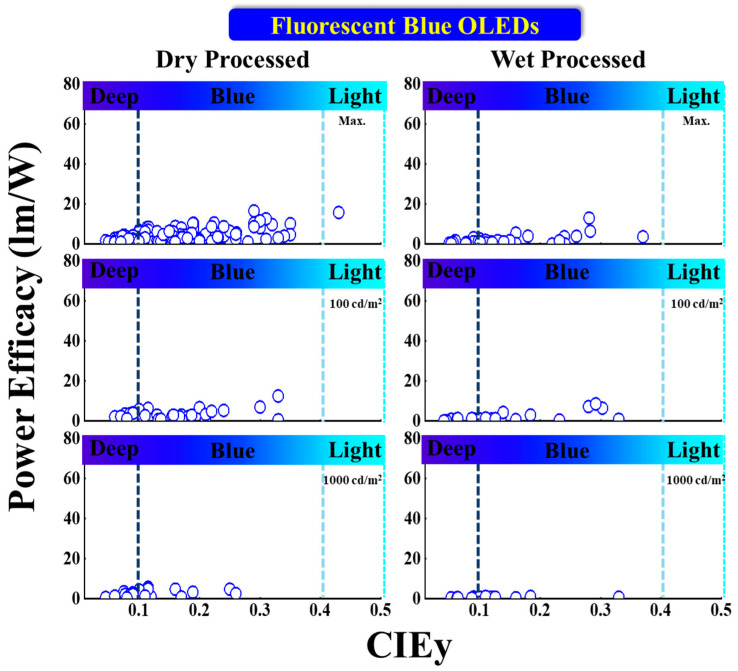
Power efficacy (PE) performance vs. CIE_y_ of fluorescent blue OLEDs with dry and wet processes. The vertical dark and light blue dashed lines represent the region for deep blue and greenish blue, respectively. None of the deep-blue devices shows a greater than 10 lm/W power efficacy at any reported luminance, regardless of whether dry or wet processes were used.

Figure 10 shows the power efficacy (PE) performance vs. CIE_y_ of fluorescent blue OLEDs with dry and wet processes. The vertical dark and light blue dashed lines represent the region for deep blue and greenish blue, respectively. None of the deep-blue devices show a greater than 10 lm/W power efficacy at any reported luminance, regardless of whether dry or wet processes were used.

Among the dry-processed deep-blue devices, Liu et al. published a PE of 6.1 lm/W at 100 cd/m^2^ with CIE coordinates of (0.14, 0.1) by using an emitter NI-2-PhTPA [78]. Li et al. reported a PE of 17 lm/W for a greenish-blue emission with coordinates (0.15, 0.26) by utilizing a DSAph emitter doped in an MBA (host) [79]. 

For wet-processed deep-blue devices, Ma et al. reported a maximum PE (PE_max_) of 3 lm/W with CIE_xy_ of (0.15, 0.08) using a host-free emitter (G0); the same device showed a 2.4 lm/W PE at 1000 cd/m^2^ [75]. Li et al. reported a PE of 13 lm/W at 100 cd/m^2^ for a bluish-green emission with CIE coordinates of (0.15, 0.28) by using a DSA-Ph emitter [76]. Moreover, Zhen et al. reported a PE of 8.5 lm/W at 1000 cd/m^2^ with coordinates (0.14, 0.29) by utilizing a host-free Blu2 emitter [77].

#### 5.1.3. Current Efficacy vs. CIE_y_

**Figure 11:**
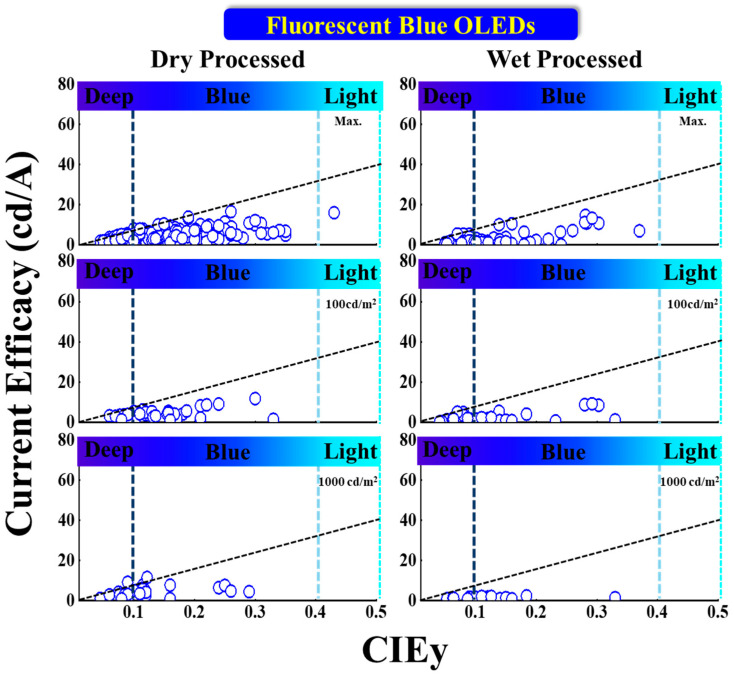
Current efficacy (CE) vs. CIE_y_ for dry- and wet-processed fluorescent blue OLEDs. From display application perspective, commercially viable ones are those with a CIE_y_ lying on or above the dashed line with a CE equal to 70*CIE_y_. The vertical dark and light blue dashed lines represent the region for deep blue and greenish blue, respectively.

Figure 11 illustrates the current efficacy (CE) vs. CIE_y_ for dry- and wet-processed fluorescent blue OLEDs. From the display application perspective, commercially viable products are those with a CIE_y_ lying on or above the dashed line with a CE equal to 70*CIEy [80]. The vertical dark and light blue dashed lines represent the region for deep blue and greenish blue, respectively.

Among the dry-processed devices, Kin and Hong et al. reported a maximum CE (CE_max_) of 5.1 cd/A for deep-blue emission with CIE_xy_ (0.15, 0.08) by using a T1b emitter [81]. Meanwhile, Liu et al. reported a 3.6 cd/A CE at 100 cd/m^2^ with coordinates (0.14, 0.08) by using an NI-2-TPA emitter [78].

Takita et al. reported one commercially viable deep-blue emitter showing a 9.2 cd/A CE at 1000 cd/m^2^ at (0.14, 0.09) by using (at 1 wt%) a BD-06 emitter. The other commercially viable emitter reported by the same group showed a CE of 11.6 cd/A at the same luminance for blue emission with coordinates (0.14, 0.12) by using (at 1 wt%) a BD-05 emitter [72]. 

For the wet-processed devices, Li et al. reported a highest CE of 14.7 cd/A for a greenish-blue emission with coordinates (0.15, 0.28) by employing a DSAph emitter [76]. 

Zou et al. published two commercially viable deep-blue emitters with a CE_max_ of 5.4 cd/A at (0.16, 0.07) by using a T3 host-free emitter. Moreover, the same device showed a 5.1 cd/A CE at 100 cd/m^2^ with the same CIE_y_ [74]. However, no commercially viable device has been obtained with a 1000 cd/m^2^ yet.

#### 5.1.4. Lifetime and Current Efficacy vs. CIE_y_

**Figure 12:**
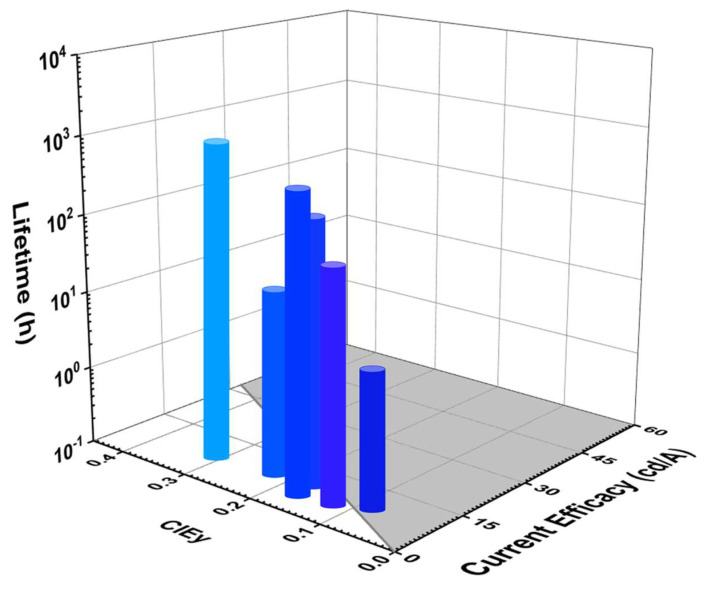
Lifetime and current efficacy vs. CIE_y_ for fluorescent blue OLEDs. The shaded area represents a CE ≥ 70*CIE_y_ [30,37,41,42,43]. The different shades of blue denote the emission-color at CIE_y_.

Figure 12 shows the lifetime and current efficacy vs. CIE_y_ for fluorescent blue OLEDs. The shaded area represents a CE ≥ 70*CIE_y_ [30,37,41,42,43]. As shown, one deep-blue fluorescent device met the commercially viable criteria. Takita et al. reported a device with a lifetime of 125 h and a CE_max_ of 9.2 cd/A corresponding to a CIE_y_ of 0.09 by using a BD-06 emitter [72]. Moreover, Jeon et al. reported a device closest to the criteria showing a lifetime of 168 h and a CE_max_ of 9.1 cd/A corresponding to a CIE_y_ of 0.15 by using a BD-6MDPA emitter [82]. 

Wei et al. reported a device close to the criteria with a lifetime of 110 h and a 4.9 cd/A CE_max_ corresponding to a CIE_y_ 0.11 by using a 5 wt% BN1 emitter [83].

Table 2 shows the efficiency and lifetime performance of dry-processed fluorescent blue OLEDs. All the lifetime data have also been converted to the same 1000 nits for easy comparison. Takita et al. reported a lifetime of 125 h at a 10% lifetime decay (LT_90_) and a maximum EQE (EQE_max_) of 11.8% at 1000 nits for a deep-blue emission with a CIE_y_ of 0.09 by using a BD-06 emitter [72]. Wei et al. reported a lifetime of 110 h at a half lifetime decay (LT_50_) at 1176 cd/m^2^ and an EQE_max_ of 8.6% for a blue emission with a CIE_y_ of 0.11. A lifetime of 121 h estimated as the initial brightness is converted to 1000 cd/m^2^ [83]. 

S.W. Wen et al. reported a highest lifetime of 7000 h (at LT_50_) at 100 cd/m^2^ and a 9.7 CE_max_ and 11.5% EQE_max_ for a bluish-green emission with a CIE_y_ of 0.32 by using a 3 wt% DSA-Ph emitter in an MADN host. At 1000 cd/m^2^_,_ the same device is estimated to have a 1662 h (LT_50_) of lifetime [84].

Table S1 shows a comprehensive list of dry-processed blue fluorescent OLED devices, including the publication year, device structure, employed host, key dopant, doping concentration, HOMO, LUMO, EQE, PE, CE, CIE (X), CIE (Y), and reference. The years span from 2006 to 2020. A complete set of efficiencies at 100 and/or 1000 cd/m^2^ and/or the maximum efficiency are reported for deep-blue and blue emission.

Table S2 shows a comprehensive list of wet-processed blue fluorescent OLED devices, including the publication year, device structure, employed host, key dopant, doping concentration, HOMO, LUMO, EQE, PE, CE, CIE (X), CIE (Y), and reference. The years span from 2006 to 2020. A complete set of efficiencies at 100 and/or 1000 cd/m^2^ and/or the maximum efficiency are reported for deep-blue and blue emission.

### 5.2. Phosphorescent Blue OLEDs

#### 5.2.1. EQE vs. CIE_y_

**Figure 13:**
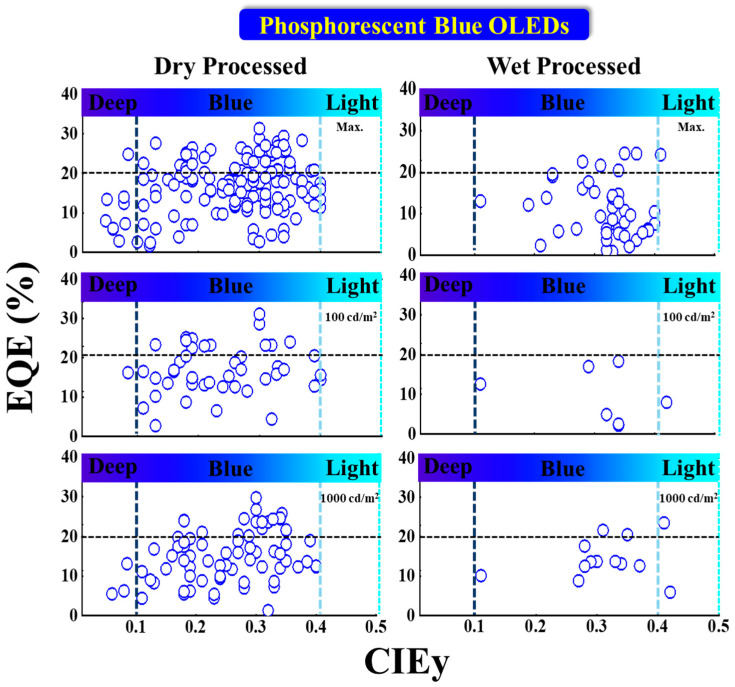
EQE performance vs. CIE_y_ of phosphorescent blue OLEDs with dry and wet processes. Some deep-blue devices were reported with a greater than 20% EQE at a maximum luminance dimmer than 100 nits with a CIE_y_ less than 0.1 by using dry processes. The horizontal black dashed lines represent the theoretical upper limit. The vertical dark and light blue dashed lines represent the region for deep blue and greenish blue, respectively.

Figure 13 shows the EQE performance vs. CIE_y_ of phosphorescent blue OLEDs with dry and wet processes. The horizontal black dashed lines represent the 20% theoretical upper limit. The vertical dark and light blue dashed lines represent the region for deep blue and greenish blue, respectively.

Some deep-blue devices were reported with a greater than 20% EQE at a maximum luminance dimmer than 100 nits with a CIE_y_ less than 0.1 by using dry-process. Park et al. reported an EQE_max_ of 25% for a deep-blue emission with coordinates of (0.14, 0.08) using a TSP01 emitter doped in a mer-Ir1 host. The same report published a 17 and 13% EQE at 100 and 1000 cd/m^2^, respectively [86].

Sarma et al. reported a highest EQE_max_ of 31% for a blue emission with CIE coordinates of (0.15, 0.3) using an 8 wt% MCPCN emitter. The same report published a 31 and 28% EQE at 100 and 1000 cd/m^2^, respectively [87].

For wet processes, none of the deep-blue devices shows an EQE greater than 20%. Li et al. published a highest EQE_max_ of 25% for a bluish-green emission at CIE coordinates of (0.15, 0.35) using a 5 wt% FIrpic doped in an m-CzPyPz host [88].

At 100 nits, no device shows a deep-blue emission or an EQE greater than 20%. However, Kim et al. reported a device close to the theoretical limit with an EQE of 18% at 100 cd/m^2^ for a bluish-green emission at CIE coordinates of (0.18, 0.34) using a 3 wt% fac-tris(2,3-dimethylimidazo[1,2-f]phenanthridinato-k2 C, N)iridium(III)] emitter [89].

At 1000 cd/m^2^, none of the devices shows a deep-blue emission. Nevertheless, Seok Oh et al. published a 24% EQE for a bluish-green emission with coordinates of (0.19, 0.41) using a 10 wt% Ir(dbi)_3_ doped in an mCBP-CN host [90].

#### 5.2.2. Power Efficacy vs. CIE_y_

**Figure 14:**
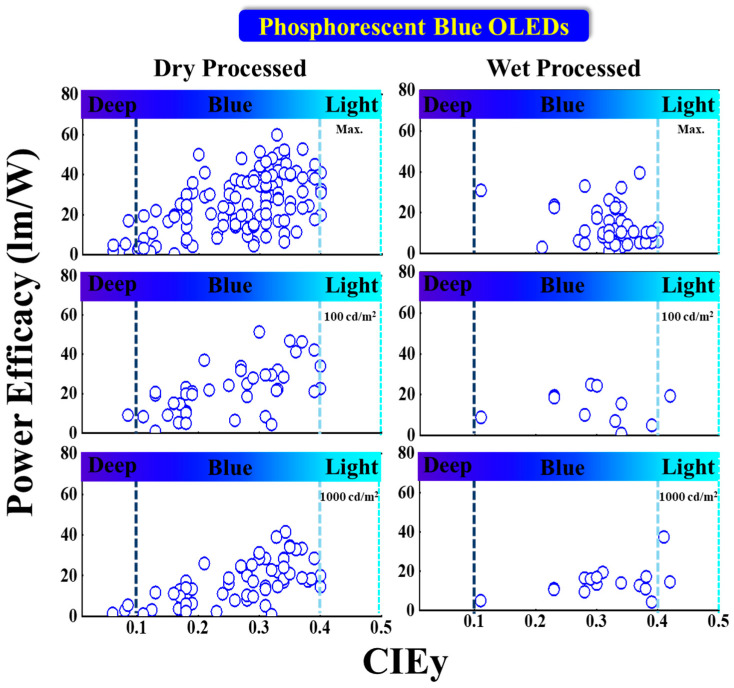
Power efficacy performance vs. CIE_y_ of phosphorescent blue OLEDs with dry and wet processes. No deep-blue devices show a power efficacy greater than 10 lumens per watt at 100 or 1000 nits. The vertical dark and light blue dashed lines represent the region for deep blue and greenish blue, respectively.

Figure 14 shows the power efficacy performance vs. CIE_y_ of fluorescent blue OLEDs with dry and wet processes. No deep-blue devices show a power efficacy greater than 10 lumens per watt at 100 or 1000 nits. The vertical dark and light blue dashed lines represent the region for deep blue and greenish blue, respectively.

For dry-processed devices, Park et al. reported a 17 lm/W PE_max_ for a deep-blue emission with CIE_xy_ (0.14, 0.08) using a TSP01 emitter doped in a mer-Ir1 host. The same device showed a 9.1 and 5.5 lm/W PE at 100 and 1000 cd/m^2^, respectively [86]. Ying et al. reported a highest PE_max_ of 60 lm/W for a bluish-green emission with CIE_xy_ (0.14, 0.32) using a 10 wt% FIrpic doped in an mCBP:PO-T2T (host:co-host) [91].

Meanwhile, Sarma et al. reported a PE of 51 lm/W at 100 cd/m^2^ for a bluish-green emission with CIE coordinates of (0.15, 0.3) by using an 8 wt% mCPCN emitter doped in an MS19 host [87]. Furthermore, Son et al. reported a 42 lm/W PE at 1000 cd/m^2^ for a bluish-green emission with CIE_xy_ (0.16, 0.34) by using a 6 wt% FIrpic doped in a TATA host [92].

Among the wet-processed devices, none shows a deep-blue emission. However, Feng et al. reported a device close to a deep-blue region with a PE_max_ of 11 lm/W at CIE coordinates of (0.14, 0.11) by using a 10 wt% Ir2 doped in a t-BuCPO host. The same device showed a PE of 8.8 and 5 lm/W at 100 and 1000 cd/m^2^, respectively [93].

Meanwhile, Seok Oh et al. reported a highest PE_max_ of 57 lm/W for a bluish-green emission with coordinates of (0.19, 0.41) using an Ir(dbi)_3_ emitter doped in an mCBP-CN host. The same device showed a 37 lm/W PE at 1000 cd/m^2^ [90]. Moreover, Jou et al. reported a PE of 26 lm/W at 100 cd/m^2^ for a bluish-green emission with coordinates of (0.18, 0.34) using an FIrpic emitter doped in a CBP host [94].

#### 5.2.3. Current Efficacy vs. CIE_y_

**Figure 15:**
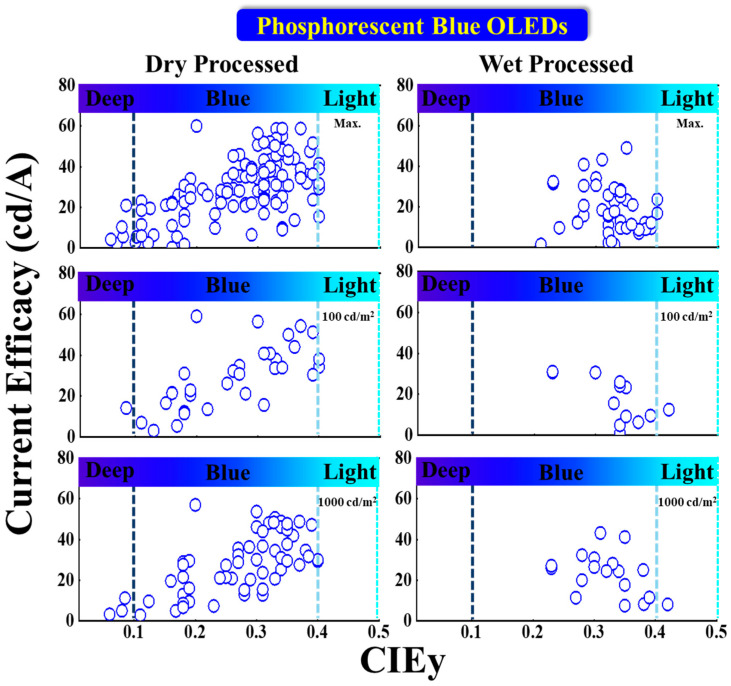
Current efficacy (CE) performance vs. CIE_y_ for dry- and wet-processed phosphorescent blue OLEDs. Many dry-processed devices exhibit a CE much greater than 70*CIE= in the typical blue region, while only a few exhibits deep-blue emission. The black dashed lines represent the minimum CE requirement for display. The vertical dark and light blue dashed lines represent the region for deep blue and greenish blue, respectively.

Figure 15 shows the current efficacy (CE) performance vs. CIE_y_ for dry- and wet-processed phosphorescent blue OLEDs. The black dashed lines represent the minimum CE requirement for display. The vertical dark and light blue dashed lines represent the region for deep blue and greenish blue, respectively.

Many dry-processed devices exhibit a CE much greater than 70*CIE_y_ in the typical blue region, while only a few exhibit deep-blue emission. Among these, Park et al. reported a commercially viable device with a CE_max_ of 21 cd/A for a deep-blue emission at (0.14, 0.08) by using a mer-Ir1 emitter doped in a TSP01 host. The same device showed a 14 and 11 cd/A CE at 100 and 1000 cd/m^2^, respectively [86].

Wu et al. also reported a commercially viable device with a CE_max_ of 59 cd/A for a bluish-green emission with CIE_xy_ (0.13, 0.37) by using a 10 wt% DPP-2 emitter doped in a 26DCzPPy host [95]. Sarma et al. also reported a commercially viable device with a 56 cd/A CE at 100 cd/m^2^ for a bluish-green emission at (0.15, 0.30) by using an 8 wt% MS19 emitter doped in an mCPCN host. The same device showed a 54 cd/A CE at 1000 cd/m^2^ [87].

For wet-processes, no devices met the criteria to be commercially viable in the deep-blue region. However, in the typical blue region, many wet-processed devices met the criteria. Among these, Li et al. reported a CE_max_ of 49 cd/A for a bluish-green emission with coordinates of (0.15, 0.35) by using a 5 wt% FIrpic emitter doped in an m-CzPyPz host. The same device showed a 41 cd/A CE at 1000 cd/m^2^ [88]. Moreover, Wee et al. reported a 31 cd/A CE at 100 cd/m^2^ for a greenish-blue emission at CIE_xy_ (0.15, 0.23) by using a 10 wt% FIr6 emitter doped in an Opt-MCBP host [96].

#### 5.2.4. Lifetime and CE vs. CIE_y_

**Figure 16:**
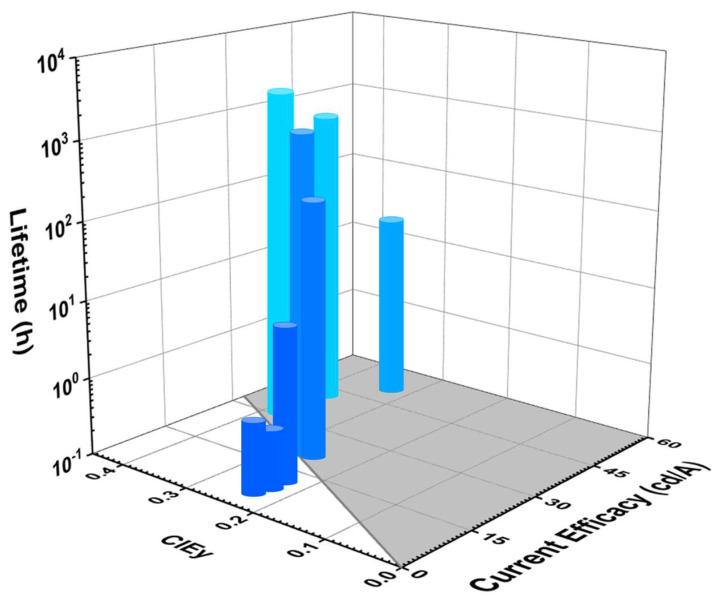
Lifetime and current efficacy vs. CIE_y_ for phosphorescent blue OLEDs. Some phosphorescent OLEDs show relatively high current efficacy and comparatively long lifetime in the sky-blue region. Those that fall into the grey area are plausibly commercially viable. The shaded area represents a CE ≥ 70*CIE_y_ [30,37,41,42,43]. The different shades of blue denote the emission-color at CIE_y_.

Figure 16 shows the lifetime and current efficacy vs. CIE_y_ for phosphorescent blue OLEDs. Those that fall on the grey area are plausibly commercially viable. The shaded area represents a CE ≥ 70*CIE_y_.

None of the deep-blue fluorescent devices met the commercially viable criteria. However, Sarma et al. reported a commercially viable device close to the deep-blue region with a lifetime of 2200 h and a CE_max_ of 43 cd/A corresponding to a CIE_y_ of 0.16 by using an 8 wt% mCPCN emitter doped in an MS19 host [87].

Some phosphorescent OLEDs show relatively high current efficacy and a comparatively long lifetime in the sky-blue region. Among these, Weaver et al. reported a highest lifetime of 100,000 h and a CE_max_ of 32 cd/A corresponding to a CIE_y_ of 0.38 by using a 6 wt% FIrpic doped in a CBP host [97]. Meanwhile, Konidena et al. reported a highest CE_max_ of 52 cd/A and a lifetime of 20 h corresponding to a CIE_y_ of 0.32 by using a 3CNCzBN emitter [98].

Table 3 shows the efficiency and lifetime performance of dry-processed phosphorescent blue OLEDs. All the lifetime data have also been converted to the same 1000 nits for easy comparison.

No devices show a deep-blue emission. However, Jung et al. reported a device close to deep-blue emission with a highest EQE_max_ of 28% and a lifetime of 10,000 h (LT_50_) at 100 cd/m^2^ corresponding to a CIE_y_ of 0.13. A lifetime of 2400 h, estimated as the initial brightness, is converted to 1000 cd/m^2^ [99].

Weaver et al. reported a highest lifetime 100,000 h (LT_50_) at 200 cd/m^2^, an EQE_max_ of 14%, and a CE_max_ of 32 cd/A corresponding to a CIE_y_ of 0.38. At 1000 cd/m^2^_,_ the same device is estimated to have a 37,000 h (LT_50_) of lifetime [97].

Table S3 shows a comprehensive list of dry-processed blue phosphorescence OLED devices, including the publication year, device structure, key dopant, doping concentration, employed host, HOMO, LUMO, EQE, PE, CE, CIE (X), CIE (Y), and reference. The years span from 2001 to 2020. A complete set of reported efficiencies (maximum at 100 and 1000 cd/m^2^) corresponding to the device composition are displayed for deep-blue and blue emission.

Table S4 shows a comprehensive list of dry-processed blue phosphorescence OLED devices, including the publication year, device structure, key dopant, doping concentration, employed host, HOMO, LUMO, EQE, PE, CE, CIE (X), CIE (Y), and reference. The years span from 2001 to 2020. A complete set of reported efficiencies (maximum at 100 and 1000 cd/m^2^) corresponding to the device composition are displayed for deep-blue and blue emission.

### 5.3. TADF-Based Blue OLEDs

#### 5.3.1. EQE vs. CIE_y_

**Figure 17:**
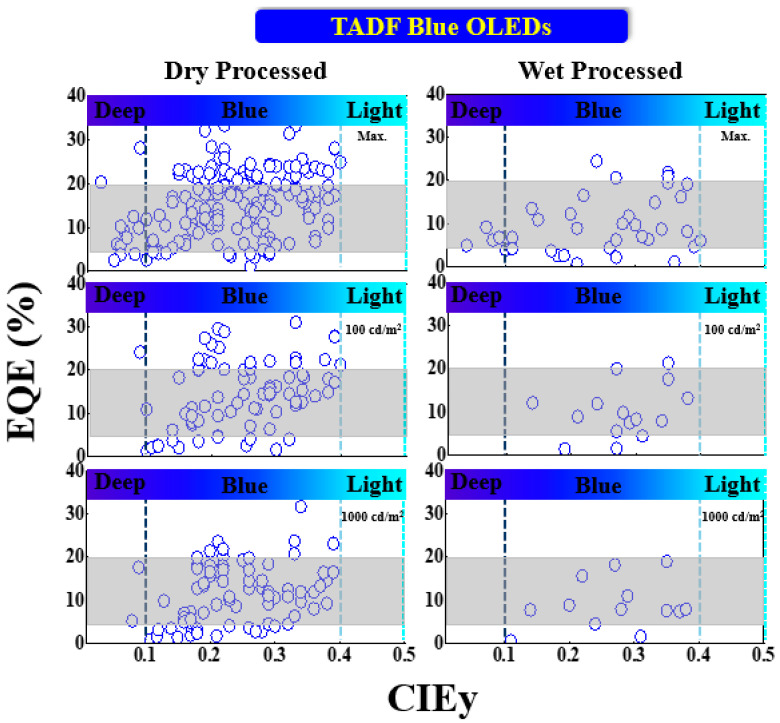
EQE performance vs. CIE_y_ of TADF blue OLEDs with dry and wet processes. Some deep-blue devices were successfully fabricated with an EQE greater than 20% via dry-process. The shaded area represents the upper and lower limits expected for a TADF-based device. The vertical dark and light blue dashed lines represent the region for deep blue and greenish blue, respectively.

Figure 17 shows the EQE performance vs. CIE_y_ of TADF blue OLEDs with dry and wet processes. The shaded area represents the upper and lower limits expected for a TADF-based device. The vertical dark and light blue dashed lines represent the region for deep blue and greenish blue, respectively.

Some deep-blue devices were successfully fabricated with an EQE greater than 20% via a dry process. Among these, Liang et al. reported an EQE_max_ of 20% with CIE coordinates of (0.16, 0.03) by using an NTN-PCZ host-free emitter [104]. Meanwhile, Lim et al. reported a highest EQE_max_ of 28%, exceeding the 20% theoretical limit, with a CIE_xy_ (0.14, 0.09) by using a 20 wt% TDBA-SAF emitter doped in a DPEPO host. The same device showed a 24 and 18% EQE at 100 and 1000 cd/m^2^, respectively [105].

Moreover, some blue devices achieved an EQE much greater than 20%. Among these, Li et al. reported a highest EQE_max_ of 33% for a bluish-green emission at (0.17, 0.33) by using a 30 wt% TspiroS-TRZ emitter doped in a DPEPO host. The same device showed a 31 and 24% EQE at 100 and 1000 cd/m^2^, respectively [106]. Furthermore, Miwa et al. reported a 33% EQE_max_ for a greenish-blue emission with CIE_xy_ (0.16, 0.22) by using a CCX-I-6B-OC emitter doped in a PPF host [107].

For wet-processed devices, Kim et al. reported an EQE_max_ of 9.2% for a deep-blue emission at (0.17, 0.07) by using a TB-3Cz host-free emitter [108]. None of the devices show a deep-blue emission at 100 or 1000 cd/m^2^. Meanwhile, Xie et al. reported a 25% EQE_max_ for a greenish-blue emission with coordinates of (0.16, 0.24) by using a 2tCz2CzBn host-free emitter. The same device showed a 12 and 4.5% EQE at 100 and 1000 cd/m^2^, respectively [109].

#### 5.3.2. PE vs. CIEy

**Figure 18:**
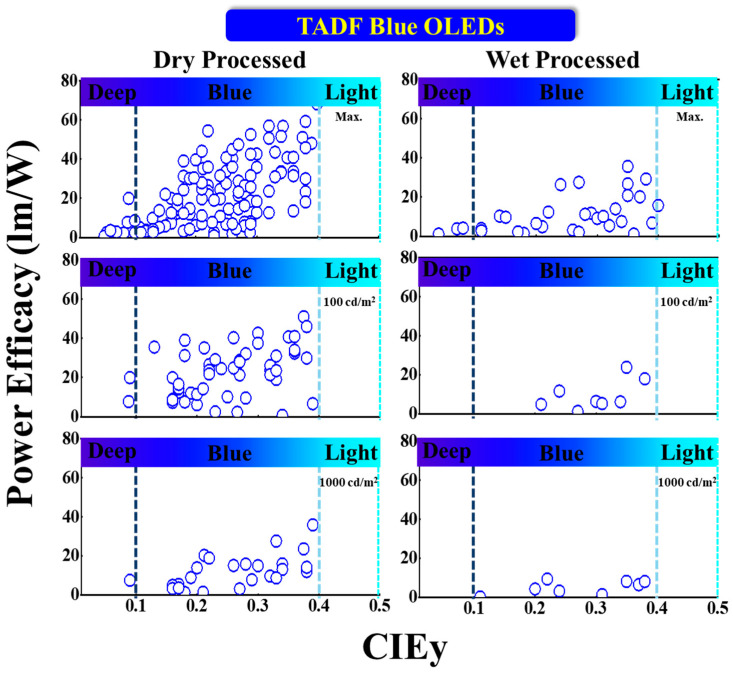
Power efficacy performance vs. CIE_y_ of TADF blue OLEDs with dry and wet processes. One device shows an around 20 lm/W power efficacy at 100 nits with deep blue emission. The vertical dark and light blue dashed lines represent the region for deep blue and greenish blue, respectively.

Figure 18 shows the power efficacy performance vs. CIEy of TADF blue OLEDs with dry and wet processes. The vertical dark and light blue dashed lines represent the region for deep blue and greenish blue, respectively.

One device shows around 20 lm/W power efficacy at 100 nits with deep-blue emission via dry-processing. Lim et al. reported a highest PE_max_ of 20 lm/W with coordinates of (0.14, 0.09) by using a 20 wt% TDBA-SAF emitter doped in a DPEPO host. The same device showed a 14 and 7.4 lm/W PE at 100 and 1000 cd/m^2^, respectively [105].

Meanwhile, Ganesan et al. reported a highest PE_max_ of 68 lm/W for a bluish-green emission with CIE_xy_ (0.18, 0.34) by using a 22 wt% 2NPMAF emitter doped in a DPEPO host. The same device showed a 46 lm/W PE at 100 nits [110]. Moreover, Oh et al. reported a PE of 44 lm/W at 1000 cd/m^2^ for a bluish-green emission at (0.17, 0.33) by using a 30 wt% 23CT emitter doped in a DPEPO host [111].

For wet-processed devices, Kim et al. reported a PE_max_ of 4.1 lm/W for a deep-blue emission with CIE_xy_ (0.15, 0.08) by using a TB-P3Cz host-free emitter [108]. None of the devices shows a deep-blue emission at 100 and 1000 nits.

Meanwhile, Ban et al. reported a PE_max_ of 36 lm/W for a bluish-green emission at (0.17, 0.35) by using a MeCz-4CzCN host-free emitter [112]. Furthermore, Xie et al. reported a PE of 24 lm/W at 100 cd/m^2^ for a bluish-green emission with CIE_xy_ (0.18, 0.35) by using a 2PhCz2tCzBn host-free emitter. The same device showed an 8.1 lm/W PE at 1000 nits [109].

#### 5.3.3. CE vs. CIE_y_

**Figure 19:**
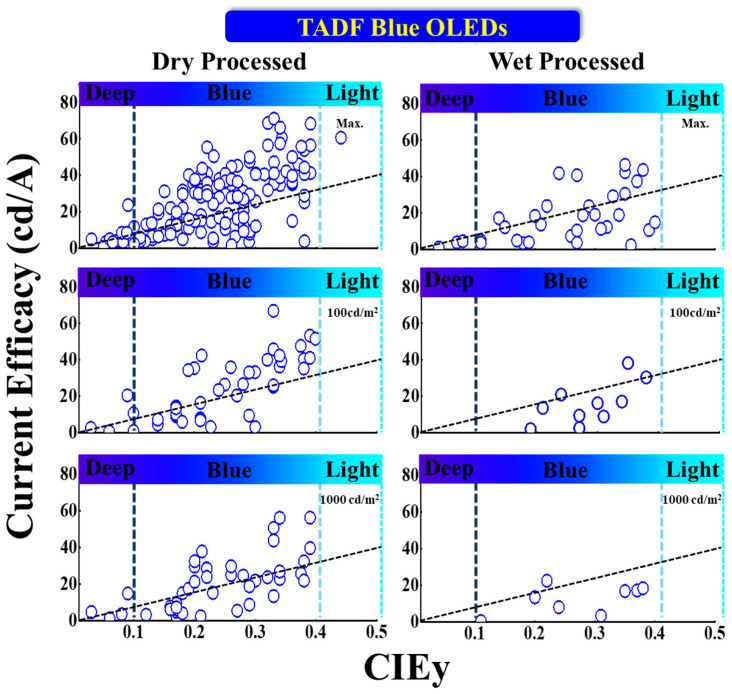
Current efficacy (CE) performance vs. CIE_y_ for dry- and wet-processed TADF blue OLEDs. One dry-processed device exhibits a near 25 cd/A maximum current efficacy with relatively deep-blue emission. Greater than 20 cd/A is also reported at 100 nits. The black dashed lines represent the minimum CE requirement for display. The vertical dark and light blue dashed lines represent the region for deep blue and greenish blue, respectively.

Figure 19 shows the current efficacy (CE) performance vs. CIE_y_ for dry- and wet-processed TADF blue OLEDs. The black dashed lines represent the minimum CE requirement for display. The vertical dark and light blue dashed lines represent the region for deep blue and greenish blue, respectively.

One dry-processed device exhibits a near 25 cd/A CE_max_ with relatively deep-blue emission. Greater than 20 cd/A is also reported at 100 nits. Lim et al. reported a commercially viable device with a CE_max_ of 24 cd/A for a deep-blue emission at (0.14, 0.09) by using a 20 wt% TDBA-SAF emitter doped in a DPEPO host. The same device showed a 21 and 15 cd/A CE at 100 and 1000 cd/m^2^, respectively [105].

Meanwhile, Li et al. reported two commercially viable devices with a CE_max_ of 71 cd/A for a bluish-green emission at (0.17, 0.33) by using a 30 wt% TspiroS-TRZ emitter doped in a DPEPO host. The same device showed a 70 cd/A CE at 100 cd/m^2^. Another device showed a 56 cd/A CE at 1000 cd/m^2^ for a bluish-green emission at (0.19, 0.39) by using a 30 wt% TspiroF-TRZ emitter doped in a DPEPO host [106].

For wet-processed devices, none of the deep-blue devices met the criteria to be commercially viable. However, Kim et al. reported a device close to the criteria with a CE_max_ of 4.6 cd/A at (0.15, 0.08) by using a TB-P3Cz host-free emitter [108]. None of the devices show a deep-blue emission at 100 or 1000 nits.

Nonetheless, Xie et al. reported two commercially viable devices with a CE_max_ of 44 cd/A for a bluish-green emission at (0.19, 0.38) by using a 2tCz2PhCzBn host-free emitter. The same device showed an 18 cd/A CE at 1000 nits. Moreover, another device showed a CE of 38 cd/A at 100 nits for a bluish-green emission at (0.18, 0.35) by using a 2PhCz2tCzBn host-free emitter [109].

#### 5.3.4. Lifetime and CE vs. CIE_y_

**Figure 20:**
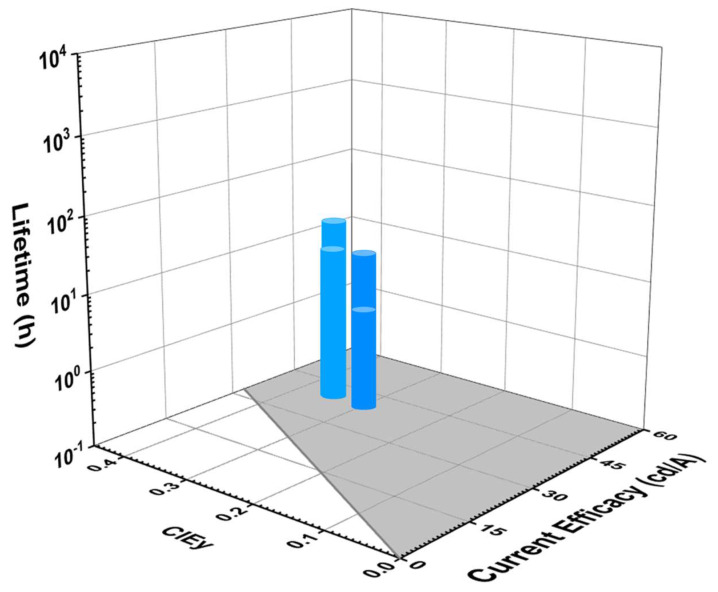
Lifetime and current efficacy vs. CIEy for TADF blue OLED. A couple TADF-based OLEDs show a fair lifetime with high current efficacy in the sky-blue region. The shaded area represents a CE ≥ 70*CIEy [80].

Figure 20 shows the lifetime and current efficacy (CEs) vs. CIEy for TADF blue OLEDs. The shaded area represents a CE ≥ 70*CIE_y_. None of the devices show a lifetime even close to a deep-blue region. However, a couple TADF-based OLEDs show a fair lifetime with high current efficacy in the sky-blue region. Yoon et al. reported a lifetime of 24 h and CE_max_ of 42 cd/A corresponding to a CIE_y_ of 0.35 by using a 30 wt% DBA-BI emitter doped in a pCzPybCz host [113].

Table 4 shows the efficiency and lifetime performance of dry-processed TADF blue OLEDs. All the lifetime data have also been converted to the same 1000 nits for easy comparison.

None of the devices shows a lifetime in the deep-blue region. However, Su et al. reported a device close to the deep-blue region with a lifetime of 95 h (LT_50_) at 1000 nits and an EQE_max_ of 12% corresponding to a CIEy of 0.19 by using a BCz-TRZ emitter doped in an ICz-TRZ host. The same report showed a highest lifetime of 495 h (LT_50_) at 1000 nits and an EQE_max_ of 20% corresponding to a CIEy of 0.3 by using a BCz-TRZ emitter doped in a PYD2 host [114].

**Table 4:** Efficiency and lifetime performance of dry-processed TADF blue OLEDs. All the lifetime data have been converted to the same 1000 nits for easy comparison.

CIEy	EQE_max_ (%)	L_0_ (cd/m^2^)	Lifetime (h)	Lifetime (h) @L_0_:1000 (cd/m^2^)	CE_max_ (cd/A)	Year	Reference
0.19	11.8	1000	95 (LT_50_)	95 (LT_50_)	-	2020	[17,115,116]
0.2	10	1000	45 (LT_50_)	45 (LT_50_)	-	2020	
0.23	18.1	1000	174 (LT_50_)	174 (LT_50_)	-	2020	
0.24	17.3	1000	72 (LT_50_)	72 (LT_50_)	-	2020	
0.26	13.6	500	21 (LT_80_)	13.6 (LT_80_)	-	2017	
0.3	24	1000	5 (LT_95_)	5 (LT_95_)	40.7	2020	[6,113]
0.3	24	1000	12 (LT_90_)	12 (LT_90_)	40.7	2020	
0.3	19.6	1000	495 (LT_50_)	495 (LT_50_)	-	2020	[115]
0.34	17.1	500	0.28 (LT_80_)	0.18 (LT_80_)	-	2017	
0.35	22.7	1000	11 (LT_95_)	11 (LT_95_)	42.2	2020	[113]
0.35	22.7	1000	24 (LT_90_)	24 (LT_90_)	42.2	2020	
0.35	17.3	500	3 (LT_90_)	1.9 (LT_90_)	-	2020	[45,117]
0.35	17.3	500	19.1 (LT_75_)	12.4 (LT_75_)	-	2020	
0.35	14.8	500	2.4 (LT_90_)	1.5 (LT_90_)	-	2020	
0.35	14.8	500	14.1 (LT_75_)	9.1 (LT_75_)	-	2020	
0.35	12.9	500	2.8 (LT_90_)	1.8 (LT_90_)	-	2020	
0.35	12.9	500	19.9 (LT_75_)	12.9 (LT_75_)	-	2020	
0.35	12.2	500	1.7 (LT_90_)	1.1 (LT_90_)	-	2020	
0.35	12.2	500	9.9 (LT_75_)	6.4 (LT_75_)	-	2020	

Table S5 shows a comprehensive list of dry-processed blue TADF OLED devices, including the publication year, device structure, key dopant, doping concentration, employed host, HOMO, LUMO, EQE, PE, CE, CIE (X), CIE (Y), and reference. The years span from 2015 to 2020. A complete set of reported efficiencies (maximum at 100 and 1000 cd/m^2^) corresponding to the device composition are displayed for deep-blue and blue emission.

Table S6 shows a comprehensive list of wet-processed blue TADF OLED devices, including the publication year, device structure, key dopant, doping concentration, employed host, HOMO, LUMO, EQE, PE, CE, CIE (X), CIE (Y), and reference. The years span from 2016 to 2020. A complete set of reported efficiencies (maximum at 100 and 1000 cd/m^2^) corresponding to the device composition are displayed for deep-blue and blue emission.

## 6. Chemical Structures and Characteristics of Blue Emitters

As mentioned earlier, the materials can be classified into three categories; fluorescent, phosphorescent, and TADF, which can be further sub-categorized according to their derivatives. This section describes the historical development and current advancement of fluorescent, phosphorescent, and TADF blue emitters based on their structural importance, color purity, and efficiency.

### 6.1. Fluorescent Type

**Figure 21:**
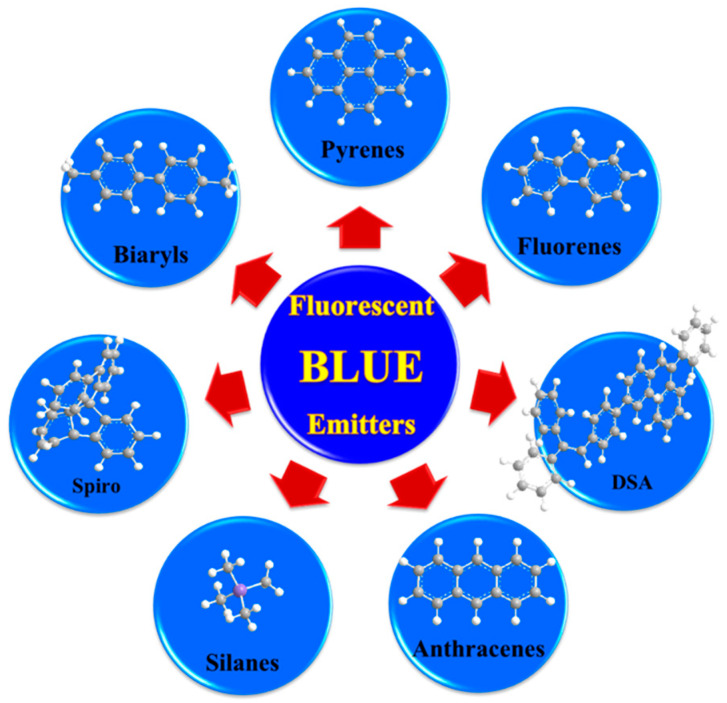
Numerous series of fluorescent blue emitters based on different core structure units.

Over the past two decades, researchers have developed various kinds of blue-fluorescent emitters, such as distyrylarylene (DSA), spiro-shaped, biaryl, silane, and polycyclic aromatic hydrocarbon (e.g., pyrene, anthracene, carbazole, fluorene, etc.) derivatives to enhance the performance of blue OLEDs. Among them, polycyclic aromatic hydrocarbon (PAH)-based small molecules, oligomers, starburst, tetrahedral, dendrimer derivatives, and long-chain polymers are extensively applicable as emitters and/or host materials in OLED devices. In this section, we comprehensively review nine series of fluorescent-type blue-light emitting materials (Figure 21).

**Figure 22:**
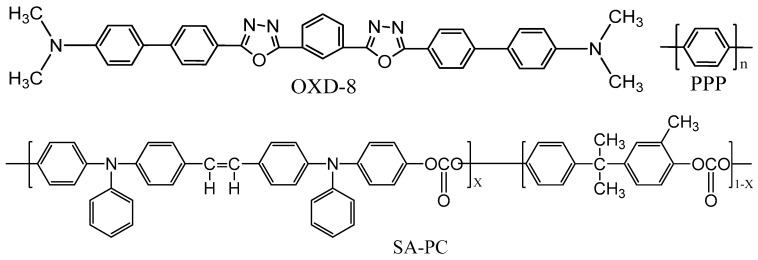
Chemical structures of some typical blue emitters, OXD-8, PPP, and SA-PC, for OLED devices.

In 1992, Hamada et al. reported a multi-layer blue OLED device employing an oxadiazole derivative, 1,3-bis(N,N-dimethylaminophenyl)-1,3,4-oxadiazole (OXD-8), as an emitter for the first time (Figure 22). It showed a maximum luminance of over 1000 cd/m^2^ and an emission peaking at 470 to 480 nm [118,119]. In the same year, Leising et al. reported an EQE of 0.05% for a single-layer blue OLED device using poly(p-phenylene) (PPP) (Figure 14) [120,121].

Hosokawa et al. also observed a blue emission for a non-conjugated polymer-based emitter with an electron donor polycarbonate-containing styrylamine repeating unit (SA-PC) (see Figure 14) in a multilayered device. The power efficacy was 0.05 lm/W with a peak emission at 460 nm [122].

#### 6.1.1. Distyrylarylene (DSA)

Over the last twenty years, researchers have extensively investigated the electroluminescent characteristics of DSA, DSA-ph, and their numerous derivatives as blue-light emitting materials in blue OLED devices [122,123,124,125,126]. In 1990, Hosokawa et al. measured light-emitting characteristics of DSA derivatives and reported that the exciplex formation or charge transfer complex with a hole-transporting layer drastically influenced device performance [123].

**Figure 23:**
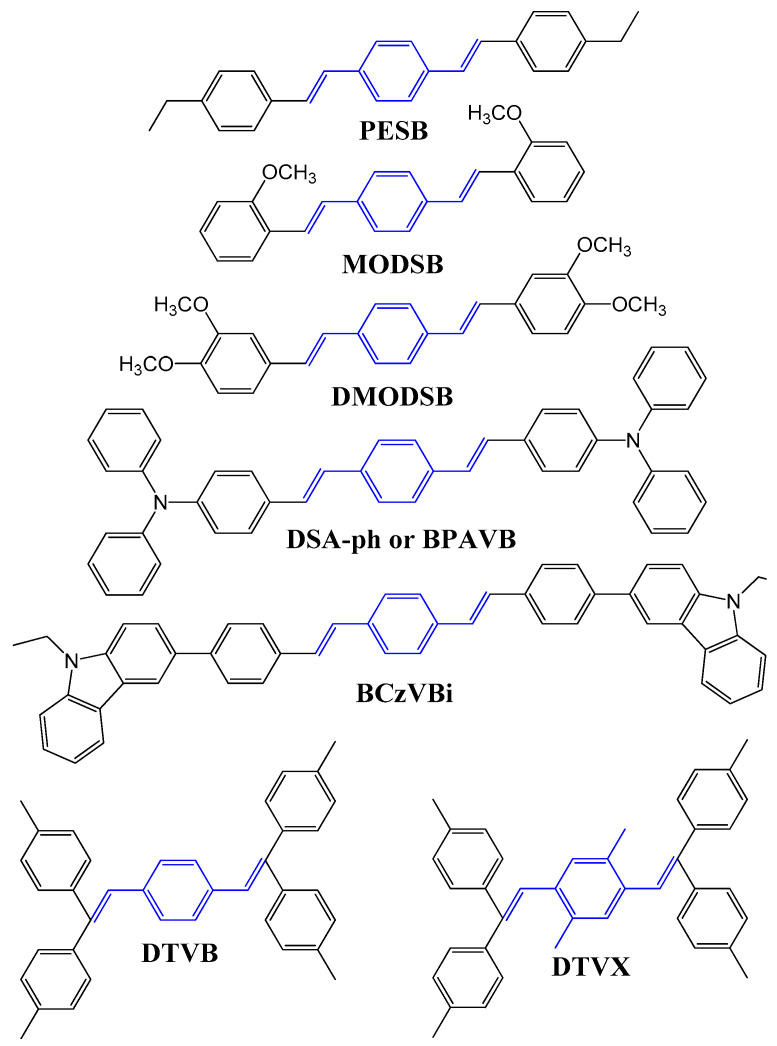
Molecular structures of typical DSA derivative-based blue emitters with electron donor groups.

Researchers at Idemitsu Kosan Co. Ltd. reported that the formation of exciplex or charge transfer complexes can be avoided by introducing electron donor groups, such as ethyl, methoxy, diethylamine, phenylamine, and N-ethyl-carbazole, in DSA derivatives (Figure 23).

**Figure 24:**
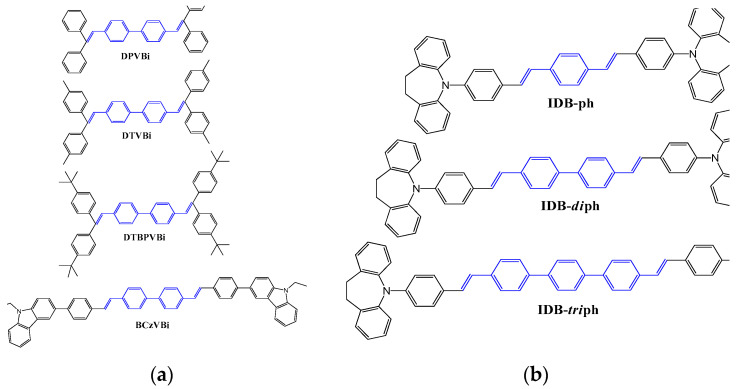
Molecular structures of non-planar (**a**) DSA diphenyl and (**b**) IDB phenyl, diphenyl, and triphenyl derivative-based blue emitters.

Later in 1993, Tokailin et al. reported a series of nonplanar DSA derivatives with different DSA cores (Figure 24), namely, DTVB and DTVX, and different alkyl group substituents at the end of the molecule, namely, DPVBi, DTVBi, and DTBPVBi, as blue emitters. They also investigated the electroluminescence characteristics of these emitters in a multi-layer OLED device (ITO as anode; TDP as HTL; DPVBi or DTVBi or DTBPVBi as EML; Alq as ETL; Mg:Ag as cathode). The OLED device fabricated with a DPVBi emitter exhibited, at 1000 cd/m^2^, a power efficacy of 0.6 lm/W and maximum luminance of 2280 cd/m^2^, with blue emission peaking at 475 nm. The EL spectra did not exhibit any emission in the longer wavelength region, supporting that both DPVBi and DTVBi had entirely avoided the formation of exciplex or charge transfer complexes [127].

High device efficiencies and long operation lifetimes were realized in 1995 by doping a fluorescent styrylamine-based guest, BCzVB, into a DSA derivative, DPVBi, host material. Hosokawa et al., in 1995, utilized a DDPVBi as a blue host material and styrylamine (known as BCzVB) as a dopant [126]. Further improvement of this system was later published, in which the current efficacy reached 10.2 cd/A at 1.89 mA/cm^2^ with 1931 CIE coordinates of (0.17, 0.33) and a half-life of 20,000 h at an initial brightness (L_o_) of 100 cd/m^2^. When oligoamine was used as a hole-injection layer for this device, the operational lifetime was further improved to 10,000 h with L_o_ = 500 cd/m^2^ [84]. It appears that the sky-blue emission was specifically designed by Idemitsu Kosan for the application of color-changing media (CCM) technology for full-color organic EL devices [128].

For a 3 wt% DSA-ph emitter doped with a 2-methyl-9,10-di(2-napthyl)anthracene (MADN) host, the device exhibited a half-decay lifetime of 46,000 h (LT_50_) at an initial brightness of 100 cd/m^2^. The resultant OLED also showed a much higher power efficacy of 5.5 lm/W and a current efficacy of 9.7 cd/A at 20 mA/cm^2^ with a sky-blue emission (0.16, 0.32) [84].

In 2007, Ho et al. reported a new series of emitters based on a seven-membered N-hetero-cyclic core structure of iminodibenzyl-distyrylarylene (IDB). In the distyrylarylene structure, a replacement of the amino group by an iminodibenzyl group improved the thermal properties (T_g_ varying between 119 and 137 °C) and slightly blue-shifted the emission with increasing phenyl units. The emission wavelength for DSA-ph is 458 nm, while it was 449, 447, and 443 nm for IDB-ph, IDB-*di*ph, and IDB-*tri*ph, respectively [129].

In 2018, Wanshu Li et al. reported a wet-processed simple structure and high efficiency OLED using a DSA-Ph emitter doped in an MADN host. In this work, MADN is also used as a hole-transporting material (HTL). At 3 wt% dopant concentration, a 10% EQE_max_ was reported at CIE coordinates of (0.15, 0.28). Moreover, a high CE of 15 cd/A and 13 lm/W PE was obtained [76].

#### 6.1.2. Pyrenes

In the last fifteen years, numerous pyrene derivatives and hybrid-based blue-emitters were investigated in OLED devices due to their rigid planar structure [130,131], high thermal stability [70,132,133], high fluorescence quantum yield [134,135,136,137,138], long fluorescent lifetime [139,140], and effective carrier mobility [141,142].

However, the utilization of pyrene-based blue emitters was limited, owing to their strong tendency to form excimers and π-aggregates, which would cause red shifts and quenching of fluorescence, resulting in a lower solid-state PL quantum yield and quantum efficiency. Numerous efforts in the structural alteration of pyrene-based compounds have hence been made to enhance the PL quantum yield and diminish the aggregation characteristics.

In recent years, pyrene derivatives were extensively employed in efficient blue OLEDs, including polypyrenes [134,143,144], dipyrenylbenzene [145], alkynylpyrene [146,147], pyrene-functionalized calix[4]arenes [148], aryl-functionalized pyrenes [116,136,149,150,151], as well as oligothiophenes with pyrenyl side groups [152], pyrene-carbazole [132,135,146,153,154], pyrene-fluorene [155,156,157,158,159,160,161,162,163], pyrene-triphenylamine [142,164,165], and pyrene-anthracene [166] hybrids, due to their good emissive characteristics and hole mobility.

In 2011, Figueira-Duarte and Müllen comprehensively reviewed the synthesis techniques for numerous types of small molecular pyrene, oligo-pyrene, pyrene-dendrimer, and polypyrenes. They also reviewed the thermal, electrochemical, photophysical, and optical characteristics of all pyrene-based emitters and their electroluminescence characteristics in OLED devices [167].

**Figure 25:**
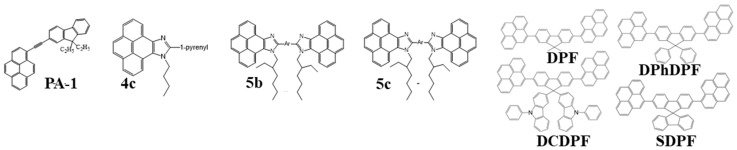
Molecular structures of pyrene-based blue emitters.

In this part, we have only covered the pyrene derivatives that were reported in the past few years. The Sonogashira, Sonogashira-Hogihara, Suzuki, Suzuki-Miyaura, Heck, and Yamamoto-coupling reactions have been extensively used to synthesize pyrene hybrid derivatives. The sky-blue emitter, 1-((9,9-diethyl-9H-fluorene-2yl)ethynyl)pyrene (PA-1) (Figure 25), was synthesized by the Sonogashira method via a cross-coupling reaction of 1-bromopyrene with 9,9-diethyl-2ethynyl-9H-fluorene by using a Pd(PPh_3_)_2_Cl_2_/PPh_3_/CuI catalytic system. The PA-1 exhibited a purplish-blue emission with CIE coordinates of (0.15, 0.06), a power efficacy of 1.2 lm/W, a current efficacy of 1.9 cd/A, and EQE of 3.5% at 100 cd/m^2^ as a 3 wt% sky-blue emitter doped into the polarity matching the CBP host [162].

A series of pyrenoimidazole-based emitters that possess markedly high thermal characteristics were synthesized in three simple steps. Firstly, pyrene-4,5-dione was prepared via the oxidation of pyrene in the presence of ruthenium trichloride and sodium periodate. Subsequently, the pyrene-4,5-dione was reacted with the corresponding aldehyde in the presence of ammonium acetate to form sparingly soluble pyrenoimidazoles. Finally, the alkylation reaction was either executed under phase transfer catalysis to synthesize mono-pyrenoimidazoles or by employing the potassium carbonate/dimethylformamide (K_2_CO_3_/DMF) to synthesize bis-pyrenoimidazoles. These rigid pyrenoimidazole moiety-based hybrid compounds offered a very high thermal stability with decomposition temperatures from 462 to 512 °C.

With a CBP host doped with a 3 wt% mono-pyrenoimidazole, 9-butyl-10-(pyrene-1-yl)-9H-pyreno[4,5-d]imidazole (Figure 25), the resultant multi-layer OLED device showed, at 100 cd/m^2^, for example, a power efficacy of 1.2 lm/W, a current efficacy of 2.4 cd/A, and an external quantum efficiency (EQE) of 2.2% with CIE coordinates of (0.15, 0.32). The resultant device also exhibited a maximum luminance of 2740 cd/m^2^ [168].

It is well established that pyrene-based emitters, either molecular or polymeric, possess a planar or partial planar molecular structure, which tends to show excimer emissions and lower quantum efficiencies, resulting in an extensive bathochromic shift in emission spectra owing to the π–π stacking of pyrene chromophore units in the solid state.

In 2012, Hu and Yamato et al. synthesized a series of pyrene-based Y-shaped blue emitters using the Suzuki cross-coupling reaction of 7-tert-butyl-1,3-dibromopyrene with p-substituted phenylboronic acids. These emitters realized a low degree of π-stacking due to the steric effect of the tert-butyl group in the pyrene ring at the 7-position [134,169].

In the search for new light-emitting compounds, Promarak et al. synthesized a series of solution-processable pyrene-functionalized carbazole derivatives, N-dodecyl-3,6-di(pyren-1-yl)carbazole (CP2), N-dodecyl-1,3,6-tri(pyren-1-yl)carbazole (CP3) and N-dodecyl-1,3,6,8-tetra(pyren-1-yl)carbazole (CP4) (Figure 25), by using the Suzuki-coupling reaction [170].

These pyrene-carbazole hybrids showed a glass-transition temperature and decomposition temperature ranging from 70 to 170 °C and 375 to 407 °C, respectively. Among the four pyrene-moieties containing carbazole compounds, CP4, showed the highest thermal stability (T_g_ = 170 °C and T_d_ = 407 °C).

However, these materials showed an undesirable donor-acceptor (DA) behavior because the carbazole central unit acts as a donor and the pyrene functional group acts as an acceptor. It is known in the literature that the DA molecular systems tend to extend π-conjugation and generate an intramolecular charge transfer (ICT) state, inducing an unwanted bathochromic shift in the search for a deep-blue emission.

An OLED device based on non-doped CP4 showed, by thermal evaporation deposition, for example, a maximum current efficacy of 6.7 cd/A and maximum luminance of 25,000 cd/m^2^ with CIE coordinates of (0.14, 0.26), and 2.5 cd/A and 9700 cd/m^2^ with CIE coordinates of (0.21, 0.28) via spin-coating [170].

#### 6.1.3. Anthracenes

**Figure 26:**
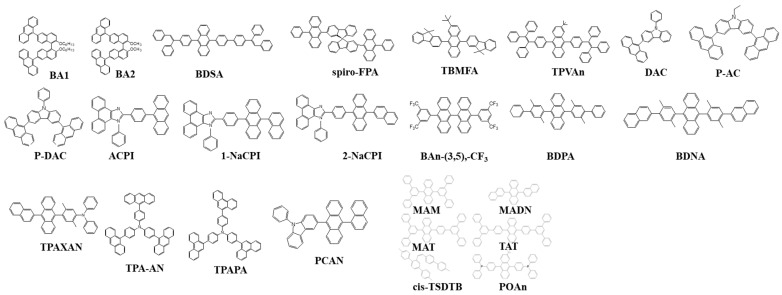
Molecular structures of anthracene-based blue emitters.

Adachi et al. reported a 9,10-diphenyl-anthracene (DPA) emitter with a 100% photoluminescence quantum yield (PLQY) [59]. The other major blue-doped emitter was developed by Shi et al. at Kodak in 2002 and utilized diphenylanthracene derivative 9,10-di(2-naphthyl)anthracene (ADN) (Figure 26) as a host and 2,5,8,11-tetra(t-butyl)-perylene (TBP) as a dopant to generate a blue emission of CIE (0.15, 0.23). The blue device showed a current efficacy of around 3.5 cd/A with a half-life of 4000 h at an initial light output of 700 cd/m^2^ [171].

In 2007, Shih et al. developed a blue-emitting material 2-tert-butyl-9,10-bis[4-(1,2,2-triphenylvinyl)phenyl]anthracene (TPVAn) (Figure 26) that contained an anthracene core and two tetraphenylethylene end-capped groups. The material possessed a high T_g_ of 155 °C and was morphologically stable due to the presence of the sterically congested terminal groups. A bright saturated-blue emission with CIE coordinates (0.14, 0.12) along with a 5.3% EQE crossing the theoretical limit of fluorescent materials was reported, the best performance among the non-doped blue devices [172].

In 2008, three materials 9,10-Bis(3′,5′-diphenylphenyl)anthracene [MAM], 9-(3′,5′-diphenylphenyl)-10-(3‴,5‴-diphenylbiphenyl-4″-yl)anthracene [MAT], and 9,10-bis(3″,5″-diphenylbiphenyl-4′-yl)anthracene [TAT] (Figure 26) were designed and emitted in the range of 439–445 nm. MAM and MAT have a T_g_ higher than 64 °C, while TAT has above 150 °C. The T_g_ for TAT is twice as high as the commonly used host material DPVBi (64 °C) and slightly higher than MADN (120 °C). The CIE coordinates were (0.15, 0.08) with an EQE of 7.2% [173].

In 2010, Zheng et al. reported a series of non-doped devices with three newly designed emitters, 2-tert-butyl-9,10-bis(9,9-dimethylfluorenyl) anthracene (TBMFA), 2-tert-butyl-9,10-bis[4-(2-naphthyl)phenyl] anthracene (TBDNPA), and 2-tert-butyl-9,10-bis[4-(9,9-dimethylfluorenyl)phenyl] anthracene (TBMFPA) (Figure 26), using naphthalene or 9,9-dimethylfluorene side units. Due to the anthracene unit, the resultant devices showed a deep-blue emission. These materials also showed a high T_g_ ≥ 133 °C, due to the presence of sterically congested terminal groups. The TBDNPA-based device outperformed the others by showing a low turn-on voltage of 3.0 V with an initial brightness of 1 cd/m^2^. The device displayed an EQE of 5.2% at 8.4 mA/cm^2^ with CIE_y_ < 0.1 [174].

In 2011, Kim et al. synthesized four molecules displaying blue emission using anthracene moieties. The material, 3-(anthracen-9-yl)-9-ethyl-9H-carbazole (AC), 3,6-di(anthracen-9-yl)-9-ethyl-9H-carbazole (DAC), 3-(anthracen-9-yl-)-9-phenyl-9H-carbazole (P-AC), and 3,6-di(anthracen-9-yl)-9-phenyl-9H-carbazole (P-DAC) (Figure 26), was also comprised of covalent carbazole units. Among these, the undoped device with P-DAC showed a near deep-blue emission at (0.16, 0.13) with a luminance efficacy of 3.1 cd/A and EQE of 2.8%. Moreover, P-DAC as a host material doped with a BDAVBi emitter displayed a 7.7 cd/A CE and 4.7% EQE with CIE coordinates of (0.15, 0.21) at 100 mA/cm^2^. A negligible efficiency roll-off is observed over a broad range of current density [175].

In 2012, Zhuang et al. reported a series of non-doped devices using emitters 2-(4-(anthracen-9-yl)phenyl)-1-phenyl-1H-phenanthro[9,10-d]imidazole (ACPI), 2-(4-(10-(naphthalen-1-yl)anthracen-9-yl)phenyl)-1-p-henyl-1H-phenanthro[9,10-d]imidazole (1-NaCPI), and 2-(4-(10-(naphthalen-2-yl)anthracen-9-yl)phenyl)-1-phenyl-1H-phenanthro[9,10-d]imidazole (2-NaCPI) (Figure 26). The materials possess good film-forming capability and a high T_d_ (290–354 °C). Among them, ACPI displayed the highest CE_max_, at 1.6 cd/A at a low turn-on voltage of 3.0 V corresponding to CIE_xy_ (0.16, 0.17). A negligible efficiency roll-off is observed at high current densities [176].

In 2019, a material (4-(diphenylamino)phenyl)anthracen-9-yl)pyren-1-yl)-N,N-diphenylaniline (p-TPA-AP-TPA) (Figure 26) was synthesized with a dual-core moiety of anthracene and pyrene with a triphenylamine (TPA) side group attached at a para position to control the emission in the blue region. Moreover, the bulky molecule yielded a twisted molecular structure that prevents intermolecular interactions. The PL emission was around 473 nm in the film with a high T_g_ of 194 °C. The device showed a low turn-on of 3.1 V with an EQE of 8.4% and a maximum luminance of 7100 cd/m^2^ at CIE of (0.14, 0.15) [177].

#### 6.1.4. Fluorenes

Fluorenes were first reported in 2006 by Lai et al. for OLED applications. They designed oligofluorenes with a certain length to obtain a high quantum yield (Փ_PL_ = 38–75%) for a pure-blue emission. The PL emission was observed around 400 nm. An emissive layer of 130 nm was spin-casted and displayed a peak brightness of 1300 cd/m^2^ at a turn-on voltage of 5.3 V with a low current density of 90 mA/cm^2^. The CIE coordinates of (0.15, 0.09) displayed a deep-blue emission with an EQE of 2.0% and CE of 2.1 cd/A. These hindered six-arm architectures (highly branched and dendritic structures) suppress aggregation and self-quenching while the triazatruxene core facilitates charge injection, transport, and combination. Moreover, the powerful microwave-enhanced synthesis diminished impurities and chain defects that may result in quenching or keto-defects [178].

**Figure 27:**
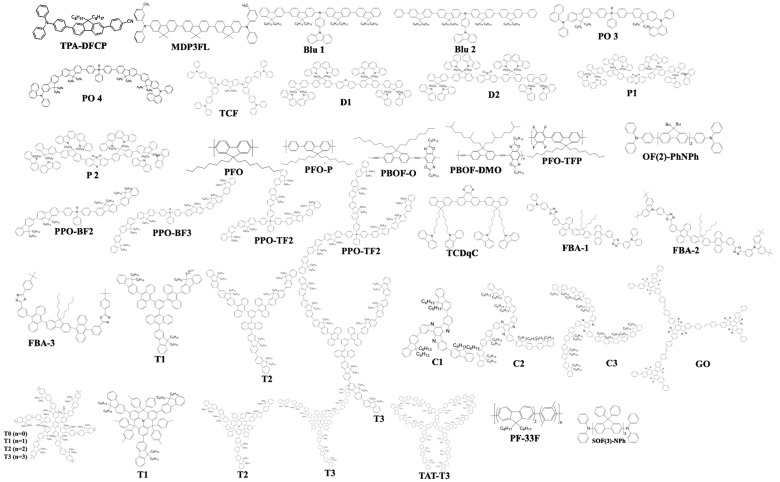
Molecular structures of fluorene-based blue emitters.

In 2007, Lai et al. reported a kinked star-shaped fluorene/triazatruxene co-oligomer (C1, C2 and C3) (Figure 27) that can effectively suppress aggregation and crystallization. The HOMO level was well matched to that of the anode’s work function that significantly improved the carrier transportation. The single-layer EL showed almost no change in color even at a higher voltage. The device displayed a low turn-on voltage of 3.3 V, EQE of 2.2%, and maximum luminance of 2400 cd/m^2^ [179].

In 2008, Jou et al. reported 2,7-bis{2[phenyl(*m*-tolyl)amino]-9,9-dimethyl-fluorene-7-yl}-9,9-dimethyl—fluorene (MDP3FL) (Figure 27) blue emitter doped in a low-polarity host, 4,4′-bis(9-carbazolyl)-biphenyl (CBP). The neat film of MDP3FL exhibits an EQE of 1.9% at 100 cd/m^2^ displaying a CIE of (0.15, 0.12). When doped with a 10 wt% in CBP, a 5.1% EQE was observed for a deep-blue emission at CIE (0.14, 0.08) [180].

In 2009, Wang et al. reported a solution-processable fluorescent π-conjugated dendrimer G0 (Figure 27). The material displayed a CE_max_ of 5.3 cd/A with a deep-blue emission at (0.15, 0.09). The EQE_max_ was 4.2% while 3.6% at 1000 cd/m^2^ [75].

Figure 27 shows the fluorene derivatives extensively utilized in efficient blue OLEDs, such as fluorene-bridged anthracene, [181] oligofluorenes, [74,77,182,183,184] p-difluorophenylene [185], bis(4-diphenylaminophenyl)carbazole end-capped fluorene [186], phosphine oxide [187,188], perfluorinated polymer [189], poly(3,6-Dimethoxy-9,9-dialkylsilafluorene)s [190], and polycyclic [191].

In 2020, Liu et al. reported a blue dye TPA-DFCP with a twisted D–π–A configuration where triphenylamine (TPA), benzonitrile (CP), and 9,9-dioctylfluorene served as D, A, and π-conjugation units, respectively. TPA is a strong electron donor group and has a high HOMO level. Moreover, a certain twist angle is present in the molecular space of TPA to avoid molecular accumulation. The non-doped device showed a high EQE of 7.7% with CIE coordinates of (0.15, 0.07) displaying a deep-blue emission. Meanwhile, the doped device showed an EQE_max_ of 8.3% and a radiative exciton utilization energy (η_r_) of 58% [192].

#### 6.1.5. Biaryl

**Figure 28:**
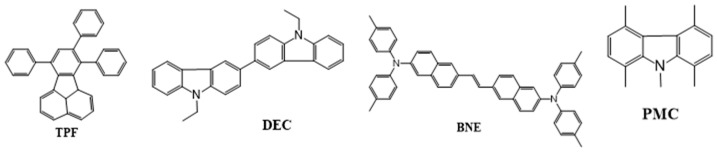
Molecular structures of biaryl-based blue emitters.

In 2005, Fisher et al. reported two materials: N,N′-diethyl-3,3′-bicarbazyl (DEC) and 1,4,5,8-N-pentamethylcarbazole (PMC) (Figure 28). Both the materials can be used as a hole-blocking layer and blue emitter. The device based on a DEC emitter doped in a DPVBi host displayed an EQE of 3.3%, CE of 4.7 cd/A, and PE of 1.3 lm/W at CIE coordinates of (0.15, 0.10) [193].

In 2006, Tseng demonstrated a device based on a 7,8,10-triphenylfluoranthene (TPF) emitter doped in a dipyrenylfluorene (DPF) host. With a 6 wt% of the TPF dopant, the device displayed a 3.3 lm/W PE and a 2.5% EQE at CIE coordinates of (0.16, 0.18) [194].

In 2007, Jou et al. reported three emitting materials, namely, trans-1,2-bis(6-(*N*,*N*-di-p-tolylamino)-naphthalene-2-yl)ethene (BNE), 2-(*N*,*N*-diphenylamino)-6-[4-(*N*,*N*-diphenylamino)styryl]naphthalene (DPASN), and di(triphenyl-amine)-1,4-divinylnaphthalene (DPVP) (Figure 28). Among them, BNE displayed the highest EQE, at 6.0%, and a PE of 12.5 lm/W at a low turn-on voltage of 3 V for a pan-blue emission at (0.19, 0.31) [195].

#### 6.1.6. Spiro-Shaped

**Figure 29:**
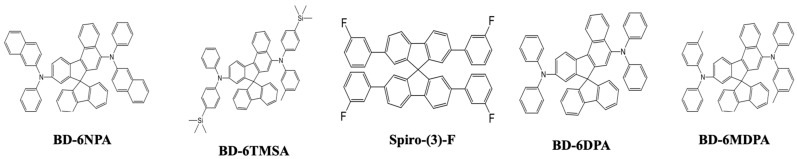
Molecular structures of spiro-based blue emitters.

In 2010, Jeon et al. reported a series of spiro-based emitters, namely, N,N,N′,N′-tetraphenylspiro[fluorene-7,9′-benzofluorene] (SFBF)-5,9-diamine (BD-6DPA), N,N′-di-(2-naphthyl)-N,N′-diphenyl-SFBF-5,9-diamine (BD-6NPA), N,N′-diphenyl-N,N′-di-m-tolyl-SFBF-5,9-diamine (BD-6MDPA), and N,N′-diphenyl-N,N′-bis(4-(trimethylsilyl)phenyl)-SFBF-5,9-diamine (BD-6TMSA) (Figure 29), synthesized using an animation reaction. Among them, BD-6MDPA displayed the highest CE, at 9.1 cd/A at 6.5 V, and an EQE of 8.2% with coordinates of (0.13, 0.15). The device showed a full width at half maxima of 48 nm and an emission peaking at 463 nm [82].

In 2013, Hou’s group designed and synthesized a series of fluorinated 9,9′-spirobifluorene derivatives (SFs). The photophysical properties, energy levels, and thermal properties can be tuned using different substitutional groups of electron-withdrawing moieties, such as F and CF_3_. Among the series, a 2,2′,7,7′-tetrakis(3-fluorophenyl)spiro-9,9′-bifluorene (Spiro-(3)-F) (Figure 29) blue emitter displayed the best result in a non-doped stage with an emission at CIE (0.16, 0.12) peaking at 408 nm. These SFs also served as a host for blue-emitting dopants, especially Spiro-(3)-F, with a low turn-on voltage of 3.4 V showing a CE of 6.7 cd/A and an EQE of 5.0% [196].

#### 6.1.7. Silanes

In 2001, Yu et al. reported for the first time a silane group as a blue emitter, tris(2,3-methyl-8-hydroxyquinoline) aluminum (III) (Alm_23_q_3_). The presence of two electron-donating methyl groups widened the HOMO/LUMO gap and blue-shifted the EL peaks to 470 nm. The resultant device showed a power efficacy of 0.6 lm/W at 300 cd/m^2^ [197].

**Figure 30:**
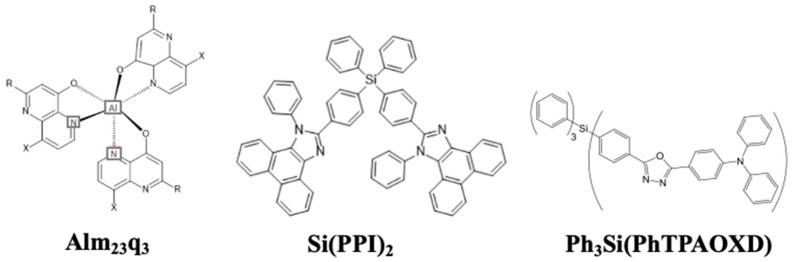
Molecular structures of silane-based blue emitters.

In 2002, Chan et al. reported a molecular glass material (4-(5-(4-(diphenylamino)phenyl)-2-oxadiazolyl)phenyl)triphenylsilane (Ph_3_Si(PhTPAOXD)) (Figure 30) as a blue emitter. The material possessed a T_g_ of 85 °C, making it less vulnerable to heat and hence more stable. The device showed a maximum luminance of 19,000 cd/m^2^ with an EQE of 2.4% at CIE coordinates of (0.16, 0.18) [198].

In 2015, Liu et al. reported an organosilane compound, bis(4-(1-phenylphenanthro[9,10-d]imidazol-2-yl)phenyl)diphenylsilane (Si(PPI)_2_) (Figure 30), as a bipolar host. The incorporation of one tetraphenylsilane and two PPI groups made the compound thermally stable with a high T_d_ of 528 °C and a T_g_ of 178 °C. Moreover, when doped with a 10 wt% An(PPI)_2_ emitter, the device showed a PE of 8.0 lm/W and an EQE of 6.1% at CIE_xy_ (0.18, 0.17) [199].

#### 6.1.8. Carbazole

**Figure 31:**
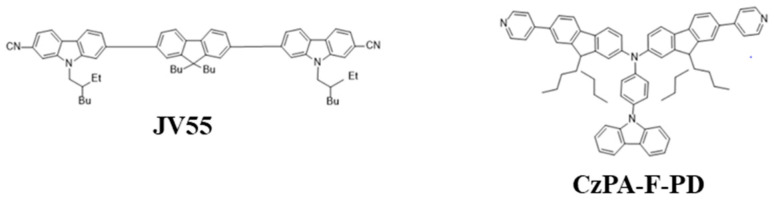
Molecular structures of carbazole-based blue emitters.

In 2018, Jou et al. reported a carbazole-based deep-blue fluorescent emitter, 6-((9,9-dibutyl-7-((7-cyano-9-(2-ethylhexyl)-9H-carbazol-2-yl)ethynyl)-9H-fluoren-2-yl)ethynyl)-9-(2-ethylhexyl)-9H-carbazole-2-carbonitrile (JV55) (Figure 31), that showed a maximum EQE of 6.5% at CIE coordinates of (0.16, 0.06). The deep-blue emission realized a 101% color saturation according to the NTSC standard. The very high PLQY of 91%, effective host-guest energy transfer, efficient triplet energy utilization, and low doping concentration realized a low roll-off [200].

In 2020, Yang et al. reported a blue fluorescent material with a twisted A–π–D–π–A configuration, namely, CzPA-F-PD (Figure 31). This material exhibited dual fluorescent properties that emitted blue and red emissions under PL and EL processes when triggered by trifluoroacetic acid (TFA). The property had been reversed to its initial state on neutralizing TFA with triethylamine (TEA). It was observed that hydrogen protonation of TFA amplified the lone pair electrons around the nitrogen atoms in pyridine, enhancing the electron acceptor ability. This scheme also played a significant role during the dual fluorescent process. The material possessed a good exciton utilization efficiency of 67%, exceeding the upper limit of spin-statics for fluorescent materials [201].

#### 6.1.9. Oxidiazole

**Figure 32:**
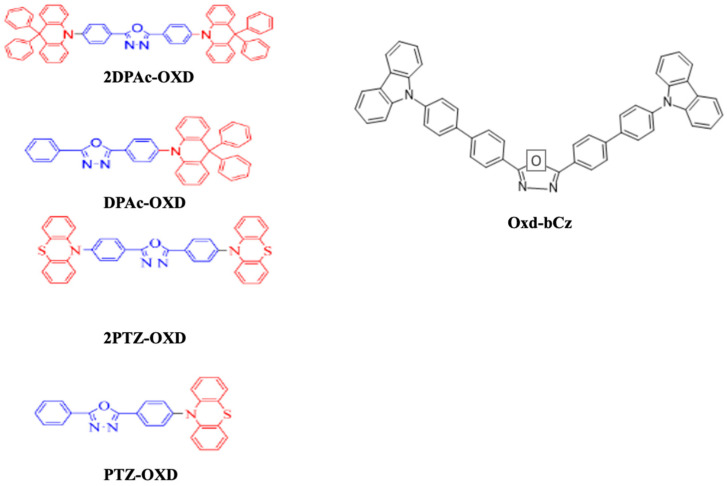
Molecular structures of oxadiazole-based blue emitters.

In 2018, Wu et al. reported that triazine, oxadiazole, triazole, cyno-substituted benzene, and benzothiadiazole worked as acceptor units while carbazole, arylamine, phenothiazine, and their derivatives were utilized as donor moieties for a bipolar structure. Consequently, they utilized phenothiazine and 9,9-diphenyl-9,10-dihydroacridine as a donor unit and oxadiazole derivatives, 2,5-bis(4-bromophenyl)-1,3,4-oxadiazole and 2-(4-bromophenyl)-5-phenyl-1,3,4-oxadiazole, as acceptor units to synthesize a series of bipolar emitters, namely, 2DPAc-OXD, DPAc-OXD, 2PTZ-OXD, and PTZ-OXD (Figure 32). All the materials possessed a high decomposition temperature above 358 °C, with emissions in the PL range of 435–512 nm. For OLED applications, these materials showed a moderate turn-on voltage of around 4.2 V along with a highest EQE of 4.0% when using the material 2PTZ-OXD [202].

Later that year, Wang et al. reported a bipolar molecule, bis(4’-(9H-carbazol-9-yl)-[1,1-biphenyl]-4-yl)-1, 3, 4-oxadiazole (Oxd-bCz) (Figure 32), by incorporating electron-donor and electron-withdrawal moieties. The material emitted a purple-blue fluorescence at 431 nm when diluted in dichloromethane. From a device perspective, the device turned on at 3.6 V and displayed a maximum EQE of 5.6% at CIE_y_ ~ 0.07 [203].

### 6.2. Phosphorescent Type

Developing phosphorescent emitters is essential as they have the potential to realize a high-efficiency OLED. In 1998, Forrest and Thompson et al. discovered a phosphorescent emitter to develop a nearly 100% internal quantum efficiency because of their ability to efficiently harvest both singlet and triplet excitons [204,205,206,207,208]. Since then, very high-efficiency phosphorescent green and red emitters have emerged. However, it was a great challenge to synthesize a highly energy-efficient phosphorescent blue emitter that exhibits a long lifetime with high color purity. Deep blue emitters should have a high triplet energy (~3 eV), a high photoluminance quantum yield (PLQY), and a short excited-state lifetime. To realize the above-mentioned features, considerable efforts have been made to develop a high performance deep-blue emitter.

On the basis of previous investigations, it is well established that complexes of heavy metals such as iridium (III), platinum (II), and ruthenium (II) have been utilized to synthesize promising blue emitters [209,210,211]. Among them, iridium complex-based phosphors have shown enormous potential to realize high-efficiency blue OLEDs. Moreover, to achieve a deep-blue emission, thermally and electrochemically stable fluorine-free cyclometalating ligands [210,212,213] were introduced either by using electron-withdrawing groups [214,215] or electron-deficient heterocycles [215,216]. Furthermore, to stabilize HOMO, ancillary ligands with strong ligand field strength, such as isocyanide or phosphine, were utilized [215,217].

#### 6.2.1. Iridium (III) Complexes

**Figure 33:**
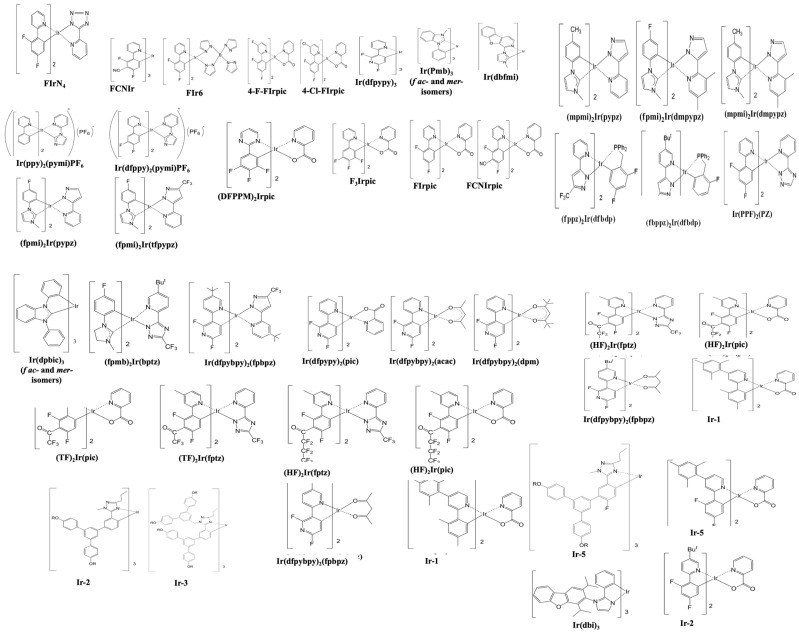
Molecular structures of Ir-complex-based blue emitters.

In 2001, Adachi et al. reported a sky-blue phosphorescent emitter, FIrpic (Figure 33), with an EQE of 5.7% and CIE coordinates of (0.16, 0.29) [60]. In 2003, Holmes et al. synthesized a blue emitter, iridium(III) bis(2,4-difluorophenylpyridinato)tetrakis(1-pyrazolyl)borate (FIr6) (Figure 33), with an EQE of 12% at (0.16, 0.26) [218]. In the same year, Bazan’s group reported a series of anthracene-containing binaphthol emitters with CIE coordinates of (0.18, 0.21) without revealing device performance [209].

In 2005, Holmes reported fac- and mer-isomers of fluorine-free emitters tris(phenyl-methyl-benzimidazole)iridium(III) (f-Ir(pmb)_3_) and (m-Ir(pmb)_3_), respectively (Figure 33). When f-Ir(pmb)_3_ was doped into a p-bis(triphenylsilyly)benzene (UGH2) host, the device showed an EQE of 5.8% for a deep-blue emission with CIE coordinates of (0.17, 0.06) [210].

Moreover, other fluorine-free Ir complexes which resulted in a blue emission were also reported. They were mer-tris(Ndibenzofuranyl-N-methylimidazole) iridium(III) [Ir-(dbfmi)] (Figure 33) [212] and tris(1-cyanophenyl-3-methylimidazolin-2-ylidene-C,C2′) iridium(III) [Ir(cnpmic)] [213].

In 2009, Kang et al. introduced a blue emitter based on an iridium(III) complex of 2′,6′-difluoro-2,3′-bipyridine (fac-Ir(dfpypy)_3_) (Figure 33) with CIE coordinates of (0.14, 0.12) [219]. Meanwhile, the same group reported a comparatively more efficient device with an EQE of 10% at 1000 cd/m^2^ at (0.15, 0.24) by employing its modified complex, (dfpypy)_2_Ir(μ-Cl)_2_) [220].

In 2010, Kido et al. reported a halogen-free blue emitter, mer-tris(N-dibenzofuranyl-N′-methylimidazole)iridium(III) (Ir(dbfmi)) (Figure 33). By doping a 10 wt% Ir(dbfmi) into a 3,6-bis(diphenylphosphoryl)-9-phenylcarbazole (PO9) host, the resultant device showed a maximum EQE of 19% with CIE_xy_ (0.15, 0.19) [212]. In 2011, Hsieh et al. reported blue emitters of iridium bis(carbene) complexes, namely, Ir(mpmi)_2_(pypz), Ir(fpmi)_2_-(pypz), and Ir(fpmi)_2_(tfpypz) (Figure 33), that showed an EQE of 15, 14, and 7.6% and CIE_xy_ of (0.14, 0.27), (0.14, 0.18), and (0.14, 0.10), respectively [221].

In 2012, Fan et al. reported two phosphoryl/sulfonyl-substituted iridium complexes, POFIrpic and SOFIrpic (Figure 33), by introducing phosphoyl/sulfonyl moieties into the 5′-position of a phenyl ring in the structural frame of FIrpic. By doping a POFIrpic emitter into PVK:OXD-7 hosts, the solution-processable blue device showed CIE coordinates of (0.16, 0.29) and an EQE of 3.5% at 1000 cd/m^2^ [222]. In 2013, Fan et al. modified the FIrpic by introducing fluorine, chlorine, and bromine atoms to the 4-position of a pyridine ring. The 4-F-FIrpic-based device showed an EQE of 15% for a blue emission with CIE_xy_ (0.15, 0.28) [223]. Meanwhile, Kessler et al. synthesized a heteroleptic iridium complex based on a 4-(tert-butyl)-2′,6′-difluoro-2,3′-bipyridine ligand (FK306) (Figure 33) that showed an EQE of 13% at 1000 cd/m^2^ for a greenish-blue emission at (0.16, 0.25) [224]. In the same year, Yoon et al. reported a series of Ir(III) complexes using (4′-substituted-2′-pyridyl)-1,2,4-triazole ancillary ligands. By doping a 10 wt% of iridium complex of 5-(4′-methylpyridine-2′-yl)-3-trifluoromethyl-1,2,4-triazolate ancillary ligand (Ir5) (Figure 33) into a 4,4′-bis(9-carbazolyl)-2,2′-dimethylbiphenyl (CDBP) host, the resultant device showed a blue emission with CIE coordinates of (0.15, 0.18) [225].

Moreover, Lee et al. reported a series of near deep-blue iridium(III) complexes, (TF)_2_Ir(pic), (TF)_2_Ir(fptz), (HF)_2_Ir(pic), and (HF)_2_Ir(fptz) (Figure 33). They consisted of 2′,4″-difluororphenyl-3-methylpyridine with a trifluoromethyl carbonyl or heptafluoropropyl carbonyl group as the main ligand and a picolinate or a trifluoromethylated triazole as the ancillary ligand. Among them, (TF)_2_Ir(fptz) achieved an EQE of 7.4% at 100 cd/m^2^ for a blue emission with CIE coordinates of (0.14, 0.11) [226].

In 2014, Liu et al. reported a host molecule, 3,6-bis(diphenylphosphoryl)-9-(4′-(diphenylphosphoryl) phenyl)-carbazole (TPCz) (Figure 33). The solvent effect was studied by dissolving a mixture of TPCz and FIrpic (emitter) in various polar solvents. They thought that the higher polar halogen-free solvent isopropanol provided good solubility, helping resist the aggregation of FIrpic and hence render a more densely packed emissive layer. The efficiencies of 22 cd/A and 16 lm/W at (0.15, 0.32) were also reported [227].

In 2015, Sun et al. reported a series of 3,3′-bicarbazole (mCP)-functionalized tetraphenylsilane derivatives (SimCPx) (Figure 33). On changing the number of mCP subunits on the central Si atom, four molecules, namely, bis(3,5-di(9H-carbazol-9-yl)phenyl)diphenylsilane (SimCP2), tris(3,5-di(9H-carbazol-9-yl)phenyl)methylsilane (SimCP3-CH3), tris(3,5-di(9H-carbazol-9-yl)phenyl)phenylsilane (SimCP3-Ph), and tetrakis(3,5-di(9H-carbazol-9-yl)phenyl)silane (SimCP4), were synthesized as bipolar hosts. The derivatives possessed a wide bandgap and good solubility. Moreover, a high thermal and morphological stability was beneficial for the formation of amorphous and homogeneous film via wet processing. Among them, SimCP4 showed the best electron-transporting ability and hence, at a low driving voltage of 4.8 eV, achieved an EQE_max_ of 14%, CE_max_ of 28 cd/A, and PE_max_ of 14 lm/W corresponding to a CIE_xy_ (0.15, 0.35) by using a FIrpic emitter [228].

In 2016, Byeong et al. reported meta-linked diphenylether-based materials, 9-(3-(3-(9H-carbazol-9-yl)phenoxy)phenyl)-9H-pyrido[2,3-b]indole (CzDPEPI) and 9,90-(oxybis(3,1-phenylene))bis(9H-carbazole) (CzDPECz) (Figure 33). The presence of a pyridoindole moiety assisted in getting a better electron injection and hence a lower driving voltage. The CzDPEPI host possessed a high triplet energy and therefore was suitable for an Ir complex (Ir(dbi)_3_) emitter. The resultant device showed an EQE_max_ of 24% and PE_max_ of 39 lm/W at CIE_xy_ (0.19, 0.37) [229].

In 2017, Park et al. reported a series of bipolar host materials, namely, N-(3-(9H-carbazol-9-yl)phenyl)-N-(pyridin-2-yl)pyridin-2-amine (mCPPy), 5-(9H-carbazol-9-yl)-N1,N1,N3,N3-tetra(pyridin2-yl)benzene-1,3-diamine (CPDPy), and N-(3,5-di(9H-carbazol-9-yl)phenyl)-N-(pyridin2-yl)pyridin-2-amine (DCPPy) (Figure 33). The materials possessed a high bandgap (>3.5 eV) and a high triplet energy (>2.9 eV). The device composing CPDPy doped with FIrpic (emitter) showed a highest EQE_max_ of 22% and PE_max_ of 24 lm/W at CIE coordinates of (0.16, 0.31) [230].

In 2018, Kim et al. reported an emitter material, *fac*-tris(2,3-dimethylimidazo[1,2-f]phenanthridinato-k2C,N)iridium(III) (Figure 33), that had a facial (*fac*) geometry around the iridium atom. A multilayered device composed of 1,3-bis(N-carbazolyl)benzene as a host/hole-transporting layer with a 5 wt% emitter displayed a high EQE_max_ of 20% and PE_max_ of 32 lm/W with coordinates of (0.18, 0.34) [89].

In 2020, Yuan et al. reported a host material, 9,9-bis(9-phenyl-9H-carbazol-3-yl)-9H-thioxanthene,10,10-dioxide (BPhCz-ThX) (Figure 33), based on an insulating C(sp^3^) bridge-linked thioxanthone via a facile Friedel–Crafts-type substitution reaction. The material possessed a high thermal stability, good film-forming ability, a high triplet energy (3.05 eV), and a total yield of 63%. On using a FIrpic emitter, the device showed an EQE_max_ of 9.6%, CE_max_ of 21 cd/A, and PE_max_ of 8.5 lm/W at (0.19, 0.36) [231].

#### 6.2.2. Platinum Complexes

Platinum (Pt) complexes are the second most appreciated organic dopants to realize a highly efficient deep-blue emitter. Pt-based phosphors appear to have the highest potential for EL applications due to their higher triplet quantum yield, relatively short triplet state lifetime, and tunable emission color [211,213,232]. Based on their structural features, these phosphors are classified into three main categories: (i) tetradentate ligands, (ii) tridentate ligands, and (iii) bidentate ligands.

**Figure 34:**
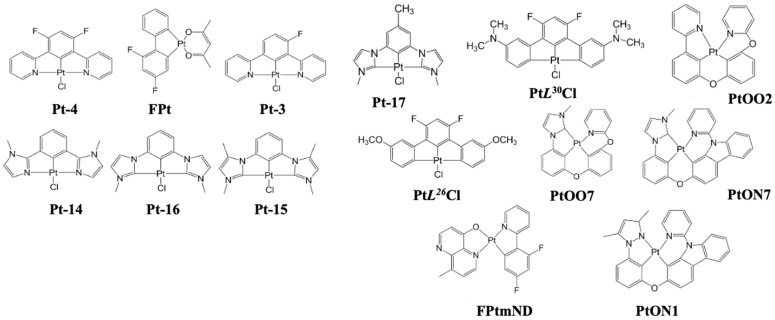
Molecular structure of platinum complex-based blue emitters.

We have described a few platinum complexes based on the above three categories. In 2008, Yang et al. reported a blue device by utilizing a platinum(II) 1,3-difluoro-4,6-di(2-pyridinyl)benzene chloride (Pt-4) (Figure 34) emitter, and the resultant device exhibited CIE coordinates of (0.15, 0.26) with a maximum EQE of 16% [211].

In 2013, Turner et al. synthesized emitters based on cyclometalated tetradentate platinum complexes, a blue PtOO1, and a blue-green PtOO2 emitter (Figure 34). The device using PtOO2 exhibited an EQE of 8.7% with CIE coordinates of (0.21, 0.38) at 1000 cd/m^2^ [232]. In the same year, Hang et al. reported Pt complexes with tetradentate ligands. These complexes have a conventional cyclometalated fragment bridged with oxygen to an ancillary chelate, such as phenoxyl pyridine (POPy) or carbazolyl pyridine (CbPy). Doping 6 wt% emitters, Pt[pmi-O-POPy] (PtOO7), Pt[pmi-O-CbPy] (PtON7) and Pt[ppz-O-CbPy] (PtON1), into a 2,6-bis(N-carbazolyl)pyridine (26mCPy) host resulted in CIE coordinates of (0.15, 0.10), (0.15, 0.14), and (0.15, 0.13) and an EQE of 0.5, 15, and 21%, respectively, at 1000 cd/m^2^ [213].

In 2015, Liao et al. reported a series of Pt(II) metal complexes [Pt(Ln)], n = 1–5 based on tetradentate bis(pyridyl azolate) chelates. Among them, L5 (Figure 34) showed a high EQE_max_ of 15%, CE_max_ of 36 cd/A, and PE_max_ of 38 lm/W at CIE coordinates of (0.19, 0.34) [233].

In 2019, Klimes et al. reported a step-wise graded doping of platinum(II) 9-(pyridin-2-yl)-2-(9-(pyridin-2-yl)-9H-carbazol-2-yloxy)-9H-carbazole (PtNON) (Figure 34). The emissive layer was composed of a 20% PtNON:mCBP (10 nm)/10% PtNON:mCBP (4 nm)/2% TBPe:mCBP (2 nm)/10% PtNON:mCBP (4 nm)/6% PtNON:mCBP (10 nm). Consequently, the device showed an EQE_max_ of 18% and a lifetime of 709 h [LT_70_] at 1000 cd/m^2^ with CIE_xy_ (0.16, 0.28) [102].

#### 6.2.3. Other Metal Complexes

Due to limited availability and high cost, some research efforts have been made to substitute heavy metal complexes with comparatively low-cost metals such as zinc (Zn), copper (Cu), etc. In 2004, Wang et al. synthesized a Zn(II) metal complex-based phosphorescent blue emitter using 4,4′-diphenyl-6,6′-dimethyl-2,2′-bipyrimidine ligands, [(Zn(pmbp)_2_(ClO_4_)_2_ and Zn(pmbp)Cl_2_]. However, no efficiencies or color coordinates were reported [234].

**Figure 35:**
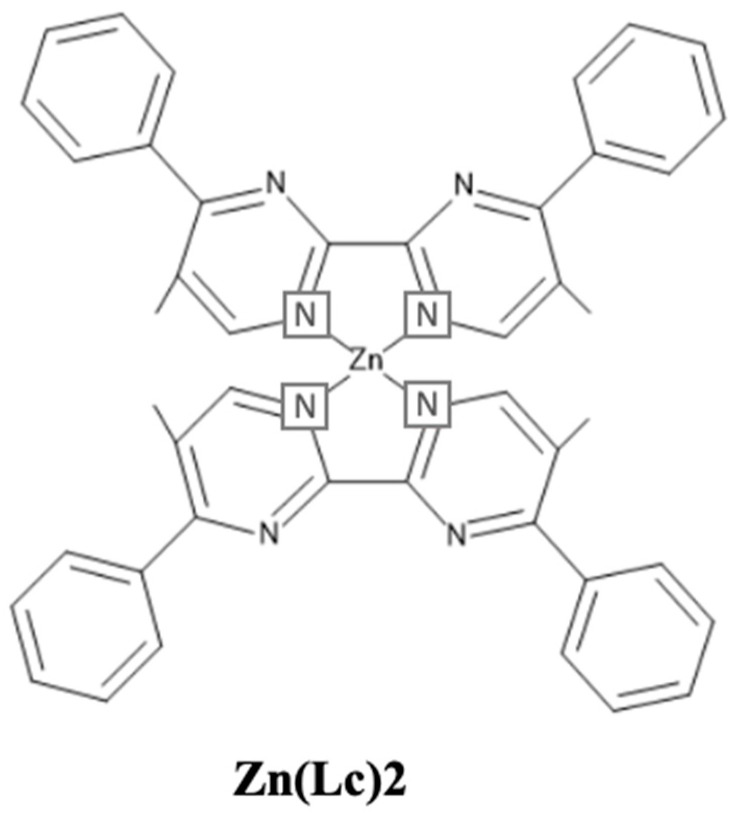
Molecular structure of Zn(Lc)2 blue emitter.

In 2008, Xu et al. reported a deep-blue emitting Zn(II) complex, Zn(Lc)2 (Lc− = 2-(1-(6-(9H-carbazol-9-yl)hexyl)-1H-benzo[d]imidazol-2-yl)phenolate) (Figure 35), based on a carbazole-functionalized N^O ligand. The material possessed a T_d_ of 427 °C and an emission peak at 422 nm [235]. In 2013, Lee et al. synthesized two Zn complexes, bis(2-(oxazol-2-yl)phenolate)zinc (ZnOx2) and bis(2-(1-methyl-1h-imidazole-2-yl)phenolate)zinc (ZnIm2) host materials. The blue device based on a 3 wt% FIrpic (emitter) doped in ZnIm2 reached an EQE of 20% at 100 cd/m^2^ and 18% at 1000 nits [236].

Suitable host materials were presented to aid the emission of phosphorescent-based blue emitters as reported by Chaskar et al [1] and Yook et al [237].

### 6.3. Thermally Activated Delayed Fluorescence (TADF) Type

High price and rare availability of heavy metals have limited the low-cost and long-term mass production of phosphorescent blue emitters. To resolve these problems, the search for a highly efficient deep-blue fluorescent emitter is still a subject of current interest, as they have a greatest possibility of a longer operational lifetime and a deep-blue emission at low cost with highly sustainable purely organic luminescent molecules [238].

In 2009, Adachi et al. designed and synthesized the first heavy metal-free TADF emitter [239]. Since then, numerous efforts have been made to realize efficient TADF-based blue emitters, especially with a smaller singlet-triplet energy difference (${\Delta E}_{ST}$) [239,240,241,242,243,244,245]. Presently, aromatic- and metal (such as Li, Al, Mg, Cu, and Zn)-based blue emitters have been found useful in achieving a small ${\Delta E}_{ST}$.

#### 6.3.1. Aromatic-based

Over the past few years, researchers have extensively designed and synthesized aromatic-based efficient TADF emitters by using abundant elements, such as carbon, hydrogen, oxygen, nitrogen, sulphur, etc. So far, a few molecular design strategies have been reported to achieve smaller ${\Delta E}_{ST}$ (<0.1 eV) values with a high PLQY (~100%). Most importantly, the delocalization of frontier molecular orbitals (highest occupied molecular orbital (HOMO) and lowest unoccupied molecular orbital (LUMO) energy levels) in a donor–acceptor (D–A) type TADF emitter with a well-separated HOMO and LUMO levels can effectively realize a smaller ${\Delta E}_{ST}$ and a nearly 100% PLQY [246]. Moreover, increasing a steric hindrance between D–A units can also lead to the spatial separation of the frontier molecular orbitals.

In aromatic-based TADF emitters, the spatial overlap between the frontier molecular orbitals can be successfully controlled by choosing a suitable D–A pair. Moreover, it has been found that the donor–acceptor–donor (D–A–D) type emitters are more effective than those of the D–A type in achieving a higher PLQY and a strong spatial separation of HOMO LUMO levels [65,247]. Since 2009, several D–A- and D–A–D-type bipolar emitters have been reported. Among them, a few emitters demonstrated a nearly 100% internal quantum efficiency (IQE) and a 20% EQE in blue devices [246,248,249].

**Figure 36:**
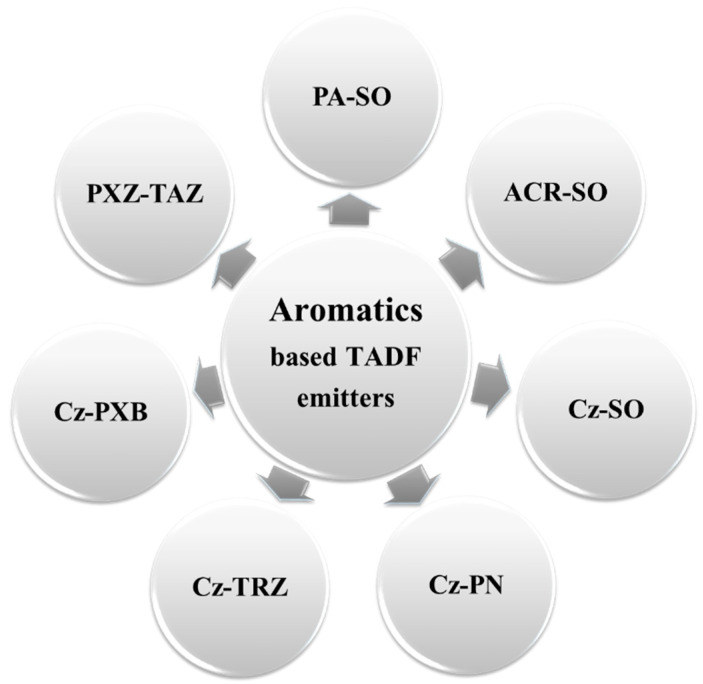
Types of aromatic TADF blue emitters based on different donor–acceptor pairs. All abbreviations are given as follows: PA (phenylamine), DPS (diphenylsulfone), ACR (acridine), Cz (carbazole), PN (phthalonitrile), PXB (phenoxaborin), PXZ (phenoxazines), TAZ (triazole), and TRZ (triazine).

**Figure 37:**
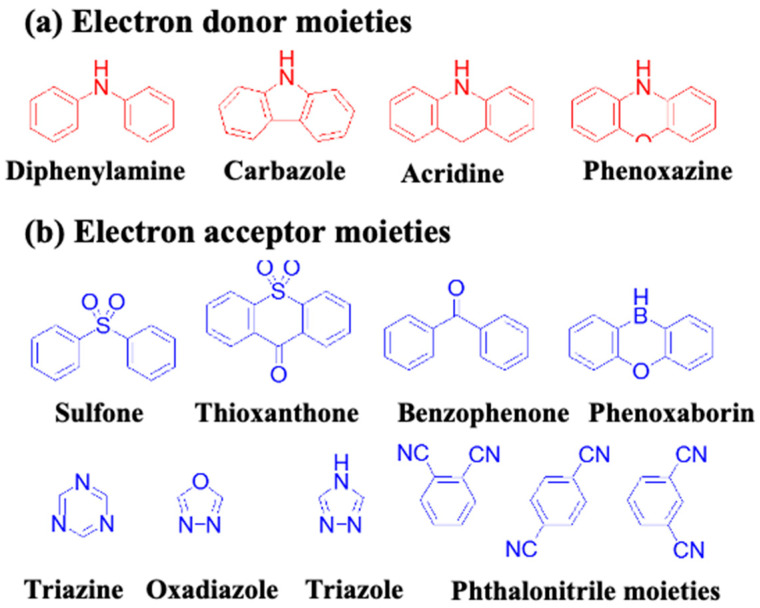
Molecular structures of (**a**) electron donor moities and (**b**) electron acceptor moities, frequently used in aromatic-based blue TADF emitters.

On the basis of D–A pairs, aromatic-based TADF blue emitters can be classified into seven sub-categories (Figure 36), which comprise diphenylamine, acridine, carbazole, and phenoxazines as electron-donor units and diphenylsulfone, thioxanthone, benzophenone, triazine, oxadiazole, triazole, and phthalonitrile as electron-acceptor units (Figure 37).

##### Diphenylamine and Diphenylsulfone (DPA-DPS) Derivatives

**Figure 38:**
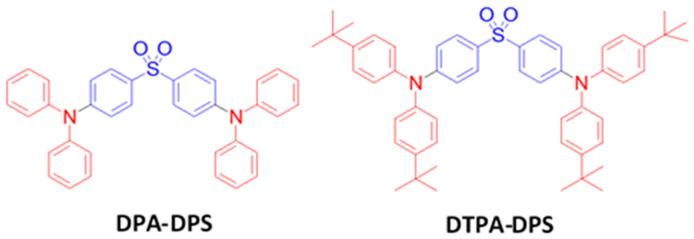
Molecular structures of DPA-DPS-based blue emitters.

Diphenylamine moiety-containing aromatic compounds are well-established hole-transporting materials in OLED devices. In contrast, diphenylsulfone moiety-comprising materials are well studied as electron transporting materials. These moieties have also been used to design bipolar hosts [250,251,252,253]. In accordance with the molecular design strategy, in 2012, Adachi et al. synthesized a series of bipolar TADF emitters, bis[4-(diphenylamino)phenyl] sulfone (DPA-DPS) and bis{4-[bis(4-tert-butylphenyl)amine]phenyl} sulfone (DTPA-DPS) (Figure 38), using diphenylamine donor and diphenylsulfone acceptor moieties [254]. The DPA-DPS and DTPA-DPS emitters possessed a ΔE_ST_ of 0.54 and 0.45 eV and a PLQY of 57 and 65%, respectively. In a doped device with a bis[2-(diphenylphosphino)phenyl]ether oxide (DPEPO) host, the DPA-DPS-based device exhibited an EQE_max_ of 2.9%, while the DTPA-DPS-based device showed an almost double EQE_max_ of 5.9%. The higher EQE of the DTPA-DPS emitter is attributed to a low ΔE_ST_, which possessed an effective triplet to singlet-exciton upconversion.

##### Acridine and Diphenylsulfone (ACR-DPS) Derivatives

**Figure 39:**
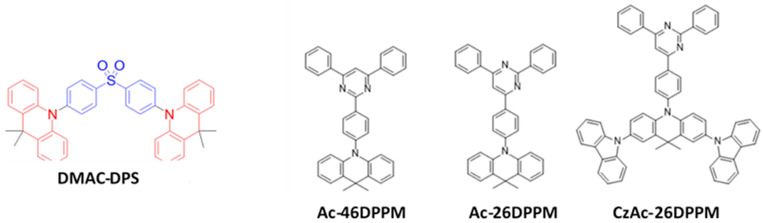
Molecular structures of ACR-DPS-based blue emitters.

Acridine derivatives were utilized as hole-transporting and bipolar host materials in OLEDs [255,256,257,258,259,260,261,262]. Combinations of acridine-based electron-donor and diphenylsulfone-based electron-acceptor units have been widely studied for TADF type emitters. In 2014, Adachi’s group designed and synthesized an acridine donor and diphenylsulfone acceptor moiety-based 9,9-(dimethyl-9,10-dihydroacridin)diphenyl sulfone (DMAC-DPS) (Figure 39) emitter. The emitter possessed a ΔE_ST_ of 0.08 eV with an 80% PLQY and a 1.7 to 3 μs TADF decay time. By doping into a DPEPO host, the DMAC-DPS-based device exhibited an EQE_max_ of 20% for a greenish-blue emission at (0.16, 0.20). The resultant device performance was almost independent of the emitter doping concentration [248]. Moreover, the same group reported an EQE_max_ of 20% for a sky-blue emission with (0.16, 0.29) by using a non-doped DMAC-DPS emitter [249].

In 2014, Nakanotani et al. achieved an EQE_max_ of 13% for a bluish-green emission with coordinates (0.17, 0.30) by using a 1 wt% fluorescent emitter, 2,5,8,11-tetra-tert-butylperylene, along with a 15 wt% of TADF emitter, 10-phenyl-10H, 10′H-spiro[acridine-9, 9′-anthracen]-10′-one (ACRSA), in a bis-(2-(diphenylphosphino)phenyl)ether oxide (DPEPO) host [263].

In 2017, Nakao reported three emitters, namely, Ac-46DPPM, Ac-26DPPM, and CzAc-26DPPM (Figure 39), with 2,4,6-triphenylpyrimidine as an acceptor and tri- or penta-substituted acridine as a donor unit. Among them, CzAc-26DPPM displayed an EQE of 15% and CE of 35 cd/A at 100 cd/m^2^ for a bluish-green emission with coordinates (0.21, 0.38) [264].

##### Carbazole and Diphenylsulfone (Cz-DPS) Derivatives

Carbazole-based polycylic aromatic hydrocarbon compounds are extensively studied in OLED devices due to their numerous superlative characteristics, such as a strong hole-transporting ability, high thermal stability, large optical band gap, high triplet energy, rational PLQY, and easy functionalization [265,266]. On the other hand, diphenyl sulfone derivatives are robust electron-transporting materials for OLED devices [254].

**Figure 40:**
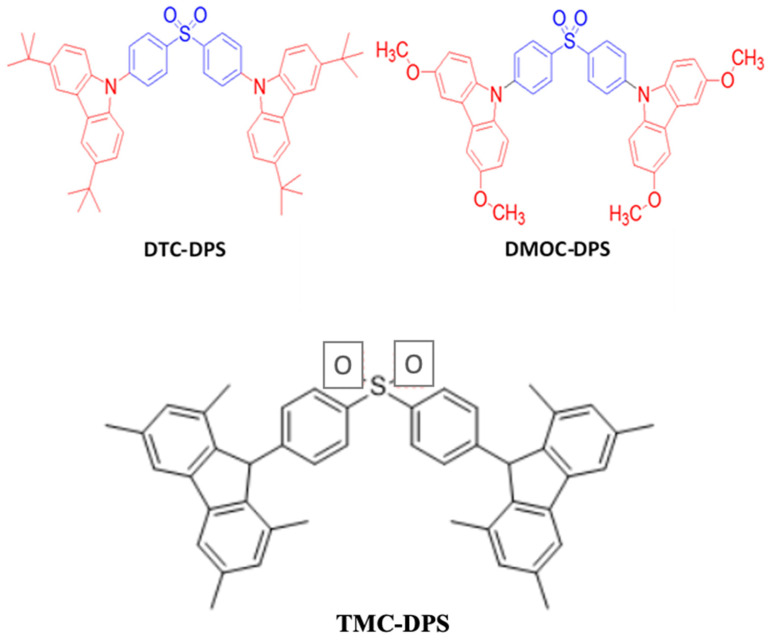
Molecular structures of Cz-DPS-based blue emitters.

In 2012, Zhang et al. reported an EQE_max_ of 10% for a deep-blue emission with CIE coordinates of (0.15, 0.07) by doping a TADF blue emitter, bis[4-(3,6-di-tert-butylcarbazole)phenyl] sulfone (DTC-DPS) (Figure 40), into a DPEPO host. The resultant device realized an EQE over three times higher than that of a diphenylamine-based DPA-DPS and nearly two times higher than that of a tert-butyl diphenylamine-derivative DTPA-DPS. The DTC-DPS exhibiting a high EQE may be attributed to a relatively small ΔE_ST_ value of 0.32 eV and a high PLQY of 69% because of a considerably strong electron donating characteristic of carbazole units [254].

In order to further decrease the ΔE_ST_ value, in 2014, Wu et al. designed a blue emitter, bis [4-(3,6-dimethoxycarbazole)phenyl]sulfone (DMOC-DPS) (Figure 40), by using a methoxy substituted carbazole with an electron-acceptor sulfone. The emitter realized a ΔE_ST_ of 0.21 eV with a 93 μs TADF decay time and a 56% PLQY. In a doped blue device with a DPEPO host, the DMOC-DPS exhibited an EQE_max_ of 15% with (0.16, 0.16) coordinates [267]. The reason why the DMOC-DPS resulted in an EQE much higher than that of the DTC-DPS may be attributed to a comparatively smaller ΔE_ST_.

Furthermore, in 2016, Lee et al. reported a TADF emitter, TMC-DPS (Figure 40), with diphenyl sulfone (DPS) as an electron acceptor and 3,6-dimethoxycarbazole (DMOC) and 1,3,6,8-Tetramethyl-9H-carbazole (TMC) as electron donor units. The material possessed a low ΔE_ST_ of 0.09 eV, much lower than that of DMOC-DPS, due to a large dihedral angle that creates a small spatial overlap between the frontier molecular orbitals [268].

##### Carbazole and Triazine (Cz-TRZ) Derivatives

Nitrogen-containing heterocyclic compounds, such as triazine and 1,3,4-triazole, have been widely used as electron-accepting units in the molecular scaffold of electron-transporting materials because of their electron-deficient nature. To design a bipolar TADF emitter, strong hole-transporting carbazole moieties have been added onto transporting triazine moieties. Both moieties are extensively utilized to design an efficient bipolar host and blue light-emitting materials.

**Figure 41:**
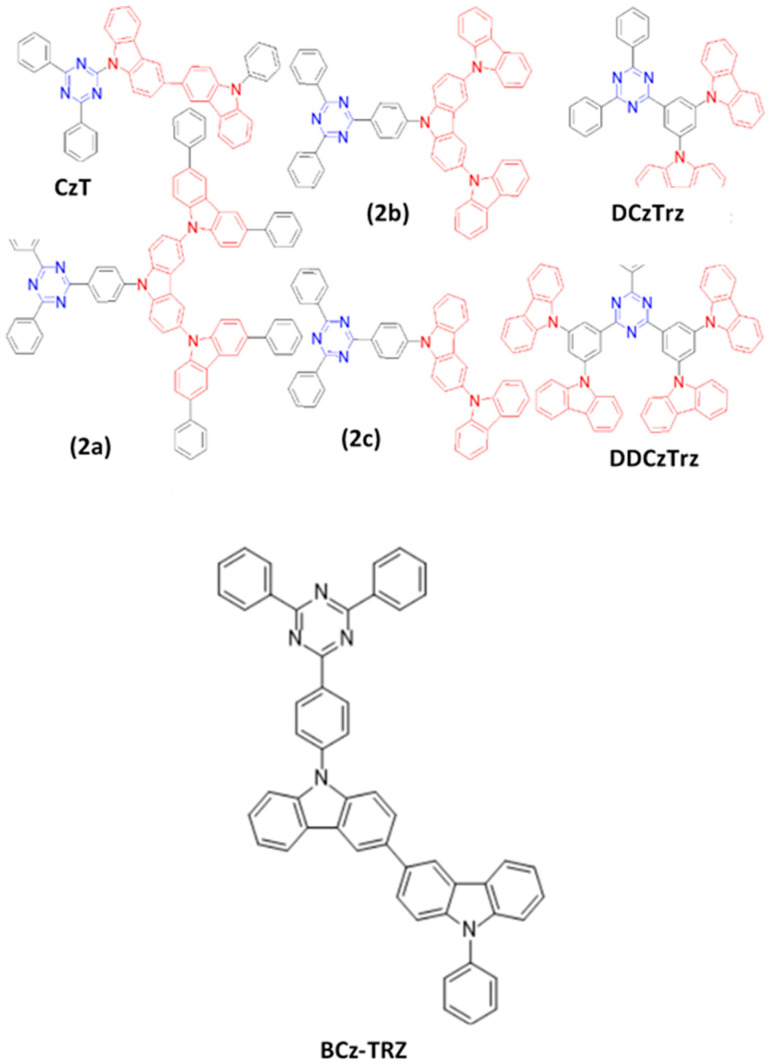
Molecular structures of Cz-TRZ-based blue emitters.

In 2013, Serevičius et al. reported a 6% EQE_max_ for a greenish-blue OLED with (0.23, 0.40) by using a 9-(4,6-diphenyl-1,3,5-triazin-2-yl)-9′-phenyl-3,3′-bicarbazole (CzT) (Figure 41) emitter [269]. In 2014, Hirata and co-workers reported a series of bipolar blue light-emitting materials (2a, 2b, and 2c) (Figure 41) by employing indolocarbazole and triazine moieties. The resultant device employing a 2a emitter doped in a DPEPO host realized a highest EQE of 21% for a sky-blue device with (0.19, 0.35) [246].

In 2015, Lee et al. reported a pair of bipolar TADF type blue emitters (DCzTRz and DDCzTRz) (Figure 41) with dicarbazolylbenzene donor and triazine acceptor moieties [270]. Both DCzTRz and DDCzTRz exhibited a ΔE_ST_ of 0.25 and 0.27 eV and a solid state PLQY of 43 and 66%, respectively. As both emitters were doped into a DPEPO host, the DCzTRz emitter-based device exhibited a maximum EQE of 18% with (0.15, 0.15), while a 19% EQE with (0.16, 0.22) was reported for the DDCzTRz-based device. Furthermore, owing to high thermal and electrical stability, the DDCzTRz-based device also demonstrated an operational lifetime (T_80_ = 52 h at an initial luminance of 500 cd/m^2^).

In 2020, Su et al. reported a 3,3′-bicarbazole/triphenyltriazine-derivative (BCz-TRZ) (Figure 41) emitter with a ΔE_ST_ of 0.37 eV by controlling the length of donor and acceptor moieties and increasing the D–A distance. The BCz-TRZ-based device exhibited a lifetime (LT_50_) of 495 h and an EQE_max_ of 20% at an emission wavelength of 486 nm [114].

##### Carbazole and Phthalonitrile (Cz-PN) Derivatives

Phthalonitrile-derivative compounds have attracted considerable attention because of their strong electron-withdrawing nature. Phthalonitrile-functionalized aromatic compounds are widely used as electron-transporting, charge-generation, and light-emitting materials in OLEDs. To achieve bipolarity in the molecular design of phthalonitrile-based aromatic compounds, such as bipolar hosts and TADF emitters, strong electron donor carbazole units are appended onto the strong electron donor phthalonitrile unit [271,272].

**Figure 42:**
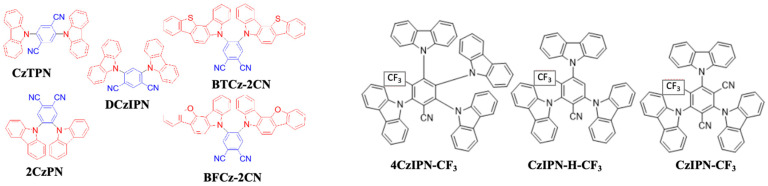
Molecular structures of Cz-PN-based blue emitters.

In 2012, Uoyama et al. designed a series of carbazole and phthalonitrile materials. Among them, 4,5-di(9H-carbazol-9-yl) phthalonitrile (CzTPN) (Figure 42) realized a sky-blue emission. The CzTPN exhibited a ΔE_ST_ of 0.29 eV with a 47% PLQY. In a doped device with a CBP host, the CzTPN emitter exhibited a maximum EQE of 8% for a sky-blue emission with an emission wavelength at 473 nm [271]. Furthermore, in 2013, Masui et al. achieved a maximum EQE of 13% with (0.17, 0.30) by doping the CzTPN emitter into an mCP host [64].

In 2015, Lee et al. reported a maximum EQE of 16% for a blue device with (0.17, 0.19) by doping a 15 wt% 4,6-di(9H-carbazol-9-yl)isophthalonitrile (DCzIPN) (Figure 42) in a mCP host [273]. In the same year, Lee et al. designed and synthesized two blue emitters, 4,5-bis(benzofuro[3,2-c]carbazol-5-yl)phthalonitrile (BTCz-2CN) and 4,5-bis(benzo[4,5]thieno [3,2-c]carbazol-5-yl)phthalonitrile (BFCz-2CN) (Figure 42), by employing benzofuro- and benzothieno-functionalized carbazole units with a phthalonitrile unit. BTCz-2CN and BFCz-2CN exhibited a ΔE_ST_ value of 0.13 and 0.17 eV with a 95 and 94% PLQY, respectively. The BTCz-2CN showed a decay lifetime of 2.6 μs, which is larger than that of the BFCz-2CN counterpart (1.98 μs). The short decay time of the BTCz-2CN and BFCz-2CN may be attributed to their small ΔE_ST_. Moreover, BTCz-2CN and BFCz-2CN also showed a high T_g_ of 204 and 219 °C and T_d_ of 397 and 434 °C, respectively. In doped devices with an mCP host, the BTCz-2CN- and BFCz-2CN-based devices resulted in a maximum EQE of 12.1 and 11.8% with a similar EL emission peaking at 486 nm [274].

In 2020, Yi et al. reported three molecules, namely, 4CzIPN-CF_3_, 3CzIPN-H-CF_3_, and 3CzIPN-CF_3_ (Figure 42). They were synthesized by lowering the electron-withdrawing ability of the acceptor group or by reducing the number of carbazole donors of the base molecule, 1,2,3,5-tetrakis(carbazol-9-yl)-4,6-dicyanobenzene (4CzIPN). Among them, the 4CzIPN-CF_3_- and 3CzIPN-H-CF_3_-based devices with a SimCP:oCF3-T2T co-host system displayed a blue-shifted emission with coordinates (0.21, 0.41) and (0.16, 0.19), respectively, as compared with a 4CzIPN emission (0.22, 0.48). The devices showed an EQE_max_ of 23 and 17%, and 21 and 15% at 1000 nits, respectively [275].

##### Acridine and Phenoxaborin (Cz-PXB) Derivatives

**Figure 43:**
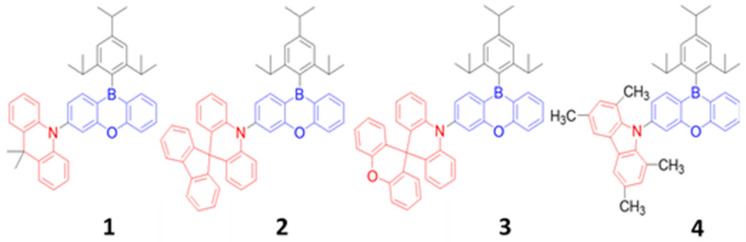
Molecular structures of Cz-PXB-based blue emitters.

In 2015, Numata et al. synthesized a series of D–A-type emitters (1-4) (Figure 43) with acridine derivatives as donor units and a 10H-phenoxaborin-type acceptor [276]. The resultant maximum EQE ranged from 20 to 13%, while chromaticity coordinates for a sky-blue emission ranged from (0.14, 0.23) to deep-blue (0.15, 0.08) by doping a 20 wt% of 1–4 emitters into an 8-bis(diphenylphosphine oxide) dibenzofuran (PPF) host. Moreover, the resulting sky-blue device also demonstrated a maximum EQE of 22% when the doping concentration of emitter 1 was further increased to 50 wt% in the same PPF host. The high efficiencies may be attributed to three key factors. First, the ACR-PXB series emitters possessed a higher PLQY value ranging from 56 to 100%, a shorter decay time ranging from 1.6 to 4.02 μs, and a smaller ΔE_ST_ value ranging from 0 to 0.16 eV. Second, rational bipolar characteristics of the emitters may help to achieve an effective charge carrier injection balance in the emissive layer. Third, the high triplet energy (3.21 eV)-host material restricted any backward energy transfer and facilitated an effective host-to-guest energy transfer. As reported, the device performance was greatly influenced by the ΔE_ST_ and PLQY of the emitters. The PLQY increased from 56 to 100% for emitter 1 as ΔE_ST_ decreased from 0.16 to 0 eV [276].

##### Phenoxazines and Triazole (PXZ-TAZ) Derivatives

**Figure 44:**
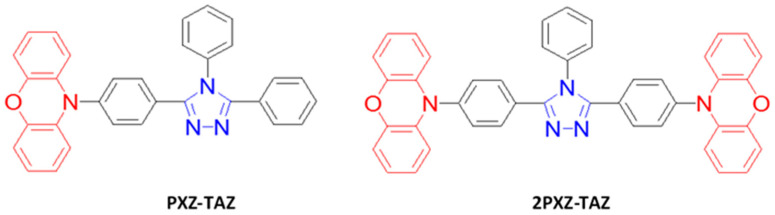
Molecular structures of PXZ-TAZ-based blue emitters.

Bipolar compounds based on phenoxazine electron-donor and triazole electron-acceptor moieties have been widely investigated to design the bipolar host materials for OLEDs [refs]. In 2013, Adachi et al. reported two TADF blue emitters, 10-(4-(4,5-diphenyl-4H-1,2,4-triazol-3-yl)phenyl)-10H-phenoxazine (PXZ-TAZ) and 10,10′-((4-phenyl-4H-1,2,4-triazole-3,5-diyl)bis(4,1-phenylene))-2-bis(10H-phenoxazine) (2PXZ-TAZ) (Figure 44), by using 1,2,4-triazole core-based emitters with a 10H-phenoxazine group. The PXZ-TAZ and 2PXZ-TAZ emitters exhibited a PLQY of 13 and 15% in neat films. The PLQY was greatly enhanced from 15 to 52% as a 6 wt% 2PXZ-TAZ emitter was doped into a DPEPO host. The 2PXZ-TAZ also showed a maximum EQE of 6.4% with (0.16, 0.15) and an EL emission peaking at 456 nm [65].

##### Acridine and Triazine (ACR-TRZ) Derivatives

**Figure 45:**
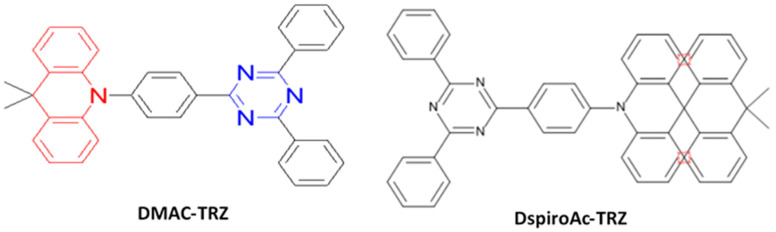
Molecular structures of ACR-TRZ-based blue emitters.

As described in prior sections, both phenoxazine and triazine moieties have been extensively utilized to design bipolar hosts and TADF emitters for OLEDs. In 2015, Tsai et al. designed and synthesized a DMAC-TRZ (Figure 45) emitter based on 9,9-dimethyl-9,10-dihydroacridine as a donor unit and 2,4,6-triphenyl-1,3,5-triazine as an acceptor unit. The DMAC-TRZ emitter showed a ΔE_ST_ of 0.05 eV and a delay lifetime of 3.6 μs with an 83% PLQY. Furthermore, the ΔE_ST_ and the delay lifetime were decreased to 0.046 eV and 1.9 μs, respectively, while the PLQY was increased to 90% as the DMAC-TRZ emitter was doped into a 9-(3-(9H-carbazol-9-yl)phenyl)-9H-carbazole-3-carbonitrile (mCPCN) host. At 100 nits, the resultant non-doped DMAC-TRZ-based device exhibited a maximum EQE of 20% with a bluish-green emission, while a doped emissive layer (at 8 wt% DMAC-TRZ and mCPCN)-based device showed a maximum EQE of 27% with a slightly blue shifted emission. These devices also exhibited relatively high power efficacy, at 46 and 66 lm/W, and current efficacy of 61 and 67 cd/A at 100 cd/m^2^, respectively, for non-doped and doped emissive layer-based devices [277].

In 2020, Li et al. reported an emitter, DspiroAc-TRZ (Figure 45), with a tight and regular intermolecular packing composed of X- or L-shaped J-aggregates. PLQYs of 79 and 84% were obtained for the DspiroAc-TRZ crystal and nondoped film, respectively, due to the synergistic effect of the intermolecular packing mode and monomolecular structure. Thus, a very high EQE_max_ of 26% and CE_max_ of 56 cd/A were realized for a sky-blue emission with (0.17, 0.35) by using a host-free emissive layer [278].

#### 6.3.2. Metal-Based TADF Emitters

Metal-based TADF blue emitters had also been reported to realize a very small ΔE_ST_, which can effectively up-convert the entire fraction of triplet excitons (75%) into singlet excitons via ultra-fast reverse intersystem crossing (RISC) [279,280,281,282]. This prudent combination of ultra-fast RISC and a very small ΔE_ST_ would result in a 100% delayed fluorescence yield, [282,283,284,285] and significantly diminish the efficiency roll-off, especially at high luminance or current density [286].

**Figure 46:**
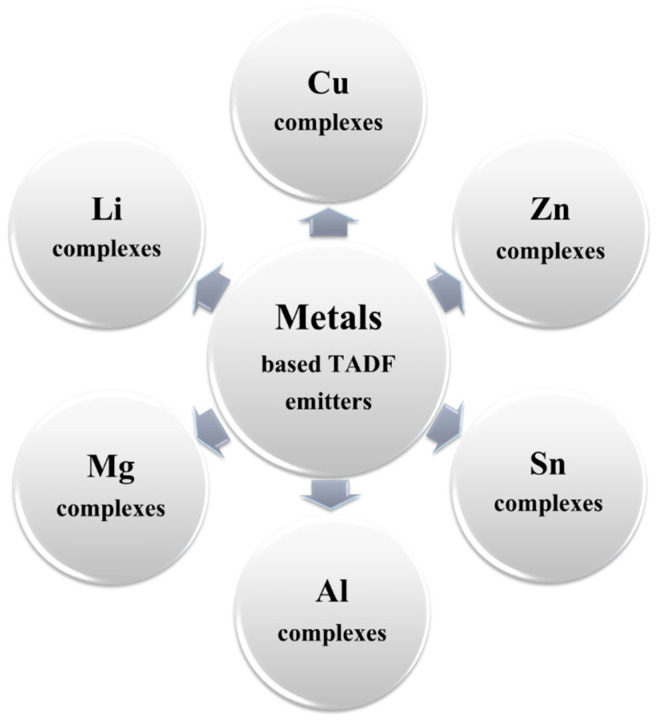
Types of non-precious metal-based TADF blue emitters.

The metal-based TADF emitters also exhibited a higher thermal stability than that of pure aromatic-based counterparts [286,287,288,289]. By virtue of these advantages, a considerable number of blue TADF emitters based on non-precious and abundant metals, such as lithium (Li), magnesium (Mg), copper (Cu), zinc (Zn), and tin (Sn), were reported (Figure 46) [290]. However, only a few of them were investigated in OLEDs [239,291,292,293,294,295,296,297].

##### Cu Complexes

Copper (Cu) is one of the highly abundant coinage metals which has been investigated in the design of high-electroluminescence (EL) active complexes for low-cost OLEDs. In order to realize a sustainable way out, Ma et al. designed and synthesized the first Cu complex-based emitter in 1999 [298,299]. Later, many Cu complex-based phosphorescent emitters were reported because of their ability to realize a high rate of intersystem crossing (ISC) (S1→T1) and triplet radiative decay (T1→S0) [282,300].

Due to a small ΔE_ST_, a few Cu complexes showed effective RISC (T1→S1) and singlet radiative decay (S1→S0) rates [294,301]. These complexes exhibited a small ΔE_ST_ and a fast RISC because of low-lying metal-ligand charge transfer (MLCT) excited states, which played a crucial role in realizing an effective triplet up-conversion [291,292,293,294,295,296,297].

**Figure 47:**
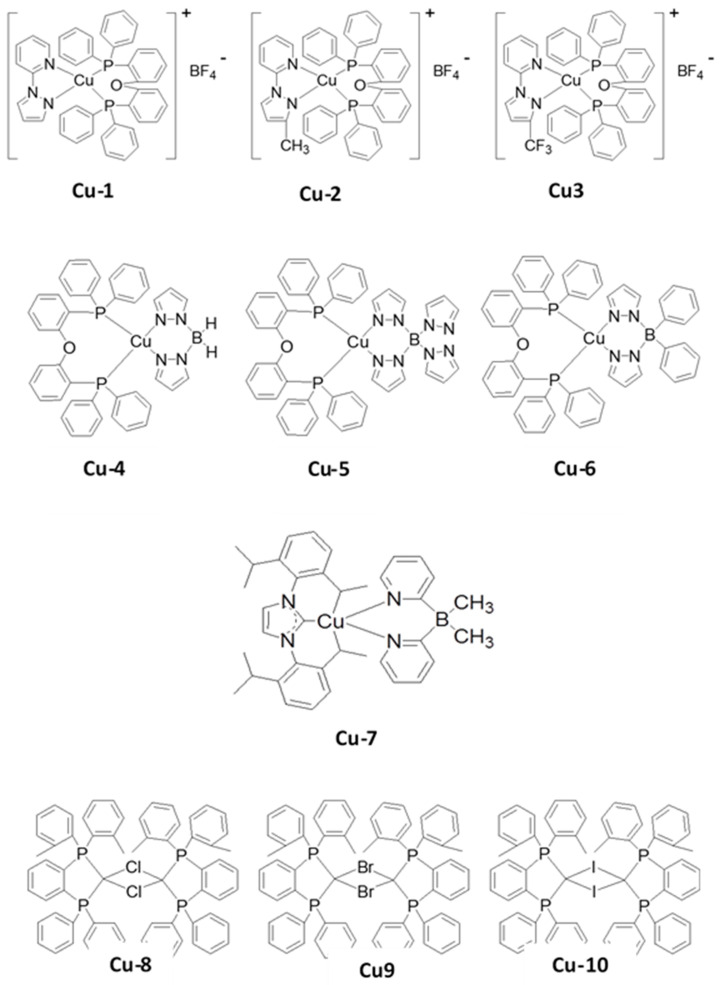
Molecular structures of copper (Cu)-based blue emitters.

Over the past few years, several Cu-based TADF emitters have been utilized in OLEDs [302,303,304,305,306,307,308,309,310]. Among them, only a few emitters demonstrated a blue emission. In 2011, Yersin et al. designed and synthesized a series of Cu (I) complexes (Cu-4 to Cu-6) (Figure 47) [298]. The ΔE_ST_ and PLQY of these compounds ranged from 0.09 to 0.16 eV and 45 to 90%, respectively, in solid state films. The chromaticity coordinates of the Cu-4 to Cu-6 emitters ranged from (0.14, 0.11) to (0.16, 0.22). In the same year, Leitl et al. reported a design strategy to realize a strong TADF emission in another Cu complex [311]. They observed that a small ΔE_ST_ value can be obtained by selecting heteroleptic ligands with a low torsion angle. A Cu complex (Cu-7) with two N-heterocyclic ligands, 1,3-bis(2,6-diisopropylphenyl)imidazol-2-ylidene and di(2-pyridyl)dimethylborate), showed a blue emission peaking at 475 nm. The Cu-7 exhibited a solid state PLQY of 70% and a ΔE_ST_ of 0.09 eV, resulting in a TADF decay fraction of 62% with a weak phosphorescence decay fraction of 38% [311].

In 2013, Chen et al. reported a series of Cu complex (Cu-1 to Cu-3) (Figure 47)-based emitters [312]. These complexes showed a constant ΔE_ST_ of 0.17 ± 0.01 eV with a PLQY ranging from 56 to 87%. Via wet-processing, the resultant maximum EQE ranged from 8.5 to 1.5%, with chromaticity coordinates ranging from greenish-blue (0.24, 0.36) to sky-blue (0.20, 0.30) as a 20 wt% of the emitters was doped into a hole-transporting host 26mCPy. The Cu-3 complex-based device also exhibited a maximum current efficiency of 24 cd/A.

In 2014, Yersin et al. reported a series of Cu complex-based emitters. Their structures were similar to the phosphorescent emitters reported earlier [311,313].

In 2015, Kang et al. reported a series of binuclear Cu halide complexes (Cu-8–Cu-10) (Figure 47) with different halogen molecules (Cl, Br, and I) [291]. These complexes exhibited a ΔE_ST_ value from 0.05 to 0.04 eV, a PLQY from 42 to 95%, and a decay time ranging from 4.9 to 5.9 μs with an emission wavelength ranging from blue (482 nm) to greenish blue (490 nm).

##### Zn Complexes

Over the past few years, several Zn complexes have been employed as EL-active emitters, hosts, and electron transporting materials in OLEDs [235,314,315,316,317,318,319,320,321,322]. However, Zn complexes exhibited a poorer device performance than that of Cu complex-based counterparts while displaying a higher stability against atmospheric moisture and air. Therefore, numerous efforts have been devoted to design Zn complex-based TADF emitters [296,323,324].

**Figure 48:**
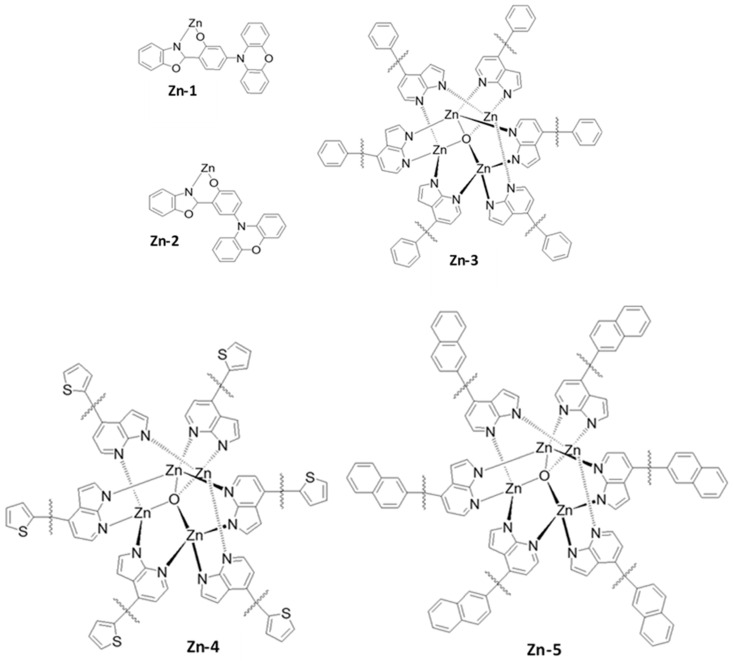
Molecular structures of zinc (Zn)-based blue emitters.

In order to devise a highly stable metal complex-based emitter, in 2015, Adachi’s group reported two different Zn complexes (Zn-1 and Zn-2) (Figure 48) with blue emission [286]. The Zn-1 and Zn-2 complexes demonstrated a PLQY of 78 and 58% and a ΔE_ST_ of 0.06 and 0.18 eV, respectively. In addition, the Zn-1 emitter also realized a low TADF decay time of 38 μs. In an mCBP host-based doped device, the Zn-1 complex showed a maximum EQE of 20% with a greenish-blue emission.

Moreover, Cheng et al. designed and synthesized a series of tetranuclear zinc(II) complexes of substituted 7-azaindoles. The decay time and PLQY of these complexes ranged from 0.015 to 0.008 μs and 96 to 71%, respectively, as a 5 wt% emitter was doped into a PMMA host. The PLQY values of these compounds were measured in solid state film. The resultant maximum EQE ranged from 5.6 to 3.3%, with chromaticity coordinates ranging from (0.16, 0.19) to (0.18, 0.26) as an 8 wt% of the emitters was doped into a mixture of PVK and OXD-7 hosts [325].

##### Other Metal Complexes

**Figure 49:**
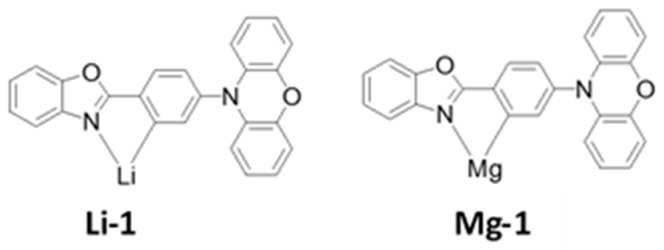
Molecular structures of Lithium (Li) and Magnesium (Mg)-based blue emitters.

In 2015, Adachi et al. studied other metal (Li, Mg, Al, and Sn) complexes for OLED device applications [286]. The lithium (Li) complex-based TADF emitter (Li-1) exhibited a ΔE_ST_ of 0.08 eV with a total PLQY of 70%. In a doped device with an mCBP host, the Li-1 (Figure 49) realized a maximum EQE of 13% with a greenish-blue emission. Moreover, a magnesium-based (Mg-1) (Figure 49) complex achieved a ΔE_ST_ of 0.08 eV and a PLQY of 71%, including a 58% delayed quantum yield. In a device doped with an mCBP host, the Mg-1 complex-based device exhibited a maximum EQE of 17% for a greenish-blue emission.

## 7. Challenges and Approaches

As seen, high efficiency and long lifetime had been reported for devices of light-blue emission, i.e., CIE_y_ > 0.1, using phosphorescent and TADF-based emitters. Even still, a host-free device displayed a much higher efficiency (EQE_max_: 25%) for a greenish-blue emission with (0.16, 0.24) coordinates [109]. Moreover, a very high EQE_max_ of 33% was reported for two different devices, one with a greenish-blue emission at CIE_xy_ (0.16, 0.22) [107] and the other with a bluish-green emission at (0.17, 0.33) [106]. The longest lifetime of 100,000 h (LT_50_) at 200 nits was reported for a bluish-green emission with a CIEy of 0.38 [97]. However, obtainina high efficiency and a long lifetime in the deep-blue region (CIE_y_ < 0.1) remains challenging.

### 7.1. Challenges for Deep-Blue Emission

Researchers have focused on designing and synthesizing suitable emitters for both blue and deep-blue OLEDs. Employment of various ligands/subunits in a molecule helps to achieve a comparatively high efficiency and long lifetime for blue emission. However, only a few displayed a moderate efficiency or lifetime in the deep-blue region.

#### 7.1.1. High-Efficiency Deep-Blue Emission

As discussed earlier, phosphorescent and TADF-based devices displayed EQE > 20% for a deep-blue emission. For example, an EQE of 25% with a CIE_y_ of 0.08, but at a luminance below 100 nits, was reported by using a phosphorescent emitter, TSP01 [86]. Moreover, an EQE of 20% with a CIE_y_ of 0.03, but also at a luminance below 100 nits, was reported by using a host-free TADF emitter, NTN-PCZ [104]. However, Lim et al. reported an EQE_max_ of 28% with a CIE_y_ of 0.09 crossing the upper theoretical limit for TADF emitters by using a TDBA-SAF TADF emitter. Most importantly, an EQE of 24% was reported at 100 nits and 18% at 1000 nits [105]. Realizing a high EQE with a CIE_y_ much less than 0.1 at applicable high luminance remains challenging.

#### 7.1.2. Long-Lifetime Deep-Blue Emission

A deep-blue fluorescent device with a CIE_y_ of 0.09 was created by Takita et al. with a lifetime of 125 h (LT_90_) at 1000 nits [72]. Before 2021, no phosphorescent or TADF-based devices had displayed any lifetime data in the deep-blue region. In 2021, there were two articles reporting deep-blue devices with a CIE_y_ of 0.09. Nam et al. reported a deep-blue device with a CIE_y_ of 0.09 and a lifetime of 254 h (LT_50_) at 1000 nits by using a phosphorescent sensitizer with a fluorescent emitter [326]. Jeon et al. reported another deep-blue device with a CIE_y_ of 0.09 and a lifetime of 151 ± 3 h (LT_50_) at 1000 nits by using a TADF emitter [327]. However, achieving a long lifetime is still required.

#### 7.1.3. High Efficiency and Long Lifetime

As shown above, the deep-blue fluorescent device reported by Takita et al. also displayed an EQE_max_ of 12%, beyond the theoretical limit for fluorescent emitters [72]. Since then, no devices were reported with a high efficiency and long lifetime for a deep-blue emission until 2020. In 2021, Jeon et al. reported a deep-blue device with a CIE_y_ of 0.09, displaying a higher EQE_max_ of 34% and a lifetime of 151 ± 3 h (LT_50_) at 1000 nits by using a TADF emitter [327]. However, realizing a deep-blue device with a long lifetime along with high efficiency is still essential.

### 7.2. Approaches for Generating Deep-Blue Emission

In the past decades, a few approaches were reported for realizing a deep-blue emission in OLEDs. They include but are not limited to the following: (i) reducing dopant concentration in the emissive layer, (ii) using a polarity-matching host, (iii) rational emitter designs, (iv) thermally activated delayed fluorescence, (v) hybrid local charge transfer, and (vi) triplet–triplet annihilation.

#### 7.2.1. Reducing Dopant Concentration in the Emissive Layer

Reports have confirmed that an undesirable, red-shifted emission, which is induced due to self-aggregation of emitter molecules, can be greatly suppressed by dispersing into a host material [51]. Unlike rational molecular design, the self-aggregation effect in the emitter molecules can be effectively diminished by decreasing their concentration in the host [168]. Jou et al. reported a blue emission peaking at 448 nm when a pure cyano fluorene acetylene-conjugate C3FLA-2 was employed as an emissive layer. The resultant blue emission peak exhibited a strong hypsochromic-shift from 448 to 428 nm when a 7 wt% C3FLA-2 emitter was doped into a CBP host [51]. The employed host can be utilized to disperse the dopant to prevent the bathochromic shift caused by emitter aggregation. The dispersion is more effective when the dopant is more diluted [328], as indicated by the main emission peak, which was blue-shifted from 428 to 416 nm as the C3FLA-2 concentration was decreased from 7 to 1 wt% [51]. The same group reported triphenylamine and carbazole-based emitters. Compared with the devices based on the neat films of these emitters, the EL shifted towards blue as they were dispersed in a CBP host. Deep-blue emission with high efficiency was obtained with no observable red shift for doping concentration up to 9 wt% [168].

Chen et al. reported three blue emitters, TPIBCz, TPINCz, and TPIBNCz, with an EL emission ranging between 435 and 448 nm for undoped devices. When a 30 wt% of these emitters was doped into a CBP host, a hypsochromic shift was observed with emission ranging between 428 and 440 nm. Furthermore, on reducing the dopant concentration to 10 wt%, their main emission peaks further blue-shifted to between 424 and 436 nm [329].

#### 7.2.2. Employing a Polarity Matching Host

Researchers found that a deep blue emission can also be obtained from sky-blue and/or blue emitters by employing a polarity matching host for the desirable emitters. Jou et al. achieved a deep-blue emission as a sky-blue emitter 1-((9,9-diethyl-9H-fluoren-2-yl)ethynyl)pyrene (PA-1) was doped into a polarity matching host CBP. The emitter PA-1 showed a relatively low polarity of 0.8 D, whilst CBP showed almost no polarity with a dipole moment of 0.0 D. Therefore, the high similarity between the low polarities of CBP and PA-1 could enhance emitter solubility. The resultant device showed a CIE_y_ of 0.06 [162].

Chen et al. reported three blue emitters, TPIBCz, TPINCz, and TPIBNCz, with an EL emission at 435, 448, and 436 nm and a low polarity of 8.2, 13, and 9.6 D, respectively. When doped into a non-polar host CBP, the three emitters exhibited a blue-shifted emission at 428, 436, and 424 nm, respectively. As said, the host decreased the overall polarity of the medium and hence resulted in a blue shift [329].

#### 7.2.3. Rational Emitter Designs

We have reviewed herein two rational designs for synthesizing deep-blue emitters. The two basic principles are to minimize the undesired intramolecular charge transfer (ICT) and shorten π-conjugation or resonance length, which may cause a red-shift in the emission under electrical excitation. The extent of the ICT effect can be minimized with the introduction of a weak D–A pair [330].

Incorporating carbazole moieties in a molecule is another way to achieve a deeper-blue emission due to their properties such as good hole-transporting, large bandgap, and weaker π-donating ability [331,332,333,334]. Jou’s group reported a deep-blue fluorescent emitter, JV234, and the composing device displayed a CIE_y_ of 0.06 [335]. The same group reported another deep-blue fluorescent emitter, JV55, and the device showed an EQE_max_ of 6.5% with a CIE_y_ of 0.06 [200].

In addition to the carbazole moieties, incorporating oxadiazole as electron-withdrawing moiety builds a bipolar molecule. The carbazole moiety helps reduce the ICT effect and blue shifts the emission. Wang et al. reported such a molecule, namely, Oxd-bCz, and the composing device showed a CIE_y_ of 0.07 [203]. Meanwhile, Huang et al. reported that a benzofuro [2,3-b]pyrazine unit (BFPz) as a withdrawing moiety along with a carbazole moiety can help achieve a CIE_y_ of 0.05 [336].

Furthermore, Pal et al. reported two homoleptic charge-neutral meridional (mer-) complexes (1 and 2). These complexes contain a CF_3_ group as an electron-withdrawing group on the cyclometalating aryl group. The only difference in their structures was that in complex 1, the CF_3_ substitution was in a more electron-withdrawing para position with respect to the C-Ir bond. Both complexes showed a deep-blue emission. Complex 2 as an emission layer showed a CIE_y_ of 0.05 and a high PLQY of 72% [337]. Wang et al. reported that a limited π-conjugation length was required for achieving a deep-blue emission. They hence used a spiro-diphenylsulfone-based TADF emitter that had a short π-conjugation length to acheive a deep-blue emission with a CIE_y_ of 0.04 for an inverted OLED [338].

Such a design strategy thus offers a formulated platform to generate functional luminescent materials with efficient deep blue emissions. These rational design features are necessary, especially for devices with a non-doped emissive layer.

#### 7.2.4. Thermally Activated Delayed Fluorescence

Weissenseel et al. reported two deep-blue emitters composed of two donor and two acceptor units. The central core consisted of two acridine units linked with a spiro-bridge. The perpendicular conformation between the donor and acceptor moieties reduced the HOMO LUMO gap which resulted in a small singlet-triplet gap. The device with a 10 wt% SBABz4 doped in a DPEPO host showed a CIE_y_ of 0.09 [339].

Chan et al. reported four blue emitters, DCzBN1-4, based on 3,6-substituted carbazole donor and benzonitrile acceptor units. The ΔE_ST_ was tuned by modifying the substituent on the donor moieties. The emitters exhibited an emission ranging between 404 and 470 nm. Among them, the device based on DCzBN3 that had a di-tert-butylcarbazole donor unit displayed an EQE_max_ of 10% and a CIE_y_ of 0.06 [340].

Wang et al. reported a spiro-diphenylsulphone-based TADF emitter that emitted in a deep-blue region. The non-doped device showed a low efficiency roll-off and achieved an EQE_max_ of 5.0 and 2.1% at 1000 nits with a CIE_y_ of 0.04 [338].

Liang et al. reported four trifluoromethyl-based compounds, namely, TN3T-PCZ, TN4T-PCZ, DTF-PCZ, and NTN-PCZ. Among them, the device based on the TN4T-PCZ emitter displayed an EQE_max_ of 20% and a CIE_y_ of 0.03 [104].

Lim et al. reported a deep-blue emitter, TDBA-SAF, based on a spiro-acridine fluorene donor and an oxygen-bridged boron acceptor unit. The composed device displayed an EQE_max_ of 28% and a CIE_y_ of 0.09 [105].

Luo et al. reported a UV-emissive TADF compound, CZ-MPS, by interlocking a diphenylsulfone acceptor unit with a dimethylmethylene bridge. The composed device exhibited an EL emission at 389 nm and an EQE_max_ of 9.3% [341].

Gudeika et al. reported a deep-blue TADF compound, di-tert-butyl-dimethylacridinyl disubstituted oxygafluorene, dispersed in an inert polymer under a nitrogen atmosphere. The composed device displayed an EQE_max_ of 9.9% with a CIE_y_ of 0.08 [342].

Kim et al. reported three color tunable emitters, 9′-(2,12-di-tert-butyl-5,9-dioxa-13b-boranaphtho[3,2,1-de]anthracen-7-yl)-9′H-9,3′:6′,9″-tercarbazole (TB-3Cz), 9′-(2,12-di-tert-butyl-5,9-dioxa-13b-boranaphtho[3,2,1-de]anthracen-7-yl)-9,9″-diphenyl-9H,9′H,9″H-3,3′:6′,3″-tercarbazole (TB-P3Cz), and 9-(2,12-di-tert-butyl-5,9-dioxa-13b-boranaphtho[3,2,1-de]anthracen-7-yl)-N3,N3,N6,N6-tetraphenyl-9H-carbazole-3,6-diamine (TB-DACz). The devices based on TP-3Cz and TB-P3Cz emitters exhibited a deep-blue emission with a CIE_y_ of 0.07 and 0.08, respectively. An EQE_max_ of 9.9% for TP-3Cz and 6.0% for TB-P3Cz were also observed [108].

#### 7.2.5. Hybrid Local Charge Transfer

Yang et al. reported a deep-blue HLCT luminogen Cz-TPB, based on triphenylbenzene. The material possessed an exciton-utilization efficiency (EUE) of 86%. The non-doped device based on this emitter displayed an EQE_max_ of 4.3% and a CIE_y_ of 0.09. As reported by the authors, the additional triplet excitons were effectively utilized due to the small ΔE_S1-T2_ of the emitter [343].

Fu et al. reported an HLCT emitter based on a pyrene structure. The composed device displayed an EUE of nearly 100%, an EQE of 11%, and a CIE_y_ of 0.06 [344].

Yu et al. reported three emitters, TPA-1PI, TPA-2PI, and TPA-3PI, based on a phenanthro[9,10-d]imidazole (PI) chromophore. All the three emitters displayed HLCT characteristics. Among them, the device based on TPA-2PI displayed a CIE_y_ of 0.08 [345].

Usta et al. reported a deep-blue HLCT emitter, 1,4-bis((2-cyanophenyl)ethynyl)-2,5-bis(2-ethylhexyloxy)benzene (2EHO–CNPE), based on an oligo(p-phenyleneethynylene) small molecule. The material possessed a wide bandgap of 2.9 eV. The composed device displayed an EQE_max_ of 7.1% and a CIE_y_ of 0.09 [346].

Chen et al. reported deep-blue HLCT emitters, TPINCz and TPIBNCz, based on naphthyl group(s) as a weak n-type π space in a donor–π–acceptor (D-π-A) system. The device based on TPINCz displayed an EQE_max_ of 6.6% with a CIE_y_ of 0.05 [329].

Most recently, Zhang et al. reported an HLCT compound, 2TA-PPI, by grafting triphenylamine moieties at -C5 and -C10 positions of a 1,2-diphenyl-1H-phenanthro[9,10-d]imidazole (PPI) unit. The composed device displayed an EQE_max_ of 11% and a CIE_y_ of 0.09 [347].

#### 7.2.6. Triplet–Triplet Annihilation

**Figure 50:**
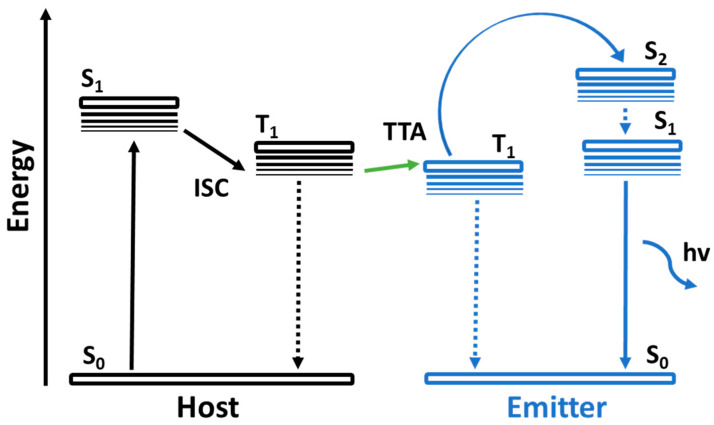
Jablonski diagram of a postulated TTA mechanism. The abbreviations used are S_0_: ground state, S_1_ and S_2_: excited singlet state, T_1_: triplet state, ISC: intersystem crossing, TTA: triplet–triplet annihilation, and hv: emitted photon.

As postulated/reported by Kondakov et al., TTA might occur when two excited electrons in the triplet state interact with each other, coupling with the occurrence of an up-conversion mechanism [348]. With that, one low-level and one high-level triplet-exciton are produced. The low-level triplet exciton returns to the ground state (S_0_) of the emitter while the other is excited to the higher-level singlet state, S_2_. After fast intermolecular relaxation (S_2_ − S_1_), the S_1_ state is re-populated, which is beneficial for the high efficiency of fluorescent emitters (Figure 50) [349].

Hu et al. reported three compounds, 1,4-bis(10-phenylanthracene-9-yl)benzene (BD1), 1-(10-phenylanthracen-9-yl)-4-(10-(4-cyanophenyl)anthracen-9-yl)-benzene (BD2), and 1-(10-(4-methoxyphenyl)anthracen-9-yl)-4-(10-(4-cyanophenyl)anthracen-9-yl)benzene (BD3). Among them, the doped device based on a BD3 emitter and CBP host displayed an EQE_max_ of 12% and a CIE_y_ of 0.06. The enhanced EQE is attributed to the occurrence of the TTA mechanism [350].

Chen et al. reported an approach to developing TTA materials for high-efficiency fluorescence via a styrylpyrene core and an electron-donating group. The device with a PCzSP emitter doped in CBP host displayed a 9.1% EQE_max_ and a CIE_y_ of 0.09 [351].

Zeng et al. reported two quaterphenyl derivatives with a PLQY of nearly 80%. The nondoped device based on a 4PS emitter displayed an EQE_max_ of 5.9% and CIE_y_ of 0.06. The doped device displayed an EQE_max_ of 6.6% corresponding to a CIE_y_ of 0.05. As said by the authors, the TTA enabled the slow roll-off in the device [352].

Peng et al. reported dianthracenylphenylene-based emitters. The non-doped devices based on these emitters displayed an EQE_max_ of 4.6 to 5.9% corresponding to a CIE_y_ from 0.07 to 0.08. They have attributed the good device performance to TTA occurrence [353].

Recently, Wu et al. reported two anthracene-based molecules, 4-(10-(3-(9H-carbazol-9-yl)phenyl)-2,6-di-tert-butylanthracen-9-yl)benzonitrile (mCz-TAn-CN) and 4-(2,6-di-tert-butyl-10-(3,5-di(9H-carbazol-9-yl)phenyl)anthracen-9-yl)benzonitrile (m2Cz-TAn-CN). Among them, the doped device based on the m2Cz-TAn-CN emitter showed an EQE_max_ of 7.3% and CIE_y_ of 0.09. They also attributed the efficiency improvement to TTA occurrence [354].

## 8. Cautions

Although high-efficiency and long-lifetime deep blue emitters have some advantages, such as reducing the pixel size/number of pixels in a display or fabricating a high-quality light for lighting, they might have some drawbacks if used inappropriately. Intense blue light tends to disrupt ecosystems, pollute night skies, suppress melatonin secretion, discolor artifacts, and cause numerous eye diseases as well as cancers.

Professor Jou, also the corresponding author of this review article, from National Tsing Hua University has recommended several precautions regarding how to avoid blue-light hazards and safeguard the health of eyes and physiologies in the popular science speeches he has delivered over 100 times in the past 10 years and the book entitled “Embracing Darkness” [355]. He has mentioned five important points to be kept in mind and followed seriously. These are:

(i) Turn off or dim the light after dusk or at least 2 h before going to bed.
(ii) Change indoor illumination to a blue light-free candlelight style lighting for nighttime.
(iii) Avoid the use of 3C products before going to bed. Audio activities are preferred over video ones.
(iv) Embrace darkness. The bedroom should be completely dark during sleep.
(v) Consult physicians for melatonin supplements for sleep improvement, especially for older persons.

Precautions are always better than cures. Now is the right time to develop a good habit of “staying in darkness at night”.

## 9. Conclusions

In summary, this review depicts the global revenue and forecast for lighting and display technologies. Since 2018, OLED display revenues have surpassed those of LCD displays, proving which truly is a disruptive display technology. However, OLED lighting is not yet successful as compared against LED lighting, although its revenue is rising gradually. Until 2023, an increment of 41%, i.e., a forecasted amount of 99 billion, is expected annually.

Overall, the number of papers published and patents filed for blue OLEDs have increased significantly over the past years, especially those based on Gen-3 materials, confirming the need for blue OLEDs. It is also surprising to see that publications based on Gen-1 materials rose significantly in 2021, indicating the importance of deep-blue emitters. Some progress was seen in the development of deep-blue OLEDs with high efficiency and/or long lifetime, increasing the possibility of mass production of displays with a higher resolution that are also more energy-saving and reliable.

Having high efficiency along with a long lifetime still remains challenging, although there are several approaches available for realizing deep-blue emission.

Furthermore, while the marked progress on deep-blue OLEDs is delightful, it is strongly suggested that we keep one eye on the plausible negative impacts that might result from the same, and embracing darkness is always essential after dusk to safeguard human health and ecosystems.

## Figures and Tables

**Table 1:** Commercial OLED lighting panels.

Company	Dimensions (mm)	η_p_ (lm/W)	Luminance (cd/m^2^)	Lifetime (h)	Reference
Philips	124 × 124.5 × 3.3	16.7	4000	10,000 @ L50	[32]
Osram	Φ75 mm	35.0	2000	10,000 @ L50	[33]
Lumiotech	145 × 145	45.0	3000	40,000 @ L70	[36]
Konica Minolta	21.4 × 25.9	-	500	-	[38]
Mitsubishi Chem.	55 × 55 × 1.08	-	2000	-	[39]
Kaneka	80 × 80 × 1.05	-	3000	50,000 @ -	[40]
LG Chem.	200 × 50 × 0.88	60.0	3000	40,000 @ L70	[34]

**Table 2:** Efficiency and lifetime performance of dry-processed fluorescent blue OLEDs. All the lifetime data have been converted to the same 1000 nits for easy comparison.

CIEy	EQE_max_ (%)	L_0_ (cd/m^2^)	Lifetime (h)	Lifetime (h) @L_0_:1000 (cd/m^2^)	CE_max_ (cd/A)	Year	Reference
0.09	11.8	1000	125 (LT_90_)	125 (LT_90_)	9.2	2018	[72]
0.11	8.6	1176	110 (LT_50_)	121 (LT_50_)	4.9	2010	[83]
0.15	8.2	5000	168 (LT_50_)	458 (LT_50_)	9.1	2010	[82]
0.17	3.0	100	5600 (LT_50_)	1330 (LT_50_)	4.0	2005	[84]
0.22	4.9	1000	78 (LT_80_)	78 (LT_80_)	7.1	2020	[85]
0.32	1.5	100	7000 (LT_50_)	1662 (LT_50_)	9.7	2005	[84]

L_0_: Initial luminance.

**Table 3:** Efficiency and lifetime performance of dry-processed phosphorescent blue OLEDs. All the lifetime data have been converted to the same 1000 nits for easy comparison.

CIEy	EQE_max_ (%)	L_0_ (cd/m^2^)	Lifetime (h)	Lifetime (h) @L_0_:1000 (cd/m^2^)	CE_max_ (cd/A)	Year	Reference
0.13	27.6	100	10,000 (LT_50_)	2375 (LT_50_)	-	2020	[99]
0.16	25	327	2203 (LT_50_)	1095 (LT_50_)	42.6	2017	[87]
0.23	5.5	200	2.9 (LT_75_)	1 (LT_75_)	6.3	2019	[100]
0.23	5.9	200	2.6 (LT_75_)	0.9 (LT_75_)	9.1	2019	
0.24	9.7	200	4 (LT_75_)	1.4 (LT_75_)	-	2019	
0.24	12.1	-	0.8 (LT_95_)	-	-	2019	[101]
0.24	17.1	-	1.3 (LT_95_)	-	-	2019	
0.24	16.1	-	13.8 (LT_95_)	-	-	2019	
0.25	16.9	20	46 (LT_70_)	4 (LT_70_)	14.5	2019	[102]
0.25	16.9	1000	602 (LT_70_)	602 (LT_70_)	-	2019	
0.28	11.6	1000	553 (LT_50_)	553 (LT_50_)	20.7	2020	[103]
0.28	17.6	20	43 (LT_70_)	3.7 (LT_70_)	-	2019	[102]
0.28	17.6	1000	709 (LT_70_)	709 (LT_70_)	-	2019	
0.28	14.7	-	2.1 (LT_95_)	-	-	2019	[101]
0.29	11	200	17,500 (LT_50_)	6410 (LT_50_)	21	2006	[97]
0.29	18	20	39.5 (LT_70_)	3.4 (LT_70_)	-	2019	[102]
0.29	18	1000	761 (LT_70_)	761 (LT_70_)	-	2019	
0.32	25.6	1000	20 (LT_70_)	20 (LT_70_)	52.1	2020	[98]
0.38	14	200	100,000 (LT_50_)	36,630 (LT_50_)	32	2006	[97]
0.38	12.6	20	37.8 (LT_70_)	3.2 (LT_70_)	-	2019	[102]
0.38	12.6	1000	466 (LT_70_)	466 (LT_70_)	-	2019	

## Supplementary Materials

The following supporting information can be downloaded at: https://www.mdpi.com/article/10.3390/nano13182521/s1.

## References

[1] Chaskar A., Chen H.-F., Wong K.-T., Chaskar A., Chen H.-F.K., Wong K.-T. (2011). Bipolar Host Materials: A Chemical Approach for Highly Efficient Electrophosphorescent Devices. Adv. Mater..

[2] Han C., Zhao Y., Xu H., Chen J., Deng Z., Ma D., Li Q., Yan P. (2011). A Simple Phosphine-Oxide Host with a Multi-Insulating Structure: High Triplet Energy Level for Efficient Blue Electrophosphorescence. Chem.-A Eur. J..

[3] Chien C.H., Chen C.K., Hsu F.M., Shu C.F., Chou P.T., Lai C.H. (2009). Multifunctional Deep-Blue Emitter Comprising an Anthracene Core and Terminal Triphenylphosphine Oxide Groups. Adv. Funct. Mater..

[4] Tao Y., Wang Q., Yang C., Zhong C., Qin J., Ma D. (2010). Multifunctional Triphenylamine/Oxadiazole Hybrid as Host and Exciton-Blocking Material: High Efficiency Green Phosphorescent OLEDs Using Easily Available and Common Materials. Adv. Funct. Mater..

[5] Swayamprabha S.S., Dubey D.K., Shahnawaz, Yadav R.A.K., Nagar M.R., Sharma A., Tung F.-C., Jou J.-H. (2021). Approaches for Long Lifetime Organic Light Emitting Diodes. Adv. Sci..

[6] Girase J.D., Tagare J., Shahnawaz, Nagar M.R., Siddiqui I., Jou J.H., Patel S., Vaidyanathan S. (2021). Deep-Blue Emitters (CIEy~0.07) Based on Phenanthroimidazole: Remarkable Substitution Effects at the N1 Position of Imidazole on the Excited States and Electroluminescence Properties. Dye. Pigment..

[7] Devesing Girase J., Rani Nayak S., Tagare J., Shahnawaz, Ram Nagar M., Jou J.H., Vaidyanathan S. (2022). Solution-Processed Deep-Blue (Y~0.06) Fluorophores Based on Triphenylamine-Imidazole (Donor-Acceptor) for OLEDs: Computational and Experimental Exploration. J. Inf. Disp..

[8] Anupriya, Thomas K.R.J., Nagar M.R., Shahnawaz, Jou J.H. (2022). Phenanthroimidazole Substituted Imidazo[1,2-a]Pyridine Derivatives for Deep-Blue Electroluminescence with CIEy~0.08. J. Photochem. Photobiol. A Chem..

[9] Jou J.-H. (2015). Introduction to OLED.

[10] Shahnawaz, Siddiqui I., Nagar M.R., Choudhury A., Lin J.T., Blazevicius D., Krucaite G., Grigalevicius S., Jou J.H. (2021). Highly Efficient Candlelight Organic Light-Emitting Diode with a Very Low Color Temperature. Molecules.

[11] Korshunov V.M., Chmovzh T.N., Knyazeva E.A., Taydakov I.V., Mikhalchenko L.V., Varaksina E.A., Saifutyarov R.S., Avetissov I.C., Rakitin O.A. (2019). A Novel Candle Light-Style OLED with a Record Low Colour Temperature. Chem. Commun..

[12] Jou J.-H., Kumar S., An C.-C., Singh M., Yu H.-H., Hsieh C.-Y., Lin Y.-X., Sung C.-F., Wang C.-W. (2015). Enabling a Blue-Hazard Free General Lighting Based on Candle Light-Style OLED. Opt. Express.

[13] Jou J.H., Singh M., Su Y.T., Liu S.H., He Z.K. (2017). Blue-Hazard-Free Candlelight OLED. J. Vis. Exp..

[14] Jou J.H., Hsieh C.Y., Tseng J.R., Peng S.H., Jou Y.C., Hong J.H., Shen S.M., Tang M.C., Chen P.C., Lin C.H. (2013). Candle Light-Style Organic Light-Emitting Diodes. Adv. Funct. Mater..

[15] Quarterly Display Capex and Equipment Market Share Report—Display Supply Chain Consultants. https://www.displaysupplychain.com/report/quarterly-display-capex-and-equipment-market-share-report.

[16] Worldwide-LED Lighting Market Size 2021|Statista. https://www.statista.com/statistics/753939/global-led-luminaire-market-size/.

[17] ElectroniCast Sees a Fast Growing OLED Lighting Market Starting in 2015|OLED-Info. https://www.oled-info.com/electronicast-sees-fast-growing-oled-lighting-market-starting-2015.

[18] Steven Van Slyke Invented OLED Technology, Increasing Efficiency. https://www.invent.org/inductees/steven-van-slyke.

[19] OLED Displays and Their Applications|Learning Corner for Beginners. https://www.electronicsforu.com/resources/oled-displays-applications.

[20] Sony OLED|OLED-Info. https://www.oled-info.com/sony-oled.

[21] AUO. https://www.auo.com/en-global/New_Archive/detail/News_Archive_Technology_190823.

[22] BOE Demonstrates a 55″ 8K Ink-Jet Printed OLED TV Prototype|OLED-Info. https://www.oled-info.com/boe-demonstrates-55-8k-ink-jet-printed-oled-tv-prototype.

[23] LG OLED TV 2016 Display Technology Shoot-Out. https://www.displaymate.com/OLED_TV2016_ShootOut_1.htm.

[24] Kosai S., Badin A.B., Qiu Y., Matsubae K., Suh S., Yamasue E. (2021). Evaluation of Resource Use in the Household Lighting Sector in Malaysia Considering Land Disturbances through Mining Activities. Resour. Conserv. Recycl..

[25] Jou J., Su Y., Liu S., He Z., Sahoo S., Yu H.-H., Chen S.-Z., Wang C.-W., Lee J.-R. (2016). Wet-Process Feasible Candlelight OLED. J. Mater. Chem. C.

[26] Jou J., Hsieh C., Chen P., Kumar S., Hong J.H. (2014). Candlelight Style Organic Light-Emitting Diode: A Plausibly Human-Friendly Safe Night Light. J. Photonics Energy.

[27] Sasabe H., Kido J. (2013). Development of High Performance OLEDs for General Lighting. J. Mater. Chem. C Mater..

[28] Reineke S., Lindner F., Schwartz G., Seidler N., Walzer K., Lüssem B., Leo K. (2009). White Organic Light-Emitting Diodes with Fluorescent Tube Efficiency. Nature.

[29] Panasonic Developed a 114 Lm/W OLED Panel-Claims World’s Most Efficient Panel|OLED-Info. https://www.oled-info.com/panasonic-developed-114-lmw-oled-panel-claims-worlds-most-efficient-panel.

[30] Panasonic Develops World’s Highest Efficiency White OLED for Lighting|Headquarters News|Panasonic Newsroom Global. https://news.panasonic.com/global/press/data/2013/05/en130524-6/en130524-6.html.

[31] Kato K., Iwasaki T., Tsujimura T. (2015). Over 130 Lm/W All-Phosphorescent White OLEDs for next-Generation Lighting. J. Photopolym. Sci. Technol..

[32] Philips-GL350-Oled-Product-Sheet-Philips Lighting. https://www.yumpu.com/en/document/view/3069680/philips-gl350-oled-product-sheet-philips-lighting.

[33] OSRAM Presents the First OLED Luminaire—OSRAM Group Website. https://www.osram-group.com/en/media/press-releases/pr-2010/05-11-2010.

[34] LG Chem’s 320x320 Mm OLED Lighting Panel Now in Production, Company Develops New Integration Solutions|OLED-Info. https://www.oled-info.com/lg-chems-320x320-mm-oled-lighting-panel-now-production-company-develops-new-integration-solutions.

[35] OLEDWorks Closes Philips Lumiblade Deal and Announces New OLED Panels|LEDs Magazine. https://www.ledsmagazine.com/leds-ssl-design/oleds/article/16696751/oledworks-closes-philips-lumiblade-deal-and-announces-new-oled-panels.

[36] Lumiotec’s New P09 OLED Lighting Panels Feature 45 Lm/W, 40,000 Hours Lifetime|OLED-Info. https://www.oled-info.com/lumiotecs-new-p09-oled-lighting-panels-feature-45-lmw-40000-hours-lifetime.

[37] OLED Lighting Introduction and Market Status|OLED-Info. https://www.oled-info.com/oled-lighting.

[38] Products|OLED Lighting|KONICA MINOLTA. https://www.konicaminolta.com/oled/products/.

[39] Mitsubishi and Pioneer to Start Mass Producing Color-Tunable OLEDs Made Using a Wet-Coating Process|OLED-Info. https://www.oled-info.com/mitsubishi-and-pioneer-start-mass-producing-color-tunable-oleds-made-using-wet-coating-process.

[40] Kaneka Demonstrate Their New 50,000 Hours OLED Lighting Panels|OLED-Info. https://www.oled-info.com/kaneka-demonstrate-their-new-50000-hours-oled-lighting-panels.

[41] Introduction to OLED Lighting (Acuity Brands)-YouTube. https://www.youtube.com/watch?v=3nVupTrnw4s.

[42] CYNORA Announces Availability of Industry’s First Device Test Kits for TADF Deep Green Emitters for Next-Generation OLED Displays-CynoraCynora. https://cynora.com/cynora-announces-availability-of-industrys-first-device-test-kits-for-tadf-deep-green-emitters-for-next-generation-oled-displays-2/.

[43] Nakamura S. (2015). Shuji Nakamura-Nobel Lecture: Background Story of the Invention of Efficient Blue InGaN Light Emitting Diodes. Rev. Mod. Phys..

[44] Blue LEDs-Filling the World with New Light. https://www.nobelprize.org/uploads/2018/06/popular-physicsprize2014-1.pdf.

[45] (2014). Nobel Prize ® and the Nobel Prize ® Medal Design Mark Are Registrated Trademarks of the Nobel Foundation. https://www.theguardian.com/science/2014/oct/07/nobel-prize-physics-inventors-blue-light-emitting-diodes-isamu-akasaki-hiroshi-amano-shuji-nakam#:~:text=Isamu%20Akasaki%20and%20Hiroshi%20Amano,and%20environment%2Dfriendly%20light%20source.

[46] Introduction to Color Imaging Science-Hsien-Che Lee-Google Books. https://books.google.com.tw/books/about/Introduction_to_Color_Imaging_Science.html?id=rN3kOMOPMmEC&redir_esc=y.

[47] Subtractive Color–HiSoUR–Hi So You Are. https://www.hisour.com/subtractive-color-24755/.

[48] Additive and Subtractive Color Mixing. https://isle.hanover.edu/Ch06Color/Ch06ColorMixer.html.

[49] Taming the Wide Gamut Using SRGB Emulation|PC Monitors. https://pcmonitors.info/articles/taming-the-wide-gamut-using-srgb-emulation/.

[50] Understanding the Color Gamut of an LCD Monitor. https://www.eizo.com/library/basics/lcd_monitor_color_gamut/.

[51] Jou J.H., Kumar S., Fang P.H., Venkateswararao A., Thomas K.R.J., Shyue J.J., Wang Y.C., Li T.H., Yu H.H. (2015). Highly Efficient Ultra-Deep Blue Organic Light-Emitting Diodes with a Wet- and Dry-Process Feasible Cyanofluorene Acetylene Based Emitter. J. Mater. Chem. C Mater..

[52] Kim H.K., Cho S.H., Oh J.R., Lee Y.H., Lee J.H., Lee J.G., Kim S.K., Park Y.I., Park J.W., Do Y.R. (2010). Deep Blue, Efficient, Moderate Microcavity Organic Light-Emitting Diodes. Org. Electron..

[53] Li W., Yao L., Liu H., Wang Z., Zhang S., Xiao R., Zhang H., Lu P., Yang B., Ma Y. (2014). Highly Efficient Deep-Blue OLED with an Extraordinarily Narrow FHWM of 35 nm and a y Coordinate <0.05 Based on a Fully Twisting Donor-Acceptor Molecule. J. Mater. Chem. C Mater..

[54] Reineke S., Schwartz G., Walzer K., Leo K. (2007). Reduced Efficiency Roll-off in Phosphorescent Organic Light Emitting Diodes by Suppression of Triplet-Triplet Annihilation. Appl. Phys. Lett..

[55] Yang H., Shi Y., Zhao Y., Meng Y., Hu W., Hou J., Liu S. (2008). High Colour Rendering Index White Organic Light-Emitting Devices with Three Emitting Layers. Displays.

[56] Jou J.H., Chou Y.C., Shen S.M., Wu M.H., Wu P.S., Lin C.R., Wu R.Z., Chen S.H., Wei M.K., Wang C.W. (2011). High-Efficiency, Very-High Color Rendering White Organic Light-Emitting Diode with a High Triplet Interlayer. J. Mater. Chem..

[57] Lumiotec Announces New High CRI OLED Lighting Panels, Targets Museums|OLED-Info. https://www.oled-info.com/lumiotec-announces-new-high-cri-oled-lighting-panels-targets-museums.

[58] LG Chem Produces High-CRI OLED Panels That Achieve 20,000-Hour Lifetimes|LEDs Magazine. https://www.ledsmagazine.com/company-newsfeed/article/16680965/lg-chem-produces-highcri-oled-panels-that-achieve-20000hour-lifetimes.

[59] Adachi C., Tsutsui T., Saito S. (1998). Blue Light-emitting Organic Electroluminescent Devices. Appl. Phys. Lett..

[60] Adachi C., Kwong R.C., Djurovich P., Adamovich V., Baldo M.A., Thompson M.E., Forrest S.R. (2001). Endothermic Energy Transfer: A Mechanism for Generating Very Efficient High-Energy Phosphorescent Emission in Organic Materials. Appl. Phys. Lett..

[61] Kawamura Y., Yanagida S., Forrest S.R. (2002). Energy Transfer in Polymer Electrophosphorescent Light Emitting Devices with Single and Multiple Doped Luminescent Layers. J. Appl. Phys..

[62] Youn Lee S., Yasuda T., Nomura H., Adachi C. (2012). High-Efficiency Organic Light-Emitting Diodes Utilizing Thermally Activated Delayed Fluorescence from Triazine-Based Donor–Acceptor Hybrid Molecules. Appl. Phys. Lett..

[63] Nakanotani H., Masui K., Nishide J., Shibata T., Adachi C. (2013). Promising Operational Stability of High-Efficiency Organic Light-Emitting Diodes Based on Thermally Activated Delayed Fluorescence. Sci. Rep..

[64] Masui K., Nakanotani H., Adachi C. (2013). Analysis of Exciton Annihilation in High-Efficiency Sky-Blue Organic Light-Emitting Diodes with Thermally Activated Delayed Fluorescence. Org. Electron..

[65] Lee J., Shizu K., Tanaka H., Nomura H., Yasuda T., Adachi C. (2013). Oxadiazole- and Triazole-Based Highly-Efficient Thermally Activated Delayed Fluorescence Emitters for Organic Light-Emitting Diodes. J. Mater. Chem. C Mater..

[66] Mallesham G., Swetha C., Niveditha S., Mohanty M.E., Babu N.J., Kumar A., Bhanuprakash K., Rao V.J. (2015). Phosphine Oxide Functionalized Pyrenes as Efficient Blue Light Emitting Multifunctional Materials for Organic Light Emitting Diodes. J. Mater. Chem. C Mater..

[67] Liu W., Zheng C.J., Wang K., Chen Z., Chen D.Y., Li F., Ou X.M., Dong Y.P., Zhang X.H. (2015). Novel Carbazol-Pyridine-Carbonitrile Derivative as Excellent Blue Thermally Activated Delayed Fluorescence Emitter for Highly Efficient Organic Light-Emitting Devices. ACS Appl. Mater. Interfaces.

[68] Hiraga Y., Nishide J.I., Nakanotani H., Aonuma M., Adachi C. (2015). High-Efficiency Sky-Blue Organic Light-Emitting Diodes Utilizing Thermally-Activated Delayed Fluorescence. IEICE Trans. Electron..

[69] Organic Electroluminescence Device. https://www.freepatentsonline.com/5389444.pdf.

[70] Specification E.P. (2002). EP000875947B1. https://data.epo.org/publication-server/document?iDocId=2079949&iFormat=0.

[71] WO2001039234 Organic Light Emitting Diode Having a Blue Phosphorescent Molecule as an Emitter. https://patentscope.wipo.int/search/en/detail.jsf?docId=WO2001039234.

[72] Takita Y. (2018). Highly Efficient Deep-Blue Fluorescent Dopant for Achieving Low-Power OLED Display Satisfying BT. 2020 Chromaticity. J. Soc. Inf. Disp..

[73] Liu H., Kang L., Li J., Liu F., He X., Ren S., Tang X., Lv C., Lu P. (2019). Highly Efficient Deep-Blue Organic Light-Emitting Diodes Based on Pyreno[4,5-d]Imidazole-Anthracene Structural Isomers. J. Mater. Chem. C.

[74] Zou Y., Zou J., Ye T., Li H., Yang C., Wu H., Ma D., Qin J., Cao Y. (2013). Unexpected Propeller-Like Hexakis(Fluoren-2-Yl)Benzene Cores for Six-Arm Star-Shaped Oligofluorenes: Highly Efficient Deep-Blue Fluorescent Emitters and Good Hole-Transporting Materials. Adv. Funct. Mater..

[75] Wang L., Jiang Y., Luo J., Zhou Y., Zhou J., Wang J., Pei J., Cao Y. (2009). Highly Efficient and Color-Stable Deep-Blue Organic Light-Emitting Diodes Based on a Solution-Processible Dendrimer. Adv. Mater..

[76] Li W., Zhang X., Zhang Y., Xu K., Xu J., Wang H., Li H., Guo J., Mo J., Yang P. (2018). Achieving Ultra-High Efficiency by Tuning Hole Transport and Carrier Balance in Fluorescent Blue Organic Light-Emitting Diode with Extremely Simple Structure. Synth. Met..

[77] Zhen C.G., Dai Y.F., Zeng W.J., Ma Z., Chen Z.K., Kieffer J. (2011). Achieving Highly Efficient Fluorescent Blue Organic Light-Emitting Diodes through Optimizing Molecular Structures and Device Configuration. Adv. Funct. Mater..

[78] Liu M., Li X.-L., Cheng Chen D., Xie Z., Cai X., Xie G., Liu K., Tang J., Su S.-J., Cao Y. (2015). Study of Configuration Differentia and Highly Efficient, Deep-Blue, Organic Light-Emitting Diodes Based on Novel Naphtho[1,2-d]Imidazole Derivatives. Adv. Funct. Mater..

[79] Li Z., Gan G., Ling Z., Guo K., Si C., Lv X., Wang H., Wei B., Hao Y. (2019). Easily Available, Low-Cost 9,9′-Bianthracene Derivatives as e Ffi Cient Blue Hosts and Deep-Blue Emitters in OLEDs. Org. Electron..

[80] Lee J.H., Chen C.H., Lee P.H., Lin H.Y., Leung M.K., Chiu T.L., Lin C.F. (2019). Blue Organic Light-Emitting Diodes: Current Status, Challenges, and Future Outlook. J. Mater. Chem. C Mater..

[81] Jeong S., Kim M., Kim S.H., Hong J. (2013). Efficient Deep-Blue Emitters Based on Triphenylamine-Linked Benzimidazole Derivatives for Nondoped Fluorescent Organic Light-Emitting Diodes. Org. Electron..

[82] Jeon Y., Lee J., Kim J., Lee C., Gong M. (2010). Deep-Blue OLEDs Using Novel Efficient Spiro-Type Dopant Materials. Org. Electron..

[83] Wei B., Liu J., Zhang Y., Zhang J., Peng H., Fan H., He Y., Gao X. (2010). Stable, Glassy, and Versatile Binaphthalene Derivatives Capable of Efficient Hole Transport, Hosting, and Deep-Blue Light Emission. Adv. Funct. Mater..

[84] Wen S., Lee M., Chen C.H. (2005). Recent Development of Blue Fluorescent OLED Materials and Devices. J. Disp. Technol..

[85] Patil V.V., Lee K.H., Lee J.Y. (2020). For Blue Fluorescent Organic Light-Emitting Diodes. J. Mater. Chem. C.

[86] Park H.-Y., Maheshwaran A., Moon C.-K., Lee H., Sudhaker Reddy S., Gopalan Sree V., Yoon J., Won Kim J., Hyuk Kwon J., Kim J.-J. (2020). External Quantum Efficiency Exceeding 24% with CIEy Value of 0.08 Using a Novel Carbene-Based Iridium Complex in Deep-Blue Phosphorescent Organic Light-Emitting Diodes. Adv. Mater..

[87] Sarma M., Tsai W.L., Lee W.K., Chi Y., Wu C.C., Liu S.H., Chou P.T., Wong K.T. (2017). Anomalously Long-Lasting Blue PhOLED Featuring Phenyl-Pyrimidine Cyclometalated Iridium Emitter. Chem.

[88] Li W., Li J., Liu D., Jin Q. (2016). Simple Bipolar Host Materials for High-E Ffi Ciency Blue, Green, and White Phosphorescence OLEDs. ACS Appl. Mater. Interfaces.

[89] Kim M., Lee I.H., Lee J., Hyun S., Park K., Kang Y. (2018). Chemistry Phenanthridine Based Ir(III) Complex for Blue Phosphorescent Organic Light-Emitting Diodes with Long-Term Operational Stability. J. Ind. Eng. Chem..

[90] Oh C.S., Lee Y., Noh H., Han S. (2016). Molecular Design of Host Materials for High Power Efficiency in Blue Phosphorescent Organic Light-Emitting Diodes Doped with an Imidazole Ligand Based Triplet Emitter. J. Mater. Chem. C.

[91] Ying S., Pang P., Zhang S., Sun Q., Dai Y., Qiao X., Yang D., Chen J., Ma D. (2019). Superior Efficiency and Low-Efficiency Roll-Off White Organic Light-Emitting Diodes Based on Multiple Exciplexes as Hosts Matched to Phosphor Emitters. ACS Appl. Mater. Interfaces.

[92] Son Y.H., Kim Y.J., Park M.J., Oh H.Y., Park J.S., Yang J.H., Suh M.C., Kwon J.H. (2013). Small Single–Triplet Energy Gap Bipolar Host Materials for Phosphorescent Blue and White Organic Light Emitting Diodes. J. Mater. Chem. C Mater..

[93] Feng Y., Zhuang X., Zhu D., Liu Y., Bryce M.R. (2016). Heteroleptic Phosphorescent Iridium(III). J. Mater. Chem. C.

[94] Diode L., Jou J., Wang W., Hsu M., Liu C., Chen C. (2008). Small Nano-Dot Incorporated High-Efficiency Phosphorescent Blue Organic Small Nano-Dot Incorporated High-Efficiency Phosphorescent Blue Organic Light-Emitting Diode. PIERS.

[95] Wu Z.G., Jing Y.M., Lu G.Z., Zhou J., Zheng Y.X., Zhou L., Wang Y., Pan Y. (2016). Novel Design of Iridium Phosphors with Pyridinylphosphinate Ligands for High-Efficiency Blue Organic Light-Emitting Diodes. Sci. Rep..

[96] Wee K.R., Kim A.L., Jeong S.Y., Kwon S., Kang S.O. (2011). The Relationship between the Device Performance and Hole Mobility of Host Materials in Mixed Host System for Deep Blue Phosphorescent Organic Light Emitting Devices. Org. Electron..

[97] Weaver M.S., Tung Y.-J., D’Andrade B., Esler J., Brown J.J., Lin C., Mackenzie P.B., Walters R.W., Tsai J.-Y., Brown C.S. (2006). 11.1: Invited Paper: Advances in Blue Phosphorescent Organic Light-Emitting Devices. SID Symp. Dig. Tech. Pap..

[98] Kumar Konidena R., Jae Chung W., Yeob Lee J., Konidena R.K., Chung W.J., Lee J.Y. (2020). Molecular Engineering of Cyano-Substituted Carbazole-Based Host Materials for Simultaneous Achievement of High Efficiency and Long Lifetime in Blue Phosphorescent Organic Light-Emitting Diodes. Adv. Electron. Mater..

[99] Jung M., Lee K.H., Lee J.Y., Kim T. (2020). A Bipolar Host Based High Triplet Energy Electroplex for an over 10,000 h Lifetime in Pure Blue Phosphorescent Organic Light-Emitting Diodes. Mater. Horiz..

[100] Yang C.Y., Jang H.J., Lee K.H., Byeon S.Y., Lee J.Y., Jeong H. (2019). 12-1: Analysis of Key Factors Affecting the Lifetime of Blue Phosphorescent OLED Using CN Modified Blue Host Materials. SID Symp. Dig. Tech. Pap..

[101] Han S.H., Lee K.H., Lee J.Y. (2019). Spatial Separation of Two Blue Triplet Emitters for Improved Lifetime in Blue Phosphorescent Organic Light-Emitting Diodes by Confining Excitons at the Interface between Two Emitting Layers. Org. Electron..

[102] Klimes K., Zhu Z.Q., Li J. (2019). Efficient Blue Phosphorescent OLEDs with Improved Stability and Color Purity through Judicious Triplet Exciton Management. Adv. Funct. Mater..

[103] Yao J., Xiao S., Zhang S., Sun Q., Dai Y., Qiao X., Yang D., Chen J., Ma D. (2020). High Efficiency, Low Efficiency Roll-off and Long Lifetime Fluorescent White Organic Light-Emitting Diodes Based on Strategic Management of Triplet Excitons via Triplet–Triplet Annihilation up-Conversion and Phosphor Sensitization. J. Mater. Chem. C Mater..

[104] Liang X., Han H.B., Yan Z.P., Liu L., Zheng Y.X., Meng H., Huang W. (2018). Versatile Functionalization of Trifluoromethyl Based Deep Blue Thermally Activated Delayed Fluorescence Materials for Organic Light Emitting Diodes. New J. Chem..

[105] Lim H., Cheon J., Woo S.-J., Kwon S.-K., Kim Y.-H., Kim J.-J., Lim H., Woo S.-J., Kim J.-J., Cheon H.J. (2020). Highly Efficient Deep-Blue OLEDs Using a TADF Emitter with a Narrow Emission Spectrum and High Horizontal Emitting Dipole Ratio. Adv. Mater..

[106] Li W., Li B., Cai X., Gan L., Xu Z., Li W., Liu K., Chen D., Su S.J. (2019). Tri-Spiral Donor for High Efficiency and Versatile Blue Thermally Activated Delayed Fluorescence Materials. Angew. Chem. Int. Ed..

[107] Miwa T., Kubo S., Shizu K., Komino T., Adachi C., Kaji H. (2017). Blue Organic Light-Emitting Diodes Realizing External Quantum Efficiency over 25% Using Thermally Activated Delayed Fluorescence Emitters. Sci. Rep..

[108] Kim H.J., Godumala M., Kim S.K., Yoon J., Kim C.Y., Park H., Kwon J.H., Cho M.J., Choi D.H. (2020). Color-Tunable Boron-Based Emitters Exhibiting Aggregation-Induced Emission and Thermally Activated Delayed Fluorescence for Efficient Solution-Processable Nondoped Deep-Blue to Sky-Blue OLEDs. Adv. Opt. Mater..

[109] Xie F.M., An Z.D., Xie M., Li Y.Q., Zhang G.H., Zou S.J., Chen L., Chen J.D., Cheng T., Tang J.X. (2020). Tert-Butyl Substituted Hetero-Donor TADF Compounds for Efficient Solution-Processed Non-Doped Blue OLEDs. J. Mater. Chem. C Mater..

[110] Ganesan P., Chen D.G., Liao J.L., Li W.C., Lai Y.N., Luo D., Chang C.H., Ko C.L., Hung W.Y., Liu S.W. (2018). Isomeric Spiro-[Acridine-9,9′-Fluorene]-2,6-Dipyridylpyrimidine Based TADF Emitters: Insights into Photophysical Behaviors and OLED Performances. J. Mater. Chem. C Mater..

[111] Oh C.S., Pereira D.D.S., Han S.H., Park H.J., Higginbotham H.F., Monkman A.P., Lee J.Y. (2018). Dihedral Angle Control of Blue Thermally Activated Delayed Fluorescent Emitters through Donor Substitution Position for Efficient Reverse Intersystem Crossing. ACS Appl. Mater. Interfaces.

[112] Ban X., Chen F., Pan J., Liu Y., Zhu A., Jiang W., Sun Y. (2020). Exciplex Formation and Electromer Blocking for Highly Efficient Blue Thermally Activated Delayed Fluorescence OLEDs with All-Solution-Processed Organic Layers. Chem.-A Eur. J..

[113] Yoon J., Kim S.K., Kim H.J., Choi S., Jung S.W., Lee H., Kim J.Y., Yoon D.W., Han C.W., Chae W.S. (2020). Asymmetric Host Molecule Bearing Pyridine Core for Highly Efficient Blue Thermally Activated Delayed Fluorescence OLEDs. Chem.-A Eur. J..

[114] Su L., Cao F., Cheng C., Tsuboi T., Zhu Y., Deng C., Zheng X., Wang D., Liu Z., Zhang Q. (2020). High Fluorescence Rate of Thermally Activated Delayed Fluorescence Emitters for Efficient and Stable Blue OLEDs. ACS Appl. Mater. Interfaces.

[115] Ihn S.G., Lee N., Jeon S.O., Sim M., Kang H., Jung Y., Huh D.H., Son Y.M., Lee S.Y., Numata M. (2017). An Alternative Host Material for Long-Lifespan Blue Organic Light-Emitting Diodes Using Thermally Activated Delayed Fluorescence. Adv. Sci..

[116] Feng X., Hu J.-Y., Yi L., Seto N., Tao Z., Redshaw C., Elsegood M.R.J., Yamato T. (2012). Pyrene-Based Y-shaped Solid-State Blue Emitters: Synthesis, Characterization, and Photoluminescence. Chem. Asian J..

[117] Yang C.Y., Lee K.H., Lee J.Y. (2020). Zig-Zag Type Molecular Design Strategy of N-Type Hosts for Sky-Blue Thermally-Activated Delayed Fluorescence Organic Light-Emitting Diodes. Chem.-A Eur. J..

[118] Hamada Y., Adachi C., Tsutsui T., Saito S. (1992). Organic Electroluminescent Devices with Bright Blue Emission. Optoelectron. Tokyo.

[119] Hamada Y., Adachi C., Tsutsui T., Saito S. (1992). Blue-Light-Emitting Organic Electroluminescent Devices with Oxadiazole Dimer Dyes as an Emitter. Jpn. J. Appl. Phys..

[120] Grem G., Leditzky G., Ullrich B., Leising G. (1992). Realization of a Blue-Light-Emitting Device Using Poly(p-Phenylene). Adv. Mater..

[121] Grem G., Leditzky G., Ullrich B., Leising G. (1992). Blue Electroluminescent Device Based on a Conjugated Polymer. Synth. Met..

[122] Hosokawa C., Kawasaki N., Sakamoto S., Kusumoto T. (1998). Bright Blue Electroluminescence from Hole Transporting Polycarbonate. Appl. Phys. Lett..

[123] Hosokawa C., Tokailin H., Higashi H., Kusumoto T. (1998). Transient Behavior of Organic Thin Film Electroluminescence. Appl. Phys. Lett..

[124] Hosokawa C., Tokailin H., Higashi H., Kusumoto T. (1998). Transient Electroluminescence from Hole Transporting Emitting Layer in Nanosecond Region. Appl. Phys. Lett..

[125] Vestweber H., Sander R., Greiner A., Heitz W., Mahrt R.F., Bässler H. (1994). Electroluminescence from Polymer Blends and Molecularly Doped Polymers. Synth. Met..

[126] Hosokawa C., Tokailin H., Higashi H., Kusumoto T. (1998). Efficient Electroluminescence of Distyrylarylene with Hole Transporting Ability. J. Appl. Phys..

[127] Tokailin T.H., Matsuura M., Higashi H., Hosokawa C., Kusumoto T., Tokailin H., Matsuura M., Kusumoto T. (1993). Characteristics of Blue Organic Electroluminescent Devices with Distyryl Arylene Derivatives. Electron. Imaging.

[128] Hosokawa C., Matsuura M., Eida M., Fukuoka K., Tokailin H., Kusumoto T. (1998). 4.1: Invited Paper: Full-Color Organic EL Display. SID Symp. Dig. Tech. Pap..

[129] Ho M.H., Chang C.M., Chu T.Y., Chen T.M., Chen C.H. (2008). Iminodibenzyl-Substituted Distyrylarylenes as Dopants for Blue and White Organic Light-Emitting Devices. Org. Electron..

[130] Wind M., Wiesler U.M., Saalwächter K., Müllen K., Spiess H.W. (2001). Shape-Persistent Polyphenylene Dendrimers—Restricted Molecular Dynamics from Advanced Solid-State Nuclear Magnetic Resonance Techniques. Adv. Mater..

[131] Rosenfeldt S., Dingenouts N., Pötschke D., Ballauff M., Berresheim A.J., Müllen K., Lindner P. (2004). Analysis of the Spatial Dimensions of Fully Aromatic Dendrimers. Angew. Chem. Int. Ed..

[132] Novel Green Light-Emitting Carbazole Derivatives: Potential Electroluminescent Materials-Thomas-2000-Advanced Materials-Wiley Online Library. https://onlinelibrary.wiley.com/doi/pdf/10.1002/1521-4095%28200012%2912%3A24%3C1949%3A%3AAID-ADMA1949%3E3.0.CO%3B2-X.

[133] Tonzola C.J., Alam M.M., Kaminsky W., Jenekhe S.A. (2003). New N-Type Organic Semiconductors: Synthesis, Single Crystal Structures, Cyclic Voltammetry, Photophysics, Electron Transport, and Electroluminescence of a Series of Diphenylanthrazolines. J. Am. Chem. Soc..

[134] Figueira-Duarte T.M., Del Rosso P.G., Trattnig R., Sax S., List E.J.W., Mullen K. (2010). Designed Suppression of Aggregation in Polypyrene: Toward High-Performance Blue-Light-Emitting Diodes. Adv. Mater..

[135] Xing Y., Xu X., Zhang P., Tian W., Yu G., Lu P., Liu Y., Zhu D. (2005). Carbazole–Pyrene-Based Organic Emitters for Electroluminescent Device. Chem. Phys. Lett..

[136] Sotoyama W., Sato H., Kinoshita M., Takahashi T., Matsuura A., Kodama J., Sawatari N., Inoue H. (2003). 45.3: Tetra-Substituted Pyrenes: New Class of Blue Emitter for Organic Light-Emitting Diodes. SID Symp. Dig. Tech. Pap..

[137] Miura Y., Yamano E., Tanaka A., Yamauchi J. (1994). Generation, Isolation, and Characterization of N-(Arylthio)-7-Tert-Butyl- and N-(Arylthio)-2,7-Di-Tert-Butyl-1-Pyrenylaminyl Radicals. J. Org. Chem..

[138] Grill E., Hauge B., Schiefelbein J., Martinez-Zapater J., Gil P., Iba K., Bevilacqua P.C., Kierzek R., Johnson K.A., Turner D.H. (1992). Dynamics of Ribozyme Binding of Substrate Revealed by Fluorescence-Detected Stopped-Flow Methods. Science.

[139] Kim J., Beardslee R., Phillips D.T., Offen H.W. (1969). Fluorescence Lifetimes of Pyrene Monomer and Excimer at High Pressures. J. Chem. Phys..

[140] Kropp J.L., Dawson W.R., Windsor M.W. (1969). Radiative and Radiationless Processes in Aromatic Molecules. Pyrene. J. Phys. Chem..

[141] Zhao Z., Li J.H., Chen X., Wang X., Lu P., Yang Y. (2009). Solution-Processable Stiff Dendrimers: Synthesis, Photophysics, Film Morphology, and Electroluminescence. J. Org. Chem..

[142] Jia W.-L., McCormick T., Liu Q.-D., Fukutani H., Motala M., Wang R.-Y., Tao Y., Wang S. (2004). Diarylamino Functionalized Pyrene Derivatives for Use in Blue OLEDs and Complex Formation. J. Mater. Chem..

[143] Lampkins A.J., O’Neil E.J., Smith B.D. (2008). Bio-Orthogonal Phosphatidylserine Conjugates for Delivery and Imaging Applications. J. Org. Chem..

[144] Vollmer A., Lorbach D., List E.J.W., Müllen K., Loi M.A., Manca M., Baumgarten M., Koch N., Trattnig R., Sax S. (2011). Deep Blue Polymer Light Emitting Diodes Based on Easy to Synthesize, Non-Aggregating Polypyrene. Opt. Express.

[145] Wu K.C., Ku P.J., Lin C.S., Shih H.T., Wu F.I., Huang M.J., Lin J.J., Chen I.C., Cheng C.H. (2008). The Photophysical Properties of Dipyrenylbenzenes and Their Application as Exceedingly Efficient Blue Emitters for Electroluminescent Devices. Adv. Funct. Mater..

[146] Zhao Z., Xu X., Wang H., Lu P., Yu G., Liu Y. (2008). Zigzag Molecules from Pyrene-Modified Carbazole Oligomers: Synthesis, Characterization, and Application in OLEDs. J. Org. Chem..

[147] Zhao Z., Xu X., Jiang Z., Lu P., Yu G., Liu Y. (2007). Oligo(2,7-Fluorene Ethynylene)s with Pyrene Moieties: Synthesis, Characterization, Photoluminescence, and Electroluminescence. J. Org. Chem..

[148] Chan K., Lim J., Yang X., Dodabalapur A., Jabbour G.E., Sellinger A. (2012). High-Efficiency Pyrene-Based Blue Light Emitting Diodes: Aggregation Suppression Using a Calixarene 3D-Scaffold. Chem. Commun..

[149] Wang Z., Xu C., Wang W., Dong X., Zhao B., Pigments B.J.-D. (2012). Novel Pyrene Derivatives: Synthesis, Properties and Highly Efficient Non-Doped Deep-Blue Electroluminescent Device. Dye. Pigment..

[150] Sonar P., Soh M.S., Cheng Y.H., Henssler J.T., Sellinger A. (2010). 1,3,6,8-Tetrasubstituted Pyrenes: Solution-Processable Materials for Application in Organic Electronics. Org. Lett..

[151] Moorthy J.N., Natarajan P., Venkatakrishnan P., Huang D.F., Chow T.J. (2007). Steric Inhibition of π-Stacking: 1,3,6,8-Tetraarylpyrenes as Efficient Blue Emitters in Organic Light Emitting Diodes (OLEDs). Org. Lett..

[152] Otsubo T., Aso Y., Takimiya K. (2002). Functional Oligothiophenes as Advanced Molecular Electronic Materials. J. Mater. Chem..

[153] Kumchoo T., Promarak V., Sudyoadsuk T., Sukwattanasinitt M., Rashatasakhon P. (2010). Dipyrenylcarbazole Derivatives for Blue Organic Light-Emitting Diodes. Chem. Asian J..

[154] Zhao Z., Xu X., Wang F., Yu G., Lu P., Liu Y., Zhu D. (2006). Synthesis and Characterization of Light-Emitting Materials Composed of Carbazole, Pyrene and Fluorene. Synth. Met..

[155] Mikroyannidis J.A., Fenenko L., Adachi C. (2006). Synthesis and Photophysical Characteristics of 2,7-Fluorenevinylene-Based Trimers and Their Electroluminescence. J. Phys. Chem. B.

[156] Tang C., Liu F., Xia Y., Xie L., Wei A., Li S.-B., Fan Q.-L., Huang W. (2006). Efficient 9-Alkylphenyl-9-Pyrenylfluorene Substituted Pyrene Derivatives with Improved Hole Injection for Blue Light-Emitting Diodes. J. Mater. Chem..

[157] Tang C., Liu F., Xia Y., Lin J., Xie L., Zhong G.-Y., Fan Q.-L., Huang W. (2006). Fluorene-Substituted Pyrenes—Novel Pyrene Derivatives as Emitters in Nondoped Blue OLEDs. Org. Electron..

[158] Peng Z., Tao S., Zhang X., Tang J., Lee C.S., Lee S.T. (2008). New Fluorene Derivatives for Blue Electroluminescent Devices: Influence of Substituents on Thermal Properties, Photoluminescence, and Electroluminescence. J. Phys. Chem. C.

[159] Liu F., Xie L.H., Tang C., Liang J., Chen Q.Q., Peng B., Wei W., Cao Y., Huang W. (2009). Facile Synthesis of Spirocyclic Aromatic Hydrocarbon Derivatives Based on O-Halobiaryl Route and Domino Reaction for Deep-Blue Organic Semiconductors. Org. Lett..

[160] Tao S., Zhou Y., Lee C.S., Zhang X., Lee S.T. (2010). High-Efficiency Nondoped Deep-Blue-Emitting Organic Electroluminescent Device. Chem. Mater..

[161] Thangthong A., Prachumrak N., Tarsang R., Keawin T., Jungsuttiwong S., Sudyoadsuka T., Promarak V. (2012). Blue Light-Emitting and Hole-Transporting Materials Based on 9, 9-Bis (4-Diphenylaminophenyl) Fluorenes for Efficient Electroluminescent Devices. Mater. Chem..

[162] Jou J., Chen Y., Tseng J., Wu R.Z., Shyue J.J., Thomas K.R.J., Kapoor N., Chen C.-T., Lin Y.-P., Wang P.-H. (2012). The Use of a Polarity Matching and High-Energy Exciton Generating Host in Fabricating Efficient Purplish-Blue OLEDs from a Sky-Blue Emitter. J. Mater. Chem..

[163] Tao S., Peng Z., Zhang X., Wang P., Lee C.-S., Lee S.-T. (2005). Highly Efficient Non-doped Blue Organic Light-emitting Diodes Based on Fluorene Derivatives with High Thermal Stability. Adv. Funct. Mater..

[164] Lai S., Tong Q., Chan M., Ng T., Lo M.-F., Lee S.-T., Lee C.-S. (2011). Distinct Electroluminescent Properties of Triphenylamine Derivatives in Blue Organic Light-Emitting Devices. J. Mater. Chem..

[165] Thomas K., Velusamy M., Lin J.T., Chuen C.H., Tao Y.-T. (2005). Hexaphenylphenylene Dendronised Pyrenylamines for Efficient Organic Light-Emitting Diodes. J. Mater. Chem..

[166] Tao S., Zhou Y., Lee C.S., Lee S.T., Huang D., Zhang X. (2008). Highly Efficient Nondoped Blue Organic Light-Emitting Diodes Based on Anthracene-Triphenylamine Derivatives. J. Phys. Chem. C.

[167] Figueira-Duarte T.M., Müllen K. (2011). Pyrene-Based Materials for Organic Electronics. Chem. Rev..

[168] Kumar D., Thomas K.R.J., Lin C.C., Jou J.H. (2013). Pyrenoimidazole-Based Deep-Blue-Emitting Materials: Optical, Electrochemical, and Electroluminescent Characteristics. Chem. Asian J..

[169] Figueira-Duarte T.M., Simon S.C., Wagner M., Druzhinin S.I., Zachariasse K.A., Müllen K. (2008). Polypyrene Dendrimers. Angew. Chem. Int. Ed..

[170] Kotchapradist P., Prachumrak N., Tarsang R., Jungsuttiwong S., Keawin T., Sudyoadsuk T., Promarak V. (2013). Pyrene-Functionalized Carbazole Derivatives as Non-Doped Blue Emitters for Highly Efficient Blue Organic Light-Emitting Diodes. J. Mater. Chem. C.

[171] Shi J., Tang C.W. (2002). Anthracene Derivatives for Stable Blue-Emitting Organic Electroluminescence Devices. Appl. Phys. Lett..

[172] Shih B.P., Chuang C., Chien C., Diau E.W., Shu C. (2007). Highly Efficient Non-Doped Blue-Light-Emitting Diodes Based on an Anthrancene Derivative End-Capped with Tetraphenylethylene Groups. Adv. Funct. Mater..

[173] Kim S., Yang B., Ma Y., Lee J., Park J. (2008). Exceedingly Efficient Deep-Blue Electroluminescence from New Anthracenes Obtained Using Rational Molecular Design. J. Mater. Chem..

[174] Zheng C., Zhao W., Wang Z., Huang D., Ye J., Ou X., Zhang X., Lee C., Lee S. (2010). Highly Efficient Non-Doped Deep-Blue Organic Light-Emitting Diodes Based on Anthracene Derivatives. J. Mater. Chem..

[175] Chem J.M., Kim S.H., Cho I., Sim M.K., Park S., Park S.Y. (2011). Highly Efficient Deep-Blue Emitting Organic Light Emitting Diode Based on the Multifunctional Fluorescent Molecule Comprising Covalently Bonded Carbazole and Anthracene Moieties. J. Mater. Chem..

[176] Zhuang S., Shangguan R., Jin J., Tu G., Wang L., Chen J., Ma D., Zhu X. (2012). Efficient Nondoped Blue Organic Light-Emitting Diodes Based on Phenanthroimidazole-Substituted Anthracene Derivatives. Org. Electron..

[177] Park J. (2019). Highly Efficient Dual-Core Derivatives with EQEs as High as 8.38% at High Brightness for OLED Blue Emitters. J. Mater. Chem..

[178] Lai W.Y., Zhu R., Fan Q.L., Hou L.T., Cao Y., Huang W. (2006). Monodisperse Six-Armed Triazatruxenes: Microwave-Enhanced Synthesis and Highly Efficient Pure-Deep-Blue Electroluminescence. Macromolecules.

[179] Lai W.Y., He Q.Y., Zhu R., Chen Q.Q., Huang W. (2008). Kinked Star-Shaped Fluorene/Triazatruxene Co-Oligomer Hybrids with Enhanced Functional Properties for High-Performance, Solution-Processed, Blue Organic Light-Emitting Diodes. Adv. Funct. Mater..

[180] You A., Be M.A.Y., In I. (2008). High Efficiency Deep-Blue Organic Light-Emitting Diode with a Blue Dye in Low-Polarity Host. Appl. Phys. Lett..

[181] Zhu M., Ye T., Li C.G., Cao X., Zhong C., Ma D., Qin J., Yang C. (2011). Efficient Solution-Processed Nondoped Deep-Blue Organic Light-Emitting Diodes Based on Fluorene-Bridged Anthracene Derivatives Appended with Charge Transport Moieties. J. Phys. Chem. C.

[182] Huang H., Fu Q., Zhuang S., Liu Y., Wang L., Chen J., Ma D., Yang C. (2011). Novel Deep Blue OLED Emitters with 1,3,5-Tri(Anthracen-10-Yl)Benzene-Centered Starburst Oligofluorenes. J. Phys. Chem. C.

[183] Liu C., Li Y., Zhang Y., Yang C., Wu H., Qin J., Cao Y. (2012). Solution-Processed, Undoped, Deep-Blue Organic Light-Emitting Diodes Based on Starburst Oligofluorenes with a Planar Triphenylamine Core. Chemistry.

[184] Zhi B., Gao Q., Li Z.H., Xia P.F., Wong M.S., Cheah K.W., Chen C.H. (2007). Efficient Deep-Blue Organic Light-Emitting Diodes: Arylamine-Substituted Oligofluorenes. Adv. Funct. Mater..

[185] Zhang T., Wang R., Ren H., Chen Z., Li J. (2012). Deep Blue Light-Emitting Polymers with Fluorinated Backbone for Enhanced Color Purity and Efficiency. Polymer.

[186] Meunmart D., Prachumrak N., Keawin T., Jungsuttiwong S., Sudyoadsuk T., Promark V. (2012). Bis(4-Diphenylaminophenyl)Carbazole End-Capped Fluorene as Solution-Processed Deep-Blue Light-Emitting and Hole-Transporting Materials for Electroluminescent Devices. Tetrahedron Lett..

[187] Liu C., Gu Y., Fu Q., Sun N., Zhong C., Ma D., Qin J., Yang C. (2012). Nondoped Deep-Blue Organic Light-Emitting Diodes with Color Stability and Very Low Efficiency Roll-off: Solution-Processable Small-Molecule Fluorophores by Phosphine Oxide Linkage. Chem.-A Eur. J..

[188] Liu C., Li Y., Li Y., Yang C., Wu H., Qin J., Cao Y. (2013). Efficient Solution-Processed Deep-Blue Organic Light-Emitting Diodes Based on Multibranched Oligofluorenes with a Phosphine Oxide Center. Chem. Mater..

[189] Giovanella U., Botta C., Galeotti F., Vercelli B., Battiato S., Pasini M. (2013). Perfluorinated Polymer with Unexpectedly Efficient Deep Blue Electroluminescence for Full-Colour OLED Displays and Light Therapy Applications. J. Mater. Chem. C Mater..

[190] McDowell J.J., Maier-Flaig F., Wolf T.J.A., Unterreiner A.N., Lemmer U., Ozin G. (2014). Synthesis and Application of Photolithographically Patternable Deep Blue Emitting Poly(3,6-Dimethoxy-9,9-Dialkylsilafluorene)s. ACS Appl. Mater. Interfaces.

[191] Hu L., Liu S., Xie G., Yang W., Zhang B. (2020). Bis(Benzothiophene-S,S-Dioxide) Fused Small Molecules Realize Solution-Processible, High-Performance and Non-Doped Blue Organic Light-Emitting Diodes. J. Mater. Chem. C Mater..

[192] Liu X., Liu W., Dongyu W., Wei X., Wang L., Wang H., Miao Y., Xu H., Yu J., Xu B. (2020). Deep-Blue Fluorescent Emitter Based on a 9,9-Dioctylfluorene Bridge with a Hybridized Local and Charge-Transfer Excited State for Organic Light-Emitting Devices with EQE Exceeding 8%. J. Mater. Chem. C..

[193] Fischer A., Forget S., Chénais S., Castex M., Adès D., Siove A., Denis C., Maisse P., Geffroy B. (2006). Highly Efficient Multilayer Organic Pure-Blue-Light Emitting Diodes with Substituted Carbazoles Compounds in the Emitting Layer. J. Phys. D Appl. Phys..

[194] Tseng R.J., Chiechi R.C., Wudl F., Yang Y. (2006). Highly Efficient 7,8,10-Triphenylfluoranthene-Doped Blue Organic Light-Emitting Diodes for Display Application. Appl. Phys. Lett..

[195] Jou J.H., Chiang P.H., Lin Y.P., Chang C.Y., Lai C.L. (2007). Hole-Transporting-Layer-Free High-Efficiency Fluorescent Blue Organic Light-Emitting Diodes. Appl. Phys. Lett..

[196] Li Z., Jiao B., Wu Z., Liu P., Ma L., Lei X., Wang D., Zhou G., Hu H., Hou X. (2013). Fluorinated 9,9′-Spirobifluorene Derivatives as Host Materials for Highly Efficient Blue Organic Light-Emitting Devices. J. Mater. Chem. C Mater..

[197] Yu J., Chen Z., Sakuratani Y., Suzuki H., Tokita M., Miyata S. (1999). A Novel Blue Light Emitting Diode Using Tris(2,3-Methyl-8-Hydroxyquinoline) Aluminum(III) as Emitter. Jpn. J. Appl. Phys..

[198] Chan L., Lee R., Hsieh C., Yeh H., Chen C. (2002). Optimization of High-Performance Blue Organic Light-Emitting Diodes Containing Tetraphenylsilane Molecular Glass Materials. J. Am. Chem. Soc..

[199] Liu D., Du M., Chen D., Ye K., Zhang Z., Liu Y., Wang Y. (2015). A Novel Tetraphenylsilane–Phenanthroimidazole Hybrid Host Material for Highly Efficient Blue Fluorescent, Green and Red Phosphorescent. J. Mater. Chem. C.

[200] Jou J.H., Li J.L., Sahoo S., Dubey D.K., Kumar Yadav R.A., Joseph V., Thomas K.R.J., Wang C.W., Jayakumar J., Cheng C.H. (2018). Enabling a 6.5% External Quantum Efficiency Deep-Blue Organic Light-Emitting Diode with a Solution-Processable Carbazole-Based Emitter. J. Phys. Chem. C.

[201] Yang J., Liu X., Liu Z., Wang L., Sun J., Guo Z., Xu H., Wang H., Zhao B., Xie G. (2020). Protonation-Induced Dual Fluorescence of a Blue Fluorescent Material with Twisted A–π–D–π–A Configuration. J. Mater. Chem. C Mater..

[202] Wu Q., Braveenth R., Zhang H.Q., Bae I.J., Kim M., Chai K.Y. (2018). Oxadiazole-Based Highly Efficient Bipolar Fluorescent Emitters for Organic Light-Emitting Diodes. Molecules.

[203] Wang Y., Huang H., Wang Y., Zhu Q., Qin J. (2018). Simple Oxadiazole Derivatives as Deep Blue Fluorescent Emitter and Bipolar Host for Organic Light-Emitting Diodes. OptMa.

[204] Tang C., VanSlyke S.A. (1987). Organic Electroluminescent Diodes. Appl. Phys. Lett..

[205] Wu C.-C., Lin Y.-T., Wong K.-T., Chen R.-T., Chien Y.-Y. (2004). Efficient Organic Blue-Light-Emitting Devices with Double Confinement on Terfluorenes with Ambipolar Carrier Transport Properties. Adv. Mater..

[206] Lee M.-T., Liao C.-H., Tsai C.-H., Chen H. (2002). Highly Efficient, Deep-blue Doped Organic Light-emitting Devices. Adv. Mater..

[207] Baldo M., Thompson M., Forrest S. (2000). High-Efficiency Fluorescent Organic Light-Emitting Devices Using a Phosphorescent Sensitizer. Nature.

[208] Kido J., Kimura M., Science K.N. (1995). Multilayer White Light-Emitting Organic Electroluminescent Device. Science.

[209] Benmansour B.H., Shioya T., Sato Y., Bazan G.C. (2003). Anthracene-Containing Binaphthol Chromophores for Light-Emitting Diode (LED) Fabrication. Adv. Funct. Mater..

[210] Holmes R.J., Forrest S.R., Sajoto T., Tamayo A., Djurovich P.I., Thompson M.E., Brooks J., Tung Y.J., D’Andrade B.W., Weaver M.S. (2005). Saturated Deep Blue Organic Electrophosphorescence Using a Fluorine-Free Emitter. Appl. Phys. Lett..

[211] Xiaohui Yang B., Wang Z., Madakuni S., Li J., Jabbour G.E., Jabbour G.E., Yang X., Li J., Wang Z., Madakuni S. (2008). Efficient Blue- and White-Emitting Electrophosphorescent Devices Based on Platinum(II) [1,3-Difluoro-4,6-Di(2-Pyridinyl)Benzene] Chloride. Adv. Mater..

[212] Sasabe H., Takamatsu J.I., Motoyama T., Watanabe S., Wagenblast G., Langer N., Molt O., Fuchs E., Lennartz C., Kido J. (2010). High-Efficiency Blue and White Organic Light-Emitting Devices Incorporating a Blue Iridium Carbene Complex. Adv. Mater..

[213] Hang X.-C., Fleetham T., Turner E., Brooks J., Li J., Hang X., Fleetham T., Turner E., Li J., Brooks J. (2013). Highly Efficient Blue-Emitting Cyclometalated Platinum(II) Complexes by Judicious Molecular Design. Angew. Chem. Int. Ed..

[214] Ertl C.D., Cerdá J., Junquera-Hernández J.M., Pertegás A., Bolink H.J., Constable E.C., Neuburger M., Ortí E., Housecroft C.E. (2015). Colour Tuning by the Ring Roundabout: [Ir(C^N)_2_(N^N)]^+^ Emitters with Sulfonyl-Substituted Cyclometallating Ligands. RSC Adv..

[215] He L., Wang Z., Duan L., Yang C., Tang R., Song X., Pan C. (2016). Toward Fluorine-Free Blue-Emitting Cationic Iridium Complexes: To Generate Emission from the Cyclometalating Ligands with Enhanced Triplet Energy. Dalton Trans..

[216] Shavaleev N.M., Scopelliti R., Grätzel M., Nazeeruddin M.K. (2012). Phosphorescent cationic iridium(III) complexes with cyclometalating 1H-indazole and 2H-[1,2,3]-triazole ligands. Inorganica Chim. Acta.

[217] Li J., Djurovich P.I., Alleyne B.D., Yousufuddin M., Ho N.N., Thomas J.C., Peters J.C., Bau R., Thompson M.E. (2005). Synthetic Control of Excited-State Properties in Cyclometalated Ir(III) Complexes Using Ancillary Ligands. Inorg. Chem..

[218] Holmes R.J., D’Andrade B.W., Forrest S.R., Ren X., Li J., Thompson M.E. (2003). Efficient, Deep-Blue Organic Electrophosphorescence by Guest Charge Trapping. Appl. Phys. Lett..

[219] Lee S.J., Park K.M., Yang K., Kang Y. (2009). Blue Phosphorescent Ir(III) Complex with High Color Purity: Fac-Tris(2′,6′-Difluoro-2,3′-Bipyridinato-N,C 4′)Iridium(III). Inorg. Chem..

[220] Kang Y., Chang Y.L., Lu J.S., Ko S.B., Rao Y., Varlan M., Lu Z.H., Wang S. (2012). Highly Efficient Blue Phosphorescent and Electroluminescent Ir(III) Compounds. J. Mater. Chem. C Mater..

[221] Hsieh C.H., Wu F.I., Fan C.H., Huang M.J., Lu K.Y., Chou P.Y., Yang Y.H.O., Wu S.H., Chen I.C., Chou S.H. (2011). Design and Synthesis of Iridium Bis(Carbene) Complexes for Efficient Blue Electrophosphorescence. Chem.-A Eur. J..

[222] Fan C., Li Y., Yang C., Wu H., Qin J., Cao Y. (2012). Phosphoryl/Sulfonyl-Substituted Iridium Complexes as Blue Phosphorescent Emitters for Single-Layer Blue and White Organic Light-Emitting Diodes by Solution Process. Chem. Mater..

[223] Fan C., Zhu L., Jiang B., Zhong C., Ma D., Qin J., Yang C. (2013). Efficient Blue and Bluish-Green Iridium Phosphors: Fine-Tuning Emissions of FIrpic by Halogen Substitution on Pyridine-Containing Ligands. Org. Electron..

[224] Kessler F., Watanabe Y., Sasabe H., Katagiri H., Nazeeruddin M.K., Grätzel M., Kido J. (2013). High-Performance Pure Blue Phosphorescent OLED Using a Novel Bis-Heteroleptic Iridium(III) Complex with Fluorinated Bipyridyl Ligands. J. Mater. Chem. C Mater..

[225] Park H.J., Kim J.N., Yoo H.J., Wee K.R., Kang S.O., Cho D.W., Yoon U.C. (2013). Rational Design, Synthesis, and Characterization of Deep Blue Phosphorescent Ir(III) Complexes Containing (4′-Substituted-2′-Pyridyl)-1,2,4-Triazole Ancillary Ligands. J. Org. Chem..

[226] Lee S., Kim S.O., Shin H., Yun H.J., Yang K., Kwon S.K., Kim J.J., Kim Y.H. (2013). Deep-Blue Phosphorescence from Perfluoro Carbonyl-Substituted Iridium Complexes. J. Am. Chem. Soc..

[227] Liu L., Liu X., Wu K., Ding J., Zhang B., Xie Z., Wang L. (2014). Efficient Solution-Processed Blue Phosphorescent Organic Light-Emitting Diodes with Halogen-Free Solvent to Optimize the Emissive Layer Morphology. Org. Electron..

[228] Sun D., Zhou X., Li H., Sun X., Ren Z., Yan S. (2015). Multi-3,3 ′ -Bicarbazole-Substituted Arylsilane Host Materials with Balanced Charge Transport for Highly E Ffi Cient Solution-Processed Blue Phosphorescent Organic Light-Emitting Diodes. ACS Appl. Mater. Interfaces.

[229] Byeon S.Y., Choi J.M., Lee J.Y. (2016). Dyes and Pigments Pyridoindole Based Intramolecular Charge Transfer Type Host Material for Blue Phosphorescent Organic Light-Emitting Diodes. Dye. Pigment..

[230] Park S.R., Kim S.M., Kang J.H., Lee J.H., Suh M.C. (2017). Bipolar Host Materials with Carbazole and Dipyridylamine Groups Showing High Triplet Energy for Blue Phosphorescent Organic Light Emitting Diodes. Dye. Pigment..

[231] Yuan J., Jiang H., Yang Q., Xiang Y., Zhang Y., Dai Y., Li P., Zheng C., Xie G., Chen R. (2021). A solution-processable wholly-aromatic bipolar host material for highly efficient blue electroluminescent devices. J. Mater. Chem. C.

[232] Kang J., Zaen R., Park K.M., Lee K.H., Lee J.Y., Kang Y. (2020). Cyclometalated Platinum(II) β-Diketonate Complexes as Single Dopants for High-Efficiency White OLEDs: The Relationship between Intermolecular Interactions in the Solid State and Electroluminescent Efficiency. Cryst. Growth Des..

[233] Liao K.Y., Hsu C.W., Chi Y., Hsu M.K., Wu S.W., Chang C.H., Liu S.H., Lee G.H., Chou P.T., Hu Y. (2015). Pt(II) Metal Complexes Tailored with a Newly Designed Spiro-Arranged Tetradentate Ligand; Harnessing of Charge-Transfer Phosphorescence and Fabrication of Sky Blue and White OLEDs. Inorg. Chem..

[234] Liu Q.D., Wang R., Wang S. (2004). Blue Phosphorescent Zn(II) and Orange Phosphorescent Pt(II) Complexes of 4,4′-Diphenyl-6,6′-Dimethyl-2,2′-Bipyrimidine. Dalton Trans..

[235] Xu H., Xu Z.F., Yue Z.Y., Yan P.F., Wang B., Jia L.W., Li G.M., Sun W.B., Zhang J.W. (2008). A Novel Deep Blue-Emitting ZnII Complex Based on Carbazole-Modified 2-(2-Hydroxyphenyl)Benzimidazole: Synthesis, Bright Electroluminescence, and Substitution Effect on Photoluminescent, Thermal, and Electrochemical Properties. J. Phys. Chem. C.

[236] Oh C.S., Lee J.Y. (2013). High Triplet Energy Zn Complexes as Host Materials for Green and Blue Phosphorescent Organic Light-Emitting Diodes. Dye. Pigment..

[237] Soo Yook K., Yeob Lee J., Yook K.S., Lee J.Y. (2012). Organic Materials for Deep Blue Phosphorescent Organic Light-emitting Diodes. Adv. Mater..

[238] Zhu M., Yang C. (2013). Blue Fluorescent Emitters: Design Tactics and Applications in Organic Light-Emitting Diodes. Chem. Soc. Rev..

[239] Endo A., Ogasawara M., Takahashi A., Yokoyama D., Kato Y., Adachi C., Adachi C., Endo A., Yokoyama D., Ogasawara M. (2009). Thermally Activated Delayed Fluorescence from Sn^4+^–Porphyrin Complexes and Their Application to Organic Light Emitting Diodes—A Novel Mechanism for Electroluminescence. Adv. Mater..

[240] Cui L.-S., Nomura H., Kim J.U., Nakanotani H., Adachi C., Cui L., Geng Y., Kim J.U., Nakanotani H., Adachi C. (2017). Controlling Singlet–Triplet Energy Splitting for Deep-Blue Thermally Activated Delayed Fluorescence Emitters. Angew. Chem. Int. Ed..

[241] Cai X., Su S.-J., Cai X.Y., Su S.-J. (2018). Marching Toward Highly Efficient, Pure-Blue, and Stable Thermally Activated Delayed Fluorescent Organic Light-Emitting Diodes. Adv. Funct. Mater..

[242] Su S.J., Chiba T., Takeda T., Kido J. (2008). Pyridine-Containing Triphenylbenzene Derivatives with High Electron Mobility for Highly Efficient Phosphorescent OLEDs. Adv. Mater..

[243] Cui L.S., Deng Y.L., Tsang D.P.K., Jiang Z.Q., Zhang Q., Liao L.S., Adachi C. (2016). Controlling Synergistic Oxidation Processes for Efficient and Stable Blue Thermally Activated Delayed Fluorescence Devices. Adv. Mater..

[244] Tao Y., Yuan K., Chen T., Xu P., Li H., Chen R., Zheng C., Zhang L., Huang W. (2014). Thermally Activated Delayed Fluorescence Materials towards the Breakthrough of Organoelectronics. Adv. Mater..

[245] Shin H., Lee S., Kim K.H., Moon C.K., Yoo S.J., Lee J.H., Kim J.J. (2014). Blue Phosphorescent Organic Light-Emitting Diodes Using an Exciplex Forming Co-Host with the External Quantum Efficiency of Theoretical Limit. Adv. Mater..

[246] Hirata S., Sakai Y., Masui K., Tanaka H., Lee S.Y., Nomura H., Nakamura N., Yasumatsu M., Nakanotani H., Zhang Q. (2014). Highly Efficient Blue Electroluminescence Based on Thermally Activated Delayed Fluorescence. Nat. Mater..

[247] Li W., Pan Y., Xiao R., Peng Q., Zhang S., Ma D., Li F., Shen F., Wang Y., Yang B. (2014). Employing ~100% Excitons in OLEDs by Utilizing a Fluorescent Molecule with Hybridized Local and Charge-Transfer Excited State. Adv. Funct. Mater..

[248] Zhang Q., Li B., Huang S., Nomura H., Tanaka H., Adachi C. (2014). Efficient Blue Organic Light-Emitting Diodes Employing Thermally Activated Delayed Fluorescence. Nat. Photonics.

[249] Zhang Q., Tsang D., Kuwabara H., Hatae Y., Li B., Takahashi T., Youn Lee S., Yasuda T., Adachi C., Zhang Q. (2015). Nearly 100% Internal Quantum Efficiency in Undoped Electroluminescent Devices Employing Pure Organic Emitters. Adv. Mater..

[250] Sasabe H., Seino Y., Kimura M., Kido J. (2012). A m -Terphenyl-Modifed Sulfone Derivative as a Host Material for High-Efficiency Blue and Green Phosphorescent OLEDs. Chem. Mater..

[251] Jeon S.O., Earmme T., Jenekhe S.A. (2014). New Sulfone-Based Electron-Transport Materials with High Triplet Energy for Highly Efficient Blue Phosphorescent Organic Light-Emitting Diodes. J. Mater. Chem. C Mater..

[252] Duan L., Qiao J., Sun Y., Qiu Y., Duan L., Qiao J., Sun Y.D., Qiu Y. (2011). Strategies to Design Bipolar Small Molecules for OLEDs: Donor-Acceptor Structure and Non-Donor-Acceptor Structure. Adv. Mater..

[253] Hsu F.M., Chien C.H., Hsieh Y.J., Wu C.H., Shu C.F., Liu S.W., Chen C.T. (2009). Highly Efficient Red Electrophosphorescent Device Incorporating a Bipolar Triphenylamine/Bisphenylsulfonyl-Substituted Fluorene Hybrid as the Host. J. Mater. Chem..

[254] Zhang Q., Li J., Shizu K., Huang S., Hirata S., Miyazaki H., Adachi C. (2012). Design of Efficient Thermally Activated Delayed Fluorescence Materials for Pure Blue Organic Light Emitting Diodes. J. Am. Chem. Soc..

[255] Romain M., Tondelier D., Geffroy B., Shirinskaya A., Jeannin O., Rault-Berthelot J., Poriel C. (2014). Spiro-Configured Phenyl Acridine Thioxanthene Dioxide as a Host for Efficient PhOLEDs. Chem. Commun..

[256] Lee C.W., Lee J.Y. (2014). A Hole Transport Material with Ortho-Linked Terphenyl Core Structure for High Power Efficiency in Blue Phosphorescent Organic Light-Emitting Diodes. Org. Electron..

[257] Seo J.A., Gong M.S., Lee J.Y. (2014). Thermally Stable Indoloacridine Type Host Material for High Efficiency Blue Phosphorescent Organic Light-Emitting Diodes. Org. Electron..

[258] Park M.S., Lee J.Y. (2013). High Efficiency Green and Blue Phosphorescent Organic Light-Emitting Diodes Using Pyrroloacridine Type Hole Transport Material. Mol. Cryst. Liq. Cryst..

[259] Kim M., Lee J.Y. (2012). Improved Power Efficiency in Deep Blue Phosphorescent Organic Light-Emitting Diodes Using an Acridine Core Based Hole Transport Material. Org. Electron..

[260] Kim M., Lee J.Y. (2012). Pyridine-Modified Acridine-Based Bipolar Host Material for Green Phosphorescent Organic Light-Emitting Diodes. Chem. Asian J..

[261] Park M.S., Lee J.Y. (2011). Indolo Acridine-Based Hole-Transport Materials for Phosphorescent OLEDs with over 20% External Quantum Efficiency in Deep Blue and Green. Chem. Mater..

[262] Seo J.A., Jeon S.K., Gong M.S., Lee J.Y., Noh C.H., Kim S.H. (2015). Long Lifetime Blue Phosphorescent Organic Light-Emitting Diodes with an Exciton Blocking Layer. J. Mater. Chem. C Mater..

[263] Nakanotani H., Higuchi T., Furukawa T., Masui K., Morimoto K., Numata M., Tanaka H., Sagara Y., Yasuda T., Adachi C. (2014). High-Efficiency Organic Light-Emitting Diodes with Fluorescent Emitters. Nat. Commun..

[264] Nakao K., Sasabe H., Komatsu R., Hayasaka Y., Ohsawa T., Kido J., Nakao K., Sasabe H., Komatsu R., Hayasaka Y. (2017). Significant Enhancement of Blue OLED Performances through Molecular Engineering of Pyrimidine-Based Emitter. Adv. Opt. Mater..

[265] Grimsdale A.C., Jacob J. (2012). Polymer-Based LEDs and Solar Cells. Polym. Sci. A Compr. Ref..

[266] Liou G.S., Yen H.J. (2012). Polyimides. Polym. Sci. A Compr. Ref..

[267] Wu S., Aonuma M., Zhang Q., Huang S., Nakagawa T., Kuwabara K., Adachi C. (2013). High-Efficiency Deep-Blue Organic Light-Emitting Diodes Based on a Thermally Activated Delayed Fluorescence Emitter. J. Mater. Chem. C Mater..

[268] High-Efficiency Diphenylsulfon Derivative-Based Organic Light-Emitting Diode Exhibiting Thermally-Activated Delayed Fluorescence|Request PDF. https://www.researchgate.net/publication/303448146_High-efficiency_diphenylsulfon_derivative-based_organic_light-emitting_diode_exhibiting_thermally-activated_delayed_fluorescence.

[269] Serevičius T., Nakagawa T., Kuo M.C., Cheng S.H., Wong K.T., Chang C.H., Kwong R.C., Xia S., Adachi C. (2013). Enhanced Electroluminescence Based on Thermally Activated Delayed Fluorescence from a Carbazole–Triazine Derivative. Phys. Chem. Chem. Phys..

[270] Kim M., Kyu Jeon S., Hwang S.-H., Yeob Lee J., Kim M., Hwang S., Jeon S.K., Lee J.Y. (2015). Stable Blue Thermally Activated Delayed Fluorescent Organic Light-Emitting Diodes with Three Times Longer Lifetime than Phosphorescent Organic Light-Emitting Diodes. Adv. Mater..

[271] Uoyama H., Goushi K., Shizu K., Nomura H., Adachi C. (2012). Highly Efficient Organic Light-Emitting Diodes from Delayed Fluorescence. Nature.

[272] Cho Y.J., Yook K.S., Lee J.Y. (2014). A Universal Host Material for High External Quantum Efficiency Close to 25% and Long Lifetime in Green Fluorescent and Phosphorescent OLEDs. Adv. Mater..

[273] Cho Y.J., Yook K.S., Lee J.Y. (2015). Cool and Warm Hybrid White Organic Light-Emitting Diode with Blue Delayed Fluorescent Emitter Both as Blue Emitter and Triplet Host. Sci. Rep..

[274] Lee D.R., Hwang S.H., Jeon S.K., Lee C.W., Lee J.Y. (2015). Benzofurocarbazole and Benzothienocarbazole as Donors for Improved Quantum Efficiency in Blue Thermally Activated Delayed Fluorescent Devices. Chem. Commun..

[275] Yi C.L., Ko C.L., Yeh T.C., Chen C.Y., Chen Y.S., Chen D.G., Chou P.T., Hung W.Y., Wong K.T. (2020). Harnessing a New Co-Host System and Low Concentration of New TADF Emitters Equipped with Trifluoromethyl- and Cyano-Substituted Benzene as Core for High-Efficiency Blue OLEDs. ACS Appl. Mater. Interfaces.

[276] Numata M., Yasuda T., Adachi C. (2015). High Efficiency Pure Blue Thermally Activated Delayed Fluorescence Molecules Having 10 H -Phenoxaborin and Acridan Units. Chem. Commun..

[277] Tsai W.L., Huang M.H., Lee W.K., Hsu Y.J., Pan K.C., Huang Y.H., Ting H.C., Sarma M., Ho Y.Y., Hu H.C. (2015). A Versatile Thermally Activated Delayed Fluorescence Emitter for Both Highly Efficient Doped and Non-Doped Organic Light Emitting Devices. Chem. Commun..

[278] Li W., Li W., Gan L., Li M., Zheng N., Ning C., Chen D., Wu Y.C., Su S.J. (2020). J-Aggregation Enhances the Electroluminescence Performance of a Sky-Blue Thermally Activated Delayed-Fluorescence Emitter in Nondoped Organic Light-Emitting Diodes. ACS Appl. Mater. Interfaces.

[279] Yu Y.J., Wang X.Q., Liu J.F., Jiang Z.Q., Liao L.S. (2021). Harvesting Triplet Excitons for Near-Infrared Electroluminescence via Thermally Activated Delayed Fluorescence Channel. iScience.

[280] Evaristo De Sousa L., Dos L., Born S., Henrique De Oliveira Neto P., De Silva P. (2022). Triplet-to-Singlet Exciton Transfer in Hyperfluorescent OLED Materials. J. Mater. Chem. C Mater..

[281] Dias F.B., Bourdakos K.N., Jankus V., Moss K.C., Kamtekar K.T., Bhalla V., Santos J., Bryce M.R., Monkman A.P. (2013). Triplet Harvesting with 100% Efficiency by Way of Thermally Activated Delayed Fluorescence in Charge Transfer OLED Emitters. Adv. Mater..

[282] Li Z.W., Peng L.Y., Song X.F., Chen W.K., Gao Y.J., Fang W.H., Cui G. (2021). Room-Temperature Phosphorescence and Thermally Activated Delayed Fluorescence in the Pd Complex: Mechanism and Dual Upconversion Channels. J. Phys. Chem. Lett..

[283] Mamada M., Inada K., Komino T., Potscavage W.J., Nakanotani H., Adachi C. (2017). Highly Efficient Thermally Activated Delayed Fluorescence from an Excited-State Intramolecular Proton Transfer System. ACS Cent. Sci..

[284] Madayanad Suresh S., Hall D., Beljonne D., Olivier Y., Zysman-Colman E., Madayanad Suresh S., Hall D., Zysman-Colman E., Beljonne D., Olivier Y. (2020). Multiresonant Thermally Activated Delayed Fluorescence Emitters Based on Heteroatom-Doped Nanographenes: Recent Advances and Prospects for Organic Light-Emitting Diodes. Adv. Funct. Mater..

[285] Weissenseel S., Gottscholl A., Bönnighausen R., Dyakonov V., Sperlich A., Maximilian J. (2021). Long-Lived Spin-Polarized Intermolecular Exciplex States in Thermally Activated Delayed Fluorescence-Based Organic Light-Emitting Diodes. Sci. Adv..

[286] Sakai Y., Sagara Y., Nomura H., Nakamura N., Suzuki Y., Miyazaki H., Adachi C. (2015). Zinc Complexes Exhibiting Highly Efficient Thermally Activated Delayed Fluorescence and Their Application to Organic Light-Emitting Diodes. Chem. Commun..

[287] Kanno H., Ishikawa K., Nishio Y., Endo A., Adachi C., Shibata K. (2007). Highly Efficient and Stable Red Phosphorescent Organic Light-Emitting Device Using Bis[2-(2-Benzothiazoyl)Phenolato]Zinc(II) as Host Material. Appl. Phys. Lett..

[288] Hamada Y., Sano T., Fujii H., Nishio Y., Takahashi H., Shibata K. (1996). White-Light-Emitting Material for Organic Electroluminescent Devices. Jpn. J. Appl. Phys..

[289] Norikazu N., Shinichi W., Keiichi M., Tsuneo F. (2006). A Novel Blue Light Emitting Material Prepared from 2-(o-Hydroxyphenyl)Benzoxazole. Chem. Lett..

[290] Osawa M. (2014). Highly Efficient Blue-Green Delayed Fluorescence from Copper(i) Thiolate Complexes: Luminescence Color Alteration by Orientation Change of the Aryl Ring. Chem. Commun..

[291] Kang L., Chen J., Teng T., Chen X.L., Yu R., Lu C.Z. (2015). Experimental and Theoretical Studies of Highly Emissive Dinuclear Cu(i) Halide Complexes with Delayed Fluorescence. Dalton Trans..

[292] Zhang Q., Chen J., Wu X.Y., Chen X.L., Yu R., Lu C.Z. (2015). Outstanding Blue Delayed Fluorescence and Significant Processing Stability of Cuprous Complexes with Functional Pyridine–Pyrazolate Diimine Ligands. Dalton Trans..

[293] Zink D.M., Bächle M., Baumann T., Nieger M., Kühn M., Wang C., Klopper W., Monkowius U., Hofbeck T., Yersin H. (2013). Synthesis, Structure, and Characterization of Dinuclear Copper(I) Halide Complexes with P^N Ligands Featuring Exciting Photoluminescence Properties. Inorg. Chem..

[294] Deaton J.C., Switalski S.C., Kondakov D.Y., Young R.H., Pawlik T.D., Giesen D.J., Harkins S.B., Miller A.J.M., Mickenberg S.F., Peters J.C. (2010). E-Type Delayed Fluorescence of a Phosphine-Supported Cu 2(μ-Nar 2) 2 Diamond Core: Harvesting Singlet and Triplet Excitons in OLEDs. J. Am. Chem. Soc..

[295] Igawa S., Hashimoto M., Kawata I., Yashima M., Hoshino M., Osawa M. (2012). Highly Efficient Green Organic Light-Emitting Diodes Containing Luminescent Tetrahedral Copper(i) Complexes. J. Mater. Chem. C Mater..

[296] Osawa M., Kawata I., Ishii R., Igawa S., Hashimoto M., Hoshino M. (2013). Application of Neutral d 10 Coinage Metal Complexes with an Anionic Bidentate Ligand in Delayed Fluorescence-Type Organic Light-Emitting Diodes. J. Mater. Chem. C Mater..

[297] Volz D., Zink D.M., Bocksrocker T., Friedrichs J., Nieger M., Baumann T., Lemmer U., Bräse S. (2013). Molecular Construction Kit for Tuning Solubility, Stability and Luminescence Properties: Heteroleptic MePyrPHOS-Copper Iodide-Complexes and Their Application in Organic Light-Emitting Diodes. Chem. Mater..

[298] Ma Y., Che C.M., Chao H.Y., Zhou X., Chan W.H., Shen J. (1999). High Luminescence Gold(I) and Copper(I) Complexes with a Triplet Excited State for Use in Light-Emitting Diodes. Adv. Mater..

[299] Ma Y.G., Chan W.H., Zhou X.M., Che C.M. (1999). Light-Emitting Diode Device from a Luminescent Organocopper(I) Compound. New J. Chem..

[300] Cu(I) Complexes–Thermally Activated Delayed Fluorescence. Photophysical Approach and Material Design|Request PDF. https://www.researchgate.net/publication/304779001_CuI_complexes_-_Thermally_activated_delayed_fluorescence_Photophysical_approach_and_material_design.

[301] Tomé L.I.N., Domínguez-Pérez M., Cláudio A.F.M., Freire M.G., Marrucho I.M., Oscar Cabeza O.C., Coutinho J.A.P. (2009). On the Interactions between Amino Acids and Ionic Liquids in Aqueous Media. J. Phys. Chem. B.

[302] Housecroft C.E., Constable E.C. (2022). TADF: Enabling Luminescent Copper(I) Coordination Compounds for Light-Emitting Electrochemical Cells. J. Mater. Chem. C Mater..

[303] Teng T., Xiong J., Cheng G., Zhou C., Lv X., Li K. (2021). Solution-Processed Oleds Based on Thermally Activated Delayed Fluorescence Copper(I) Complexes with Intraligand Charge-Transfer Excited State. Molecules.

[304] Lv L.L., Yuan K., Wang Y.C. (2017). Theoretical Studying of Basic Photophysical Processes in a Thermally Activated Delayed Fluorescence Copper(I) Complex: Determination of Reverse Intersystem Crossing and Radiative Rate Constants. Org. Electron..

[305] Czerwieniec R., Leitl M.J., Homeier H.H.H., Yersin H. (2016). Cu(I) Complexes–Thermally Activated Delayed Fluorescence. Photophysical Approach and Material Design. Coord. Chem. Rev..

[306] Czerwieniec R., Yersin H. (2015). Diversity of Copper(I) Complexes Showing Thermally Activated Delayed Fluorescence: Basic Photophysical Analysis. Inorg. Chem..

[307] Linfoot C.L., Leitl M.J., Richardson P., Rausch A.F., Chepelin O., White F.J., Yersin H., Robertson N. (2014). Thermally Activated Delayed Fluorescence (TADF) and Enhancing Photoluminescence Quantum Yields of [CuI(Diimine)(Diphosphine)]^+^ Complexes-Photophysical, Structural, and Computational Studies. Inorg. Chem..

[308] Xu H., Yang T., Wang F., Zhang J., Zhang X., Wang H., Xu B. (2019). Thermally Activated Delayed Fluorescence of Copper(I) Complexes Using N, N′-Heteroaromatic of 2-(5-Phenyl-1,2,3-Triazole)Pyridine as Ligand. J. Lumin..

[309] Stoïanov A., Gourlaouen C., Vela S., Daniel C. (2018). Luminescent Dinuclear Copper(I) Complexes as Potential Thermally Activated Delayed Fluorescence (TADF) Emitters: A Theoretical Study. J. Phys. Chem. A.

[310] Leitl M.J., Zink D.M., Schinabeck A., Baumann T., Volz D., Yersin H. (2016). Copper(I) Complexes for Thermally Activated Delayed Fluorescence: From Photophysical to Device Properties. Top. Curr. Chem..

[311] Leitl M.J., Krylova V.A., Djurovich P.I., Thompson M.E., Yersin H. (2014). Phosphorescence versus Thermally Activated Delayed Fluorescence. Controlling Singlet-Triplet Splitting in Brightly Emitting and Sublimable Cu(I) Compounds. J. Am. Chem. Soc..

[312] Chen X.L., Yu R., Zhang Q.K., Zhou L.J., Wu X.Y., Zhang Q., Lu C.Z. (2013). Rational Design of Strongly Blue-Emitting Cuprous Complexes with Thermally Activated Delayed Fluorescence and Application in Solution-Processed OLEDS. Chem. Mater..

[313] Czerwieniec R., Yu J., Yersin H. (2011). Blue-Light Emission of Cu(I) Complexes and Singlet Harvesting. Inorg. Chem..

[314] Lee C.F., Chin K.F., Peng S.M., Che C.M. (1993). A Luminescent Tetrameric Zinc(II) Complex Containing the 7-Azaindolate Ligand. Photophysical Properties and Crystal Structure. J. Chem. Soc. Dalton Trans..

[315] Yu G., Yin S., Liu Y., Shuai Z., Zhu D. (2003). Structures, Electronic States, and Electroluminescent Properties of a Zinc(II) 2-(2-Hydroxyphenyl)Benzothiazolate Complex. J. Am. Chem. Soc..

[316] Xu X., Liao Y., Yu G., You H., Di C., Su Z., Ma D., Wang Q., Li S., Wang S. (2007). Charge Carrier Transporting, Photoluminescent, and Electroluminescent Properties of Zinc(II)-2-(2-Hydroxyphenyl)Benzothiazolate Complex. Chem. Mater..

[317] Singh S.P., Mohapatra Y.N., Qureshi M., Manoharan S.S. (2005). White Organic Light-Emitting Diodes Based on Spectral Broadening in Electroluminescence Due to Formation of Interfacial Exciplexes. Appl. Phys. Lett..

[318] Hao Y., Meng W., Xu H., Wang H., Liu X., Xu B. (2011). White Organic Light-Emitting Diodes Based on a Novel Zn Complex with High CRI Combining Emission from Excitons and Interface-Formed Electroplex. Org. Electron..

[319] Roh S.G., Kim Y.H., Seo K.D., Lee D.H., Kim H.K., Park Y.I., Park J.W., Lee J.H. (2009). Synthesis, Photophysical, and Electroluminescent Device Properties of Zn(II)-Chelated Complexes Based on Functionalized Benzothiazole Derivatives. Adv. Funct. Mater..

[320] Li Z., Dellali A., Malik J., Motevalli M., Nix R.M., Olukoya T., Peng Y., Ye H., Gillin W.P., Hernández I. (2013). Luminescent Zinc(II) Complexes of Fluorinated Benzothiazol-2-Yl Substituted Phenoxide and Enolate Ligands. Inorg. Chem..

[321] Wang R., Deng L., Fu M., Cheng J., Li J. (2012). Novel Zn II Complexes of 2-(2-Hydroxyphenyl)Benzothiazoles Ligands: Electroluminescence and Application as Host Materials for Phosphorescent Organic Light-Emitting Diodes. J. Mater. Chem..

[322] Son H.J., Han W.S., Chun J.Y., Kang B.K., Kwon S.N., Ko J., Su J.H., Lee C., Sung J.K., Sang O.K. (2008). Generation of Blue Light-Emitting Zinc Complexes by Band-Gap Control of the Oxazolylphenolate Ligand System: Syntheses, Characterizations, and Organic Light Emitting Device Applications of 4-Coordinated Bis(2-Oxazolylphenolate) Zinc(II) Complexes. Inorg. Chem..

[323] Zhang Q., Komino T., Huang S., Matsunami S., Goushi K., Adachi C. (2012). Triplet Exciton Confinement in Green Organic Light-Emitting Diodes Containing Luminescent Charge-Transfer Cu(I) Complexes. Adv. Funct. Mater..

[324] Hashimoto M., Igawa S., Yashima M., Kawata I., Hoshino M., Osawa M. (2011). Highly Efficient Green Organic Light-Emitting Diodes Containing Luminescent Three-Coordinate Copper(I) Complexes. J. Am. Chem. Soc..

[325] Cheng G., So G.K.M., To W.P., Chen Y., Kwok C.C., Ma C., Guan X., Chang X., Kwok W.M., Che C.M. (2015). Luminescent Zinc(II) and Copper(I) Complexes for High-Performance Solution-Processed Monochromic and White Organic Light-Emitting Devices. Chem. Sci..

[326] Nam S., Kim J.W., Bae H.J., Maruyama Y.M., Jeong D., Kim J., Kim J.S., Son W.J., Jeong H., Lee J. (2021). Improved Efficiency and Lifetime of Deep-Blue Hyperfluorescent Organic Light-Emitting Diode Using Pt(II) Complex as Phosphorescent Sensitizer. Adv. Sci..

[327] Jeon S.O., Lee K.H., Kim J.S., Ihn S.G., Chung Y.S., Kim J.W., Lee H., Kim S., Choi H., Lee J.Y. (2021). High-Efficiency, Long-Lifetime Deep-Blue Organic Light-Emitting Diodes. Nat. Photonics.

[328] Bulović V., Deshpande R., Thompson M.E., Forrest S.R. (1999). Tuning the Color Emission of Thin Film Molecular Organic Light Emitting Devices by the Solid State Solvation Effect. Chem. Phys. Lett..

[329] Chen W.C., Yuan Y., Ni S.F., Tong Q.X., Wong F.L., Lee C.S. (2017). Achieving Efficient Violet-Blue Electroluminescence with CIEy <0.06 and EQE >6% from Naphthyl-Linked Phenanthroimidazole–Carbazole Hybrid Fluorophores. Chem. Sci..

[330] Lin S.L., Chan L.H., Lee R.H., Yen M.Y., Kuo W.J., Chen C.T., Jeng R.J. (2008). Highly Efficient Carbazole-π-Dimesitylborane Bipolar Fluorophores for Nondoped Blue Organic Light-Emitting Diodes. Adv. Mater..

[331] Reig M., Puigdollers J., Velasco D. (2014). Molecular Order of Air-Stable p-Type Organic Thin-Film Transistors by Tuning the Extension of the π-Conjugated Core: The Cases of Indolo[3,2-b]Carbazole and Triindole Semiconductors. J. Mater. Chem. C Mater..

[332] Chen S., Sun B., Hong W., Yan Z., Aziz H., Meng Y., Hollinger J., Seferos D.S., Li Y. (2014). Impact of N-Substitution of a Carbazole Unit on Molecular Packing and Charge Transport of DPP–Carbazole Copolymers. J. Mater. Chem. C Mater..

[333] Yu M., Wang S., Shao S., Ding J., Wang L., Jing X., Wang F. (2015). Starburst 4,4′,4″-Tris(Carbazol-9-Yl)-Triphenylamine-Based Deep-Blue Fluorescent Emitters with Tunable Oligophenyl Length for Solution-Processed Undoped Organic Light-Emitting Diodes. J. Mater. Chem. C Mater..

[334] Xiao J., Yang S. (2011). Sequential Crystallization of Sea Urchin-like Bimetallic (Ni, Co) Carbonate Hydroxide and Its Morphology Conserved Conversion to Porous NiCo2O4 Spinel for Pseudocapacitors. RSC Adv..

[335] Sahoo S., Dubey D.K., Singh M., Joseph V., Thomas K.R.J., Jou J.H. (2018). Highly Efficient Deep-Blue Organic Light Emitting Diode with a Carbazole Based Fluorescent Emitter. Jpn. J. Appl. Phys..

[336] Huang C.C., Xue M.M., Wu F.P., Yuan Y., Liao L.S., Fung M.K. (2019). Deep-Blue and Hybrid-White Organic Light Emitting Diodes Based on a Twisting Carbazole-Benzofuro[2,3-b]Pyrazine Fluorescent Emitter. Molecules.

[337] Pal A.K., Krotkus S., Fontani M., Mackenzie C.F.R., Cordes D.B., Slawin A.M.Z., Samuel I.D.W., Zysman-Colman E., Pal A.K., Fontani M. (2018). High-Efficiency Deep-Blue-Emitting Organic Light-Emitting Diodes Based on Iridium(III) Carbene Complexes. Adv. Mater..

[338] Wang R., Liu Y., Hu T., Wei X., Liu J., Li Z., Hu X., Yi Y., Wang P., Wang Y. (2019). Solution-Processed White Organic Light-Emitting Diodes with Bi-Component Emitting Layer Based on Symmetry Blue Spiro-Sulfone Derivative. Org. Electron..

[339] Weissenseel S., Drigo N.A., Kudriashova L.G., Schmid M., Morgenstern T., Lin K.H., Prlj A., Corminboeuf C., Sperlich A., Brütting W. (2019). Getting the Right Twist: Influence of Donor-Acceptor Dihedral Angle on Exciton Kinetics and Singlet-Triplet Gap in Deep Blue Thermally Activated Delayed Fluorescence Emitter. J. Phys. Chem. C.

[340] Chan C.-Y., Cui L.-S., Uk Kim J., Nakanotani H., Adachi C., Chan C., Cui L., Kim J.U., Nakanotani H., Adachi C. (2018). Rational Molecular Design for Deep-Blue Thermally Activated Delayed Fluorescence Emitters. Adv. Funct. Mater..

[341] Luo Y., Li S., Zhao Y., Li C., Pang Z., Huang Y., Yang M., Zhou L., Zheng X., Pu X. (2020). An Ultraviolet Thermally Activated Delayed Fluorescence OLED with Total External Quantum Efficiency over 9%. Adv. Mater..

[342] Gudeika D., Bezvikonnyi O., Volyniuk D., Skuodis E., Lee P.H., Chen C.H., Ding W.C., Lee J.H., Chiu T.L., Grazulevicius J.V. (2020). Oxygen Sensing and OLED Applications of Di-Tert-Butyl-Dimethylacridinyl Disubstituted Oxygafluorene Exhibiting Long-Lived Deep-Blue Delayed Fluorescence. J. Mater. Chem. C Mater..

[343] Yang J., Guo Q., Wang J., Ren Z., Chen J., Peng Q., Ma D., Li Z., Yang J., Wang J. (2018). Rational Molecular Design for Efficient Exciton Harvesting, and Deep-Blue OLED Application. Adv. Opt. Mater..

[344] Fu C., Luo S., Li Z., Ai X., Pang Z., Li C., Chen K., Zhou L., Li F., Huang Y. (2019). Highly Efficient Deep-Blue OLEDs Based on Hybridized Local and Charge-Transfer Emitters Bearing Pyrene as the Structural Unit. Chem. Commun..

[345] Yu Y., Zhao R., Liu H., Zhang S., Zhou C., Gao Y., Li W., Yang B. (2020). Highly Efficient Deep-Blue Light-Emitting Material Based on V-Shaped Donor-Acceptor Triphenylamine-Phenanthro[9,10-d]Imidazole Molecule. Dye. Pigment..

[346] Usta H., Alimli D., Ozdemir R., Dabak S., Zorlu Y., Alkan F., Tekin E., Can A. (2019). Highly Efficient Deep-Blue Electroluminescence Based on a Solution-Processable A-π-D-π-A Oligo(p-Phenyleneethynylene) Small Molecule. ACS Appl. Mater. Interfaces.

[347] Zhang H., Xue J., Li C., Zhang S., Yang B., Liu Y., Wang Y., Zhang H., Xue J., Li C. (2021). Novel Deep-Blue Hybridized Local and Charge-Transfer Host Emitter for High-Quality Fluorescence/Phosphor Hybrid Quasi-White Organic Light-Emitting Diode. Adv. Funct. Mater..

[348] Kondakov D.Y., Pawlik T.D., Hatwar T.K., Spindler J.P. (2009). Triplet Annihilation Exceeding Spin Statistical Limit in Highly Efficient Fluorescent Organic Light-Emitting Diodes. J. Appl. Phys..

[349] Turshatov A.A., Baluschev S.B. (2012). Triplet-Triplet Annihilation Assisted Upconversion: All-Optical Tools for Probing Physical Parameter of Soft Matter. Handbook of Coherent-Domain Optical Methods.

[350] Hu J.Y., Pu Y.J., Satoh F., Kawata S., Katagiri H., Sasabe H., Kido J. (2014). Bisanthracene-Based Donor–Acceptor-Type Light-Emitting Dopants: Highly Efficient Deep-Blue Emission in Organic Light-Emitting Devices. Adv. Funct. Mater..

[351] Chen Y.H., Lin C.C., Huang M.J., Hung K., Wu Y.C., Lin W.C., Chen-Cheng R.W., Lin H.W., Cheng C.H. (2016). Superior Upconversion Fluorescence Dopants for Highly Efficient Deep-Blue Electroluminescent Devices. Chem. Sci..

[352] Zeng W., Zhao Y., Ning W., Gong S., Zhu Z., Zou Y., Lu Z.H., Yang C. (2018). Efficient Non-Doped Fluorescent OLEDs with Nearly 6% External Quantum Efficiency and Deep-Blue Emission Approaching the Blue Standard Enabled by Quaterphenyl-Based Emitters. J. Mater. Chem. C Mater..

[353] Peng L., Yao J.W., Wang M., Wang L.Y., Huang X.L., Wei X.F., Ma D.G., Cao Y., Zhu X.H. (2019). Efficient Soluble Deep Blue Electroluminescent Dianthracenylphenylene Emitters with CIE y (Y ≤ 0.08) Based on Triplet-Triplet Annihilation. Sci. Bull..

[354] Wu Z., Song S., Zhu X., Chen H., Chi J., Ma D., Zhao Z., Tang B.Z. (2021). Highly Efficient Deep-Blue Fluorescent OLEDs Based on Anthracene Derivatives with a Triplet–Triplet Annihilation Mechanism. Mater. Chem. Front..

[355] 《擁抱暗黑》新書講座(憑書報名入場)|ACCUPASS. https://www.accupass.com/event/2011030819584976501710.

