# Supplementary material for: Status and Challenges of Blue OLEDs: A Review
Source: Nanomaterials (Basel). 2023 Sep 8;13(18):2521. doi: 10.3390/nano13182521 (PMC10536903; doi:10.3390/nano13182521)
Supplement: Supplementary file 1 [file nanomaterials-13-02521-s001.zip › nanomaterials-2590386-supplementary.pdf]

Status and Challenges of Blue OLEDs: A Review

Iram Siddiqui [1], Sudhir Kumar [2], Yi-Fang Tsai [1], Prakalp Gautam [1], Shahnawaz [1], Kiran Kesavan [1], Jin-Ting Lin [1], Luke Khai [1], Kuo-Hsien Chou [1], Abhijeet Choudhury [1], Saulius Grigalevicius [3,*] and Jwo-Huei Jou [1,*]

[1] Department of Materials Science and Engineering, National Tsing Hua University, Hsinchu 30013, Taiwan; iramsidd29@gmail.com (I.S.); amyapple19901203@gmail.com (Y.-F.T.); prakalpgautam@gmail.com (P.G.); shaan88usmani@gmail.com (S.); kirankishorek7@gmail.com (K.K.); s10030267@gmail.com (J.-T.L.); lukethe168@gmail.com (L.K.); sfcb352@yahoo.com (K.-H.C.); abhijeetchoudhury101@gmail.com (A.C.)

[2] Institute for Chemical and Bioengineering, ETH Zürich, 8093 Zürich, Switzerland; sudhir.kumar@chem.ethz.ch

[3] Department of Polymer Chemistry and Technology, Kaunas University of Technology, LT50254 Kaunas, Lithuania

* Correspondence: saulius.grigalevicius@ktu.lt (S.G.); jjou@mx.nthu.edu.tw (J.-H.J.)

Table S1. A comprehensive list of dry-processed blue fluorescent OLED devices, including the publication year, device composition, key dopant, doping concentration, employed host, HOMO, LUMO, EQE, PE, CE, CIE (X), CIE (Y), and reference. The years span from 2006 to 2020

Device Structure	Host	Emitter				EQE (%)			PE (lm/W)			CE (cd/A)			CIE		Year	Ref.
		Name	Conc. (wt%)	HOMO	LUMO	Max	100 (cdm^{-2})	1000 (cdm^{-2})	Max	100 (cdm^{-2})	1000 (cdm^{-2})	Max	100 (cdm^{-2})	1000 (cdm^{-2})	(X)	(Y)		
ITO/NPB/Ph3Si(PhTPAOXD)/Alq3/Mg:Ag	-	Ph_3Si(PhTPAOXD)	-	-	2.7	1.7	-	-	0.9	-	-	2	-	-	0.17	0.17	2001	[1]

	-	Ph_3Si(PhTPAOXD)	-	-	2.7	1.1	-	-	0.2	-	-	0.8	-	-	0.17	0.08		
ITO/NPB/Ph3Si:PhTPAOXD/Alq3/Mg:Ag	-	Ph3Si(PhTPAOXD)	-	5.4	-	1.8	-	-	1.1	-	-	3.4	-	-	0.18	0.28		
ITO/Ph2Si(Ph(NPA)2)2/Ph3Si(PhTPAOXD)/ Alq3/Mg:Ag	-	$Ph_2Si(Ph(NPA)_2)_2$	-	5.2	-	3	-	-	3.1	-	-	6.1	-	-	0.21	0.33	2002	[2]
ITO/Ph2Si(Ph(NPA)2)2/NPB/Ph3Si(PhTPAOXD)/Alq3/Mg:Ag	-	$Ph_2Si(Ph(NPA)_2)_2$	-	5.2	2.65	2.4	-	-	1.1	-	-	3.1	-	-	0.16	0.18		
ITO/CuPc/α-NPD/EML/SAlq/Alq3/LiF/Al	-	BA1	-	-	-	-	-	-	-	0.2	-	-	0.7	-	0.18	0.21		
ITO/CuPc/α-NPD/EML/SAlq/Alq3/LiF/Al	-	BA2	-	-	2.9	-	-	-	-	0.6	-	-	1.5	-	0.22	0.33	2003	[3]
ITO/CuPc/α-NPD/EML/SAlq/Alq3/LiF/Al	CBP	BA2	-	-	2.9	-	-	-	-	0.9	-	-	2.2	-	0.19	0.21		

ITO/NPB/MeC1/TPBI/Mg9:Ag1	-	MeC1	-	-	2.93	2.7	-	-	1.1	-	-	3.2	-	-	458	-	2003	[4]
ITO/XTPS/TPBI/Mg9:Ag1	-	XTPS	-	-	-	4.1	-	-	2.9	-	-	5.5	-	-	462	-		
ITO/CuPc/NPB/spiro-FPA/Alq3/LiF/Al.	-	Spiro-FPA	-	5.47	-	-	-	-	1.15	-	-	2.76	-	-	0.16	0.14	2004	[5]
ITO/CuPc/NPB/spiro-FPA:TBP/Alq3/LiF/Al.	TBP		1	5.47	-	-	-	-	2.07	-	-	4.88	-	-	0.14	0.2		
ITO/CuPc/α-NPD/BDSA/Alq3/LiF/Al	-	BDSA	100	5.91	2.53	-	-	-	-	-	-	2.6	2.6	-	0.14	0.1	2005	[6]
ITO/CuPc/α-NPD/BTSA/Alq3/LiF/Al	-	BTSA	100	6.17	-	-	-	-	-	-	-	1.4	0.8	-	0.15	0.09		
ITO/CuPc/NPB/DPF/Alq3/ Mg:Ag	-	DPF	100	5.7	-	-	-	-	-	-	-	4.8	-	-	0.17	0.24	2005	[7]
ITO/CuPc/NPB/DPF/TPBi/ Mg:Ag	-	DPF	100	5.7	-	-	-	-	3	-	-	5.3	-	-	0.16	0.22		

ITO/CFx/NPB/blue host/ Alq/LiF/Al	DPVPA	DSA-Ph	3%	5.4	-	-	-	-	4.8	-	-	10.2	-	-	0.16	0.35	2005	[8]
ITO/CFx/NPB/blue host/ Alq/LiF/Al	MADN	DSA-Ph	3%	5.4	-	1.5	-	-	5.5	-	-	9.7	-	-	0.16	0.32		
ITO/CFx/NPB/blue host/Alq/LiF/Al	MADN	BD-3		-	-	4.2	-	-	-	-	-	5.6	-	-	0.14	0.17		
ITO/CFx/NPB/blue host/Alq/LiF/Al	DPVPA	nondoped		-	-	3	-	-	1.7	-	-	4	-	-	0.14	0.17		
ITO/CuPc/NPB/DPhDPF/Alq3/Mg:Ag	-	DPhDPF	100	5.7	-	-	-	-	-	-	-	5	-	-	0.17	0.26	2005	[7]
ITO/CuPc/NPB/SDPF/Alq3/Mg:Ag	-	SDPF	100	5.8	-	-	-	-	-	-	-	4.9	-	-	0.17	0.27		
ITO/CuPc/NPB/PMC/BCP/Alq3/LiF/Al	-	PMC	100	5.9	2.88	0.6	-	-	0.2	-	-	0.4	-	-	0.153	0.1	2006	[9]
ITO/CuPc/NPB/DPVBi :PMC/ Alq3/LiF/Al	DPVBi		5	5.9	5.42	2.8	-	-	1.2	-	-	4.1	-	-	0.16	0.176		
ITO/CuPc/NPB/DEC/BCP/Alq3/LiF/Al	-	DEC	100	5.6	-	1.5	-	-	-	-	-	-	-	-	0.192	0.209		

ITO/CuPc/NPB/DPVBi: DEC/Alq3/LiF/Al	DPVBi		2	5.6	-	3.3	-	-	1.3	-	-	4.7	-	-	0.158	0.169		
ITO/CuPc/NPB/DPVBi/Alq3/LiF/Al	-	DPVBi	100	5.9	2.93	2.7	-	-	1.2	-	-	2.4	-	-	0.149	0.112		
ITO/PEDOT/NPB/CBP/blue-emitting layer/BCP/Ca/Al	-	TPF	100	6.3	2.3	1.75	-	-	-	-	-	2.89	-	-	0.177	0.24		
ITO/PEDOT/NPB/CBP/blue-emitting layer /BCP/Ca/Al	DPF		6	-	2.7	2.48	-	-	-	-	-	3.33	-	-	0.164	0.188	2006	[10]
ITO/PEDOT/NPB/CBP/blue-emitting layer/BCP/Ca/Al	-	DPF	100	-	2.57	0.72	-	-	-	-	-	0.84	-	-	0.168	0.157		
ITO/NPB/TCTA/EML/BPhen/LiF/Al	CBP	OF(3)-NPh	4	5.15	-	4.1	3.1	-	1.01a	-	-	2.9	2.24	-	0.152	0.083		
ITO/NPB/TCTA/EML/BPhen/LiF/Al	CBP	SOF(3)-NPh	4	5.2	3	3.2	-	-	1.24a	-	-	2.7	-	-	0.151	0.099	2007	[11]
ITO/NPB/TCTA/EML/BPhen/LiF/Al	CBP	OF(2)-PhNPh	4	5.2	-	2.9	-	-	0.8a	-	-	1.75	-	-	0.153	0.072		

ITO/PEDOT/TFTPA /TPVAn/TPBi/ Mg:Ag	-	TPVAn	-	5.7	2.66	5.3	5	4.2	2.8	-	-	5.3	5	4.2	0.14	0.12	2007	[12]
ITO/blue emissive layer/TPBi/LiF/Al	-	BNE	100	5.2	-	-	6	-	-	12.5	-	-	-	-	0.18	0.33	2007	[13]
ITO/blue emissive layer/TPBi/LiF/Al	-	DPASN	100	5.2	1.1	-	3.9	-	-	6.7	-	-	-	-	0.15	0.2		
ITO/NPB/CzPhB/TPBI/LiF/Al	-	CzPhB	100	5.92	-	4.3	4.1	-	1.3	1.1	-	3.3	3.2	-	0.15	0.09	2008	[14]
ITO/NPB/ CzThB/TPBI/LiF/Al	-	CzThB	100	5.8	-	6.9	5.6	-	4.9	3.3	-	10.1	8.2	-	0.13	0.21		
ITO/2TNATA/NPB/ EML/Bphen/LiF/Al	-	PhQ-CVz	100	5.84	-	2.45	2.31	-	1.77	0.85	-	2.06	1.9	-	0.156	0.093	2008	[15]
ITO/2TNATA/NPB/ EML/Bphen/LiF/Al	-	MeO-PhQ-CVz	100	5.84	-	1.95	1.93	-	1.09	0.82	-	1.69	1.67	-	0.158	0.102		
ITO/2TNATA/NPB/ EML/Bphen/Li/A	PhQ-CVz	BCzVBi	5	-	-	5.22	4.19	-	5.98	2.44	-	6.9	5.44	-	0.155	0.157		
ITO/2TNATA/NPB/ EML/Bphen/LiF/Al	MeO-PhQ-CVz	BCzVBi	5	-	1.37	3.53	2.91	-	4	2.17	-	5.2	4.22	-	0.158	0.181		

ITO/PEDOT/CBP:MDP3FL/TPBi/LiF/Al	CBP	MDP3FL	4	5.4	1.37	-	4.6	-	-	1.4	-	-	-	-	0.14	0.06	2008	[16]
ITO/PEDOT/CBP:MDP3FL/TPBi/LiF/Al			10	5.4	-	-	5.1	-	-	1.9	-	-	-	-	0.14	0.08		
ITO/NPB/CBP/AlmND3:Dopant/AlmND3/LiF/Al	$AlmND_3$	DPA	0.5	-	2.87	2.8	-	-	0.89	-	-	1.39	-	-	0.15	0.06	2008	[17]
ITO/NPB/CBP/AlmND3:Dopant/AlmND3/LiF/Al		Perylene	0.5	-	-	3.56	-	-	3.06	-	-	4.67	-	-	0.14	0.17		
ITO/NPB/CBP/AlmmND3/AlND3/LiF/Al	-	$AlND_3$	100	-	2.53	3.67	-	-	1.04	-	-	2	-	-	0.15	0.07		
ITO/NPB/CBP/AlND3/AlmND3/LiF/Al	-	$AlND_3$	100	-	2.65	0.76	-	-	0.45	-	-	1.09	-	-	0.15	0.19		
ITO/NPB/CBP/AlmND3/AlmND3/LiF/Al	-	$AlmND_3$	100	6.4	2.48	1.91	-	-	0.77	-	-	1.43	-	-	0.15	0.09		
ITO/NPB/CBP/AlmND3/Bebq2/LiF/Al	-	$AlmND_3$	100	6.4	2.68	1.81	-	-	0.98	-	-	1.84	-	-	0.15	0.13		

ITO/NPB/CBP/AlmmND3/AlmND3/LiF/Al	-	$AlmmND_3$	100	-	2.66	4.18	-	-	0.86	-	-	2.27	-	-	0.15	0.07		
ITO/NPB/CBP/AlmpND3/AlmND3/LiF/Al	-	$AlmpND_3$	100	-	-	1.62	-	-	0.71	-	-	1.58	-	-	0.15	0.12		
ITO/2-TNATA/NPB/MAM/Alq_3/LiF/Al	-	MAM	100	-	-	3.9	-	-	1.28	-	-	2.94	-	-	0.163	0.124	2008	[18]
ITO/2-TNATA/NPB/MAT/Alq_3/LiF/Al	-	MAT	100	-	-	6.14	-	-	1.52	-	-	2.97	-	-	0.156	0.085		
ITO/2-TNATA/NPB/TAT/Alq_3/LiF/Al	-	TAT	100	-	-	7.18	-	-	1.87	-	-	3.64	-	-	0.156	0.088		
ITO/2-TNATA/NPB/MADN/Alq_3/LiF/Al	-	MADN	100	-	-	3.18	-	-	1.48	-	-	2.86	-	-	0.171	0.138		
ITO/NPB/cis-TSDTB/TPBI/LiF/Al	-	Cis-TSDTB	100	5.7	-	-	-	-	1.9	-	-	3.4	-	-	0.15	0.1	2008	[19]

ITO/BPAF/POAn/TPBI/LiF/Al	-	POAn	100	5.6	2.77	4.7	4.5	-	3.3	2.3	-	3.2	3	-	0.15	0.07	2009	[20]
ITO/NPB⁄DPF⁄TPBI⁄MgAg	-	DPF	100	5.7	2.37	-	-	-	3.6	-	-	6	-	-	0.15	0.19	2010	[21]
ITO/NPB/DCDPF/TPBi/LiF/MgAg	-	DCDPF	100	5.9	-	-	-	-	3.1	-	-	4.4	-	-	0.15	0.15	2010	[22]
ITO/DNTPD/α-NPB/MADN: dopant/Alq3/LiF/Al	MADN	BD-6DPA	5	5.48	-	6.54	-	-	4.43	-	-	7.79	-	-	0.135	0.175	2010	[23]
ITO/DNTPD)/α-NPB/MADN: dopant/Alq3/LiF/Al	MADN	BD-6NPA	5	5.44	-	7.32	-	-	4.61	-	-	8.56	-	-	0.134	0.168		
ITO/DNTPD/α-NPB/MADN: dopant/Alq3/LiF/Al	MADN	BD-6MDPA	5	5.42	-	8.16	-	-	6.13	-	-	9.11	-	-	0.135	0.156		

ITO/DNTPD/α-NPB/MADN: dopant/Alq3/LiF/Al	MADN	BD-6TMSA	5	5.45	-	7.45	-	-	5.12	-	-	8.11	-	-	0.135	0.151		
ITO/NPB/BN1/BN1: BN2/Alq3/LiF/Al	BN2	BN1	5	5.67	-	8.6	-	-		-	-	4.9	-	-	0.16	0.11	2010	[24]
ITO/PEDOT:PSS/NPB/EML/Alq_3/CsF/Mg:Ag	-	TBMFA	100	5.79	-	3.58	-	-	1.41	-	-	1.79	-	-	0.151	0.085		
ITO/PEDOT:PSS/NPB/EML/Alq_3/CsF/Mg:Ag	-	TBDNPA	100	5.83	2.4	5.17	-	-	1.96	-	-	2.63	-	-	0.149	0.086	2010	[25]
ITO/PEDOT:PSS/NPB/EML/Alq_3/CsF/Mg:Ag	-	TBMFPA	100	5.82	-	4.76	-	-	1.92	-	-	2.67	-	-	0.147	0.096		

ITO/NPB/ PCVtPh/BAlq/Liq/Al	-	PCVtPh	100	5.6	-	2.17(At a current density of 1 mA/cm2)	-		1.77(At a current density of 1 mA/cm2)	-		2.43(At a current density of 1 mA/cm2)	-	-	0.15	0.13		
ITO/NPB/ MADN:PCVtPh/BAlq/Liq/Al	MADN	PCVtPh	8	5.6	2.4	2.97(At a current density of 1 mA/cm2)	-		1.9(At a current density of 1 mA/cm2)	-		2.81(At a current density of 1 mA/cm2)	-	-	0.15	0.09	2010	[26]
ITO/NPB/ DNBN:PCVtPh/BAlq/Liq/Al	DNBN	PCVtPh	8	5.6	2.4	3.36 (At a current density of 1 mA/cm2)	-		1.74(At a current density of 1 mA/cm2)	-		3.02(At a current density of 1 mA/cm2)	-	-	0.15	0.09		

ITO/NPB/ MADN:PCVtPh/DNBN:PCVtPh/BAlq/Liq/Al	MADN/DNBN	PCVtPh	8	-	2.7	4.68	3.4	-		2.02	-	5.22	3.23	-	0.15	0.09		
ITO/NPB/MADN:DPAFS-2/Bphen/Liq/Al	MADN	DPAFS-2	15	5.43	2.6	5.9	5.8	-	11.6	7	-	12.1	11.8	-	0.17	0.3	2010	[27]
ITO/NPB/MADN:DPAFS-3/Bphen/Liq/Al	MADN	DPAFS-3	15	5.79	2.61	5.6	4.8	-	6.2	2.9	-	6	5.3	-	0.15	0.13		
ITO/NPB/MADN:DPAFS-4/Bphen/Liq/Al	MADN	DPAFS-4	10	5.67	2.71	5.3	5.1	-	8.7	5.3	-	9.5	9.1	-	0.17	0.24		
ITO/NPB/MADN:DPAFS-5/Bphen/Liq/Al	MADN	DPAFS-5	15	5.69	2.7	5.5	5.2	-	8.7	4.9	-	9.1	8.7	-	0.16	0.22		
ITO/NPB/MADN:blue dopants/Bphen/Liq/Al.	MADN	1,4-Bis(2-diphenylamino-9,9-diethylfluoren-7-	15%	5.6	2.4	6.3	-	-	15.8	-	-	16	-	-	0.2	0.43	2010	[27]

		ylethenyl)benzene																
ITO/NPB/MADN:blue dopants/Bphen/Liq/Al.	MADN	9-[4-(2-Diphenylamino-9,9-diethylfluoren-7-ylethenyl)phenyl]-9-phenylfluorene	15%	5.8	2.4	5.6	-	-	6.2	-	-	6	-	-	0.15	0.13		
ITO/BCP/Alq3/LiF/Al	-	AC	100	5.4	2.4	2.84	2.07	-	1.39	0.69	-	3.45	2.32	-	0.162	0.134	2011	[28]
ITO/2-TNATA/NPB/EML/BCP/Alq_3/LiF/Al	-	DAC	100	5.41	-	1.38	1.33	-	0.87	0.52	-	2.09	1.82	-	0.179	0.171		
ITO/2-TNATA/NPB/EML/BCP/Alq_3/LiF/Al	-	P-AC	100	5.47	-	2.53	2.35	-	1.19	0.72	-	3.11	2.56	-	0.163	0.132		
ITO/2-TNATA/NPB/EML/BCP/Alq_3/LiF/Al	-	P-DAC	100	5.5	-	2.5	2.75	-	1.28	0.92	-	3.09	3.14	-	0.162	0.136		

ITO/NPB/Be(4-mpp)2/LiF/Al	-	Be(4-mmp)$_2$	100	5.7	-	5.4	5.22	5.15	4.2	4.1	3	-	-	-	0.14	0.09	2011	[29]
ITO/NPB/Bepp2/LiF/Al	-	Bepp$_2$	100	5.7	-	3.9	3.9	3.75	4.1	3.95	2.85	-	-	-	0.16	0.18		
-	-	-	-	-	-	5.8	-	4.39	2.86	-	-	3.82	-	-	0.15	0.08	2011	[30]
ITO/2-TNATA/NPB/EML/TPBi/(CH3)3CCOOCs/Al	-	ACPI	100	5.62	-	3.52	3.27	-	3.03	2.55	-	4.36	4.17	-	0.156	0.155	2012	[31]
ITO/2-TNATA/NPB/EML/TPBi/(CH3)3CCOOCs/Al	-	1-NaCPI	100	5.58	-	3.23	3.13	-	2.46	2.12	-	4.22	4.13	-	0.15	0.167		
ITO/2-TNATA/NPB/EML/TPBi/(CH3)3CCOOCs/Al	-	2-NaCPI	100	5.55	-	3.68	3.65	-	3.6	2.95	-	4.68	4.67	-	0.151	0.157		

ITO/DNTPD/NPB/FP-SBFF:dopant/Alq3/LiF/Al	DN-SBFF	TPA-SBFF	7	5.44	-	4.69	-	-	3.35	-	-	7.14	-	-	0.14	0.169	2012	[32]
ITO/DNTPD/NPB/FP-SBFF:dopant/Alq3Alq3/LiF	BP-SBFF	TPA-SBFF	7	5.92	-	2.53	-	-	1.51	-	-	4.1	-	-	0.14	0.166		
ITO/DNTPD/NPB/FP-SBFF:dopant/Alq3Alq3/LiF	FP-SBFF	TPA-SBFF	7	5.97	-	4.13	-	-	3.26	-	-	6.61	-	-	0.14	0.172		
ITO/MoO3/NPB/TCTA/TPA-PPI/TPBi/LiF/Al	-	TPA-PPI	100	5.22	-	5.02	3.76	3.13	6.13	2.7	-	5.66	4.25	3.5	0.15	0.11	2012	[33]
ITO/NPNPB/NPB/DMPPP:Dopant/BAlq/LiF/Al	DMPPP	PPIE	5	5.35	-	8.1	-	-	4.8	-	-	10	-	-	0.14	0.14	2012	[34]

ITO/NPNPB/NPB/DMPPP:Dopant/BAlq/LiF/Al	DMPPP		5	5.35	2.6	7.8	-	-	6.4	-	-	10.4	-	-	0.14	0.15		
ITO/NPNPB/NPB/DMPPP:Dopant/BAlq/LiF/Al	DMPPP	TPIE	5	5.47	-	7.7	-	-	3.1	-	-	7.9	-	-	0.15	0.11		
ITO/TAPC/BDNA/Bphen/LiF/Al	BDNA	-	100	5.59	-	5.26	-	1.9	-	-	-	-	-	-	0.159	0.072	2012	[35]
ITO/NPB/TcTa/BDNA:BCzVBi/Bphen/LiF/Al		BCzVBi	5	5.5	2.5	6.78	-	6.6	-	-	-	-	-	-	0.147	0.131		
ITO/NPB/BDPA/Bphen/LiF/Al	BDPA	-	100	5.58	2.1	3.55	-	1.51	-	-	-	-	-	-	0.153	0.06		
ITO/NPB/TcTa/BDPA:BCzVBi/Bphen/LiF/Al		BCzVBi	5	5.5	2.1	6.55	-	5.48	-	-	-	-	-	-	0.147	0.141		
ITO/PEDOT:PSS/NPB/TPA-AN/TPBI/LiF/Al	-	TPA-AN	100	5.18	-	3.59	-	-	4.02	-	-	4.54	-	-	0.152	0.189	2012	[36]

ITO/PEDOT:PSS/NPB/TPA-PA/TPBI/LiF/Al	-	TPA-PA	100	5.18	-	7.23	-	-	3.36	-	-	4.07	-	-	0.157	0.073		
ITO/MoO3/CBP/EML/TPBi/LiF/Al	CBP	Pht-1	4	6.14	-	2.12	-	-	-	-	-	-	-	-	0.16	0.09	2012	[37]
ITO/MoO3/CBP/EML/TPBi/Li/Al	CBP	Pht-2	4	6.11	-	1.9	-	-	-	-	-	-	-	-	0.16	0.12		
ITO/PEDOT:PSS/CRL-1/EML-1/CRL-2/EML-2/TPBi/LiF/Al	-	MDP3FL	100	-	-	-	5.7	4.5	-	3.6	1.9	-	3.8	3	0.143	0.085	2012	[38]
ITO/PEDOT:PSS/CRL-1/EML-1/CRL-2/EML-2/TPBi/LiF/Al	-		100	-	2.45	-	5.8	4.6	-	3.9	2.2	-	3.9	3.1	0.143	0.09		
ITO/NPNPB/NPB/DMPPP:Dopant/BAlq/LiF/Al	DMPPP	PPIE	5%	5.35	2.45	8.1	-	-	4.8	-	-	10	-	-	0.14	0.14	2012	[34]
ITO/MoO3/CBP/host:BCzVBi/Bepp2/LiF/Al	CBP	BCzVBi	6	5.4	3.05	4	-	-	-	-	-	-	-	-	0.16	0.17	2013	[39]

ITO/MoO3/CBP/host:BCzVBi/Bepp2/LiF/Al	$Bepp_2$	BCzVBi	50	5.4	3.09	6.1	-	-		-	-	-	-	-	0.18	0.31		
ITO/MoO3/NPB/TCTA/Be(PPI)2/TPBI/LiF/Al	-	$Be(PPI)_2$	100	5.8	2.7	2.82	2.55	2.4	2.52	1.6	0.95	2.41	2.18	2.04	0.15	0.09	2013	[40]
ITO/MoO3/NPB/TCTA/Zn(PPI)2/TPBI/LiF/Al	-	$Zn(PPI)_2$	100	5.8	2.7	2.08	1.32	0.75	2.02	0.75	0.26	2.06	1.31	0.74	0.15	0.09		
ITO/NPB/DPBF/Bphen/LiF/Al	-	DPBF	100	5.7	2.7	1	-	-	1	-	-	1.1	1.1	1	0.17	0.16	2013	[41]
ITO/NPB/TCTA/POTA/TPBI/LiF/Al	-	POTA	100	5.41	2.7	4.2		3.4	5		2.6	5.9	-	4.8	0.15	0.26	2013	[42]
ITO/MoO3/TcTa/EML/TPBi/LiF/Al	BCzVBi	Spiro-(3)-F	10	6.04	2.9	4.92	4.17	-	5.4	2.87	-	6.66	5.64	-	0.149	0.187	2013	[43]
ITO/m-MTDATA/α-NPD/host:BDAVBi/Alq3/LiF/Al	DPVBi	BDAVBi	3	5.3	3	5.4	-	-	-	-	-	-	-	-	0.15	0.22	2013	[44]
ITO/m-MTDATA/α-NPD/host:BDAVBi/Alq3/LiF/Al	Spiro-FA	BDAVBi	3	5.3	2.8	5	-	-	-	-	-	-	-	-	0.17	0.29		

ITO/m-MTDATA/α-NPD/host:BDAVBi/Alq3/LiF/Al	Spiro-FPA	BDAVBi	3	5.3	2.8	7.2	-	-	-	-	-	-	-	-	0.16	0.23		
ITO/m-MTDATA/α-NPD/host:BDAVBi/Alq3/LiF/Al	MADN	BDAVBi	3	5.3	2.5	7.4	-	-	-	-	-	-	-	-	0.15	0.22		
ITO/2-TNATA/NPB/EMLs/B3PYMPM/LiF/Al	-	TPAXAN	100	5.59	2.5	4.62	4.37	-	-	-	-	-	-	-	0.155	0.049	2013	[45]
ITO/2-TNATA/NPB/EMLs/B3PYMPM/LiF/Al	-	MADN	100	5.6	2.8	2.99	2.71	-	-	-	-	-	-	-	0.153	0.06		
ITO/NPB/DPDT-ICZ/$Bepp_2$/LiF/Al	-	DPDT-ICZ	100	4.96	3.1	0.96	0.71	0.4	1.58	0.82	0.28	1.31	1.02	0.56	0.15	0.08	2013	[46]
ITO/NPB/DNDT-ICZ/$Bepp_2$/LiF/Al	-	DNDT-ICZ	100	5	-	1.35	1.25	0.85	1.64	1.13	0.49	1.36	1.29	0.88	0.15	0.08		
ITO/PEDOT:PSS/NPB/TcTa/EML	CBP	BAn-(3,5)-CF_3	5	5.86	-	5.02	-	-	2.62	-	-	3.05	-	-	0.156	0.083	2013	[47]

/TPBi/CsC O_3/Al																		
PEDOT:PSS/TCTA/ CBP:1/TPBi/CsF/Al	CBP	N-BF-1	3	5.57	-	4.5	3.4	-	1	-	-	2.3	-	-	0.15	0.07	2013	[48]
ITO/NPB/TCTA/T1 B/TPBI/LiF/Al	-	T1B	100	5.23	-	3.05	-	-	-	-	-	5.12	-	-	0.15	0.08	2013	[48]
ITO/NPB/TCTA/T2 B/TPBI/LiF/Al	-	T2B	100	5.21	-	4.67	-	-	-	-	-	3.13	-	-	0.15	0.08		
ITO/NPB/EML/Bph en/LiF/Al	MADN	DSA-ph	5	5.4	2.9	-	-	-	8.7	-	-	-	-	4. 5	0.15	0.29	2013	[49]
ITO/MoO3/NPB/M ADN/Alq3/LiF/Al	MADN	nondoped	-	-	2.16	-	-	-	-	-	-	4.6	-	-	0.18	0.17	2014	[50]
ITO/MoO3/NPB/M ADN/BPhen/LiF/Al	MADN	nondoped	-	-	2.09	-	-	-	-	-	-	4.8	-	-	0.16	0.09		
ITO/NPB/1 or 2/Bph en/Liq/Al	9,10-Bis(4- (triphenyls ilyl) phenyl) anthracene	nondoped	-	-	-	2.05	-	-	2.92	-	-	3.26	-	-	0.18	0.18	2014	[51]

ITO/MoO3/NPB/TCTA/SBFBI/TPBi/LiF/Al	SBFBI	nondoped	-	-	2.91	1.81	-	-	1.56	-	-	2.63	-	-	0.18	0.2	2014	[52]
ITO/MoO3/NPB/TCTA/SAFBI/TPBi/LiF/Al	SAFBI	nondoped	-	-	3.07	2.23	-	-	1.34	-	-	1.96	-	-	0.15	0.1		
ITO/N,N′-di(1-naphthyl)-N,N′-bis[(4-diphenylamino)phenyl]-biphenyl-4,4′-diamie/N,N,N′,N′-tetra(1-biphenyl)-biphenyl-4,4′-diamine/ NA-MSBAF/LG201/LiF/Al	NA-MSBAF	nondoped	-	-	2.85	5.15	-	-		-	-	7.67	-	-	0.147	0.202	2014	[53]
ITO/NPNPB/NPB/DMPPP:6g/BAlq2/LiF/Al	DMPPP	3,6,9,12-Tetrakis(bis(4-tert-butylphenyl)amino)chrysene	5%	5.02	2.79	6.31 (3V)	-	-	3.21	-	-	7.2	-	-	0.13	0.2	2014	[54]

ITO/MoO3/NPB/EML/TPBi/LiF/Al	DTPBA	BCzVBi	-	5.4	-	8.4	-	-	2.92	-	-	6.5	-	-	0.147	0.125	2015	[55]
ITO/MoO3/NPB/EML/TPBi/LiF/Al	AND	BCzVBi	-	5.4	-	7.83	-	-	3.2	-	-	5.7	-	-	0.167	0.231		
ITO/MoO3/NPB/EML/TPBi/LiF/Al	DTPBA	nondoped	-	5.6	2.66	1.38	-	-	1.61	-	-	2.6	-	-	0.15	0.086		
ITO/MoO3/NPB/EML/TPBi/LiF/Al	AND	nondoped	-	5.8	2.4	1.57	-	-	0.72	-	-	1.7	-	-	0.155	0.075		
ITO/DNTPD/TBB/H3 hosts: D1 or D2/LG201/LiF/Al	1-(2-naphthyl)-10-(2-(6-phenyl)naphthyl)spiro[benzo[ij]tetraphene-7,9′-fluorene] (SBTF)	3,9-di(di(p-tolyl))amino(spiro[benzo[de]anthracene-7,9′-fluorene]	5%	5.31	2.4	5.16	-	-	5.6	-	-	8.91	-	-	0.144	0.261	2015	[56]
ITO/DNTPD/TBB/H3 hosts: D1 or D2/LG201/LiF/Al	1-(2-naphthyl)-10-(2-(6-phenyl)naphthyl)spiro	3,9-di(di-4-biphenyl)aminospiro[benzo[de]a	5%	5.42	2.4	4.83	-	-	4.63	-	-	7.37	-	-	0.139	0.217		

	[benzo[ij]tetraphene-7,9′-fluorene] (SBTF)	nthracene-7,9′-fluorene]																
ITO/DNTPD/TBB/H3 hosts: D1 or D2/LG201/Li/Al	1-(2-naphthyl)-10-(2-(6-phenyl)naphthyl)spiro[benzo[ij]tetraphene-7,9′-fluorene] (SBTF)	9,10-p-bis(p-N,N-diphenyl-aminostyryl)benzene (DSA-Ph)	5%	5.4	2.4	4.76	-	-	4.86	-	-	7.91	-	-	0.148	0.239		
ITO/2-TNATA/NPB/MADN: BD-1/TmPyPB/LiF/Al	MADN	BD-1	5%	5.2	-	-	-	-	3.8	-	-	4.69	-	-	0.15	0.17	2015	[57]
ITO/TMDI/NPB/MADN: BD-1/TmPyPB/LiF/Al	MADN	BD-1	5%	5.2	-	-	-	-	5.26	-	-	5.79	-	-	0.15	0.17		

ITO/DNTPD/NPB/MADN: BD-1/TmPyPB/LiF/Al	MADN	BD-1	5%	5.2	-	-	-	-	4.33	-	-	5.88	-	-	0.15	0.17		
ITO/HATCN/NPB/TCTA/NI/TPBI/LiF/Al	NI-1-TPA	nondoped	-	-	-	4.12	4.06	3.81	3.48	3.19	2.16	3.33	3.28	3.08	0.145	0.087	2015	[58]
ITO/HATCN/NPB/TCTA/NI/TPBI/LiF/Al	NI-2-TPA	nondoped	-	-	3	5.16	4.92	4.13	3.93	3.52	2.2	3.76	3.58	3.01	0.146	0.077		
ITO/HATCN/NPB/TCTA/NI/TPBI/LiF/Al	NI-1-PhTPA	nondoped	-	-	3	6.08	5.96	5.46	6.96	6.37	4.91	6.35	6.22	5.7	0.143	0.115		
ITO/HATCN/NPB/TCTA/NI/TPBI/LiF/Al	NI-2-PhTPA	nondoped	-	-	-	5.95	5.89	5.54	6.12	5.74	4.45	5.46	5.41	5.09	0.145	0.1		
ITO/NPB/EML 1–3/Alq3/Liq/Al	9-ethyl-3,6-bis(10-phenylanthracen-9-yl)-9H-carbazole	nondoped	-	-	-	1.36	-	-	0.92	-	-	2.28	-	-	0.2	0.22	2015	[59]

ITO/NPB/EML 1–3/Alq3/Liq/Al	2,8-bis(10-phenylanthracen-9-yl)dibenzo[b,d]furan	nondoped	-	-	5.55	1.46	-	-	1.38	-	-	2.06	-	-	0.18	0.17		
ITO/NPB/EML 1–3/Alq3/Liq/Al	2,8-bis(10-phenylanthracen-9-yl)dibenzo[b,d]thiophene	nondoped	-	-	5.53	1.23	-	-	0.84	-	-	1.39	-	-	0.16	0.13		
ITO/NPB/designed emitting materials (1)/Alq3/Liq/Al	N-phenyl-4-(10-phenylanthracen-9-yl)-N-(4-(10-phenylanthracen-9-yl)phenyl)benzenamine	nondoped	-	-	5.54	-	-	-	2.52	-	-	4.99	-	-	0.18	0.26	2015	[60]

ITO/NPB/TCTA/PhPC/TPBi/LiF/Al	PhPC	nondoped	-	-	5.5	3.6	-	-	1.1	-	-	1.8	-	-	0.15	0.05	2015	[61]

ITO/NPB/(9,9-Dimethyl-2-(10-(10-phenylanthracen-9-yl)anthracen-9-yl)-9H-fluoren-7-yl)triphenylsilane/Bphen/Liq/Al	(9,9-Dimethyl-2-(10-(10-phenylanthracen-9-yl)anthracen-9-yl)-9H-fluoren-7-yl)triphenylsilane	nondoped	-	-	2.4	1.82	-	-	2.82	-	-	2.76	-	-	0.18	0.16	2015	[62]
ITO/NPB/TCTA/BBTPI/TPBI/LiF/Al	BBTPI	nondoped	-	-	2.5	5.77	-	-	-	-	-	5.48	-	-	0.15	0.1		
ITO/NPB/TCTA/NBTPI/TPBI/LiF/Al	NBTPI	nondoped	-	-	2.77	5.73	-	-	-	-	-	5.43	-	-	0.15	0.11	2015	[63]
ITO/NPB/TCTA/2NBTPI/TPBI/LiF/Al	2NBTPI	nondoped	-	-	2.77	5.95	-	-	-	-	-	3.93	-	-	0.15	0.09		
ITO/TAPC/TCTA/CZN/TmPyPB/LiF/Al	CZN	nondoped	-	-	2.7	4.6	-	4.5	6.4	-	4.8	7.7	-	7.6	0.17	0.25	2015	[64]
ITO/NPB/TCTA/EML/TPBi/LiF/Al	$Si(PPI)_2$	$An(PPI)_2$	10%	5.6	2.7	6.1	4	1.9	8	3	0.9	-	-	-	0.18	0.17	2015	[65]

ITO/NPB/TCTA/BBTPI/TPBI/LiF/Al	BBTPI	nondoped	-	-	-	5.77	5.6	5.41	4.77	4.41	2.9	5.48	5.35	5.1	0.15	0.1	2015	[66]
ITO/NPB/TCTA/SOTPA/TPBI/LiF/Al	SOTPA	nondoped	-	-	2.92	4.6	-	3.9	5.3	-	2.8	5.4	-	4.5	0.14	0.14	2015	[67]
ITO/NPB/TCTA/Ph-BPA-BPI/TPBI/LiF/Al	Ph-BPA-BPI	nondoped	-	-	2.95	4.56	-	-	3.66	-	-	3.6	-	-	0.15	0.08	2016	[68]
ITO/NPB/TCTA/Py-BPA-BPI/TPBI/LiF/Al	Py-BPA-BPI	nondoped	-	-	2.94	5.64	-	-	10.5	-	-	10.9	-	-	0.17	0.29		
ITO/HAT-CN/TAPC/EML/TPBi/LiF/Al	Spiro-(3,5)-F	4,4'-bis(9-ethyl-3-carbazovinylene)-1,1'-biphenyl (BCzVBi)	10%	5.4	-	3.85	-	3.83	3.97	-	-	6.51	-	6.46	0.15	0.24	2016	[69]
ITO/NPB/Blue materials 1−3/Bphen/Liq/Al	3-(10-(naphthalen-7-yl)anthrace	nondoped	-	-	-	-	-	-	1.03	-	-	1.1	-	-	0.17	0.22	2016	[70]

	n-9-yl) quinoline																	
ITO/NPB/Blue materials 1−3/Bphen/Liq/Al	2-methyl-6-(10-(naphthalen-7-yl)anthracen-9-yl) quinoline	nondoped	-	-	-	-	-	-	1.04	-	-	1.3	-	-	0.17	0.22		
ITO/NPB/Blue materials 1−3/Bphen/Liq/Al	2-methyl-4-(10-(naphthalen-7-yl)anthracen-9-yl) quinoline	nondoped	-	-	-	-	-	-	1.42	-	-	1.67	-	-	0.15	0.18		
ITO/TAPC/TCTA/NAPT/B3PYMPM/LiF/Al	NAPT	nondoped	-	-	2.5	6	-	-	-	-	-	11.3	-	-	0.158	0.25	2016	[71]
ITO/TAPC/TCTA/NAPPT/B3PYMPM/LiF/Al	NAPPT	nondoped	-	-	2.5	4.7	-	-	-	-	-	6	-	-	0.149	0.142		

ITO/TAPC/TCTA/NAXPT/B3PYMPM/LiF/Al	NAXPT	nondoped	-	-	2.5	6.6	-	-	-	-	-	3.9	-	-	0.145	0.068		
ITO/PEDOT:PSS/NPB/EML/TPBi/Ca:Ag	2,5-BTPE-TP	nondoped	-	-	-	2.67	-	-	4.07	-	-	6.8	-	-	0.19	0.35	2016	[72]
ITO/NPB/CHDANT/TmPyPB/LiF/Al	CHDANT	nondoped	-	-	2.89	-	-	-	3.48	-	-	4.22	-	-	0.2	0.26	2017	[73]
ITO/NPB/SAF-BPI/TPBI/LiF/AL	SAF-BPI	nondoped	-	-	2.78	-	-	-	2.08	-	-	3.95	-	-	0.15	0.1	2017	[74]
ITO/HATCN/NPB/TCTA/PTHPI/TPBi/LiF/Al	PTHPI	nondoped	-	-	2.79	3.41	-	-	4	-	-	7.2	-	-	0.18	0.34	2017	[75]
ITO/NPB/TCTA/EML/TPBI/LiF/Al	DTPA-DSO2	nondoped	-	-	2.36	6.4	-	-	10.6	-	-	9.8	-	-	0.144	0.224	2017	[76]
ITO/NPB/blue emitter/Bphen/Liq/Al	N-(4-((E)-2-(9,9-diethyl-2-(1,4-diphenyltriphenylen-3-yl)-9H-	nondoped	-	5.65	2.42	2.13	-	-	1.31	-	-	2.82	-	-	0.18	0.25	2017	[77]

	fluoren-7-yl)vinyl) phenyl)-N-Phenylbenzenamine																	
ITO/2-TNATA/NPB/mADN:blue dopant 3/Alq3/Liq/Al	mADN	3,6-bis[4′-(1-di(tert-butyl))phenyl]-11,14-bis(diphenylamino) dibenzo[g,p]chrysene	3%	5.19	2.45	4.75	-	-	4.29	-	-	6.42	-	-	0.14	0.18	2017	[78]
ITO/MoO3/TCTA/DATPC)/TPBi/LiF/Al	DATPC-SCF3	nondoped	-	-	2.6	3.05	-	-	-	-	-	-	-	-	0.15	0.06	2017	[79]
ITO/MoO3/TCTA/DATPC/TPBi/LiF/Al	DATPC-OCF3	nondoped	-	-	2.6	2.7	-	-	-	-	-	-	-	-	0.14	0.09		
ITO/NPB/Cz-BPIS/TPBI/LiF/Al	Cz-BPIS	nondoped	-	-	2.54	2.61	-	-	1.63	-	-	1.91	-	-	0.16	0.09	2017	[80]

ITO/ NPB/TAPC/chrom I/ BCP/LiF/Al	4-[4-(2,2′:6′,2″-Terpyridinyl)]phenyltriphenylamine (chromophore I)	nondoped	-	-	2.56	-	-	-	-	-	-	3.6	-	-	0.19	0.22	2017	[81]
ITO/NPB/MPPIS-Cz/TPBi/LiF/Al	MPPIS-Cz	nondoped	-	-	2.55	1.48	-	-	1.3	-	-	1.52	-	-	0.16	0.08	2017	[82]
ITO/HAT-CN/TAPC/EML/Bphen/Liq/Al	MBAn-(4)-F	BBPA	5%	4.87	2.31	10.27	8.7	7.25	-	-	-	-	-	-	0.15	0.05	2018	[83]
ITO/HAT-CN/TAPC/EML/Bphen/Liq/Al	mCP	BBPA	5%	4.87	2.78	6.02	5.14	2.41	-	-	-		-	-	0.15	0.03		
ITO/m-MTDATA:NPB/DMPPP:BCzVBi/Bphen/Cs2CO3:Ag2O/Al	DMPPP	BCzVBi	8%	-	2.61	-	-	-	-	-	-	3.53	-	-	0.155	0.128	2018	[84]
ITO/MoO3/TCTA/CBP:CzPAF-	CBP	CzPAF-TFMP	5%	5.76	2.27	5.4	4.1	2.4	3.17	-	-	3.33	-	-	0.155	0.066	2018	[85]

TFMP/TmPyPB/LiF/Al																		
ITO/PEDOT:PSS/CzPAF-TFMP/TPBI/LiF/Al	CzPAF-TFMP	nondoped	-		2.66	4.6	4.5	3.4	3.99	-	-	5.19	-	-	0.167	0.143		
ITO/ReO3:mCP/mCP/emitter:mCP/TSPO1/Rb2CO3:TSPO1/Al	mCP	BDTPDDA	10%	5.45	2.66	8.5	-	4.6	8.4	-	2.3	-	-	-	0.142	0.116		
ITO/ReO3:mCP/mCP/emitter:mCP/TSPO1/Rb2CO3:TSPO1/Al	mCP	BDTPSAF	10%	5.46	2.66	8.5	-	4.8	10.3	-	3.3	-	-	-	0.139	0.189	2018	[86]
ITO/ReO3:mCP/mCP/emitter:mCP/TSPO1/Rb2CO3:TSPO1/Al	mCP	DTXSAF	10%	5.52	2.7	7.7	-	-	5.2	-	-	-	-	-	0.149	0.082		
ITO/HTM: MoOx/HTM/HOST: dopant/HOST/ETM/LiF/Al	-	BD-06	1%	5.48	2.7	-	-	11.4	-	-	-	-	-	9.15	0.141	0.09	2018	[87]

ITO/ HTM: MoOx/HTM/HOST: dopant/HOST/ETM/ LiF/Al	-	BD-05	1%	5.41	2.55	-	-	11.8	-	-	-	-	-	11.6	0.14	0.122		
ITO/NPB/Blue emitters 1 or 2/BPhen/Liq/Al	7-(1-(1-(2-(diphenylamino)-9,9-diethyl9H-fluoren-7-yl)naphthalen-4-yl)naphthalen-4-yl)-9,9-diethylN,N-diphenyl-9H-fluoren-2-amine	nondoped	-	-	2.4	2.3	-	2.12	1.19	-	1.02	2.79	-	2.7	0.14	0.12	2018	[88]
ITO/HATCN/TAPC/TCTA/PIAnCN/TPBI/LiF/Al	PIAnCN	nondoped	-	-	2.48	9.44	-	-	10.44	-	-	13.16	-	-	0.14	0.19	2018	[89]

ITO/2-TNATA/NPB/α,β-AND: synthesized material/Bphen/Liq/Al	α,β-ADN	7 -(4-(4-(N,N-diphenyl)styryl)phenyl)-spiro[9H-fluorene-9,5 -[5H]indeno[1,2-b]pyridine]	10%	5.45	2.4	4.49	-	-	8.18	-	-	9.93	-	-	0.18	0.3	2018	[90]
ITO/2-TNATA/NPB/Synthesized material/Bphen/Liq/Al	2,7,7,13,13-pentamethyl-9-(10-phenylanthracen-9-yl)7,13-dihydrobenzo [5,6]-s-indaceno[1,2-g]quinoline	nondoped	-	-	2.57	4.92	-	-	2.52	-	-	4.51	-	-	0.15	0.1	2018	[91]

	-		-	-	2.08	5.1	-	-	-	-	-	3.75	-	-	0.148	0.084	2018	[92]
ITO/PEDOT/TCTA/mPAC/TPBi/LiF/Al	mPAC	nondoped	-	-	2.93	6.76	-	-	3.48	-	-	5.61	-	-	0.16	0.09		
ITO/HAT-CN/HAT-CN:TAPC/7 or 8:TCTA/7or 8:26 DCzPPy/Tm3PyP26PyB/LiF/Al	TCTA/26 DCzPPy	7-(Diphenylamino)-4-(2,4,6-trimethoxyphenoxy)-2H-chromen-2-one	10%	5.51	2.86	5.1	-	-	-	-	-	3.75	-	-	0.148	0.084	2018	[93]
				-	-	-	-	-	0.79	-	-	1.14	-	-	0.161	0.046		
ITO/2-TNATA/NPB/α,β-ADN: synthesized material/TPBi/LiF/Al	α,β-ADN	p-TPA-AP-TPA	4%	5.66	-	-	6.51	8.09	-	-	-	-	6.93	8.8	0.14	0.15	2019	[94]
ITO/MoO3/TCTA/L-	L-BPPI	nondoped	-	5.5	2.84	-	-	-	2.89	-	-	3.82	-	-	0.16	0.1	2019	[95]

BPPI/TmPyPB/Liq/Al																		
ITO/HATCN/TAPC/BD4PP:dopants/TPBi/Liq/Al	BD4PP	BCzVB	5%	5.4	2.59	3.8	-	-	-	-	-	4.41	-	-	0.16	0.13	2019	[96]
ITO/HATCN/NPB/TCTA/N-BPyIA/CBP-15/TPBi/LiF/Al	CBP	N-BPyIA	15%	5.41	2.65	7.67	6.6	5.13	6.78	-	-	7.98	-	-	0.15	0.1	2019	[97]
ITO/HATCN/NPB/TCTA/N-BPyIA/TPBi/LiF/Al	N-BPyIA	nondoped	-	-	2.6	5.63	5.63	4.93	10.66	-	-	12.64	-	-	0.22	0.31		
ITO/HATCN/NPB/PCzAc/PhPC:BPPyA/p-bPPhenB/LiF/Al	PhPC	BPPyA	3%	5.7	2.6	9.36	-	9.18	6.6	-	5.73	7.84	-	7.72	0.135	0.115	2019	[98]
ITO/HATCN/NPB/CCP/PhPC:BPPyA/p-bPPhenB/LiF/Al	PhPC	BPPyA	3%	-	2.6	8.65	-	7.27	8.23	-	4.02	7.35	-	6.05	0.135	0.113		
ITO/TAPC/TCTA/mCP/CBP:TPP-PPI/TPBI/LiF/Al	CBP	TPP-PPI	20%	5.58	2.6	6.34	5.94	5.35	2.85	2.14	1.33	3.63	3.24	2.84	0.15	0.06	2019	[99]

ITO/MoO3/mCP:MoO3/mCP/Cz-Oxa-BPh/TPBi/LiF/Al	Cz-Oxa-BPh	nondoped	-	-	2.59	4	3.8	3.1	1.3	-	-	1.4	-	-	0.16	0.06	2019	[100]
ITO/TPD/ZnL/mCP/PBD/Ca/Al	mCP	zinc complex with N,N′-ethylamine-bis[1-phenyl-3-methyl-4-formylimino-2-pyrazoline-5-ol] ligand	20%	5.6	2.6	5	-	-	7.6	-	-	13.8	-	-	0.22	0.19	2019	[101]
ITO/HATCN/TAPC/TCTA/EML/TPBi/LiF/Al	PIAnTPE	nondoped	-	-	2.1	4.46	4.06	4.42	5	-	-	6.9	-	-	0.16	0.23	2019	[102]
ITO/HAT-CN/TAPC/TBBA/TPBi/Liq/Al	TBBA	nondoped		-	2.14	3.2	-	-	2.7	-	-	2.52	-	-	0.15	0.06	2019	[103]

ITO/HAT-CN/TAPC/BAFs:DSA-ph/TPBi/Liq/Al	MBA	DSA-ph	3%	-	2.78	9.47	-	-	16.57	-	-	16.54	-	-	0.15	0.26		
ITO/NPB/Blue emitter/Bphen/Liq/Al	(6-(4-(10-phenylanthracen-9-yl)styryl)-N,N-diphenylnaphthalen-2-amine)	nondoped	-	-	2.6	3.41	3.39	-	2.41	1.99	-	5.79	5.69	-	0.19	0.31	2019	[104]
ITO/HAT-CN/TAPC/TCTA/Host:BCz–BFPz/TmPyPB/Liq/Al	mCBP	BCz–BFPz	3%	5.58	1.6	4.34	-	-	-	-	-	2	-	-	0.15	0.05	2019	[105]
ITO/PEDOT:PSS/NPB/CzB-MOPPI/TPBi/LiF/Al	CzB-MOPPI	nondoped	-	-	1.58	5.97	-	-	1.32	-	-	3.34	-	-	0.16	0.08	2019	[106]
ITO/2-TNATA/NPB/α,β-ADN: synthesized	α,β-ADN	4-(6-(10-(4-(diphenylamino)phen	4%	5.66	2.7	8.38 (5000 nits)	-	-	-	-	-	9.14(5000nits)	-	-	0.14	0.15	2019	[94]

material/TPBi/LiF/Al		yl)anthracen-9-yl)pyren-1-yl)-N,N-diphenylaniline (p-TPA-AP-TPA)																
ITO/NPB/TCTA/2FPPICz/TPBi/LiF/Al	2FPPICz	nondoped	-	-	-	4.3	-	2.5	1.7	-	0.7	1.8	-	1.1	0.16	0.045	2020	[107]
	2FPPIPCz	nondoped	-	-	-	5.6	-	5.4	4.5	-	3.5	4.8	-	4.6	0.15	0.075		
ITO/BPBPA:HATCN/BPBPA/TNPA/PANB:emitter/TNPT/ZADN/LiF/Al	PANB	N6,N6,N12,N12-tetraphenylbenzo[b]indolo[1,2,3-lm]carbazole-6,12-diamine (NIDPA-1)	2%	5.55	-	4.9	-	-	-	-	-	7.1	-	-	0.13	0.22	2020	[108]

ITO/MoO3/TAPC/TCTA/CBP:IP-DPPI/TPBI/LiF/Al	CBP	IP-DPPI	40%	5.48	-	6.22	-	5.83	3.81	-	1.8	4.42	-	4.07	0.15	0.094	2020	[109]
ITO/NPB/PyEtCz/TPBI/LiF/Al	PyEtCz	nondoped	-	-	-	3.35	-	-	1.91	-	-	3.24	-	-	0.15	0.1	2020	[110]
ITO/HATCN/TAPC/TCTA/TPAATPE/TPBi/LiF/Al	TPAATPE	nondoped	-	-	-	6.97	-	5.96	8.8	-	4.8	9.4	-	7.7	0.15	0.16	2020	[111]
ITO/MoO3/TAPC/TCTA/CBP:TPA-DFCP/TmPyPB/LiF/Al	CBP	TPA-DFCP	5%	-	2.29	8.3	-	-	4.87	-	-	5.63	-	-	0.153	0.077	2020	[112]

Table S2. A comprehensive list of wet-processed blue fluorescent OLED devices, including the publication year, device composition, key dopant, doping concentration, employed host, HOMO, LUMO, EQE, PE, CE, CIE (X), CIE (Y), and reference. The years span from 2006 to 2020

**Device Structure**	**Host**	**Emitter**				**EQE (%)**			**PE (lm/W)**			**CE (cd/A)**			**CIE**		**Year**	**Ref.**
		**Name**		**HOMO**	**LUMO**	**Max**	**100**	**1000**	**Max**	**100**	**1000**	**Max**	**100**	**1000**	**(X)**	**(Y)**		

			**Conc. (wt%)**				**(cdm^{-2})**			**(cdm^{-2})**			**(cdm^{-2})**					
ITO/PEDOT:PSS/blue material:PVK/BCP/Alq3/LiF/Al	PVK	PP-C	2	4.9	2.06	1.6	-	-	-	-	-	2.2	-	-	0.16	0.2	2005	[113]
ITO/PEDOT:PSS/T3/Ba/ Al	-	TAT-T3	100	-	-	2	-	-	-	-	-	2.07	-	-	0.15	0.09	2006	[114]
ITO/PEDOT:PSS/C1/Ba/Al	-	C1	100	5.19	2.27	0.242	0.22	0.2	-	-	-	0.174	0.16	0.14	0.17	0.14	2008	
ITO/PEDOT:PSS/C2/Ba/Al	-	C2	100	5.28	2.23	1.36	1.24	1.36	-	-	-	0.976	0.89	0.98	0.16	0.14		[115]
ITO/PEDOT:PSS/C3/Ba/Al	-	C3	100	5.27	2.15	2.16	1.46	2.06	-	-	-	1.56	1.06	1.48	0.16	0.15		
ITO/PEDOT:PSS/PVK/G0/Ba/Al	-	G0	100	5.8	-	4.2	-	3.6	1.7	-	1.2	3.3	-	2.9	0.155	0.09	2009	
ITO/PEDOT:PSS/PVK/G0/Ba/Al	-	G0	100	5.8	-	4.5	-	3.7	1.8	-	1.3	3.6	-	3	0.155	0.087		[116]
ITO/PEDOT:PSS/PVK/G0/Ba/Al	-	G0	100	5.8	-	6.6	-	6.2	3	-	2.4	5.3	-	4.9	0.155	0.086		

ITO/PEDOT:PSS/TCDqC/Ba/Al	-	TCDqC	100	5.4	2.74	-	-	-	0.4	-	-	0.7	-	-	0.15	0.1	2010	[117]
ITO/PEDOT:PSS/TCDqC/TPBi/LiF/Al	-	TCDqC	100	5.4	2.74	-	-	-	1.7	-	-	3.7	-	-	0.15	0.15		
ITO/PEDOT:PSS/EML/TPBi/LiF/Al	-	FBA-1	100	5.3	2.4	-	-	-	1.08	-	-	1.96	-	-	0.15	0.13	2011	
ITO/PEDOT:PSS/EML/TPBi/LiF/Al	-	FBA-2	100	5.46	2.45	-	-	-	0.19	-	-	0.38	-	-	0.16	0.1		[118]
ITO/PEDOT:PSS/EML/TPBi/LiF/Al	-	FBA-3	100	5.45	2.46	-	-	-	0.05	-	-	0.1	-	-	0.19	0.22		
ITO/PEDOT:PSS/EML/TPBi/LiF/Al	-	Blu1	100	4.8	1.36	2	0.53	-	-	0.89	-	1.83	0.49	-	0.151	0.097	2011	
ITO/PEDOT:PSS/EML/TPBi/LiF/Al	-	Blu2	100	-	-	5.8	4.6	-	-	6.42	-	10.76	8.5	-	0.159	0.303		
ITO/PEDOT:PSS/EML/TPBi/LiF/Al	-	Blu2	100	-	-	6.1	4.8	-	-	7.23	-	11.2	8.8	-	0.148	0.28		[119]
ITO/PEDOT:PSS/EML/TPBi/LiF/Al	-	Blu2	100	-	-	7.4	5.16	-	-	8.55	-	13.27	9.24	-	0.149	0.292		
ITO/PEDOT:PSS/EML/TPBi/LiF/Al	PVK	Blu2	2	-	-	2.71	1.33	-	-	0.7	-	2.64	1.29	-	0.147	0.135		

ITO/PEDOT:PSS/EML/TPBi/LiF/Al	CBP	Blu2	6	-	-	9.4	5.11	-	-	4.29	-	10.08	5.48	-	0.147	0.139		
ITO/PEDOT:PSS/ T1/TPBi/LiF/Al	-	TAB-OF (T1)	100	5.53	2.58	-	-	-	0.74	-	-	1.13	-	-	0.151	0.121	2011	
ITO/PEDOT:PSS/ T2 /TPBi/LiF/Al	-	TAB-OF (T2)	100	5.55	2.61	-	-	-	1.35	-	-	1.8	-	-	0.149	0.098		[120]
ITO/PEDOT:PSS/T3/TPBi/LiF/Al	-	TAB-OF (T3)	100	5.57	2.64	-	-	-	0.93	-	-	1.39	-	-	0.149	0.101		
ITO/PEDOT:PSS/polymers/TPBi/LiF/Al	-	PF-33F	100	5.57	2.45	1.14	-	-	-	-	-	1.14	-	-	0.16	0.13	2012	[121]
ITO/PEDOT:PSS/T1/TPBi/CsF/Al	-	T1	100	5.15	2.22	1.82	-	-	0.98	-	-	1.63	-	-	0.19	0.11	2012	
ITO/PEDOT:PSS/T2/TPBi/CsF/Al	-	T2	100	5.14	2.29	2.3	-	-	1.23	-	-	1.96	-	-	0.16	0.11		[122]
ITO/PEDOT:PSS/T3/TPBi/CsF/Al	-	T3	100	5.15	2.32	4.19	-	-	3.16	-	-	3.83	-	-	0.16	0.09		

ITO/PEDOT:PSS/PCAN/Bphen/LiF/Al	-	PCAN	100	5.49	2.51	4.61	-	-	1.41	-	-	3.64	-	-	0.15	0.08	2012	[123]
ITO/PEDOT:PSS/EML/TPBi/LiF/Al	-	PO1	100	5.26	2.3	1.79	1.7	1.77	1.11	1.08	0.91	1.78	1.69	1.76	0.15	0.09	2012	[124]
ITO/PEDOT:PSS/EML/TPBi/LiF/Al	-	PO2	100	5.24	2.28	1.54	1.5	1.5	1.11	1.06	0.86	1.84	1.79	1.79	0.15	0.12		
ITO/PEDOT:PSS/EML/TPBi/LiF/Al	-	PO3	100	5.14	2.21	1.66	1.66	1.61	1.19	1.1	0.86	1.72	1.72	1.67	0.15	0.1		
ITO/PEDOT:PSS/EML/TPBi/LiF/Al	-	PO4	100	5.11	2.23	2.06	2.01	1.87	1.86	1.55	1.18	2.36	2.32	2.15	0.15	0.11		
ITO/PEDOT:PSS/EML/BCP/LiF:Al	-	TCF	100	5.19	1.96	-	-	-	-	-	-	0.93	-	-	0.16	0.09	2012	[125]
ITO/PEDOT:PSS/EML/TPBi/LiF/Al	-	PA-1	100	5.55	2.5	-	-	-	-	-	-	0.02	-	-	0.19	0.241	2012	[126]
ITO/PEDOT:PSS/EML/TPBi/LiF/Al	CBP	PA-1	1	5.55	2.5	-	3.3	2.3	-	0.9	0.5	-	1.3	1	0.157	0.053		
ITO/PEDOT:PSS/EML/TPBi/LiF/Al	CBP	PA-1	3	-	-	-	3.5	2.5	-	1.2	0.6	-	1.9	1.4	0.155	0.063		
ITO/PEDOT:PSS/EML/TPBi/LiF/Al	Spiro-2CBP	PA-1	3	5.55	2.5	-	3.1	2.4	-	1.3	0.6	-	1.6	1.2	0.157	0.064		

ITO/PEDOT:PSS/EML/TPBi/LiF/Al	SimCP2	PA-1	3	5.55	2.5	-	2.9	1.4	-	1.3	0.4	-	2	0.9	0.164	0.087		
ITO/PEDOT:PSS/EML/TPBi/LiF/Al	TCTA	PA-1	5	5.55	2.5	-	2.9	1.9	-	3	1.2	-	4.1	2.4	0.166	0.184		
ITO/PEDOT:PSS/T1/TPBi/Ba/Al	-	T1	100	-	-	6.1	6.1	-	-	-	-	4.9	4.9	-	0.17	0.08	2013	[127]
ITO/PEDOT:PSS/T2/TPBi/Ba/Al	-	T2	100	-	-	6.7	4.6	-	-	-	-	5.3	3.5	-	0.16	0.08		
ITO/PEDOT:PSS/T3/TPBi/Ba/Al	-	T3	100	-	-	6.8	6.3	-	-	-	-	5.4	5.1	-	0.16	0.07		
	-	D1	100	-	-	1.7	-	-	-	-	-	0.9	-	-	0.17	0.09	2013	60_Ma_JPCC_2013
	-	D2	100	-	-	2.6	-	-	-	-	-	2.8	-	-	0.17	0.22		
	-	P2	100	-	-	1.2	-	-	-	-	-	1.2	-	-	0.21	0.19		
	PVK+PBD	P2	10	-	-	0.7	-	-	-	-	-	0.3	-	-	0.17	0.08		
ITO/PEDOT:PSS/EML/TPBi/CsF/Al	-	PPO-BF2	100	5.52	2.4	1.74	-	-	-	-	-	1.08	-	-	0.17	0.11	2013	[128]

ITO/PEDOT:PSS/EML/TPBi/CsF/Al	-	PPO-BF3	100	5.45	2.47	3.17	-	-	-	-	-	1.76	-	-	0.16	0.08		
ITO/PEDOT:PSS/EML/TPBi/CsF/Al	-	PPO-TF2	100	5.56	2.46	1.82	-	-	-	-	-	1.13	-	-	0.17	0.1		
ITO/PEDOT:PSS/EML/TPBi/CsF/Al	-	PPO-TF3	100	5.48	2.5	3.39	-	-	-	-	-	1.88	-	-	0.16	0.09		
ITO/PEDOT:PSS/PFO–P/Ba/Al	-	PFO-P	100	5.8	2.2	0.31	-	-	-	-	-	0.07	-	-	0.18	0.18	2013	[129]
ITO/PEDOT:PSS/PFO/Ba/Al	-	PFO	100	5.6	2.2	0.56	-	-	-	-	-	0.2	-	-	0.16	0.11		
ITO/PEDOT:PSS/PFO–TFP/Ba/Al	-	PFO-TFP	100	6.2	2.4	0.54	-	-	-	-	-	0.12	-	-	0.17	0.06		
ITO/PEDOT:PSS/PVK/PFO–TFP/TPBI/Ba/Al	-	PFO-TFP	100	-	-	5.02	-	-	-	-	-	1.07	-	-	0.16	0.05		
ITO/PEDOT:PSS/EML/BPhen/LiF/Al	CBP	PBOF-O	1	5.91	3.26	2.4	-	-	1.7	-	-	3.4	-	-	0.14	0.13	2013	[130]
ITO/PEDOT:PSS/EML/BPhen/LiF/Al	PVK		4	5.91	3.26	1.9	-	-	1.1	-	-	2.5	-	-	0.14	0.14		
ITO/PEDOT:PSS/EML/BPhen/LiF/Al	CBP		0.5	5.88	3.23	1.5	-	-	0.77	-	-	1.6	-	-	0.15	0.12		

ITO/PEDOT:PSS/EML/BPhen/LiF/Al	PVK	PBOF-DMO	0.5	5.88	3.23	1.2	-	-	0.57	-	-	1.2	-	-	0.15	0.11		
ITO/PEDOT:PSS/EML/TPBI/LiF/Al	-	4c	100	5.5	2.5	-	0.55	0.69	-	0.9	0.8	-	1.2	1.5	0.188	0.33	2013	[131]
ITO/PEDOT:PSS/EML/TPBI/LiF/Al	CBP		3	5.5	2.5	-	2.24	2.12	-	1.2	0.8	-	2.4	2.1	0.156	0.126		
ITO/PEDOT:PSS/EML/TPBI/LiF/Al	-	5b	100	5.33	2.2	-	0.71	0.76	-	0.7	0.5	-	0.9	0.9	0.176	0.16		
ITO/PEDOT:PSS/EML/TPBI/LiF/Al	CBP		3	5.33	2.2	-	1.53	-	-	0.2	-	-	0.4	-	0.158	0.045		
ITO/PEDOT:PSS/EML/TPBI/LiF/Al	-	5c	100	5.4	2.1	-	0.47	-	-	0.5	-	-	0.7	-	0.185	0.232		
ITO/PEDOT:PSS/EML/TPBI/LiF/Al	CBP		3	5.4	2.1	-	0.194	-	-	0.1	-	-	0.3	-	0.163	0.041		
ITO/PEDOT:PSS/poly-TPD/XL-PHNSF-E50/Ca/Al	-	PHNSF-E50	100	5.7	2.7	3.2	-	-	1.62	-	-	-	-	-	0.204	0.232	2014	[132]
ITO/PEDOT:PSS/NPB/M1/TPBi/LiF/Al	(2-(4-(9H-carbazol-9-yl)phenyl)	nondoped		-	-	1.94	-	-	0.48	-	-	0.65	-	-	0.165	0.05	2014	[133]

	-1-phenyl-1H-phenanthro[9,10-d]imidazole (M1)																
ITO/PEDOT:PSS/NPB/M2/TPBi/LiF/Al	1-phenyl-2-(4-(3-(1-phenyl-1H-phenanthro[9,10-d]imidazol-2-yl)-9H-carbazol-9-yl)phenyl)-1H-phenanthro[9,10-d]imidazole (M2)	nondoped	-	-	3.02	-	-	0.86	-	-	1.53	-	-	0.167	0.056		

ITO/PEDOT/NPB/M3/TPBi/LiF/Al	1-phenyl-2-(9-phenyl-9H-carbazol-3-yl)-1H-phenanthro[9,10-d]imidazole (M3)	nondoped	-	-	1.51	-	-	0.32	-	-	0.57	-	-	0.165	0.053		
ITO/PEDOT/NPB/M4/TPBi/LiF/Al	2,2′-(9-phenyl-9H-carbazole-3,6-diyl)bis(1-phenyl-1H-phenanthro[9,10-d]imidazole) (M4)	nondoped	-	-	2.11	-	-	0.76	-	-	0.125	-	-	0.169	0.078		
ITO/PEDOT/NPB/M5/TPBi/LiF/Al	2-(4′-(9H-carbazol-	nondoped	-	-	1.46	-	-	0.3	-	-	0.88	-	-	0.17	0.077		

	9-yl)-[1,1′-biphenyl]-4-yl)-1-phenyl-1H-phenanthro[9,10-d]imidazole (M5)																
ITO/PEDOT:PSS/NPB/TCTA/TBPMCN/TPBi/LiF/Al	TBPMCN	nondoped	-	-	7.8	-	-	5.5	-	-	10.5	-	-	0.16	0.16	2015	[134]
ITO/TDMS/TPBi/Ca/Al	TDMS	nondoped	-	-	1.19	-	-	0.23	-	-	1.06	-	-	0.156	0.1	2015	[135]
ITO/TDPS/TPBi/Ca/Al	TDPS	nondoped	-	-	1.36	-	-	0.38	-	-	1.62	-	-	0.175	0.144		
ITO/PEDOT:PSS/EML/TPBi/Ca:Ag	CAC	nondoped	-	-	1.53	1.26	1.37	0.77	0.69	0.61	1.63	1.35	1.46	0.16	0.1	2015	[136]
ITO/PEDOT:PSS/EML/TPBi/Ca:Ag	BAC	nondoped	-	-	0.47	0.46	0.31	0.27	0.27	0.13	0.61	0.6	0.4	0.18	0.16		

ITO/PEDOT:PSS/PVK:OXD-7 (7:3):EML/TPBi)/Ca:Ag	PVK:OXD-7 (7:3)	CAC	15%	-	-	2.81	2.78	2.05	1.64	1.39	0.71	3.03	3.01	2.22	0.16	0.1		
ITO/PEDOT:PSS/NPB/OCADN/TPBi/LiF/Al	OCADN	nondoped		-	-	-	-	-	1.13	-	-	2.25	-	-	0.16	0.3	2015	[137]
ITO/PEDOT:PSS/NPB/OC4PADN/TPBi/LiF/Al	OC4PADN	nondoped		-	-	-	-	-	0.48	-	-	0.99	-	-	0.16	0.14		
ITO/PEDOT:PSS/I or II or III or IV/TPBI/LiF/Al	2-(4-(9,9-dimethyl-10-phenyl-9,10-dihydroacridin-2-yl)phenyl)-1-phenyl-1H-phenanthro[9,10-d]imidazole	nondoped		-	-	5.12	-	-	2.49	-	-	4.6	-	-	0.15	0.09	2016	[138]

ITO/PEDOT:PSS/I or II or III or IV/TPBI/LiF/Al	2-(3-(9,9-dimethyl-10-phenyl-9,10-dihydroacridin-2-yl)phenyl)-1-phenyl-1H-phenanthro[9,10-d]imidazole	nondoped	-	-	2.87	-	-	0.7	-	-	1.33	-	-	0.17	0.09		
ITO/PEDOT:PSS/I or II or III or IV/TPBI/LiF/Al	9,9-dimethyl-10-phenyl-2-(4-(1,4,5-triphenyl-1H-imidazol-2-yl)phenyl)-9,10-	nondoped	-	-	3.56	-	-	1.33	-	-	3.36	-	-	0.14	0.13		

	dihydroacridine																	
ITO/PEDOT:PSS/I or II or III or IV/TPBI/LiF/Al	9,9-dimethyl-10-phenyl-2-(3-(1,4,5-triphenyl-1H-imidazol-2-yl)phenyl)-9,10-dihydroacridine	nondoped		-	-	1.96	-	-	0.5	-	-	1.45	-	-	0.16	0.08		
ITO/ PEDOT/ EML/ TPBi/ LiF/Al	CBP	JV234	3%	5.03	2.35	-	4.1	2.8	-	1.3	0.7	-	2	1.4	0.15	0.06	2017	[139]
ITO/PEDOT:PSS/mCBP:JV55/TPBi/ LiF/Al	mCBP	JV55	3%	5.7	2.7	6.5	5.5	2.7	-	1.7	0.7	-	2.6	1.4	0.16	0.06	2018	[140]
ITO/PEDOT:PSS/MADN/MADN: DSA-Ph/BPhen/LiF/Al	MADN	DSA-Ph	3%	5.4	2.7	10	-	-	13	-	-	14.7	-	-	0.159	0.281	2018	[141]

ITO/PEDOT:PSS/TAPC/TCTA/ mcp:Oxd-bCz/TmPyPB/LiF/Al	mcp	Oxd-bCz	6%	5.95	2.69	5.6	-	-	1.9	-	-	2.4	-	-	0.16	0.07	2018	[142]
ITO/Wox/NPB/EML/Bphen/LiF/ Al	MADN	1-4-di-[4-(N,N-diphenyl)amino]styryl-benzene		-	-	7.2	-	-	6.4	-	-	10.8	-	-	0.167	0.283	2018	[143]
ITO/Wox/NPB/EML/Bphen/LiF/ Al	MADN	nondoped		-	-	4.6	-	-	2.5	-	-	3.2	-	-	0.155	0.101		
ITO/NPB/TCTA/mCP/Bepp2: dopant/TPBi/ Liq/ Al	Bepp2	DPAc-OXD	6%	5.69	2.56	1.84	-	-	0.99	-	-	1.3	-	-	0.16	0.12	2018	[144]
ITO/PEDOT:PSS/5,9-CSBF/TPBi/LiF/Al (150 nm)	5,9-CSBF	nondoped		-	-	4.1	-	-	-	-	-	4.42	-	-	0.15	0.091	2020	[145]
ITO/PEDOT:PSS/2,7-CSBF/TPBi/LiF/Al	2,7-CSBF	nondoped		-	-	2.49	-	-	-	-	-	1.23	-	-	0.155	0.05		
ITO/PEDOT:PSS/emission layer/DPEPO/TmPyPB/Liq/Al	CzPA-F-PD	nondoped		-	-	4.61	-	-	3.97	-	-	6.32	-	-	0.17	0.18	2020	[146]
ITO/PEDOT:PSS/emission layer/DPEPO/TmPyPB/Liq/Al	CzPA-F-PD	TFA		-	-	4.67	-	-	3.92	-	-	7.11	-	-	0.25	0.26	2020	

			CzPA-F-PD:TFA (5000/1, v-v)															
				-	-	4.07	-	-	3.65	-	-	6.97	-	-	0.36	0.37		
ITO/PEDOT:PSS/EML/DPEPO/TmPyPB/Liq/Al	FBTO-EHFF	nondoped		-	-	3.66	0.78	-	3.66	-	-	6.37	-	-	0.2	0.24	2020	[147]

Table S3. A comprehensive list of dry-processed blue phosphorescence OLED devices, including the publication year, device composition, key dopant, doping concentration, employed host, HOMO, LUMO, EQE, PE, CE, CIE (X), CIE (Y), and reference. The years span from 2001 to 2020

Device Structure	Host	Emitter				EQE (%)			PE (lm/W)			CE (cd/A)			CIE		Year	Ref.
		Name	Conc. (wt%)	HOMO	LUMO	Max	100	1000	Max	100	1000	Max	100	1000	(X)	(Y)		

							**(cdm^{-2})**			**(cdm^{-2})**			**(cdm^{-2})**					
ITO/CuPc/a-NPD/CBP:FIrpic/BAlq/LiF/Al	CBP	FIrpic	6	5.8	3.1	5.7	-	-	6.3	-	-	-	-	-	0.16	0.29	2001	[148]
ITO/NPD/mCP/UGH2:FIr6/BCP/LiF/Al	UGH2	FIr6	10	6.1	3.1	11.6	-	-	13.9	-	-	-	-	-	0.16	0.26	2003	[149]
ITO/PEDOT:PSS/CDBP:Firpic/Balq/LiF/Al	CDBP	FIrpic	3	5.8	3.1	10.4	-	-	10.5	-	-	20.4	-	-	0.17	0.34	2003	[150]
ITO/NPB/DCB:FIrpic/DCB:FIrpic/DCB:FIrpic/DCB:FIrpic/Bphen/Mg: Ag.	DCB	FIrpic	20	5.8	3.1	-	-	-	-	-	-	15.4	-	-	-	-	2004	[151]
ITO/NPB/DCB:FIrpic/BPhen/Mg:Ag.	DCB	FIrpic	8	5.8	3.1	5.8	-	-	-	-	-	9.8	-	-	0.17	0.34	20	[152]

																	04	
-	UGH2	f-Irpmb3	-	-	-	5.9	-	-	1.7	-	-	-	-	-	0.17	0.06	2005	[153]
-	-	-	-	-	-	11	-	-	-	-	-	21	-	-	0.16	0.29	2006	[154]
-	-	-	-	-	-	14	-	-	-	-	-	32	-	-	0.17	0.38	2006	[154]
ITO/PEDOT:PSS/PVK/26mCPy:OXD-7:Pt-4/BCP/CsF/Al	26mCPy:OXD-7	:Pt-4	2	-	-	-	12.6	-	-	6.4	-	20.6	-	-	0.15	0.26	2008	[155]
-	-	(dfbmb)2Ir(fptz	-	-	-	6	2.7	-	4	0.9	-	6.3	3	-	0.16	0.13	2008	[156]

ITO/MeO-TPD:F4-TCNQ/TAPC/mCP:FIr6 /UGH2:FIr6/Bphen/Bphen:Li/LiF/Al	mCP	FIr6	4	6.1	3.1	17	-	-	25	25	20	35	-	-	0.16	0.28	2008	[157]
ITO/CuPC/NPD/Firpic:TCTEB/BAlq/LiF/Al	TCTEB	FIrpic	8	5.8	3.1	8.5	-	-	11.3	-	-	13.7	-	-	0.17	0.36	2008	[158]
ITO/HTL / mCP / mCP/UGH2:FIr6 / BCP / BCP / LiF / LiF / Al / Al	UGH2	FIr6	10	6.1	3.1	18	-	-	16	-	-	-	-	-	0.16	0.27	2008	[159]
TO/PEDOT/TAPC/mCP:FIrpic/BCP/LiF/Al	mCP	FIrpic	-	5.8	3.1	-	-	-	50	-	-	60	59	57	0.19	0.2	2009	[160]
ITO/DNTPD/NPB/mCP/PPO2:FCNIr/BCP/Alq3/LiF/Al	PPO2	FCNIr	15%	5.8	3	18.4	13.5	11.8	16.6	9.1	-	21.1	16.5	-	0.14	0.15	2009	[161]

ITO/BCP/Alq3/LiF/Al	mCP	FCNIr	-	5.8	3	9.2	-	-	-	-	-	11	-	-	0.15	0.16	2009	[162]
ITO/TAPC/Pt(ptp)2Pt(ptp)2:HM-A1/PO-15/LiF/Al	-	-	-	-	-	16.7	-	16	42.8	-	30.7	-	-	-	0.16	0.3	2009	[163]
ITO/NPB/host:FIrpic/TPBI/LiF/Al	DPSiCBP	FIrpic	8	5.8	3.1	3.5	-	-	4.5	-	-	6.5	-	-	0.16	0.29	2009	[164]
ITO/Al/Me2SiCBP2:FIr6/TAZ/LiF/Al	Me2SiCBP2	Fir6	16	6.1	3.1	15.75	-	-	18.6	-	-	26.7	-	-	0.15	0.24	2009	[165]

ITO/NPB/mCP/mCP:FIrpic/BAlq3/LiF/Al	mCP	FIrpic	9	5.8	3.1	10.6	-	-	12.8	-	-	-	-	-	0.16	0.28	2009	[166]
ITO/BPAF/EML/TAZ/LiF/Al	POAPF(5.26,2.40)	FIrpic	7	5.8	3.1	20.6	20.2	18.8	36.7	33.8	24.3	35.4	34.8	32.3	0.13	0.27	2009	[167]
ITO/MeO-TPD:F4-TCNQ/TAPC/mCP:FIr6/GH2:FIr6/3TPYMB/CsEIL/LiF/Al	mCP:UGH2: 2:1	FIrpic	4:25	5.8	3.1	20	-	-	36	-	-	41	-	-	0.16	0.28	2009	[168]
ITO/BPAF/FIrpic:PCF/TAZ/LiF/Al	PCF(5.86,2.76)	FIrpic	28	5.8	3.1	13	-	-	26.2	-	-	30.8	-	-	0.13	0.35	2009	[169]
ITO/MoO3/FIrpic:BCBP/BCP/Alq3/LiF/Al	BCBP	FIrpic	7	5.8	3.1	4	-	-	6.3	-	-	9	-	-	0.11	0.34	2009	[170]

ITO/TAPC/EML/Tm3PyPB/Tm3PyPB:Cs/LiF/Al	SPPO1	FIr6	10	-	-	20.1	-	-	29.2	-	-	37.8	-	-	0.16	0.32	2009	[171]
ITO/TAPC/Ir(dbfmi):PO9/PO9/B3PyPB/LiF/Al	PO9	Ir(dbfmi)	10	-	-	18.6	13.3	6.2	35.9	19.6	6.3	28.6	20.5	9.4	0.15	0.19	2010	[172]
ITO/TAPC/EML/Tm3PyPB/Tm3PyPB:Cs/LiF/Al	mCP:UGH3 = 5:5	FIr6	10	6.1	3.1	22.9	-	-	39.2	27.9	17.2	39.5	-	-	0.16	0.29	2010	[173]
ITO/MoO3/NPB/1/TPBI/LiF/Al		N,N,N’,N’-tetraphenyl-5’-(1-phenyl1H-benzimidazol-2-yl)-1,1’:3’,1’’-terphenyl-4,4’’-diamine		5.2	2.06	2.9	-	-	1.6	-	-	2.2	-	-	0.17	0.07	2010	[174]
ITO/NPB/TAPC/Me2Si(CBP)2/Me2Si(CBP)2:FIr6/SiTAZ/LiF/Al	Me2Si(CBP)2	FIr6	15	6.1	3.1	15.5	-	13.8	-	-	-	-	-	-	0.16	0.22	2010	[175]

-	CP2	Ir(dbfmi)	8	-	-	-	-	-	7.6	4.9	2.3	13.4	11.4	6.7	0.15	0.18	2011	[176]
ITO/NPB/mCP/UGH3:FIr6/UGH3/Bphen/LiF/Al	mCP	FIr6	10	6.1	3.1	14.8	-	-	15.4	-	-	22.8	-	-	0.15	0.23	2011	[177]
ITO/PEDOT:PSS/MoO3-doped DPBIC/DPBIC/1: Ir(dbfmi)/1)/Cs2CO3-doped BCP/Cs2CO3/Al	DPBIC	*meta*-CBP	-	5.71	1.77	-	8.7	6.1	-	10.2	6	-	12.2	8.5	0.16	0.18	2011	[178]
ITO/TPD/TCTA/2: BSB-CzSi/BSB/TPBI/LiF/Al	BSB-CzSi	[Ir(mpmi)2(pypz)]	-	5.03	1.95	15.2	-	-	19.6	-	-	28.1	-	-	0.14	0.27	2011	[179]
ITO/TAPC/*t*-CzSA/BCPO:(fpmi)2Ir(dmpypz)/3TPYMB/BPhen/LiF/Al	BCPO	(fpmi)2Ir(dmpypz)	8	5.2	2	17.1	16.5	15.1	19.8	15	11.2	22.3	21.5	19.6	0.13	0.16	2011	[180]

ITO/PEDOT:PSS/NPB/BIPPA or BCPPA/mCPPO1:FCNIrpic/TSPO1/LiF/Al	mCPPO1	FCNIrpic	3	5.72	2.98	24.7	24.4	23.9	30	19.8	13.9	30.7	-	27.6	0.14	0.18	2011	[181]
ITO/DNTPD/NPB/mCP/mCPPO1:FCNIrpic/TSPO1/LiF/Al	mCPPO1	FCNIrpic	-	5.72	2.98	25.1	25.1	24	29.8	23.1	17.2	-	31	28.9	0.14	0.18	2011	[182]
ITO/DNTPD/NPB/mCP/DFCzPO:FCNIrpic/TSPO1/LiF/Al	DFCzPO	FCNIrpic	3	5.72	2.98	21.4	-	17.5	22.6	-	10	26.4	-	21.5	0.14	0.18	2011	[183]
ITO/PEDOT:PSS/TAPC/FIr6:Ad-CzPd/TmPyPB/LiF/Al	Ad-CzPd	FIr6	-	6.1	3.1	13	-	-	15	-	-	-	-	-	0.16	0.26	2011	[184]
ITO/TPD/TCTA/3:UGH2-CzSi/UGH2/TPBI/LiF/Al	CzSi:UGH2; 2:1	FIrpic	4	5.8	3.1	14.1	-	-	13.8	-	-	1.7	-	-	0.14	0.18	2011	[179]

ITO/NPB/mCP/FIrpic:mCP/Firpic:UGH2/BPhen/Liq/Al	mCP:UGH2: 2:1	FIrpic	8:20	5.8	3.1	11.44	-	-	16.8	-	-	23.14	-	-	0.17	0.33	2011	[185]
ITO/MoO3/NPB/TCTA/EML/TPBI)/LiF/Al	*m*-BISiPCz, 2	FIrpic	8	5.41	1.78	11.4	-	-	19.8	-	-	29.3	-	-	0.18	0.4	2011	[186]
ITO/PEDOT/DTAF/host:dopant/TPBI/LiF/Al	mCPNBI (5.8,2.36)	FIrpic	8	-	-	16.3	-	-	23.3	-	-	35.7	-	-	0.17	0.37	2011	[187]
ITO/DNTPD/NPB/mCP/DBT2:FCNIrpic/TSPO1/LiF/Al	DBT2 (6.09,2.57)	FCNIrpic	15	5.72	2.98	20.2	-	17.9	28.9	-	-	28.9	-	-	0.14	0.21	2011	[188]
ITO/DNTPD/NPB/FPCC or FPCA/mCPPO1:FCNIrpic/TSPO1/LiF/Al	*m*CPPO1	FCNIrpic	3	5.72	2.98	22	-	19.7	25.2	-	13	26	-	-	0.14	0.17	2011	[189]

ITO/NPB/mCP/Host:Firpic/TPBI/LiF/Al	Bim-4	Firpic	10	5.8	3.1	-	-	14.1	15	-	9.7	22	-	20.3	0.17	0.29	2012	[190]
ITO/NPB:MoO3/NPB/DOPPP/POAPF/POAPF:Firpic/BmPyPB/LiF/Mg:Ag	POAPF	Firpic	-	5.8	3.1	-	-	-	20.3	-	13.3	23.5	-	15.5	0.16	0.32	2012	[191]
ITO/graphene/TAPC/HAT-CN/TAPC/HAT-CN/TAPCTCTA:FIrpic/DCzPPy:FIrpic/BmPyPB/LiF/Al	-	FIrpic	-	5.8	3.1	-	-	15.6	-	-	24.1	-	-	-	0.15	0.34	2012	[192]
ITO/Plexcore OC/MoO3:DPBIC/DPBIC/4:SimCP/SimCP/P3PyPB/CsF/Al	SimCP	Ir(dfpybpy)2(fpbpz)	-	-	-	7	-	-	4.14	-	-	-	-	-	0.16	0.19	2012	[193]
ITO/DNTPD/NPB/mCP/FCNIrpic/TSPO1/LiF/Al	bis-CMPC	FCNIrpic	10	5.72	2.98	13.3	13.1	8.8	-	-	-	-	-	-	0.14	0.21	2012	[194]

ITO/DNTPD/NPB/mCP/DFPO3:FCNIrpic/TSPO1/LiF/Al	DFPO3	FCNIrpic	10	5.72	2.98	25.9	23.2	-	-	-	-	-	-	-	0.14	0.22	2012	[195]
ITO/TAPC or DCDPA/DCDPA:DBFPO:FCNIrpic/TSPO1/LiF/Al	DCDPA:DBFPO	FCNIrpic	3	5.72	2.98	26.4	24.8	15	-	-	-	34	-	29.5	0.14	0.19	2012	[196]
ITO/DNTPD/NPB/mCP/BDDS:mCP:FCNIrpic/BDDS/LiF/Al	BDDS	FCNIrpic	10	5.72	2.98	18.1	14.5	12.2	-	-	-	-	-	-	0.14	0.19	2012	[197]
ITO/DNTPD/NPB/mCP/BCPCB or CPBDC:FCNIrpic/TSPO1/LiF/Al	PEDOT–CPBDC	FCNIrpic	10	5.72	2.98	24.5	22.5	19.4	31	21	13.4	-	-	-	0.14	0.19	2012	[198]
ITO/NPB/TAPC/Host/Host:FIr6/SiTAZ/LiF/Al	*p*-Cb	FIr6	10	6.1	3.1	15.3	-	-	-	-	-	25.3	-	-	0.15	0.24	2012	[199]

ITO/HATCN/NPB/TAPC/B2tCz:FIrpic/TmPyPB/LiF/Al	B2tCz	FIrpic	10	5.54	2.25	24.6	23.2	-	34	29.5	-	43.3	40.8	-	0.14	0.31	2012	[200]
ITO/MoO3/NPB/TCTAPO/TCTAPO:FIrpic/TmPyPB/LiF/Al	TCTAPO	FIrpic	7	5.8	3.1	17.8	17	15.6	40.7	28.4	18.5	35.6	33.9	31	0.14	0.34	2012	[201]
ITO/NPB/SimCP:FIrpic/TPBI/LiF/Al	SimCP	FIrpic	7	5.8	3.1	12.3	-	-	9.3	-	-	-	-	-	0.15	0.28	2012	[202]
ITO/NPB/DTASi/EML/Bphen/n-ETL/Al	New Host	FIrpic	10	5.8	3.1	12	-	12	17	-	17	25.3	-	25.3	0.16	0.34	2012	[203]
ITO/NPB/mCP/Bim-4:Firpic/TPBI/LiF/Al	Bim-4 (5.8,2.4)	FIrpic	10	5.8	3.1	14.1	-	-	15	-	9.7	22	-	20.3	0.17	0.29	2012	[190]

ITO/MoO3/NPB/mCP/1,8-mBICz:FIrpic/TmPyPB/LiF/Al	1,8-mBICz (5.58,2.31)	FIrpic	6	5.8	3.1	11.6	-	-	27	-	-	27.6	-	-	0.15	0.31	2012	[204]
ITO/HATCN/NPB/TAPC/B2tCz:FIrpic/TmPyPB/LiF/Al	B2tCz (5.54,2.25)	FIrpic	10	5.8	3.1	24.6	23.2	-	34	29.5	-	43.3	40.8	-	0.13	0.32	2012	[200]
ITO/Plexcore OC/MoO3:DPBIC/DPBIC/4:SimCP/SimCP/P3PyPB/CsF/Al	CDBP	Ir(III) compound	20	5.95	2.8	13.5	12.6	9.9	23.9	-	-	22.2	-	-	0.15	0.24	2013	[205]
-	mCPPO1	(TF)2Ir(pic)	10	5.9	2.9	17.1	16.8	-	19	15.2	-	21.7	21.3	-	0.14	0.16	2013	[206]
ITO/TAPC/FK306:mCP/B3PyPB/LiF/Al	mCP	FK306	17	-	-	17	15.3	12.7	30.2	24.2	16.1	29	26.1	21.5	0.16	0.25	2013	[207]

ITO/MoO3/mCP:MoO3/mCP/DPEPO:Ir(fdpt)$_3$/DPEPO/TPBi/LiF/Al	DPEPO	Ir(fdpt)3	10	5.27	2.41	22.5	20.4	18.5	24.7	-	-	-	-	-	0.14	0.18	2013	[208]
ITO/HATCN/NPD/TAPC/tON7:26 mCPy/DPPS/LiF/Al	26 mCPy	PtON7	6	-	-	23.3	20.4	16.8	-	20.6	11.7	-	-	-	0.15	0.13	2013	[209]
ITO/PEDOT:PSS/TAPC/3DCPO:FCNIrpic/TSPO1/LiF/Al	3DCPO	FCNIrpic	3	5.72	2.98	18.8	-	13.9	18.1	11	-	22.6	-	-	0.14	0.18	2013	[210]
ITO/PEDOT:PSS/NPB/BIPIA/mCPPO1:FCNIrpic/TSPO1/LiF/Al	mCPPO1	FCNIrpic	3	5.72	2.98	19.3	19	17.4	-	14.7	9.8		-	-	0.14	0.17	2013	[211]
ITO/PEDOT:PSS/TAPC/mCP/CbOTCb:Firpic/TSPO1/LiF/Al	CbOTCb	FIrpic	-	5.8	3.1	28.8	28.6	26.7	44	-	-	47.4	-	-	0.14	0.3	2013	[212]

ITO/TAPC/Firpic:pBCb2Cz/TmPyPB/LiF/Al	pBCb2Cz	FIrpic	6	5.8	3.1	23	-	22	34.6		28.4	38.3	-	36.7	0.15	0.31	2013	[213]
ITO/HAT-CN/TAPC/host:FIrpic/TmPyPB/Liq/Al	SSTF	FIrpic	-	5.8	3.1	18.7	-	-	41.5	41.3	32.9	44	44	41.9	0.16	0.36	2013	[214]
ITO/HAT-CN/TAPC/Host: FIrpic/TmPyPB/Liq/Al	-	FIrpic	8	5.8	3.1	21	-	18	23	-		43.9	-	37.7	0.15	0.35	2013	[215]
-	-	-	-	-	-	27.1	-	24.6	52.3		28.2	48.2		46.1	0.16	0.34	2013	[216]
ITO/NPB/TCTA/EML/TAZ/LiF/Al	SiBSTPA	FIrpic	8	5.8	3.1	10.2	-	-	15.6	8.3	5.2	21.4	15.6	12.8	0.16	0.31	2013	[217]

ITO/DNTPD/NPB/mCP/33DCTPFIrpic/TSPO1/LiF/Al	33DCTP	FIrpic	3	5.8	3.1	23.7	-	-	-	-	-	-	-	-	0.14	0.28	2013	[218]
ITO/MoO3/NPB/mCP/Host:FIrpic/TmPyPB/LiF/Al	mCPmPO	FIrpic	6	5.8	3.1	20.3	-	-	39.7	-	-	40	-	-	0.14	0.32	2013	[219]
ITO/MoO3/TAPC/TCTA:dopants/Tm/LiF/Al	TCTA	4-Cl-FIrpic	-	5.76	2.19	16	15.6	12.5	41	34	20	39	38	30	0.18	0.4	2013	[220]
ITO/TAPC/mCP/mCP:2 or 4/UGH2:2 or 4/UGH2/TmPyPB/LiF/Al	mCP	[Ir(b5bpm)2(fppz)]	6	5.8	2.09	17.9	15.8	-	35.8	21.6	-	38	33.6	-	0.19	0.328	2013	[220]
-	MBPTRZ	FIrpic	5	5.8	3.1	7	-	5.5	6.3	-	3.7	16.3	-	12.9	0.16	0.18	2013	[221]

ITO/TAPC/host:FIrpic/TmPyPB/LiF/Al	TATA (5.66,2.4)	FIrpic	6	5.8	3.1	25.8	-	25.7	45.4	-	41.5	46.2	-	46.1	0.16	0.343	2013	[216]
ITO/HAT–CN/TAPC/POSTF:Firpic/TmPyPB/Liq/Al	POSTF	FIrpic	15	5.8	3.1	26.8	-	-	50.5	-	-	53.9	-	-	0.14	0.33	2014	[222]
ITO/m-MTDATA/(Ir(ppz)3/23'ODSFX:FIrpic/BPhen/LiF/Al	23ˊODSFX	FIrpic	10	5.8	3.1	11.9	11.5	7	19.5	18.5	8.1	21.7	21.1	12.9	0.15	0.28	2014	[223]
ITO/MoO3/TAPC/EML/TmPyPB/LiF/Al	NSF-NSF (5.31,1.81)	FIrpic	10	5.8	3.1	19.1	17.7	16.2	42	32	22	41	37.8	34.5	0.15	0.33	2014	[224]
ITO/HAT-CN/TAPC/Host:FIrpic/TmPyPB/Liq/Al	mCP	FIrpic	8	5.8	3.1	14.6	-	-	24.4	-	17.4	36	-	34.6	0.16	0.38	2014	[225]

ITO/MoO3/TAPC/POBPmDPA:FIr6/TmPyPB/LiF/Al	POBPmDPA	FIr6	10	6.1	3.1	18.1	-	-	34	-	-	34	-	-	0.15	0.25	2014	[226]
ITO/MoO3/TAPC/POPCPA:FIr6/TP3PO/LiF/Al.	POPCPA (5.24,1.84)	FIr6	10	6.1	3.1	20.8	-	-	37.5	-	-	45.3	-	-	0.15	0.26	2014	[227]
ITO/TAPC/mCP:Pt complex/DPEPO:Pt complex/ DPEPO/TmPyPB/LiF/Al	DPEPO	Pt(II) complex 5	8	-	-	15.3	12.8	-	38	21.1	-	36.3	30.4	-	0.19	0.39	2015	[228]
ITO/TAPC/SiDMAC/(dfpypy)2Ir(acac):host material/BPSPF/B3PyPB/Liq/Al	SiDMAC:BPSPF	[(dfpypy)2Ir(acac)	16	-	-	-	17	15.9	-	31.8	24.7	-	30.8	28.8	0.15	0.27	2015	[229]
ITO/PEDOT:PSS/CBP:C3FLA-2/TPBi/LiF/Al	CBP	C3FLA-2	1	-	-	8	-	-	-	-	-	-	-	-	0.156	0.048	2015	[230]

ITO/HAT-CN/TAPC/PBCz-PO:FIrpic/TmPyPB/Liq/Al	PBCz-PO	FIrpic	5	5.8	3.1	13.4	-	-	31	-	-	31.5	-	-	0.16	0.4	2015	[231]
ITO/NOB/mCP/CbzTAZ:Firpic/Taz/LiF/Al	CbzTAZ	FIrpic	9	5.8	3.1	21.78	-	-	39.8	-	-	46.06	-	-	0.14	0.35	2015	[232]
-	CzBPDCb (6.23,2.73)	FIrpic	3	5.8	3.1	25.3	-	24.4	36.4	-	22.5	-	-	-	0.14	0.28	2015	[233]
ITO/MoO3/SQTPA/mCP/SPPO1:FIrpic/SPPO1/LiF/Al	SQTPA	FIrpic	10	5.8	3.1	17.5	14.4	12.3	32.5	22.6	14.6	41.5	34.2	29.4	0.18	0.4	2015	[234]
ITO/TAPC/mCP:Pt complex/DPEPO:Pt complex/ DPEPO/ TmPyPB/LiF/Al	mCP/DPEPO	Pt II complex	5	-	-	15.3	12.8	-	38	21.1	-	36.3	30.4	-	0.19	0.39	2015	[228]

ITO/MoO3/NPB/*m*CP/BBDC, BBDP, BSBDC and BSBDP-FIrpic/TmPyPB/LiF/Al	BSBDP (5.89,2.55)	FIrpic	7	5.8	3.1	18.7	-	-	34.4	-	30.3	34.2	-	30.1	0.15	0.3	2015	[235]
ITO/HATCN/NPB/TCTA/EML/TPBI/LiF/Al	-	TPA-SO2	-	-	-	3.91	-	-	-	5.26	3.68	5.46	5.35	4.92	0.154	0.168	2015	[236]
ITO/NPB/blue materials (1–5)/Bphen/Liq/Al		9-(2-Naphthalenyl)-10-[6-(triphenylsilyl)-2-naphthalenyl]-anthracene		5.71	2.63	2.64	-	-	2.43	-	-	2.71	-	-	0.16	0.1	2015	[237]
-	*o,o*´-NPh_2	-	-	-	-	15.3	-	12.3	31.4	-	18.9	34.5	-	27.5	0.17	0.37	2016	[238]
-	mCBP	Ir(itpim)3	15	5.53	-	-	-	20.1	-	-	25.3	-	-	36.3	0.16	0.28	2016	[239]

ITO/MoO3/TAPC/mCP/mCPCN:Ir(III) dopants/3TPYMB/LiF/Al	mCPCN	[Ir(pimf)	12	5.48	2.72	31.4	30.8	28.4	108.7	94.7	65.6	110.8	108.5	100.2	0.34	0.6	2016	[240]
ITO/MoO3/NPB/*m*CP/CzDPOPB:FIrpic/TmPyPB/LiF/Al	CzDPOPB	FIrpic	6	5.8	3.1	16.5	-	-	29.1	-	14.7	32.2	-	23.7	0.15	0.31	2016	[241]
ITO/MoO3/TAPC/TcTa/(dfppy)2Ir(dpp):26DCzPPy/TmPyPB/LiF/Al	26DCzPPy	DPP-2	10	5.96	2.95	28.3	-	-	52.74	46.34	33.31	58.78	54.24	48.79	0.13	0.37	2016	[242]
ITO/ReO3:mCP/mCP/EML/BM-A10/Rb2CO3:BM-A10/Al	mCP:BM-A10	FCNIr	12	-	-	24	23	21	41	37	26	-	-		0.15	0.21	2017	[243]
ITO/PEDOT:PSS/TAPC/host:Ir dopant/TmPyPB/LiF/Al	-	FIrpic	6	5.8	3.1	27	-	23.6	46.5	-	-	51.9	-	44	0.14	0.31	2017	[244]

ITO/MoO3/TAPC/*m*CP/*m*CP:ETM:FIrpic/EBL/TmPyPB/LiF/Al	*m*CP:*i*TPBIPy	FIrpic	8	5.8	3.1	19.3	-	-	36.9	-	-	38.5	-	-	0.15	0.29	2017	[245]
ITO/MoO3/TAPC/mCP/mCPCN:Ir complex/3TPYMB/LiF/Al	mCPCN	MS 19	8	5.76	3.08	31.3	31.1	29.7	51.4	51.4	31.2	56.4	56.4	53.7	0.15	0.3	2017	[246]
ITO/HAT-CN/TAPC/*f*-Ir-(dbfmi)$_3$:TSPO1/TSPO1/TmPyPB/Liq/Al	TSPO1	*f*-Ir-(dbfmi)$_3$	10	-	-	18.5	-	-	19.4	-	-	18.6	-	-	0.14	0.11	2017	[247]
ITO/NPB/mCP:iridium complex/BCP/Alq3/LIF/Al	mCP	Ir2	-	5.7	2.02	2.7	-	-	-	-	-	-	-	-	0.18	0.3	2017	[248]
ITO/HATCN/TAPC/DP4CzDBPSi:FIrpic/TmPyPB/LiF/Al	DP4CzDBPSi (2.5,6)	FIrpic	6	5.8	3.1	20.9	-	7.3	41.58	22.19	-	-	-	-	0.14	0.33	2017	[249]

	CzDBTCN	Ir(dbi)3	5	5.29	2.52	26.9	-	12.6	-	-	-	28.3	-	21.1	0.15	0.24	2018	[250]
ITO/HAT-CN/TAPC/Host: FIrpic/TmPyPB/Liq/Al	PO1TPA	FIrpic	15	5.8	3.1	13.8	-	8.6	27.6	-	14.7	31	-	20.7	0.16	0.33	2018	[251]
ITO/PEDOT:PSS/TAPC/TCTA/ host:FIrpic/TmPyPB/LiF/Al	*m*-TzCCz	FIrpic	6	5.8	3.1	11	-	-	28	-	-	22	-	-	0.16	0.33	2018	[252]
ITO/ReO3:mCP/mCP/Ir1:DPEPO/DPEPO/TSPO1/LiF/Al	DPEPO	Ir2	20	5.57	1.39	-	-	20	-	-	27.1	-	-	67	0.28	0.62	2018	[253]
ITO/TAPC/1/DPEPO:2/TPBi/LiF/Al	DPEPO	2	10	5.23	0.17	13.4	-	-	-	-	-	-	-	-	0.15	0.05	2018	[254]

ITO/MoO3/mCP:MoO3/mCP/DPEPO:Ir(fdpt)3/DPEPO/TPBi/LiF/Al	DPEPO	Ir(fdpt)3	10	5.27	2.41	22.5	16.5	11.1	-	-	-	23	-	-	0.15	0.11	2018	[255]
ITO/MoO3/TAPC/mCP/Ir(III) complex:PP021/TmPyPb/LiF/Al	PP021(2.68,6.21)	D3	8	5.86	2.88	27.9	-	-	44.9	-	-	58.6	-	50.6	0.16	0.33	2018	[256]
ITO/PEDOT:PSS/TAPC/TCTA/host:FIrpic/TmPyPB/LiF/Al	PyTzSCz (5.54,2.63)	FIrpic	4	5.8	3.1	26.4	-	20.4	48.1	-	-	45.9	-	35.6	0.14	0.27	2018	[257]
ITO/HAT-CN/TAPC/TCTA/SPBI-TPA:blue phosphor/TmPyPB/LiF/Al	SPBI-TPA (5.40,5.29)	FIrpic	10	5.8	3.1	25.5	-	-	-	-	-	55.1	-	-	0.17	0.34	2018	[258]
ITO/HATCN/TAPC/mCP/Ir(dfppy)2phpzpy:PPO21/TmPyPB/LiF/Al	mCP	Ir(dfppy)2phpzpy	10	5.44	2.8	29.3	-	24.4	49.85	-	28.4	58.74	-	48.84	0.18	0.34	2018	[259]

ITO/HAT-CN/TAPC/TCTA/TCzSi:FIrpic/TmPyPB/Liq/Al	TCzSi	FIrpic	20	5.35	1.8	13.32	-	-	18.15	-	-	25.5	-	-	0.159	0.31	2018	[260]
ITO/HAT-CN/TAPC/TCTA/mCP:Ir(III) complex/TmPyPB/Liq/Al	mCP	*Ir(dfmppm)(taz)*	15	6.06	3.42	14.5	-	-	19.5	-	-	28	-	-	0.15	0.3	2018	[261]
ITO/PEDOT:PSS/Emitter/TPBi/LiF/Al	-		-	5.72	1.86	-	-	-	0.35	-	-	0.133	-	-	0.18	0.21	2019	[262]
ITO/TAPC/mCP/dopant:DPEPO/DPEPO/TmPyPB/LiF/Al	DPEPO	Cz-5	10	-	-	18.7	13.7	-	30.1	21.8	-	25.9	13.5	-	0.145	0.218	2019	[263]
ITO/2-TNATA/NPB/EML/TPBi/Liq/Al	MADN	Pyrene-CN	10	-	-	-	-	-	3.62	-	-	5.45	-	-	0.142	0.102	2019	[264]

ITO/HAT-CN/TAPC/TCTA/mCBP/mCBP:PO-T2T:Dopants/PO-T2T/Liq/Al	mCBP:PO-T2T	FIrpic	10	5.8	3.1	25.2	-	24.3	59.9	-	39	50.3	-	48.4	0.147	0.328	2019	[265]
ITO/HATCN/BPBPA/TAPC/26mCPy:dopant/DPEPO/TmPyPB/LiF/Al	26mCPy	Pt(2-*ptz*)	8	5.27	2.34	14.7	14.6	12.3	24.2	-	-	30.8	-	-	0.17	0.31	2019	[266]
ITO/HAT-CN/TAPC/TCTA/$TCZSO_2$:FIrpic/TmPyPB/Liq/Al	$TCZSO_2$	FIrpic	15			22.8	-	21.6	40.3	-	34	47.8	-	47.7	0.15	0.35	2019	[267]
ITO/PEDOT:PSS/TAPC/mCP/exciplex host: blue phoshporsecent dopant/TSPO1/TPBi/LiF/Al	DPEPO :CDBP:PO-T2T	FCNIr				25.51	-	23.62	44.13	-	28.33	50.65	-	46.13	0.16	0.3	2019	[268]
ITO/HATCN/NPD/TAPC/PtNON or TBPe:26 mCPy/DPPS/BmPyPB/LiF/Al	mCBP	PtNON	6	-	-	12.6	-	11.6	-	-	-	-	-	-	0.17	0.38	2019	[269]

ITO/HATCN/NPD/TAPC/PtNON or TBPe:26 mCPy/DPPS/BmPyPB/LiF/Al	mCBP	PtNON	-	-	-	17.8	-	16.8	-	-		-	-	-	0.19	0.41	2019	
ITO/HATCN/NPD/TAPC/PtNON or TBPe:26 mCPy/DPPS/BmPyPB/LiF/Al	mCBP	PtNON	-	-	-	17.4	-	17.4	-	-		-	-	-	0.19	0.41	2019	
ITO/HATCN/NPD/TAPC/PtNON or TBPe:26 mCPy/DPPS/BmPyPB/LiF/Al	mCBP	PtNON	-	-	-	17.6	-	16.2	-	-		-	-	-	0.16	0.28	2019	
ITO/HATCN/NPD/TAPC/PtNON or TBPe:26 mCPy/DPPS/BmPyPB/LiF/Al	mCBP	PtNON	-	-	-	16.9	-	15.2	-	-		-	-	-	0.16	0.25	2019	
ITO/HATCN/NPD/TAPC/PtNON or TBPe:26 mCPy/DPPS/BmPyPB/LiF/Al	mCBP	PtNON	-	-	-	18	-	17.1	-	-	-	-	-	-	0.16	0.29	2019	

ITO/PEDOT:PSS/CzSi:Pt complex/DPEPO/*m*-TmPyPB/LiF/Al	CzSi	Pt complex	30	-	-	9.8	-	4.5	10.5	-	2.3	16.7	-	7.3	0.18	0.23	2019	[270]
ITO/BPBPA:HATCN/BABPA/PCZAC/emitting layer/DBFTRZ/ZADN/LiF/Al	O-39BCzCN	CN-Ir	5	6.02	2.83	5.5	-	5.4	-	-	-	6.3	-	4.7	0.15	0.23	2019	
ITO/BPBPA:HATCN/BABPA/PCZAC/emitting layer/DBFTRZ/ZADN/LiF/Al	M-39BCzCN	CN-Ir	5	6.02	2.82	5.9	-	5.2	-	-	-	9.1	-	5.1	0.15	0.23	2019	[271]
ITO/BPBPA:HATCN/BABPA/PCZAC/emitting layer/DBFTRZ/ZADN/LiF/Al	P-39BCzCN	CN-Ir	5	6.01	2.82	9.7	-	9.4	-	-	-	14.5	-	11.1	0.15	0.24	2019	
-	-	-	-	-	-	2.47	-	-	2.35	-	-	1.42	-	-	0.153	0.121	2019	[272]

ITO/MoO3/TAPC/TcTa/Firpic:p2PCB2CZ/TmPyPB/LiF/Mg:Ag	p2PCB2CZ	FIrpic	10	5.8	3.1	19.2	-	-	35.2	-	-	36.9	-	-	0.15	0.29	2019	[273]
ITO/BPBPA:HATCN/BPBPA/PCZAC/EML/DBFTrz/ZADN/LiF/Al	mCBP	CNImIr	5	5.79	2.79	12.1	-	5.7	20.2	-	9.6	-	-	-	0.15	0.24	2019	[274]
ITO/BPBPA:HATCN/BPBPA/PCZAC/EML/DBFTrz/ZADN/LiF/Al	mCBP	iPrImIr	5	5.03	2.36	17.1	-	12.6	28.3	-	21.1	-	-	-	0.15	0.24	2019	
ITO/BPBPA:HATCN/BPBPA/PCZAC/EML/DBFTrz/ZADN/LiF/Al	mCBP	DBFTrz:CNImIr	50 wt%:5 wt%	5.79	2.79	14.7	-	12.3	27.1	-	23.3	-	-	-	0.15	0.28	2019	
ITO/BPBPA:HATCN/BPBPA/PCZAC/EML/DBFTrz/ZADN/LiF/Al	mCBP	mCBP:CNImIr(15 nm:5 wt%)/mCBP:iPrImIr(15 nm:5 wt%)		-	-	16.1	-	13.8	26.6	-	23.3	-	-	-	0.15	0.24	2019	

ITO/PEDOT:PSS/CzSi:Pt(ppzOczpy-4m)/DPEPO/TmPyPB/Liq/Al	CzSi	Pt(ppzOczpy-4m)	30	5.3	2.1	19.5	-	9	16.7	-	4.1	26.6	-	12.1	0.15	0.16	2019	[275]
ITO/HAT-CN/TAPC/TCTA/SAIP:FIrpic/B4PyMPM/Liq/Al	SAIP	FIrpic	10	5.8	3.1	19.2	-	-	36.9	-	-	35.2	-	-	0.15	0.29	2019	[276]
ITO/PEDOT:PSS/NPB/TSPO1:*mer*-Ir1/TSPO1/TPBi/LiF/Al	TSPO1	*mer*-Ir1	40	5.22	2.08	24.8	16.6	13.1	16.9	9.1	5.5	20.9	14.1	11.2	0.14	0.08	2020	[277]
ITO/TAPC/TCTA/mCP/EML/3TPYMB/LiF/Al	DPEPO	Flu-4	-	-	-	22.3	14.9	10	-	-	-	24.6	22.8	16	0.17	0.19	2020	[278]
ITO/TAPC/TCTA/DPEPO:dopant/DPEPO/TPBi/LiF/Al	DPEPO	-	15	-	-	14.1	10.2	-	-	-	-	-	-	-	0.16	0.13	2020	[279]

-	-	CN-TPB-TPA	-	-	-	7.27	-	6.26	5.35	-	2.92	5.59	-	5.03	0.15	0.08	2020	[280]
ITO/BPBPA:HAT-CN/BPBPA/PCZAC or mCBP/EML/DBFTrz/ZADN:LiQ/LiQ/Al	-	-	-	-	-	27.6	-	-	-	-	-	-	-	-	0.12	0.13	2020	[281]
ITO/HATCN/TAPC/TCTA/Host:Dopant/TmPyPb/LiF/Al	4CzMC	FIrpic	-	5.8	3.1	18.9	-	-	38.8	-	-	37.1	-	-	0.16	0.31	2020	[282]
ITO/PEDOT:PSS/TAPC/mCP/Ir3:PPF/TmPyPB/LiF/Al	PPF	Ir3	7	5.8	2.8	21.23	18.73	-	28.68	-	-	36.53	32.23		0.15	0.26	2020	[283]
ITO/MoO3/mCP:MoO3/mCP/DPEPO:complexes/DPEPO/TPBi/LiF/Al	DPEPO	Ir3	13	5.8	3	13	-	-	8.8	-	-	16.9	-	-	0.15	0.31	2020	[284]

ITO/HATCN/TAPC/mCP/Ir-1:mCPPO1/TmPyPB/LiF/Al	mCPPO1	Ir-1	8	5.44	2.8	20.6	-	13.6	39.3	-	18.4	47.6	-	31.7	0.187	0.385	2020	[285]
ITO/HAT-CN/TAPC/TCTA/MADN:DSA-ph/TPBi/LiF/Al	MADN	DSA-ph	3	-	-	11.6	-	8.4	13	-	10.3	20.7	-	15.2	0.14	0.28	2020	[286]
-	SPA-2,7-F$(POPh_2)_2$	FIrpic	10	5.8	3.1	18	-	-	38.4	-	-	39	-	-	0.15	0.37	2020	[287]
ITO/PEDOT:PSS/TAPC/mCP/mCP:phosphor/TSPO1/TPBi/LiF/Al	mCP	-	-	-	-	4.4	4.4	1.3	-	4.3	0.8	-	-	-	0.2	0.32	2020	[288]
-	MADN	DSA-ph	-	5.4	2.7	6.1	-	5.5	4.7	-	1.4	4.2	-	3.2	0.15	0.06	2020	[289]

BPBPA:HATCN/BPBPA/mCBP/mCBP:host:CN-IM/DBFTrz/ZADN/LiF/Al	mCP:3CNCzBN	CNIm	15	-	-	25.6	-	23.6	48.2	-	22.8	52.1	-	48.1	0.16	0.32	2020	[290]
ITO/MoO3/CzSi:MoO3/CzSi/BCPO:Ce-1/TSPO1/TPBi/LiF/Al	BCPO	Ce-1	10	6.2	3.1	14	-	-	-	-	-	10.3	-	-	0.146	0.078	2020	[291]
ITO/HAT-CN/TAPC/TCTA/Host:FIrpic/TmPyPB/Liq/Al	BPCN-2Cz	FIrpic	5	5.8	3.1	-	24	21.5	-	46.8	34.5	-	50	44.8	0.15	0.35	2020	[292]
ITO/MoO3/CzSi:MoO3/CzSi/TSPO1:CzSi:Ce-1/TSPO1/Bphen/LiF/Al	TSPO1:CzSi	Ce-1	0.18:0.72:0.1	6.2	3.1	12.4	-	-	-	-	-	8.7	-	-	0.146	0.078	2020	[291]
ITO/MoO_3/TAPC/mCP:Ce-2/TmPyPB/LiF/Al	mCP	Ce-1	10	6.2	3.1	20.8	-	-	30.8	-	-	45.6	-	-	0.17	0.33	2020	[293]

ITO/ReO3:mCBP/mCBP/DPEPO:Cz6/3TPYMB/Liq/Al	DPEPO	Cz6	15	5.42	2.47	16.3	-	11.7	17.6	-	7.9	29.2	-	20.8	0.16	0.26	2020	[294]

Table S4. A comprehensive list of wet-processed blue phosphorescence OLED devices, including the publication year, device composition, key dopant, doping concentration, employed host, HOMO, LUMO, EQE, PE, CE, CIE (X), CIE (Y), and reference. The years span from 2001 to 2020

**Device Structure**	**Host**	**Emitter**				**EQE (%)**			**PE (lm/W)**			**CE (cd/A)**			**CIE**		**Year**	**Ref.**
		**Name**	**Conc. (wt%)**	**HOMO**	**LUMO**	**Max**	**100 (cdm^{-2})**	**1000 (cdm^{-2})**	**Max**	**100 (cdm^{-2})**	**1000 (cdm^{-2})**	**Max**	**100 (cdm^{-2})**	**1000 (cdm^{-2})**	**(X)**	**(Y)**		
ITO/PEDOT/PVK/PSiFC6C6+Ir(PPF)2(PZ)/Ba/Al	PSiFC6C6	Ir(PPF)2(PZ)(4%)	4	-	-	5.9	-	-	-	-	-	9.5	-	-	0.19	0.39	2006	[295]
-	-	fac-Ir(dpbic)3		-	-	-	-	-	-	-	-	-	-	-	0.16	0.05	2006	[296]

ITO/αNPD/TCTA/CzSi/1:UGH2/TAZ/LiF/Al	UGH2	Ir(dfb-pz)2(fptz)	10	5.89	2.12	2.3	-	-	2.9	-	-	1.5	-	-	0.19	0.21	2008	[297]
ITO/PEDOT:PSS/CBP:FIrpic/TPBi/LiF/Al	CBP	FIrpic		5.8	3.1	-	-	-	28.4	25.9	-	-	25.9	-	0.18	0.34	2008	[298]
ITO/NPB/TAPC/p-type host/mixed host:FIr6/SiTAZ/LiF/Al	Opt-MCBP	Fir6	10	6.1	3.1	19	-	-	23.5	19.3	11.1	31.5	30.6	25.9	0.15	0.23	2011	[299]
ITO/PEDOT:PSS/EML/vacuum-deposited TPyQB/Al	PVK+OXD-7	Firpic	-	5.8	3.1	16	-	-	10.9	-	-	30.5	-	-	0.14	0.28	2011	[300]
ITO/PEDOT:PSS/p -TAZSiTPA:FIr6/Tm3PyPB/TPBI/LiF/Al	p -TAZSiTPA	FIr6	10	6.1	3.1	6.3	-	8.8	6.2	-	-	12.5	-	11.4	0.16	0.27	2011	[301]
ITO/PEDOT:PSS/EML/CsF/Al	[PVK (35%) + SimCP2 (35%), OXD-7	FIrpic	5	5.8	3.1	6.2	-	-	15.8	-	-	11	-	-	0.14	0.32	2012	[302]

ITO/PEDOT:PSS/polymeric EML/TPBi/Bphen/LiF/Al	mCP	FIrpic	7	5.8	3.1	6.2	-	-	8.3	-	-	-	-	-	0.174	0.389	2012	[303]
ITO/PEDOT:PSS/hosts:FIrpic/Tm3PyPB/TPBI/LiF/Al	mOXDDSiPA	FIrpic	15	5.8	3.1	10.7	-	-	10.2	-	-	23.4	23.3	17.7	0.15	0.35	2012	[304]
ITO/PEDOT:PSS/hosts:FIrpic/Tm3PyPB/TPBI/LiF/Al	mOXDDSiCz	FIrpic	15	5.8	3.1	4.5	-	-	4.3	-	-	9.7	9.1	7.5	0.15	0.35	2012	
ITO/EIL/SPPO13:TCTA:Firpic/TCTA/MoO3/Al	SPPO13:TCTA	FIrpic	-	5.8	3.1	15.2	-	13.7	20.5	-	13.4	34.3	-	30.8	0.16	0.3	2012	
ITO/ PEDOT:PSS/Host:FIrpic/Cs2CO3/Al	BDPPC	FIrpic	10	5.8	3.1	3.6	-	-	5.2	-	-	7.1	-	-	0.15	0.32	2012	[305]
f ITO/PEDOT:PSS/Hosts:OXD-7:FIrpic/Cs2CO3/Al	Cz-mCP	FIrpic	10	5.8	3.1	11.5	-	-	-	-	-	22.7	-	-	0.15	0.33	2012	[306]
ITO/PEDOT:PSS/PVK/Host:FCNIrpic/TSPO1/LiF/Al	CMPC	FCNIrpic	15	5.72	2.98	12.1	-	-	-	-	-	-	-	-	0.14	0.19	2012	[307]

ITO/PEDOT:PSS/Host:FCNIrpic/TPBi/Cs2CO3/Al	TDPA	FCNIrpic	10	5.72	2.98	5.7	-	-	-	-	-	9.7	-	-	0.18	0.24	2012	[308]
ITO/PEDOT:PSS/PVK/CPBDC:FCNIrpic/TSPO1/LiF/Al	CPBDC	FCNIrpic	10	5.72	2.98	13.8	-	-	-	-	-	-	-	-	0.14	0.22	2012	[309]
ITO/PEDOT:PSS/TCTA:OXD-7:FIrpic/TmPyPB/LiF/Al	TCTA:OXD-7	FIrpic	15	5.8	3.1	-	-	-	16.4	16	12.9	23.5	23.2	22.6	0.16	0.3	2012	[310]
ITO/PEDOT:PSS/DMBC:26DCzPPy:FIrpic/TmPyPB/LiF/Al	DMBC:26DCzPPy	FIrpic	10	5.8	3.1	-	-	12.5	10.5	10	9.5	20.7	-	20	0.17	0.28	2012	[311]
ITO/PEDOT:PSS/hosts:FIrpic/Tm3PyPB/TPBI/LiF/Al	DBISiPA	FIrpic	15	5.8	3.1	-	-	-	4.7	-	-	16.2	-	-	0.16	0.32	2013	[312]
ITO/PEDOT:PSS/Host: FIrpic/TmPyPB/LiF/Al	36FCzG2	Firpic	20	5.8	3.1	9.3	-	-	8.1	-	-	18.5	-	-	0.15	0.31	2013	[313]
ITO/PEDOT:PSS/Host-Ir/TmPyPB/LiF/Al	PVK:OXD-7:UGH3	(dfpmpyCF3)Ir(pic)	12	(dfpmpyCF3)Ir(pic)	3.1	7.53	-	-	5.88	-	-	16.86	-	-	0.17	0.4	2013	[314]

ITO/PEDOT:PSS 1.1/PVK:Ir complex/TPBi/LiF/Al	PVK			-	-	10.4	-	-	12.6	-	-	23.7	-	-	0.18	0.4	2013	[315]
ITO/PEDOT:PSS/first EML/second EML/Au+ITO/LiF/first EML/second EML/LiF/Al	-	SPPO13:FIrpic		-	-	-	-	-	-	-	11	-	-	25	0.16	0.38	2013	[316]
ITO/PEDOT:PSS/NPB/mCP:FIrpic/BCP/LiF/Al	mCP	FIrpic	10	5.8	3.1	-	-	-	10.18	-	-	2.95	-	-	0.167	0.325	2014	[317]
ITO/PEDOT:PSS/PVK/TPCz:FIrpic/SPPO13/LiF/Al	TPCz	FIrpic	10	5.8	3.1	-	-	-	22.3	-	-	15.6	-	-	0.15	0.32	2014	[318]
ITO/PEDOT:PSS/host:co-host:FIrpic/CsF/Al	CzPPO:OXD-7	FIrpic	-	5.8	3.1	-	-	-	5.4	4.9	4.3	12.2	9.5	11.5	0.16	0.39	2014	[319]
ITO/Pedot:PSS/DY/H2:FIrpic/TPBI/LiF/Al	8,8´-spirobi[indolo[3,2,1-de]acridine]	FIrpic	8	5.8	3.1	-	-	-	8.8	-	-	12.2	-	-	0.17	0.27	2015	[320]

ITO/PEDOT:PSS/PVKDPF:OXD-7:FIrpic/TPBi/Ca:Ag	PVKDPF:OXD-7	FIrpic	1	5.8	3.1	-	-	-	17.3	-	17.2	9	-	8.2	0.184	0.381	2015	[321]
ITO/PEDOT:PSS/host:FIrpic/TPBI/LiF/Al	PEPEK	FIrpic	12	5.8	3.1	2.03	-	-	4.14	-	-	11.3	-	-	0.197	0.358	2015	[322]
ITO/PEDOT:PSS/host:FIrpic/TPBI/Cs2CO3/Al	CP-QPBI	FIrpic	12	5.8	3.1	-	-	-	8.2	7	-	17.8	15.5	-	0.16	0.33	2015	[323]
ITO/PEDOT:PSS/exciplexes:FIrpic/TPBI/Ca/Ag	TCTA:POPH	FIrpic	10	5.8	3.1	-	-	-	22.5	-	-	25.8	-	24.5	0.15	0.32	2015	[324]
ITO/PEDOT:PSS/host:FIrpic/Tm3PyPB/TPBi/LiF or CsF/Al	PCzSiPh	FIrpic	10	5.8	3.1	14.3	-	-	10.4	-	-	29.3	-	-	0.15	0.33	2015	[325]
ITO/PEDOT:PSS/TCTA/Hosts: FIrpic/TmPyPB/Cs2CO3/Al	9PhCz-BPO	FIrpic	10	5.8	3.1	12.7	-	-	10.6	-	-	27.8	-	-	0.16	0.34	2015	[326]
ITO/PEDOT:PSS/PVK: OXD-7:emitter/TPBI/Liq/Al	PVK: OXD-7	Ir(dfpmpy)2(pzpy)](PF6)	10	6.01	2.69	0.77	-	-	0.33	-	-	1.56	-	-	0.19	0.33	2016	[327]

ITO/PEDOT:PSS/TAPC/mCP/CzDPEPI:Ir(dbi)3/TSPO1/TPBi/LiF/Al	CzDPEPI	Ir(dbi)3	10	6.13	2.88	24.46	-	12.58	39.47	-	12.71	-	-	-	0.19	0.37	2016	[328]
ITO/PEDOT:PSS/Exciplex:FIrpic/TmPyPB/Cs2CO3/Al	TCTA:TPOB	FIrpic	10		3.1	13.8	-	13.7	22	-	-	28.2	-	28.1	0.15	0.33	2016	[329]
ITO/PEDOT:PSS/TAPC/mCBP-CN:Ir(dbi)3/TSPO1/TPBI/LiF/Al	mCBP-CN	Ir(dbi)3	10	6.13	2.88	24.2	-	23.5	57.1	-	37.4	-	-	-	0.19	0.41	2016	[330]
ITO/PEDOT:PSS/EML-2/CsF/Al	-	Ir-3b	-		-	8.55	-	-	6.15	-	-	17.6	-	-	0.17	0.33	2016	[331]
ITO/PEDOT:PSS/PVK:OXD-7/Ir complex/TPBi/LiF/Al	PVK	FIrpic	15	5.49	1.87	5.4	-	-	5.1	-	-	12.2	-	-	0.19	0.38	2016	[332]
ITO/PEDOT:PSS/Exciplex:FIrpic/TmPyPB/Cs2CO3/Al	TCTA:TPOB	FIrpic	10	5.8	3.1	13.8	-	13.7	13.8	-	-	28.2	-	28.1	0.15	0.33	2016	[329]
ITO/PEDOT:PSS/TAPC/TCTA/host:FIrpic/TmPyPB/LiF/Al	m-CzPyPz	FIrpic	5	5.8	3.1	24.5	-	20.5	30.9	-	-	49.1	-	41.2	0.15	0.35	2016	[333]

ITO/TAPC/mCP/EML/TPBi/LiF/Al	t-BuCPO	Ir2	10	6.19	1.99	13	12.6	10.1	11.2	8.8	5	-	-	-	0.14	0.11	2016	[334]
ITO/NPB/mCP/host:dopant/TmPyPB/LiF/Al	BP2	FIrpic	10	5.8	3.1	5.3	4.9	-	-	-	-	-	-	-	0.17	0.32	2016	[335]
ITO/PEDOT:PSS/TAPC/mCP/DCPPy:FIrpic/TmPyPB/LiF/Al	CPDPy	FIrpic	10	5.8	3.1	21.6	-	21.6	24.4	-	19.4	43.3	-	43.2	0.16	0.31	2017	[336]
ITO/PEDOT/TAPC/mCP/mCP:1/TSPO1/TPBi/LiF/Al	mCP	fac-tris(2,3-dimethylimidazo[1,2-f]phenanthridinato-k2 C, N)iridium(III)	3		-	20.41	18.35	13.11	32.23	-	-	-	-	-	0.18	0.34	2018	[337]
ITO/PEDOT:PSS/iridium (III) complex:mCP/TmPyPB/Liq/Al	mCP	(DNaTPA)2DBF(FIrpic)2	2	5.58	2.4	6.22	2.24	-	3.13	0.81	-	12.96	0.81	-	0.19	0.34	2018	[338]

ITO/PEDOT:PSS/TAPC/mCP/mCP:CNIr/TSPO1/TPBi/LiF/Al	mCP	CNIr	5	5.77	2.54	22.5	-	17.6	33	-	16.5	40.9	-	32.3	0.15	0.28	2018	[339]
ITO/PEDOT:PSS/TAPC/PCZAC/mCP/ mCPPO1:dopant 10/TSPO1/TPBi/LiF/Al	mCPPO1	1,1-Bis(2´,6´-difluoro-2,3´-bipyridyl-N,C4’)-1-methoxyethane]platinum(II)	5		-	17.6	17	13.6	- -	24.9	16	-	-	-	0.15	0.29	2018	[340]
-	SimCP2	FIrpic	-	5.8	3.1	-	-	-	9.9	-	-	15.76	-	-	0.14	0.28	2018	[341]
ITO/PEDOT:PSS/PVK/ Host:OXD-7:FIrpic/Ba/Al	134OXD	FIrpic	15	5.8	3.1	-	-	-	-	-	-	8.75	6.26	-	0.17	0.37	2018	[342]
ITO /PEDOT:PSS/poly-TPD/t-Ph-mCP:FIrpic/TSPO1/TPBi/LiF/Al	t-Ph-mCP	Firpic	10	5.8	3.1	5.2	2.5	-	-	-	-	9.6	4.8	-	0.16	0.34	2019	[343]

ITO/PEDOT:PSS/TCTA:Ir complexes/TmPyPB/Liq/Al	TCTA	Ir(ppySCF3)2 (bor)	10	6.28	2.26	1.14	-	-	1.21	-	-	2.31	-	-	0.16	0.32	2019	[344]
ITO/ PEDOT: PSS:GNPs/CBP:FIrpic/TPBi/LiF/Al	CBP	FIrpic	15	5.8	3.1	-	8	5.9	-	19.3	14.5	-	12.4	8.1	0.17	0.42	2020	[345]
ITO/PEDOT:PSS/host:Firpic/DPEPO/TmPyPB/Liq/Al	BPhCz-ThX	FIrpic	15	5.8	3.1	9.6	-	-	8.5	-	-	21	-	-	0.19	0.36	2020	[346]

Table S5. A comprehensive list of dry-processed blue TADF OLED devices, including the publication year, device structure, key dopant, doping concentration, employed host, HOMO, LUMO, EQE, PE, CE, CIE (X), CIE (Y), and reference. The years span from 2015 to 2020.

**Device Structure**	**Host**	**Emitter**				**EQE (%)**			**PE (lm/W)**			**CE (cd/A)**			**CIE**		**Year**	**Ref.**
		**Name**	**Conc.**	**HOMO**	**LUMO**	**Max**	**100**	**1000**	**Max**	**100**	**1000**	**Max**	**100**	**1000**	**(X)**	**(Y)**		

			(wt %)				(cdm^{-2})			(cdm^{-2})			(cdm^{-2})					
ITO/ReO3:mCP/mCP/ mCP:TSPO1:DPTDDA/TSPO1/ Rb2CO3:TSPO1/Al	mCP	DTPDDA	16 %	5.57	2.8	22.3	-	10.6	30.4		9.8	35.6		17.9	0.149	0.197	2015	[347]
ITO/α-NPD/mCP/mCP:2CzPN/mCP/PPT/LiF/Al	mCP	2CzPN	10 %	5.8	3	24	12.6	-	30.9	12.6	-	47.2	26.1	-	0.17	0.33	2015	[348]
ITO/F4-TCNQ/α-NPD/EML/BCP/Alq3/LiF/Al	-	-	-	-	-	4	-	-	7.65	-	-	14.6	-	-	0.168	0.227	2015	[349]
ITO/NPB/TAPC/mCBP: Dopant (A-D or D1 or D2/PPT/TmPyPb/LiF/Al	mCBP	BPy-pC	-	5.68	2.54	4.2	-	-	3.9	-	-	5	-	-	0.16	0.13	2016	[350]
ITO/NPB/TAPC/mCBP: Dopant (A-D or D1 or D2/PPT/TmPyPb/LiF/Al	mCBP	BPy-pTC	-	5.6	2.63	9.4	-	-	14.6	-	-	16.3	-	-	0.17	0.27	2016	[350]
ITO/NPB/TAPC/mCBP: Dopant (A-D or D1 or D2/PPT/TmPyPb/LiF/Al	mCBP	BPy-p2C	-	5.58	2.63	11	-	-	16.2	-	-	20.8	-	-	0.18	0.28	2016	[350]
ITO/NPB/TAPC/mCBP: Dopant (A-D or D1 or D2/PPT/TmPyPb/LiF/Al	mCBP	BPy-p3C	-	5.53	2.58	23.9	-	-	50.6	-	-	56.5	-	-	0.19	0.32	2016	
ITO/NPB/TAPC/mCBP: Dopant (A-D or D1 or D2/PPT/TmPyPb/LiF/Al	mCBP	BPy-p3C	-	5.53	2.58	14.5	-	-	31.5	-	-	35.2	-	-	0.2	0.36	2016	

ITO/NPB/TAPC/mCBP: Dopant (A-D or D1 or D2/PPT/TmPyPb/LiF/Al	mCBP	BPy-p3C	-	5.53	2.58	9.9	-	-	18.1	-	-	25.3	-	-	0.2	0.38	2016	
ITO/NPB/TCTA/CyFbCz:mCP/TPBi/LiF/Al	mCBP	CyFbCz	8%	5.3	2.4	-	-	-	2.66	-	-	6.64	-	-	0.18	0.13	2016	[351]
ITO/ReO3:mCP/mCP/mCP:2CzPN/ReO3:PO-15/Al		-	-	-	-	21.8	-	2.8	47.4	-	3	45.3	-	5.5	0.17	0.27	2016	[352]
ITO/α-NPD/mCP/EML/PPF/TPBi/LiF/Al	PPF	Cz-VPN	-	6.1	2.9	8.7	-	-	12.3	-	-	18	-	-	0.15	0.18	2016	[353]
ITO/HAT-CN/a-NPD/CCP/emitter:PPF/PPF/TPBi/Liq/Al	PPF	2DPAC-MPM	-	6.04	3.04	19	9.4	-	27.9	11.1	-	32	16.5	-	0.16	0.21	2016	[354]
ITO/HAT-CN/a-NPD/CCP/emitter:PPF/PPF/TPBi/Liq/Al	PPF	2DPAC-PPM	-	6.04	3.07	20.8	12.4	-	31.5	14.6	-	38.1	23.5	-	0.16	0.24	2016	
ITO/CuI/TPD/host:*p*-TPB-2Cz/PBD/Ca/Al	mCP	m-TPB-2Cz	-	5.36	2.56	3.5	-	-	0.85	-	-	3.7	-	-	0.25	0.23	2017	[355]
ITO/CuI/TPD/host:*p*-TPB-2Cz/PBD/Ca/Al	mCP	m-TPB-2Cz	-	5.36	2.56	14.1	-	-	4.2	-	-	11.1	-	-	0.22	0.19	2017	
ITO/MoO3/TAPC/Hosts/TAPC/MoO3/Al	DPEPO	CzDPPy	-	5.52	1.97	16	-	-	33.1	-	-	34.8	-	-	0.18	0.34	2017	[356]
ITO/MoO3/TAPC/Hosts/TAPC/MoO3/Al	DPEPO	tCzPN	-	5.4	1.98	11.7	-	-	23.3	-	-	28.9	-	-	0.22	0.38	2017	

ITO/HATCN/α-NPD/CCP/EML/PPF/TPBi/Liq/Al	PPF	MXAc-BF	-	5.44	2.48	16	15.7	12	31.5	-	-	-	-	-	0.17	0.29	2017	[357]
ITO/HATCN/α-NPD/CCP/EML/PPF/TPBi/Liq/Al	PPF	MXAc-CM	-	5.56	2.77	15	14.6	11.1	25.7	-	-	-	-	-	0.16	0.29	2017	
ITO/HATCN/α-NPD/CCP/EML/PPF/TPBi/Liq/Al	PPF	XAc-CM	-	5.73	2.87	12.1	11.6	7.1	14.7	-	-	-	-	-	0.15	0.19	2017	
ITO/TAPC/TCTA/mCP/mCP:B4PyMPM:FIrpic:PO-01/B4PyMPM/Liq/Al	-	-	-	-	-	17.3	17.2	16.5	48	45.1	35.8	41.3	41.1	39.6	0.18	0.39	2017	[358]
ITO/HATCN/NPB/mcp/DPEPO:BdPyInCz/DBFPO/LiQ/Al	-	-	-	-	-	17.1	12.6	-	48	-	-	-	-	-	0.196	0.34	2017	[359]
ITO/HATCN/NPB/mcp/DPEPO:BdPyInCz/DBFPO/LiQ/Al	-	-	-	-	-	13.6	11.2	-	-	-	-	-	-	-	0.173	0.266	2017	
ITO/TAPC/TCTA/DAB-DCNB:mCP/TmPyPB/LiF/Al	mCP	DAB-DCNB	-	5.07	2.21	16.5	-	-	45.8	-	-	40.8	-	-	0.22	0.38	2017	[360]
ITO/PPBI/TAPC/emitter-host/B3PyPB/LiF/Al	mCP	Ac-46DPPM	-	5.14	2.18	11.8	4.48	1.73	19.7	5.45	1.29	18.3	6.95	2.46	0.16	0.21	2017	[361]
ITO/PPBI/TAPC/emitter-host/B3PyPB/LiF/Al	mCP	Ac-26DPPM	-	5.26	2.21	18.6	11.9	6.34	43.5	21.2	8.61	39.6	25.3	13.5	0.18	0.33	2017	
ITO/PPBI/TAPC/emitter-host/B3PyPB/LiF/Al	mCP	CzAc-26DPPM	-	5.34	2.36	22.8	14.9	9.28	59.2	29.3	14.1	53.9	35.3	21.9	0.21	0.38	2017	

ITO/TAPC/CCX-I:DPEPO/BmPyPhB/Liq/Al	PPF	CCX-I-6B	-	5.49	1.93	17.6	13.7	9	24.7	-	-	28.3	-	-	0.16	0.21	2017	[362]
ITO/TAPC/CCX-I:DPEPO/BmPyPhB/Liq/Al	PPF	CCX-II-6B	-	5.5	2.03	25.9	20.2	14.4	35.9	-	-	41.1	-	-	0.15	0.22	2017	
ITO/TAPC/CCX-I:DPEPO/BmPyPhB/Liq/Al	PPF	CCX-II-10B	-	5.5	2.03	24.5	21.8	17	44.9	-	-	44.8	-	-	0.16	0.26	2017	
ITO/TAPC/CCX-I:DPEPO/BmPyPhB/Liq/Al	PPF	CCX-II-15B	-	5.5	2.03	24	22.1	18.4	52.5	-	-	47.5	-	-	0.16	0.29	2017	
ITO/TAPC/CCX-I:DPEPO/BmPyPhB/Liq/Al	PPF	CCX-I-6B-OC	-	5.49	1.93	33.3	28.9	21.9	54.4	-	-	55.4	-	-	0.16	0.22	2017	
ITO/ MoO3/NPB/PFBP-1b:mCP/TPBi/Ca:Al	mCP	PFPB-1b	-	6.19	3.06	3.3	-	-	12.6	-	-	6.5	-	-	0.177	0.266	2018	[363]
ITO/ MoO3/NPB/PFBP-1b:mCP/TPBi/Ca:Al	TCz1	PFPB-2a	-	5.92	2.88	3.9	-	-	4.4	-	-	3.7	-	-	0.199	0.289	2018	
ITO/PEDOT:PSS/TAPC/mCP/DPEPO:dopant/TSPO1/TPBi/LiF/Al	DPEPO	23CT	30%	-	-	21.8	21.8	20.8	45.8	45.8	43.8	35.5	35.5	27.5	0.17	0.33	2018	[364]
ITO/PEDOT:PSS/TAPC/mCP/DPEPO:dopant/TSPO1/TPBi/LiF/Al	DPEPO	24CT	30%	-	-	22.4	20.4	14.5	36	36	25	28	28	15	0.15	0.26	2018	
ITO/PEDOT:PSS/TAPC/mCP/DPEPO:dopant/TSPO1/TPBi/LiF/Al	DPEPO	34CT	30%	-	-	13.3	9.4	5.6	13.6	13.6	7.3	9.5	9.5	3.5	0.15	0.17	2018	

ITO/TAPC:MoO3/TAPC/TCTA/CzSi/DPEPO:PhPMAF or 2NPMAF/DPEPO/TmPyPB/LiF/Al	DPEPO	PhPMAF	22%	-	-	19.7	14.4	-	40.7	21.4	-	36.3	26.5	-	0.168	0.25	2018	[365]
ITO/TAPC:MoO3/TAPC/TCTA/CzSi/DPEPO:PhPMAF or 2NPMAF/DPEPO/TmPyPB/LiF/Al	DPEPO	2NPMAF	22%	-	-	23.6	18.4	-	56.7	33.9	-	50.6	39.4	-	0.172	0.231	2018	
ITO/TAPC:MoO3/TAPC/TCTA/CzSi/DPEPO:PhPMAF or 2NPMAF/DPEPO/TmPyPB/LiF/Al	DPEPO	2NPMAF	22%	-	-	24.9	21.2	-	68.2	46	-	60.7	51.6	-	0.187	0.343	2018	
ITO/NPB/mCP/EML/PPT/TmPyPB/LiF/Al	mCP	2CzdOXMe	6%	5.83	2.7	4.7	2.6	1.8	3.7	-	-	4.2	-	-	0.156	0.118	2018	[366]
ITO/NPB/mCP/EML/PPT/TmPyPB/LiF/Al	PPT	2CzdOXPh	6%	5.84	2.8	6.8	2	1.4	5.9	-	-	7.5	-	-	0.161	0.15	2018	
ITO/NPB/mCP/EML/PPT/TmPyPB/LiF/Al	PPT	2CzdOX4CF3Ph	6%	5.84	2.86	11.2	2.5	-	14.5	-	-	18.9	-	-	0.165	0.254	2018	
ITO/NPB/mCP/EML/PPT/TmPyPB/LiF/Al	mCP	2CzdOXD4MeOph	6%	5.88	2.74	6.6	2	0.8	5	-	-	5.7	-	-	0.147	0.108	2018	
ITO/NPB/mCP/EML/PPT/TmPyPB/LiF/Al	PPT	2CzdOXD4meOpPh	6%	5.88	2.74	4.2	2.4	1.5	3.2	-	-	3.8	-	-	0.153	0.118	2018	

ITO/NPB/mCP/EML/PPT/TmPyPB/LiF/Al	mCP	2CzPN	6%	5.88	2.97	14.2	10.3	4	18.4	-	-	25.9	-	-	0.16	0.3	2018	
ITO/ MoO3/TAPC/ 2,6-DCzppy:TBN-TPA/TmPyPB/LiF/Al	2,6-Dczppy	TBN-TPA	4%	5.19	2.56	32.1	27.4	13.9	30	23.5	8.8	40.2	34.4	17.4	0.12	0.19	2018	[367]
ITO/MoO3/TAPC/mCP/DPEPO:emitters/DPEPO/TmPyPB/LiF/Al	DPEPO	*p*BFCz-26DPPM	10 %	5.26	1.82	6.2	-	-	2.5	-	-	3.57	-	-	0.154	0.054	2018	[368]
ITO/MoO3/TAPC/mCP/DPEPO:emitters/DPEPO/TmPyPB/LiF/Al	DPEPO	*p*BTCz-26DPPM	20 %	5.3	1.84	5.67	-	-	3.27	-	-	3.55			0.152	0.06	2018	
ITO/HATCN/TAPC/DCDPA/host:DMAC-DPS/TSPO1/TPBi/LiF/Al	DPEPO	DMAC-DPS	-	-	-	23.9	-	16.6	-	-	-	30.7		21.3	0.15	0.2	2018	[369]
ITO/HATCN/TAPC/DCDPA/host:DMAC-DPS/TSPO1/TPBi/LiF/Al	PPO2	DMAC-DPS	-	-	-	27.8	-	21.8	-	-	-	36.1		28.4	0.15	0.22	2018	
ITO/HATCN/TAPC/DCDPA/host:DMAC-DPS/TSPO1/TPBi/LiF/Al	3DCPO	DMAC-DPS	-	-	-	23.1	-	17	-	-	-	32.5		23.9	0.15	0.22	2018	
ITO/HAT-CN/NPB/mCBP/DPEPO:dopant/DPEPO/TPBi/Liq/Al	-	Cy-2Cz	-	5	1.48	11.9	10.9	-	8.5	6.2	-	11.8	10.8	-	0.16	0.1	2018	[370]
ITO/HAT-CN/NPB/mCBP/DPEPO:dopant/DPEPO/TPBi/Liq/Al	-	Cy-3Cz	-	4.91	1.46	5.4	3.7	-	5.1	2.3	-	6.6	4.6	-	0.17	0.14	2018	

ITO/HAT-CN/NPB/mCBP/DPEPO:dopant/DPEPO/TPBi/Liq/Al	-	sf-3Cz	-	5.02	1.51	15.8	6.1	-	13.7	3.2	-	19.2	7.3	-	0.16	0.14	2018	
ITO/MoO3/TAPC/mCP/EML/DPEPO (5 nm) /TmPyPB/LiF/Al	mCBP	IndCzpTr-1	20%	4.92	1.91	14.5	11	-	-	11.5	-	28.1	20.5	-	0.17	0.27	2018	[371]
ITO/MoO3/TAPC/mCP/EML/DPEPO/TmPyPB/LiF/Al	DPEPO	TCTZ	6%	5.68	2.27	17.1	-	8.6	19.73	-	-	28	-	-	0.16	0.24	2018	[372]
ITO/HAT-CN/TAPC/emitter:DPEPO/TPBi/LiF/Al	DPEPO	BP-Ph-DPAC	16%	5.27	2.25	4.25	-	-	6.85	-	-	8.08	-	-	0.16	0.29	2018	[373]
ITO/HAT-CN/TAPC/emitter:DPEPO/TPBi/LiF/Al	DPEPO	BP-DPAC	25%	5.31	2.61	6.82	-	-	13.52	-	-	15.08	-	-	0.18	0.36	2018	
ITO/HAT-CN/TAPC/mCP/DPEPO:TRTZ or SFCC/3TPYMB/Liq/Al	DPEPO	TRTZ	30%	5.42	1.95	4.46	-	-	2.5	-	-	4	-	-	0.16	0.11	2018	[374]
ITO/HAT-CN/TAPC/mCP/DPEPO:TRTZ or SFCC/3TPYMB/Liq/Al	DPEPO	SFCC	30%	5.27	1.97	10.59	-	-	11.04	-	-	15	-	-	0.17	0.21	2018	
ITO/TPBi/PHCz2BP/TPBi/LiF/Al	DPEPO	PHCz2BP	-	5.7	2.2	4	4	3.6	11.04	-	-	-	-	-	0.15	0.26	2018	[375]
ITO/HATCN/TAPC/TCTA/mCP/DBFPO:emitters/TSPO1/TPBi/LiF/Al	-	TCzTrZ	-	5.36	2.54	22.1	-	10.1	6.9	-	-	33.5	-	15.3	0.16	0.23	2018	[376]

ITO/HATCN/TAPC/TCTA/mCP/DBFPO:emitters/TSPO1/TPBi/LiF/Al	-	DCzCbTrZ	-	5.39	2.51	7.1	-	-	-	-	-	5.9	-	-	0.16	0.11	2018	
ITO/HATCN/TAPC/TCTA/mCP/DBFPO:emitters/TSPO1/TPBi/LiF/Al	-	TCzCbTrZ	-	5.54	2.55	12.8	-	3.2	-	-	-	13.2	-	3.3	0.15	0.12	2018	
ITO/MoO3/TAPC/2,6- DCzppy:guest /TmPyPB/LiF/Al	-	TN4T-PCZ	-	5.76	2.56	3.8	-	-	-	-	-	1.38	0.87	1.3	0.17	0.06	2018	[377]
ITO/MoO3/TAPC/2,6- DCzppy:guest /TmPyPB/LiF/Al	-	NTN-PCZ	-	5.73	2.36	20.4	-	-	-	-	-	5.01	2.69	4.74	0.16	0.03	2018	
ITO/TAPC/mCP/DCzBN1-4:DPEPO/DPEPO/TPBi/LiF/Al	mcp	DCzBN1	-	5.82	2.52	2.5	-	-	0.6	-	-	0.8	-	-	0.15	0.05	2018	[378]
ITO/TAPC/mCP/DCzBN1-4:DPEPO/DPEPO/TPBi/LiF/Al	mcp	DCzBN2	-	5.72	2.54	7.7	-	-	2.7	-	-	3.9	-	-	0.15	0.07	2018	
ITO/TAPC/mCP/DCzBN1-4:DPEPO/DPEPO/TPBi/LiF/Al	mcp	DCzBN3	-	5.72	2.49	10.3	-	-	3.5	-	-	5.1	-	-	0.16	0.06	2018	
ITO/TAPC/mCP/DCzBN1-4:DPEPO/DPEPO/TPBi/LiF/Al	mcp	DCzBN4	-	5.72	2.54	18	10.4	4.1	19	-	-	24.2	-	-	0.16	0.23	2018	
ITO/HATCN/TAPC/mCP/emitter:DPEPO/DPEPO/TPBi/LiF/Al	DPEPO	2SPAc-HPM	-	5.46	2.6	25.56	18.86	11.94	51.6	26.65	12.98	57.5	42.42	26.85	0.18	0.34	2018	[379]
ITO/HATCN/TAPC/mCP/emitter:DPEPO/DPEPO/TPBi/LiF/Al	DPEPO	2SPAc-MPM	-	5.45	2.54	24.34	16.14	9.16	42.45	20.03	7.68	50	33.15	18.82	0.17	0.29	2018	

ITO/HATCN/TAPC/mCP/emitter:DPEPO/DPEPO/TPBi/LiF/Al	DPEPO	2SPAcPPM	-	5.45	2.57	31.45	18.34	10.84	56.85	23.78	9.55	68.77	40.11	23.71	0.18	0.32	2018	
ITO/HATCN/TAPC/DCDPA/host:DMAC-DPS/TSPO1/TPBi/LiF/Al	-	-	-	-	-	17.5	-	10.8	-	-	-	24.4	-	15.2	0.16	0.23	2018	[380]
ITO/HATCN/TAPC/DCDPA/host:DMAC-DPS/TSPO1/TPBi/LiF/Al	-		-	-	-	22.9	-	19.8	-	-	-	35	-	29.7	0.15	0.26	2018	
ITO/HATCN/TAPC/DCDPA/host:DMAC-DPS/TSPO1/TPBi/LiF/Al	-		-	-	-	18.8	-	14.5	-	-	-	31.3	-	24.6	0.16	0.28	2018	
ITO/TAPC/mCP/mitter:DPEPO/DPEPO/TmPyPB/LiF/Al	DPEPO	PDTCz-DpmS	-	5.98	1.86	14	7	-	21.8	-	-	-	-	-	0.19	0.26	2019	[381]
ITO/HAT-CN/TAPC/TCTA/emitter:mCP/TmPyPb/LiF/Al	mCP	DiKTa	3.5	-	-	14.7	8.3	3.3	-	-	-	-	-	-	0.14	0.18	2019	[382]
ITO/HAT-CN/TAPC/TCTA/emitter:mCP/TmPyPb/LiF/Al	mCP	Mes3DiKTa	3.5	-	-	21.1	14.5	4.5	-	-	-	-	-	-	0.12	0.32	2019	
ITO/HAT-CN/NPB/mCP/Host:emitter/DBFPO/DBFPO:Liq/Liq/Al	-	CPT2	-	-	-	18.7	-	-	-	-	-	30.1	-	-	0.162	0.206	2019	[383]

ITO/HAT-CN/NPB/mCP/Host:emitter/DBFPO/DBFPO:Liq/Liq/Al	DPEPO	BMK-T138	-	5.16	1.79	19.6	-	-	-	-	-	40.6	-	-	0.155	0.314	2019	
ITO/TAPC/mCP/DPEPO:emitter/DPEPO/TmPyPB/LiF/Al	DPEPO	2CPTCB	-	4.96	1.84	21.1	-	-	-	-	-	-	-	-	0.15	0.19	2019	[384]
ITO/NPB/TSBPA/DPEPO:DtBuAc-DBT/DPEPO/TPBi/LiF/Al	DPEPO	DtBuAc-DBT	-	5.47	0.71	10.5	-	9.8	-	-	-	13.9	-	-	0.133	0.129	2019	[385]
ITO/MoOx/TCTA/NPB/TPBi:PPh3O/PPh3O/BPhen/CsF/Al.	-	PPh3O	-	6	1.5	4	-	-	2.23	-	-	3.6	-	-	0.1525	0.082	2019	[386]
ITO/HATCN/TAPC/DCDPA/DBFPO:dopant/DBFPO/TPBi/LiF/Al	DPEPO	Trz-oIc	20	5.75	2.8	23.2	-	13.3	-	-	-	46.1	-	-	0.19	0.37	2019	[387]
ITO/HATCN/TAPC/DCDPA/DBFPO:dopant/DBFPO/TPBi/LiF/Al	DPEPO	Trz-mIc	20	5.72	2.75	28	-	23.2	-	-	-	56.5	-	-	0.2	0.39	2019	
ITO/Moo3/NPB/mCP/SXSPO:DMAC-DPS/Bphen/LiF/Al	-	DMACDPS	30	5.9	2.4	22.6	21.7	18	39.6	24.8	13.8	37.2	35.5	29.6	0.15	0.2	2019	[388]
ITO/Moo3/NPB/mCP/SXSPO:DMAC-DPS/Bphen/LiF/Al	-	DMACDPS	30	5.9	2.4	2.9	3.5	2.4	3.2	2.4	1.2	5	6.1	4.1	0.17	0.18	2019	
ITO/HATCN/NPB/TCTA/mCBP/mCBP:dopant/PPT/Li2CO3:Bepp2/Li2CO3/Al	mCBP	NOBF2-Cz	10	5.7	2.99	11	-	-	12.5	-	-	12.6	-	-	0.14	0.16	2019	[389]

ITO/HATCN/NPB/TCTA/mCBP/mCBP:dopant/PPT/Li2CO3:Bepp2/Li2CO3/Al	mCBP	NOBF2-DTCz	10	5.59	3	12.7	-	-	44	-	-	40.6	-	-	0.14	0.21	2019	
ITO/HATCN/NPB/TCTA/mCBP/mCBP:dopant/PPT/Li2CO3:Bepp2/Li2CO3/Al	mCBP	NOBF2-DPCz	10	5.6	3	15.8	-	-	22.7	-	-	25.3	-	-	0.14	0.28	2019	
ITO/TPBi/DPAc-DCzBN in DPEPO, DPAc-DCzBN, DPAc-DtCzBN in DPEPO, or DPAc-DtCzB/TPBi/LiF/Al	DPEPO	DPAc-DCzBN	20 %	5.53	2.39	22.8	18	-	-	-	-	40.9	-	-	0.17	0.25	2019	[390]
ITO/TPBi/DPAc-DCzBN in DPEPO, DPAc-DCzBN, DPAc-DtCzBN in DPEPO, or DPAc-DtCzBN/TPBi/LiF/Al	DPEPO	DPAc-DtCzBN	30 %	5.55	2.47	23.1	18.3	-	33.8	-	-	31.3	-	-	0.16	0.15	2019	
ITO/NPB/TSBPA/emitter I–IV:DPEPO/DPEPO/TPBi/LiF/Al	DPEPO	I	35 %	5.7	2.6	21.2	20	19.3	22	-	-	36.8	-	-	0.16	0.25	2019	[391]
ITO/NPB/TSBPA/emitter I–IV:DPEPO/DPEPO/TPBi/LiF/Al	DPEPO	II	35 %	5.6	2.5	15.6	14.3	11.2	27.5	-	-	29.6	-	-	0.17	0.29	2019	
ITO/NPB/TSBPA/emitter I–IV:DPEPO/DPEPO/TPBi/LiF/Al	DPEPO	III	35 %	5.7	2.6	22.6	22.5	19.9	18.6	-	-	31.7	-	-	0.15	0.18	2019	
ITO/TAPC/ mCP/EML/DPEPO/TmPyPb/LiF/Al	DPEPO	TspiroS-TRZ	30.00 %	5.42	4.93	33.3	31.1	23.7	24.3	-	-	71.1	66.9	50.6	0.17	0.33	2019	[392]

ITO/TAPC/ mCP/EML/DPEPO/TmPyPb/LiF/Al	DPEPO	TspiroF-TRZ	30.00%	5.67	5.04	28.1	27.7	23.1	-	-	-	68.4	53.3	56.2	0.19	0.39	2019	
ITO/TAPC/mCP/EML/DPEPO/TmPyPb/LiF/Al	DPEPO	TspiroS-TRZ	100%	-	-	20	16.2	10.6		26.1	14.9	41.1	33.3	21.9	0.16	0.3	2019	
ITO/TAPC/mCP/EML/DPEPO/TmPyPb/LiF/Al	-	-	-	-	-	16.5	15.4	9.7	35.8	29.1	15.8	39	36.4	22.8	0.18	0.34	2019	
ITO/HATCN/NPB/X/TPBi/LiF/Al	-	DPECTT	-	-	-	-	-	-	33.5	-	-	3.96	-	-	0.19	0.38	2019	[393]
ITO/Moo3/mCP/mCP:TCzPzCp/PPT/LiQ/Al	-	TCzPzCN	10%	5.57	2.92	7.6	-	-	-	-	-	-	-	-	0.15	0.29	2019	[394]
ITO/Moo3/mCP/mCP:TCzPzCp/ PT/LiQ/Al	-	2TCzPzCN	10%	5.53	2.95	12.2	-	-	-	-	-	-	-	-	0.15	0.26	2019	
ITO/HATCN/TAPC/DCDPA/host:dopant/DBFPO/TPBi/LiF/Al	DBFPO	PXB-DI	20%	5.54	2.62	37.4	-	31.7	-	-	-	66.2	-	56.1	0.16	0.34	2019	[395]
ITO/HATCN/TAPC/DCDPA/host: dopant/DBFPO/TPBi/LiF/Al	PPBI	PXB-DI	20%	5.54	2.62	28.4	-	21.4	-	-	-	37.8	-	32.5	0.14	0.2	2019	
ITO/HATCN/TAPC/DCDPA/host:dopant/DBFPO/TPBi/LiF/Al	DBFPO	PXB-mIC	20%	5.73	2.63	18.8	-	12.8	-	-	-	22.1	-	15.1	0.14	0.18	2019	

ITO/HATCN/TAPC/DCDPA/host:dopant/DBFPO/TPBi/LiF/Al	PPBI	PXB-mIC	20%	5.73	2.63	12.5	-	5.2	-	-	-	8.6	-	3.6	0.15	0.08	2019	
ITO/NPB/TCTA/mCPPO1/mCPPO1:PO-T2T/PO-T2T/LiF/Al	-	-	-	-	-	6.1	4.7	-	-	-	-	8.9	8	-	0.16	0.21	2019	[396]
ITO/NPB/TCTA/mCPPO1/mCPPO1:PO-T2T/PO-T2T/LiF/Al	-	-	-	-	-	16	14.5	12.9	7	-	-	27	26.7	24.6	0.16	0.28	2019	
ITO/NPB/TCTA/mCPPO1/mCPPO1:PO-T2T/PO-T2T/LiF/Al	-	-	-	-	-	6.5	6.3	4.5	26.4	-	-	9.4	9.5	8.8	0.18	0.29	2019	
ITO/PPBI/TAPC/PXZ-BIP-mCP/PXZ-BIP-DPEPO/B3PyPB/LiF/Al	-	-	-	-	-	2.64	1.2	-	8	0.62	-	2.44	1.05	-	0.16	0.1	2019	[397]
ITO/PPBI/TAPC/PXZ-BIP-mCP/PXZ-BIP-DPEPO/B3PyPB/LiF/Al	-	-	-	-	-	5.93	1.55	-	2.4	2.29	-	12.2	3.18	-	0.19	0.3	2019	
ITO/PEDOT:PSS/mCP:polymers/DPEPO/TmPyPB/Liq/Al	mCP	CP1	5%	5.11	1.31	6.36	2.8	-	6.68	-	-	10.64	-	-	0.18	0.25	2019	[398]
ITO/PEDOT:PSS/mCP:polymers/DPEPO/TmPyPB/Liq/Al	mCP	CP2	5%	4.89	1.03	1.12	0.47	-	0.81	-	-	1.81	-	-	0.2	0.26	2019	
ITO/PEDOT:PSS/mCP:polymers/DPEPO/TmPyPB/Liq/Al	mCP	CP3	5%	5.18	1.19	3.49	3.3	-	2.51	-	-	5.59	-	-	0.16	0.23	2019	
ITO/PEDOT:PSS/mCP:polymers/DPEPO/TmPyPB/Liq/Al	CzAcSF	CP1	5%	5.11	1.31	9.74	8.8	-	10.75	-	-	16.96	-	-	0.17	0.24	2019	

ITO/PEDOT:PSS/mCP:polymers/DPEPO/TmPyPB/Liq/Al	CzAcSF	CP2	5%	4.89	1.03	6.51	5.77	-	7.75	-	-	12.32	-	-	0.19	0.28	2019	
ITO/PEDOT:PSS/mCP:polymers/DPEPO/TmPyPB/Liq/Al	CzAcSF	CP3	5%	5.18	1.19	4.87	4.5	-	5.89	-	-	9.38	-	-	0.19	0.28	2019	
ITO/Moo3/TCTA/mCP/Tczi: (1) and (4)/TSPo1/TPBi/LiQ/Al	TCz1	4	25%	-	-	6.3	4	-	12.6	-	-	-	-	-	0.18	0.32	2019	[399]
ITO/MoO3/TAPC/ TCTA/Emitters/TmPyPB/LiF/Al	Nill	3CP-DPSDMAC	Film (20 nm)	5.31	2.01	9.1	-	2.8	-	-	15.7	17	-	-	0.16	0.28	2020	[400]
ITO/NPB/TSBPA/DMAC-TXO2:DPEPO/DPEPO/TPBi/LiF/Al.	DPEPO	DMAC-TXO2	30%	-	-	20.6	-	-	24.5	-	-	35.1	-	-	0.16	0.24	2020	[401]
ITO/PEDOT:PSS/TAPC/mCP/ mCP:dopant/DPEPO/TmPyPB/LiF/Al	mCP	TrzDMAC-Me	5%	5.24	2.91	19.01	-	-	30.93	-		41.12	-	-	0.18	0.33	2020	[402]
ITO/MoO3/NPB/mCP/tBCzPPOSPO:DAMC-DPS:4CzTPNBu/DBFDPO/LiF/Al,	tBCzPPOSPO (5.45,1.012)	-	-	-	-	21.1	20.8	17.5	63.5	42.3	26.1	60.7	60.2	50.2	0.36	0.44	2020	[403]
ITO/HATCN/NPB/PCZAC/mCP or pCzBzbCz or pCzPybCz:DBA-DI/DDBFT/p-bPPhenB/LiF/Al	-	30 wt% DBA-DI	-	-	-	24	-	-	42.6	-	-	40.7	-	-	0.14	0.3	2020	[404]

ITO/HATCN/NPB/PCZAC/mCP or pCzBzbCz or pCzPybCz:DBA-DI/DDBFT/p-bPPhenB/LiF/Al	-	30 wt% DBA-DI	-	-	-	22.7	-	-	40.7	-	-	42.2	-	-	0.15	0.35	2020	
ITO/HATCN/TAPC/TCTA/DPEPO or DMAC-DPS:TBPe/TmPyPB/LiF/Al	DPEPO (6.1,2.0)	TBPe	1%	5.4	2.7	10	7.4	1.9	12.5	6.6	1	12.7	9.2	2.3	0.14	0.17	2020	[405]
ITO/HATCN/TAPC/TCTA/DPEPO or DMAC-DPS:TBPe/TmPyPB/LiF/Al	DPEPO (6.1,2.0)	TBPe	1%	5.4	2.7	14.1	11.3	7.3	16	11.4	5.3	16.2	14.5	9.4	0.14	0.17	2020	
ITO/HATCN/TAPC/ TCTA/DPEPO or DMAC-DPS:TBPe/TmPyPB/LiF/Al	DPEPO (6.1,2.0)	TBPe	1%	5.4	2.7	11	8.2	4.1	14.2	7.5	2.3	13.6	10	5	0.14	0.17	2020	
ITO/HATCN/TAPC/TCTA/DPEPO or DMAC-DPS:TBPe/TmPyPB/LiF/Al	DPEPO (6.1,2.0)	TBPe	1%	5.4	2.7	9.7	8	4.9	14.4	8.5	3.5	12.9	10.3	6.2	0.14	0.17	2020	
ITO/MoO3/TAPC/TCTA/mCP/DPEPO:emitter/DPEPO/Bphen/LiF/Al	DPEPO (6.1,2.0)	TDBA-SAF	20%	4.96	1.58	28.2	24.2	17.6	19.9	14.2	7.4	23.7	20.6	14.9	0.142	0.09	2020	[406]
ITO/MoO3/TAPC/TCTA/mCP/DPEPO:emitter/DPEPO/Bphen/LiF/Al	DPEPO (6.1,2.0)	DBA-SAB	20%	4.91	1.68	25.7	25.2	23.6	35.1	32.1	20.2	43.8	42.4	37.8	0.144	0.212	2020	

ITO/MoO3/mCP/DPEPO/host:FIrpic/DPEPO/TPBi/LiF/Al	mSOAD (5.8.2.8) Host work	Firpic	6%	-	-	16.7	-	8	32.46	-	-	36.19	-	-	0.19	0.36	2020	[407]
ITO/HATCN/TAPC/DCDPA/DBFPO:dopant/DBFPO/TPBi/LiF/Al	DPEPO (6.1,2.0)	TPDICz	20%	5.34	2.39	16.9	-	3.5	-	-	-	21.4	-	-	0.14	0.14	2020	[408]
ITO/TAPC/TCTA/emitter:DPEPO/TmPyPB/LiF/Al	DPEPO (6.1,2.0)	3CbzPYR	10%	5.52	2.37	19.7	-	-		-	-	-	-	-	0.16	0.23	2020	[409]
ITO/NPB/mCP/DPEPO:tACR:DBO/TAZ/LiF/Al	DPEPO (6.1,2.0)	tACR-DBO	1%	5.2	2.3	9.9	-	-	7.7	-	-	8.6	-	-	0.152	0.088	2020	[410]
ITO/HAT-CN/NPB/TCTA/mCBP/BCz-TRZ:host/PPT/ BePP2:Li2CO3/Li2CO3/Al	PyD2 (5.6,2.1)	BCz-TRZ	30%	-	-	19.6	-	-	-	-	-	-	-	-	0.16	0.3	2020	[411]
ITO/HAT-CN/NPB/TCTA/mCBP/BCz-TRZ:host/PPT/BePP2:Li2CO3/Li2CO3/Al	PyD2 (5.6,2.1)	PyBCz-TRZ	-	-	-	17.3	-	-	-	-	-	-	-	-	0.15	0.24	2020	

ITO/HAT-CN/NPB/TCTA/mCBP/BCz-TRZ:host/PPT/BePP2:Li2CO3/Li2CO3/Al	PyD2 (5.6,2.1)	ICz-TRZ	-	-	-	18.1	-	-	-	-	-	-	-	-	0.15	0.23	2020	
ITO/HAT-CN/NPB/TCTA/mCBP/BCz-TRZ:host/PPT/BePP2:Li2CO3/Li2CO3/Al	mCBP(5.6,2.0)	PyBCz-TRZ	-	-	-	10	-	-	-	-	-	-	-	-	0.14	0.2	2020	
ITO/HAT-CN/NPB/TCTA/mCBP/BCz-TRZ:host/PPT/BePP2:Li2CO3/Li2CO3/Al	mCBP(5.6,2.0)	ICz-TRZ	-	-	-	11.8	-	-	-	-	-	-	-	-	0.14	0.19	2020	
ITO/MoO3/mCP/DMAC-DPS/ETM/ETM/Liq/Al	A3	DMAC-DPS	30 %	5.9	2.9	22.3	-	-	-	-	-	-	-	-	0.15	0.15	2020	[412]
ITO/MoO3/mCP/DMAC-DPS/ETM/ETM/Liq/Al	PPT	DMAC-DPS	30 %	5.9	2.9	14.9	-	-	-	-	-	-	-	-	0.14	0.2	2020	
ITO/MoO3/mCP/DMAC-DPS/ETM/ETM/Liq/Al	DPEPO (6.1,2.0)	DMAC-DPS	30 %	5.9	2.9	23.1	-	-	-	-	-	-	-	-	0.14	0.16	2020	
ITO/MoO3/mCP/DMAC-DPS/ETM/ETM/Liq/Al	A1	DMAC-DPS	30 %	5.9	2.9	18.9	-	-	-	-	-	-	-	-	0.14	0.18	2020	
ITO/MoO3/mCP/DMAC-DPS/ETM/ETM/Liq/Al	A2	DMAC-DPS	30 %	5.9	2.9	22.4	-	-	-	-	-	-	-	-	0.14	0.18	2020	
ITO/MoO3/mCP/DMAC-DPS/ETM/ETM/Liq/Al	A3	DMAC-DPS	30 %	5.9	2.9	21.9	-	-	-	-	-	-	-	-	0.14	0.17	2020	

ITO/MoO3/mCP/DMAC-DPS/ETM/ETM/Liq/Al	A4	DMAC-DPS	30%	5.9	2.9	14.2	-	-	-	-	-	-	-	-	0.15	0.21	2020	
ITO/MoO3/mCP/DMAC-DPS/ETM/ETM/Liq/Al	A5	DMAC-DPS	30%	5.9	2.9	7.9	-	-	-	-	-	-	-	-	0.19	0.36	2020	
ITO/ TAPC/ TCTA/ emitter:mCP/DPEPO/TmPyPB/LiF/Al	mCP(6.1,2.4)	*t*Cz-ND	7%	5.6	2.7	17	9.9	7	19.88	-	-	22.17	-	-	0.14	0.16	2020	[413]
ITO/TAPC/TCTA/emitter:mCP/DPEPO/TmPyPB/LiF/Al	mCP(6.1,2.4)	metCz-ND	7%	5.6	2.7	17.6	14.4	19.8	23.6	-	-	31.98	-	-	0.18	0.32	2020	
ITO/HAT-CN/HAT-CN:TAPC/TCTA:CZ-MPS/CzSi:CZ-MPS/Tm3PyP26PyB/LiF/Al	TCTA	CZ-MPS	10%	5.6	2.3	9.3	-	-	-	-	-	3.4	-	-	0.175	0.065	2020	[414]
ITO/HAT-CN/TAPC/mCP/emitter:DPEPO/TSPO1/TPBi/LiF/Al	DPEPO (6.1,2.0)	Cz-ND	20%	5.72	2.76	15.3	-	-	16.5	-	-	19.9	-	-	0.15	0.17	2020	[415]
ITO/HAT-CN/TAPC/mCP/emitter: DPEPO/TSPO1/TPBi/LiF/Al	DPEPO (6.1,2.0)	tBuCz-ND	20%	5.62	2.71	20.9	-	-	26.5	-	-	35.9	-	-	0.15	0.22	2020	
ITO/HATCN/TAPC/mCP:emitter/DPEPO/TmPyPb/LiF/Al	DPEPO (6.1,2.0)	4	8%	-	-	15.5	-	-	10.1	-	-	13.9	-	-	0.18	0.25	2020	[416]

ITO/HATCN/TAPC/mCP:emitter/DPEPO/TmPyPb/LiF/Al	DPEPO (6.1,2.0)	5	8%	-	-	10.8	-	-	6.1	-	-	15	-	-	0.16	0.2	2020	
ITO/HATCN/TAPC/mCP:emitter/DPEPO/TmPyPb/LiF/Al	DPEPO (6.1,2.0)	6	8%	-	-	17.6	-	-	12	-	-	16	-	-	0.16	0.19	2020	
ITO/HAT-CN/TAPC/CCP/EML/PPF/B3PyPB/Liq/Al	-	TMCz-BO	5%	-	-	20.7	20.2	17.4	12	-	-	29.8	-	-	0.14	0.18	2020	[417]
ITO/HAT-CN/TAPC/CCP/EML/PPF/Liq/Al	-	TMCz-3P	5%	-	-	20.4	18.3	12.8	31.2	-	-	37.8	-	-	0.14	0.26	2020	
ITO/HATCN/NPB/PCZAC/host:DBA-DI/DDBFT/p-bPPhenB/LiF/Al	mCBP-CN	DBA-DI	30%	5.51	2.71	23.1	22.8	-	40.3	-	-	-	-	-	0.15	0.33	2020	[418]
ITO/HATCN/NPB/PCZAC/host:DBA-DI/DDBFT/p-bPPhenB/LiF/Al	mCBP-CN: DDBFT	DBA-DI	30%	5.51	2.71	28.1	27.8	-	-	-	-	-	-	-	0.16	0.39	2020	
ITO/NPB/TCTA/EML/TSPO1/TPBi/LiF/Al	DCzDPy	4CzDBN	5%	5.79	3.15	18.3	14	11.9	33.6	-	-	39.8	-	-	0.19	0.36	2020	[419]
ITO/HATCN/Spiro-TPD/HMTPD/mCP/EML/PPT/Liq:PPT/Liq/Al	PPT	PyCF3BN	15%	-	-	19.5	18	14.6	29.9	19	11.9	44.4	40.4	32.4	0.18	0.38	2020	[420]

ITO/MoO3/mCP/9ArFDBFxPO:emitter/DBFDPO/LiF/Al	-	-	-	-	-	-	29.6	16.7	29.9	-	-	-	-	-	0.17	0.21	2020	[421]
ITO/MoO3/NPB/mCP/9ArFDBFxPO:emitter/DBFDPO/LiF/Al	-	-	-	-	-	-	23.3	20.2	-	-	-	-	-	-	0.18	0.22	2020	
ITO/MoO3/NPB/mCP/9ArFDBFxPO:emitter/DBFDPO/LiF/Al	-	-	-	-	-	-	22.3	13.4	-	-	-	-	-	-	0.16	0.19	2020	
ITO/MoO3/NPB/mCP/9ArFDBFxPO:emitter/DBFDPO/LiF/Al	-	-	-	-	-	-	26	15.6	-	-	-	-	-	-	0.16	0.2	2020	
ITO/HATCN/BPBPA/mCBP/mCBP:X-Host5CzCN/DBFTrZ/ZADN/Liq/Al	-	-	-	6.67	2.97	17.3	-	-	-	-	-	-	-	-	0.17	0.35	2020	[422]
ITO/HATCN/BPBPA/mCBP/mCBP: X-Host-5CzCN/DBFTrZ/ZADN/Liq/Al	-	-	-	6.8	3.13	14.8	-	-	-	-	-	-	-	-	0.17	0.35	2020	
ITO/HATCN/BPBPA/mCBP/mCBP: X-Host-5CzCN/DBFTrZ/ZADN/Liq/Al	-	-	-	6.69	2.95	12.9	-	-	-	-	-	-	-	-	0.17	0.35	2020	
ITO/HATCN/BPBPA/mCBP/mCBP: X-Host-5CzCN/DBFTrZ/ZADN/Liq/Al	-	-	-	6.85	3.14	12.2	-	-	-	-	-	-	-	-	0.17	0.35	2020	
ITO/TPBi/TPAATPE or PPIATPE/TPBi/LiF/Al	-	-	-	5.34	2.8	6.97	-	5.96	8.8	-	4.8	9.4	-	7.7	0.15	0.16	2020	[423]
ITO/TPBi/TPAATPE or PPIATPE/TPBi/LiF/Al	-	-	-	5.6	2.78	6.1	-	5	7.3	-	3.2	7.9	-	6.3	0.16	0.16	2020	

ITO/TAPC/mcp/DspiroAc-TRZ/TmPyPb/LiF/Al	-	DspiroAc-TRZ	-	5.31	2.94	25.7	22.4	16.4	47.7	37.5	22.4	55.8	51	35.7	0.175	0.375	2020	[424]
ITO/ReO3:SimCP/SimCP/EML/oCF3-T2T/CN-T2T/Liq/Al	-	3CzIPN-H-CF3	10	5.66	2.27	19.7	-	17.9	23.6	-	18.8	31.5	-	28.6	0.16	0.22	2020	[425]
ITO/NPB/mcp/DPEPO: Eimtter/DPEPO/LiF/Al	DPEPO	TrSAc	10	5.35	2.85	23.8	-	-	40.9	-	-	50.1	-	-	0.19	0.36	2020	[426]
ITO/NPB/mcp/DPEPO: Eimtter/DPEPO/LiF/Al	DPEPO	moTrSAc	10	5.35	2.75	21.3	-	-	28.7	-	-	36.6	-	-	0.17	0.27	2020	
ITO/NPB/mcp/DPEPO:Eimtter/DPEPO/LiF/Al	DPEPO	tmTrSAc	10	5.35	2.75	15.5	-	-	21.3	-	-	28.5	-	-	0.17	0.27	2020	
ITO/NPB/mcp/DPEPO: Eimtter/DPEPO/LiF/Al	DPEPO	motmTrSAc	10	5.35	2.65	19.5	-	-	21.8	-	-	29.2	-	-	0.16	0.22	2020	

Table S6. A comprehensive list of wet-processed blue TADF OLED devices, including the publication year, device structure, key dopant, doping concentration, employed host, HOMO, LUMO, EQE, PE, CE, CIE (X), CIE (Y), and reference. The years span from 2016 to 2020.

**Device Structure**	**Host**	**Emitter**	**EQE (%)**	**PE (lm/W)**	**CE (cd/A)**	**CIE**	**Year**	**Ref.**

		Name	Conc. (wt%)	HOMO	LUMO	Max	100	1000	Max	100	1000	Max	100	1000	(X)	(Y)		
							(cdm^{-2})			(cdm^{-2})			(cdm^{-2})					
ITO/PEDOT:PSS/tbCz-SO or poCz-SO/TmPyPB/Cs2CO3/Al	-	tbCz-So	100	5.5	2.3	2.6	1.4	-	-	-	-	4	2.1	-	0.16	0.19	2016	[427]
ITO/PEDOT:PSS/tbCz-SO or poCz-SO/TmPyPB/Cs2CO3/Al	-	poCz-So	100	5.6	2.4	6.2	5.5	-	-	-	-	10.5	9.5	-	0.18	0.27	2016	
ITO/PEDOT:PSS/EML/TPBI/Liq/Al	-	DMAC-DPS	100%	5.92	2.92	7	4.4	1.6	10.1	5.2	1.3	11.4	9	3.4	0.18	0.31	2016	[428]
ITO/PEDOT:PSS/EML/TPBI/Liq/Al	-	DCzDMAC-DPS	100%	5.2	2.36	2.2	1.5	-	2	1.2	-	3.8	2.7	-	0.18	0.27	2016	
ITO/PEDOT:PSS/TAPC/mCP/mCP:BisCz-Trz)/TSPO1/TPBi/LiF/Al	mCP	BisCz-Trz	3%	6.1	3.53	12.2	-	-	6.5	-	-	18.5	-	-	0.15	0.2	2017	[429]
ITO/PEDOT:PSS/TAPC/mCP/mCP:BisCz-Trz)/TSPO1/TPBi/LiF/Al	mCP	trisCz-Trz	7%	6.11	3.33	16.5	-	-	12.3	-	-	23.9	-	-	0.15	0.22	2017	
ITO/PEDOT:PSS/mCP:TSPO1/BmPyPb/LiF/Al	-	DTPDDA	10%	5.57	2.8	4.43	-	-	3.13	-	-	7.46	-	-	0.15	0.26	2018	[430]

ITO/PEDOT:PSS/TAPC/mCP/EML/TSPO1/TBPi/LiF/Al	-	CzTrZ	-	6.04	3.39	4.2	-	0.6	2.7	-	0.1	3.8	-	0.53	0.17	0.11	2018	[431]
ITO/PEDOT:PSS/m-ACSO2/ETLs/Liq/Al	CzSi	o-ACSO2	10%	5.33	2.44	8.7	7.9	-	7.5	6.1	-	19	17.2	-	0.2	0.34	2018	[432]
ITO/PEDOT:PSS/m-ACSO2/ETLs/Liq/Al	CzSi	m-ACSO2	10%	5.36	2.26	9.7	8.3	-	9.1	6.3	-	19.2	16.3	-	0.19	0.3	2018	
ITO/PEDOT:PSS/m-ACSO2/ETLs/Liq/Al	CzSi	DMAC-DPS	10%	5.33	2.32	8.9	8.9	-	4.8	4.8	-	13.8	13.7	-	0.16	0.21	2018	
ITO/PEDOT:PSS/NPB/mCP/SBABz4:DPEPO/DPEPO/TPBi/Ca/Al	DPEPO	SBABz4	10%	-	-	6.8	-	-	-	-	-	-	-	-	0.16	0.09	2019	[433]
ITO/PEDOT:PSS/mCP:emitter/TSPO1/LiF/Al	mCP	TSXA	1%	-	-	6.34	-	-	5.39	-	-	12.41	-	-	0.16	0.32	2019	[434]
ITO/PEDOT:PSS/mCP:emitter/TSPO1/LiF/Al	mCP	TTSA	1%	-	-	14.94	-	-	13.94	-	-	29.29	-	-	0.16	0.33	2019	
ITO/ZnO/PEIE:Rb2CO3/EML/TCTA/MoO3/Al	-	SDPA-4PhCz	-	5.74	2.49	4.92	-	-	1.12	-	-	1.15	-	-	0.15	0.04	2019	[435]
ITO/PEDOT:PSS/mCP/DPEPO:Emitter/TSPO1/TPBI/LiF/Al	-	DCz-mPym	-	5.65	2.53	6.72	-	-	3.91	-	-	4.99	-	-	0.15	0.11	2019	[436]
ITO/PEDOT:PSS/mCP/DPEPO:Emitter/TSPO1/TPBI/LiF/Al	-	DtBCz-mPym	-	5.5	2.52	10.9	-	-	9.67	-	-	12.3	-	-	0.15	0.15	2019	

ITO/PEDOT:PSS/CzAcSF:emitters/DPEPO/TmPyPB/Liq/Al	CzAcSF	2tDMG	10%	5.12	2.37	16.2	-	7.4	20.1	-	6.4	37.5	-	17.2	0.22	0.37	2020	[437]
ITO/HATCN/TCTA/EML/TSPO1/TPBi/LiF/Al	mCP	PTZTPE-1	-	5.48	2.6	3.9	-	-	-	-	-	-	-	-	0.16	0.1	2020	[438]
ITO/HATCN/TCTA/EML/TSPO1/TPBi/LiF/Al	mCP	PTZTPE-3	-	5.56	3.24	8.2	-	-	-	-	-	-	-	-	0.28	0.38	2020	
ITO/HATCN/TCTA/EML/TSPO1/TPBi/LiF/Al	mCP	PTZTPE-4	-	5.49	2.95	0.7	-	-	-	-	-	-	-	-	0.17	0.21	2020	
ITO/TAPC/TCTA/emitter:mCP/DPEPO/TmPyPB/LiF/Al	mCP	tCz-ND	7%	5.6	2.7	13.5	12.1	7.8	10.24	-	-	17.26	-	-	0.15	0.14	2020	[413]
ITO/TAPC/TCTA/emitter:mCP/DPEPO/TmPyPB/LiF/Al	mCP	MetCZ-ND	7%	5.6	2.7	11.7	7.5	11	11.76	-	-	23.84	-	-	0.16	0.29	2020	
ITO/PEDOT: PSS/host:5TCzBN/TPBI/Cs2CO3/Al	-	3CzPhCN-O-Cz	60%	5.27	1.69	4.63	-	-	6.76	-	-	10.75	-	-	0.18	0.39	2020	[439]
ITO/PEDOT: PSS/host:5TCzBN/TPBI/Cs2CO3/Al	-	3PhCzCN-O-Cz	60%	5.24	1.94	1.05	-	-	1.09	-	-	2.43	-	-	0.2	0.36	2020	
ITO/PEDOT:PSS/PVK/nondoped emitter (TB-3Cz, TB-P3Cz, or TB-DACz)/TPBi/LiF/Al	-	TB-3Cz	0.30%	5.15	1.93	9.19	-	-	3.76	-	-	4	-	-	0.17	0.07	2020	[440]

ITO/PEDOT:PSS/PVK/nondoped emitter (TB-3Cz, TB-P3Cz, or TB-DACz)/TPBi/LiF/Al	-	TB-P3Cz	0.20%	4.88	1.72	6.13	-	-	4.12	-	-	4.59	-	-	0.15	0.08	2020	
ITO/PEDOT:PSS/PVK/nondoped emitter (TB-3Cz, TB-P3Cz, or TB-DACz)/TPBi/LiF/Al	-	TB-DACz	0.10%	4.62	1.76	6.04	-	-	15.7	-	-	15	-	-	0.18	0.4	2020	
ITO/PEDOT:PSS/PVK/EML/DPEPO/TmPyPb/LiF/Al	-	2tCz2CzBn	-	5.65	2.8	24.5	11.9	4.5	26.3	11.6	3.1	41.9	21	8.1	0.16	0.24	2020	
ITO/PEDOT:PSS/PVK/EML/DPEPO/TmPyPb/LiF/Al	-	2PhCz2tCzBn	-	5.71	2.96	19.5	17.6	7.6	26.7	23.8	8.1	42.5	38.3	16.9	0.18	0.35	2020	[441]
ITO/PEDOT:PSS/PVK/EML/DPEPO/TmPyPb/LiF/Al	-	2tCz2PhCzBn	-	5.61	2.86	19.1	13.2	7.9	29.2	17.9	8	43.7	30.4	18.4	0.19	0.38	2020	
ITO/PEDOT:PSS/Emitter/PHPO/Cs2CO3/Al	-	MeCz-4CzCN	-	5.33	2.52	21.8	21.3	19	35.6	-	-	46.5	-	-	0.17	0.35	2020	
ITO/PEDOT:PSS/Emitter/PHPO/Cs2CO3/Al	-	PhCz-4CzCN	-	5.36	2.55	20.6	20	18.2	27.5	-	-	40.8	-	-	0.16	0.27	2020	[442]
ITO/PEDOT:PSS/Emitter/PHPO/Cs2CO3/Al	-	PhDCz-4CzCN	-	5.41	2.58	10	9.8	7.9	11.2	-	-	18.9	-	-	0.19	0.28	2020	

ITO/PEDOT:PSS/T-CNDF-T-tCz/DPEPO/TmPyPB/Liq/Al	DPEPO	T-CNDF-T-*t*Cz	-	-	-	21	-	-	20.8	-	-	46.4	-	-	0.19	0.35	2020	[443]
ITO/PEDOT:PSS/T-CNDF-T-tCz/DPEPO/TmPyPB/Liq/Al	DPEPO	S-CNDF-S-*t*Cz	-	-	-	2.6	-	-	1.4	-	-	3.6	-	-	0.16	0.18	2020	
ITO/PEDOT:PSS/T-CNDF-T-tCz/DPEPO/TmPyPB/Liq/Al	DPEPO	S-CNDF-D-*t*Cz	-	-	-	3.7	-	-	2.2	-	-	5	-	-	0.16	0.17	2020	

References

1. Blue Light-Emitting Devices Based on Molecular Glass Materials of Tetraphenylsilane. **2001**, 1637–1641.

2. Chan, L.; Lee, R.; Hsieh, C.; Yeh, H.; Chen, C. Optimization of High-Performance Blue Organic Light-Emitting Diodes Containing Tetraphenylsilane Molecular Glass Materials. **2002**, *78*, 6469–6479.

3. Benmansour, B.H.; Shioya, T.; Sato, Y.; Bazan, G.C. Anthracene-Containing Binaphthol Chromophores for Light-Emitting Diode (LED) Fabrication **. **2003**, 883–886, doi:10.1002/adfm.200304456.

4. Chen, C.; Chiang, C.; Lin, Y.; Chan, L.; Huang, C.; Tsai, Z.; Chen, C. Ortho-Substituent Effect on Fluorescence and Electroluminescence of Arylamino-Substituted Coumarin and Stilbene. **2003**, 2000–2003.

5. Shen, W.; Dodda, R.; Wu, C.; Wu, F.; Liu, T.; Chen, H.; Chen, C.H.; Shu, C. Spirobifluorene-Linked Bisanthracene : An Efficient Blue Emitter with Pronounced Thermal Stability. **2004**, 930–934.

6. Kim, B.Y.; Jeong, H.; Kim, S.; Yang, K.; Kwon, S. High-Purity-Blue and High-Efficiency Electroluminescent Devices Based on Anthracene **. **2005**, 1799–1805, doi:10.1002/adfm.200500051.

7. Tao, B.S.; Peng, Z.; Zhang, X.; Wang, P.; Lee, C.; Lee, S. Highly Efficient Non-Doped Blue Organic Light-Emitting Diodes Based on Fluorene Derivatives with High Thermal Stability **. **2005**, doi:10.1002/adfm.200500067.

8. Wen, S.; Lee, M.; Chen, C.H. Recent Development of Blue Fluorescent OLED Materials and Devices. **2005**, *1*, 90–99.

9. Fischer, A.; Forget, S.; Chénais, S.; Castex, M.; Adès, D.; Siove, A.; Denis, C.; Maisse, P.; Geffroy, B. Highly Efficient Multilayer Organic Pure-Blue-Light Emitting Diodes with Substituted Carbazoles Compounds in the Emitting Layer . **2006**, *1*, 4–5.

10. Tseng, R.J.; Chiechi, R.C.; Wudl, F.; Yang, Y. Highly Efficient 7,8,10-Triphenylfluoranthene-Doped Blue Organic Light-Emitting Diodes for Display Application. *Appl Phys Lett* **2006**, *88*, 093512, doi:10.1063/1.2167814.

11. Zhi, B.; Gao, Q.; Li, Z.H.; Xia, P.F.; Wong, M.S.; Cheah, K.W.; Chen, C.H. Efficient Deep-Blue Organic Light-Emitting Diodes : Arylamine- Substituted Oligofluorenes **. **2007**, 3194–3199, doi:10.1002/adfm.200700238.

12. Shih, B.P.; Chuang, C.; Chien, C.; Diau, E.W.; Shu, C. Highly Efficient Non-Doped Blue-Light-Emitting Diodes Based on an Anthrancene Derivative End-Capped with Tetraphenylethylene Groups **. **2007**, 3141–3146, doi:10.1002/adfm.200700355.

13. Jou, J.H.; Chiang, P.H.; Lin, Y.P.; Chang, C.Y.; Lai, C.L. Hole-Transporting-Layer-Free High-Efficiency Fluorescent Blue Organic Light-Emitting Diodes. *Appl Phys Lett* **2007**, *91*, 043504, doi:10.1063/1.2759984.

14. Lin, S.L.; Chan, L.H.; Lee, R.H.; Yen, M.Y.; Kuo, W.J.; Chen, C.T.; Jeng, R.J. Highly Efficient Carbazole-π-Dimesitylborane Bipolar Fluorophores for Nondoped Blue Organic Light-Emitting Diodes. *Advanced Materials* **2008**, *20*, 3947–3952, doi:10.1002/ADMA.200801023.

15. Seung, B.; Lee, J.; Park, J.S.; Yoon, K.; Kim, Y.; Jin, S.; Kang, S.K.; Gal, Y.; Kang, S.; Lee, J.Y.; et al. High-Efficiency Deep-Blue Light-Emitting Diodes Based on Phenylquinoline / Carbazole-Based Compounds **. **2008**, 3922–3930, doi:10.1002/adfm.200800697.

16. You, A.; Be, M.A.Y.; In, I. High Efficiency Deep-Blue Organic Light- Emitting Diode with a Blue Dye in Low- Polarity Host. **2008**, *193314*, 2–5, doi:10.1063/1.2931071.

17. Liao, S.H.; Shiu, J.R.; Liu, S.W.; Yeh, S.J.; Chen, Y.H.; Chen, C.T.; Chow, T.J.; Wu, C.I. Hydroxynaphthyridine-Derived Group III Metal Chelates: Wide Band Gap and Dee Blue Analogues of Green Alq (Tris(8-Hydroxyquinolate)Aluminum) and Their Versatile Applications for Organic Light-Emitting Diodes. *J Am Chem Soc* **2009**, *131*, 763–777, doi:10.1021/JA807284E/SUPPL_FILE/JA807284E_SI_002.PDF.

18. Kim, S.; Yang, B.; Ma, Y.; Lee, J.; Park, J. Exceedingly Efficient Deep-Blue Electroluminescence from New Anthracenes Obtained Using Rational Molecular Design †. **2008**, 3376–3384, doi:10.1039/b805062g.

19. Liu, Y.; Tao, X.; Wang, F.; Dang, X.; Zou, D.; Ren, Y. Efficient Non-Doped Pure Blue Organic Light-Emitting Diodes Based on an Asymmetric Phenylenevinylene with Cis Double Bond. **2008**, *9*, 609–616, doi:10.1016/j.orgel.2008.03.011.

20. Chien, B.C.; Chen, C.; Hsu, F.; Shu, C. Multifunctional Deep-Blue Emitter Comprising an Anthracene Core and Terminal Triphenylphosphine Oxide Groups. **2009**, 560–566, doi:10.1002/adfm.200801240.

21. You, A.; Be, M.A.Y.; In, I. Efficient Blue and White Organic Light- Emitting Devices Based on a Single Bipolar Emitter. **2013**, *013507*, doi:10.1063/1.2742289.

22. Tao, S.; Zhou, Y.; Lee, C.; Zhang, X.; Lee, S. High-Efficiency Nondoped Deep-Blue-Emitting Organic Electroluminescent Device. **2010**, 2138–2141, doi:10.1021/cm100100w.

23. Jeon, Y.; Lee, J.; Kim, J.; Lee, C.; Gong, M. Deep-Blue OLEDs Using Novel Efficient Spiro-Type Dopant Materials. *Org Electron* **2010**, *11*, 1844–1852, doi:10.1016/j.orgel.2010.08.007.

24. Wei, B. Bin; Liu, J.; Zhang, Y.; Zhang, J.; Peng, H.; Fan, H.; He, Y.; Gao, X. Stable , Glassy , and Versatile Binaphthalene Derivatives Capable of Efficient Hole Transport , Hosting , and Deep- Blue Light Emission. **2010**, 2448–2458, doi:10.1002/adfm.201000299.

25. Zheng, C.; Zhao, W.; Wang, Z.; Huang, D.; Ye, J.; Ou, X.; Zhang, X.; Lee, C.; Lee, S. Highly Efficient Non-Doped Deep-Blue Organic Light-Emitting Diodes Based on Anthracene Derivatives. **2010**, 1560–1566, doi:10.1039/b918739a.

26. Hoon, J.; Hee, K.; Min, B.; Ryong, J.; Jin, S.; Keun, J.; Soo, S.; Kwan, Y. High-Efficiency Deep-Blue Organic Light-Emitting Diodes Using Dual-Emitting Layer. *Org Electron* **2010**, *11*, 1605–1612, doi:10.1016/j.orgel.2010.07.012.

27. Ong, S.; Hee, K.; Young, G.; Hoon, J.; Kwan, Y.; Soo, S. A Highly Efficient Deep Blue Fluorescent OLED Based on Diphenylaminofluorenylstyrene-Containing Emitting Materials. *Synth Met* **2010**, *160*, 1259–1265, doi:10.1016/j.synthmet.2010.03.020.

28. Chem, J.M.; Kim, S.H.; Cho, I.; Sim, M.K.; Park, S.; Park, S.Y. Highly Efficient Deep-Blue Emitting Organic Light Emitting Diode Based on the Multifunctional Fluorescent Molecule Comprising Covalently Bonded Carbazole and Anthracene Moieties †. **2011**, 9139–9148, doi:10.1039/c1jm11111f.

29. Peng, T.; Ye, K.; Liu, Y.; Wang, L.; Wu, Y.; Wang, Y. Novel Beryllium Complex as the Non-Doped Emitter for Highly Efficient Deep-Blue Organic Light-Emitting Diode. *Org Electron* **2011**, *12*, 1914–1919, doi:10.1016/j.orgel.2011.08.006.

30. Chem, J.M.; Zhang, Y.; Lai, S.; Tong, Q.; Chan, M.; Ng, T.; Wen, Z. Synthesis and Characterization of Phenanthroimidazole Derivatives for Applications in Organic Electroluminescent Devices †. **2011**, 8206–8214, doi:10.1039/c1jm10326a.

31. Zhuang, S.; Shangguan, R.; Jin, J.; Tu, G.; Wang, L.; Chen, J.; Ma, D.; Zhu, X. Efficient Nondoped Blue Organic Light-Emitting Diodes Based on Phenanthroimidazole-Substituted Anthracene Derivatives. *Org Electron* **2012**, *13*, 3050–3059, doi:10.1016/j.orgel.2012.08.032.

32. Lee, I.; Seo, J.; Gong, M. Deep Blue Fluorescent Host Materials Based on a Novel Spiro [Benzo [c] Fluorene-7 , 9 ' -Fluorene] Core Structure with Side Aromatic Wings. **2012**, *33*, 2287–2294.

33. Li, W.; Liu, D.; Shen, F.; Ma, D.; Wang, Z.; Feng, T.; Xu, Y.; Yang, B.; Ma, Y. A Twisting Donor-Acceptor Molecule with an Intercrossed Excited State for Highly Efficient, Deep-Blue Electroluminescence. *Adv Funct Mater* **2012**, *22*, 2797–2803, doi:10.1002/ADFM.201200116.

34. Chou, H.; Chen, Y.; Hsu, H.; Chang, W.; Chen, Y. Synthesis of Diimidazolylstilbenes as N-Type Blue Fluorophores : Alternative Dopant Materials for Highly Effi Cient Electroluminescent Devices. **2012**, 5867–5871, doi:10.1002/adma.201202222.

35. Chem, J.M.; Park, H.; Lee, J.; Kang, I.; Chu, Y.; Lee, J.; Kwon, S.; Kim, Y. Highly Rigid and Twisted Anthracene Derivatives : A Strategy for Deep Blue OLED Materials with Theoretical Limit Efficiency. **2012**, 2695–2700, doi:10.1039/c2jm16056k.

36. Chem, J.M. Highly Efficient Deep-Blue Electroluminescence Based on the Triphenylamine- Cored and Peripheral Blue Emitters with Segregative HOMO – LUMO. **2012**, 4401–4408, doi:10.1039/c1jm14639d.

37. Kamino, B.A.; Chang, Y.L.; Lu, Z.H.; Bender, T.P. Phthalonitrile Based Fluorophores as Fluorescent Dopant Emitters in Deep-Blue OLEDs: Approaching the NTSC Standard for Blue. *Org Electron* **2012**, *13*, 1479–1485, doi:10.1016/J.ORGEL.2012.04.014.

38. Jou, J.; Tseng, J.; Tseng, K.; Wang, W.; Jou, Y.; Shen, S.; Chen, Y.; Hung, W.; Chen, S.; Ding, T.; et al. High-Efficiency Host Free Deep-Blue Organic Light-Emitting Diode with Double Carrier Regulating Layers. *Org Electron* **2012**, *13*, 2893–2897, doi:10.1016/j.orgel.2012.08.040.

39. He, S.J.; Wang, Z.B.; Wang, D.K.; Jiang, N.; Lu, Z.H. Highly Efficient Blue Fluorescent Organic Light-Emitting Diodes with a High Emitter/Host Ratio. *Appl Phys Lett* **2013**, *103*, 083301, doi:10.1063/1.4819155.

40. Wang, K.; Zhao, F.; Wang, C.; Chen, S.; Chen, D. High-Performance Red , Green , and Blue Electroluminescent Devices Based on Blue Emitters with Small Singlet – Triplet Splitting and Ambipolar Transport Property. **2013**, 2672–2680, doi:10.1002/adfm.201202981.

41. Lee, Y.; Wu, T.; Liaw, C.; Wen, T.; Feng, S.; Lee, J.; Wu, Y.; Guo, T. Non-Doped Active Layer , Benzo [k] Fluoranthene-Based Linear Acenes , for Deep Blue- to Green-Emissive Organic Light-Emitting Diodes. *Org Electron* **2013**, *14*, 1064–1072, doi:10.1016/j.orgel.2013.01.021.

42. Liu, X.; Zheng, C.; Lo, M.; Xiao, J.; Chen, Z.; Liu, C.; Lee, C.; Fung, M.; Zhang, X. Novel Blue Fluorophor with High Triplet Energy Level for High Performance Single-Emitting-Layer Fluorescence and Phosphorescence Hybrid White Organic Light-Emitting Diodes. **2013**, 3–8.

43. Li, Z.; Jiao, B.; Wu, Z.; Liu, P.; Ma, L.; Lei, X.; Wang, D.; Zhou, G.; Hu, H.; Hou, X. Fluorinated 9,9′-Spirobifluorene Derivatives as Host Materials for Highly Efficient Blue Organic Light-Emitting Devices. *J Mater Chem C Mater* **2013**, *1*, 2183–2192, doi:10.1039/C3TC00466J.

44. Fukagawa, H.; Shimizu, T.; Ohbe, N.; Tokito, S.; Tokumaru, K.; Fujikake, H. Anthracene Derivatives as Efficient Emitting Hosts for Blue Organic Light-Emitting Diodes Utilizing Triplet – Triplet Annihilation Spiro-FA. *Org Electron* **2012**, *13*, 1197–1203, doi:10.1016/j.orgel.2012.03.019.

45. Online, V.A. Extremely Deep Blue and Highly Efficient Non-Doped Organic Light Emitting Diodes Using an Asymmetric Anthracene Derivative with a Xylene Unit †. **2013**, 4664–4666, doi:10.1039/c3cc41441h.

46. Chen, S.; Wei, J.; Wang, K.; Wang, C.; Chen, D.; Liu, Y.; Wang, Y. Multifunctional Organic Materials †. **2013**, 6594–6602, doi:10.1039/c3tc31271b.

47. Yu, Y.; Wu, Z.; Li, Z.; Jiao, B.; Li, L.; Ma, L.; Wang, D.; Zhou, G.; Hou, X. Highly Efficient Deep-Blue Organic Electroluminescent Devices (CIE y ≈ 0.08) Doped with Fluorinated 9,9′-Bianthracene Derivatives (Fluorophores). *J Mater Chem C Mater* **2013**, *1*, 8117–8127, doi:10.1039/C3TC31743A.

48. Yang, X.H.; Zheng, S.J.; Chae, H.S.; Li, S.; Mochizuki, A.; Jabbour, G.E. Fluorescent Deep-Blue and Hybrid White Emitting Devices Based on a Naphthalene – Benzofuran Compound. **2023**, *14*, 2023–2028, doi:10.1016/j.orgel.2013.03.012.

49. Yue, S.; Zhang, S.; Zhang, Z.; Wu, Y.; Wang, P.; Guo, R.; Chen, Y.; Qu, D.; Wu, Q.; Zhao, Y.; et al. Improved Power Efficiency of Blue Fluorescent Organic Light-Emitting Diode with Intermixed Host Structure. *J Lumin* **2013**, *143*, 619–622, doi:10.1016/J.JLUMIN.2013.06.007.

50. Zhang, X.; Mo, B.; Liu, L.; Wang, H.; Chang, D.; Xu, J.; Wang, H.; Wei, B. Blue Organic Light-Emitting Diodes with 2-Methyl-9 , 10- Bis (Naphthalen-2-Yl) Anthracene as Hole Transport and Emitting Layer and the Impedance Spectroscopy Analysis. *Current Applied Physics* **2014**, *14*, 1460–1464, doi:10.1016/j.cap.2014.08.021.

51. Song, J.Y.; Lee, S.J.; Kim, Y.K.; Yoon, S.S. Highly Efficient Non-Doped Organic Light Emitting Diodes (OLEDs) Using Anthracene Derivatives with Triphenylsilane Unit. *Mater Res Bull* **2014**, *58*, 145–148, doi:10.1016/J.MATERRESBULL.2014.03.021.

52. Liang, H.; Wang, X.; Zhang, X.; Ge, Z.; Ouyang, X.; Wang, S. Efficient Tuning of Electroluminescence from Sky-Blue to Deep-Blue by Changing the Constitution of Spirobenzofluorene Derivatives. *Dyes and Pigments* **2014**, *108*, 57–63, doi:10.1016/J.DYEPIG.2014.04.019.

53. Kim, M.; Lee, C.; Gong, M. Dyes and Pigments New Spirobenzoanthracene Derivatives with Naphthylanthracene Core : Synthesis and Application in Sky-Blue Fl Uorescent Host Materials. *Dyes and Pigments* **2014**, *105*, 202–207, doi:10.1016/j.dyepig.2014.02.005.

54. Wu, T.; Chou, H.; Huang, P.; Cheng, C.; Liu, R. 3,6,9,12-Tetrasubstituted Chrysenes: Synthesis, Photophysical Properties, and Application as Blue Fluorescent OLED. **2014**.

55. Liu, Y.; Zhang, Q.; Song, X.; Li, J.; Tian, J.; Gao, C. Dianthranide Derivative for Efficient Deep Blue-Emitting Organic Electroluminescence Devices., doi:10.1088/0022-3727/48/40/405104.

56. Cha, J.R.; Lee, C.W.; Gong, M.S. All Fused Ring Spiro Host and Dopant Materials for Efficient Blue Fluorescent Organic Light-Emitting Diodes. *Dyes and Pigments* **2015**, *Complete*, 251–257, doi:10.1016/J.DYEPIG.2015.04.023.

57. Kim, G.W.; Lampande, R.; Choe, D.C.; Bae, H.W.; Kwon, J.H. Efficient Hole Injection Material for Low Operating Voltage Blue Fluorescent Organic Light Emitting Diodes. *Thin Solid Films* **2015**, *589*, 105–110, doi:10.1016/J.TSF.2015.04.076.

58. Liu, M.; Li, X.-L.; Cheng Chen, D.; Xie, Z.; Cai, X.; Xie, G.; Liu, K.; Tang, J.; Su, S.-J.; Cao, Y.; et al. Study of Configuration Differentia and Highly Efficient, Deep-Blue, Organic Light-Emitting Diodes Based on Novel Naphtho[1,2-d]Imidazole Derivatives. *Adv Funct Mater* **2015**, *25*, 5190–5198, doi:10.1002/ADFM.201502163.

59. Seok, Y.; Yoon, J.; Woo, H.; Kim, J.; Won, H.; Eun, S.; Kwan, Y.; Soo, S. Blue Fluorescent Materials Based on Bis (10-Phenylanthracen-9-Yl) Derivatives Containing Heterocyclic Moiety. *Opt Mater (Amst)* **2015**, *46*, 247–253, doi:10.1016/j.optmat.2015.04.027.

60. Lee, S.B.; Song, J.Y.; Yang, H.J.; Kim, Y.K.; Yoon, S.S. Blue Fluorescent Materials Composed of Anthracene-Aryl Amine-Anthracene Derivatives for Organic Light-Emitting Diodes. **2015**, *15*, 5238–5241, doi:10.1166/jnn.2015.10391.

61. Huang, Y.; Du, X.; Tao, S.; Yang, X.; Zheng, C.J.; Zhang, X.; Lee, C.S. High Efficiency Non-Doped Deep-Blue and Fluorescent/Phosphorescent White Organic Light-Emitting Diodes Based on an Anthracene Derivative. *Synth Met* **2015**, *203*, 49–53, doi:10.1016/J.SYNTHMET.2014.10.019.

62. Song, J.Y.; Park, S.N.; Lee, S.J.; Kim, Y.K.; Yoon, S.S. Novel Fluorescent Blue-Emitting Materials Based on Anthracene-Fluorene Hybrids with Triphenylsilane Group for Organic Light-Emitting Diodes. *Dyes and Pigments* **2015**, *114*, 40–46, doi:10.1016/J.DYEPIG.2014.11.003.

63. Chen, W.; Yuan, Y.; Wu, G.; Wei, H.; Ye, J.; Chen, M.; Lu, F.; Tong, Q.; Wong, F.; Lee, C. Molecular Modification on Bisphenanthroimidazole Derivative for Deep-Blue Organic Electroluminescent Material with Ambipolar Property and High Performance. *Org Electron* **2015**, *17*, 159–166, doi:10.1016/j.orgel.2014.11.024.

64. Liu, W.; Chen, Z.; Zheng, C.; Liu, X.; Wang, K.; Li, F.; Dong, Y.; Ou, X.; Zhang, X. A Novel Nicotinonitrile Derivative as an Excellent Multifunctional Blue Fluorophore for Highly Efficient Hybrid White Organic Light-Emitting Devices. **2015**, *3*, doi:10.1039/c5tc01415h.

65. Liu, D.; Du, M.; Chen, D.; Ye, K.; Zhang, Z.; Liu, Y.; Wang, Y. A Novel Tetraphenylsilane – Phenanthroimidazole Hybrid Host Material for Highly Efficient Blue Fluorescent , Green and Red Phosphorescent. **2015**, 4394–4401, doi:10.1039/c5tc00333d.

66. Chen, W.C.; Wu, G.F.; Yuan, Y.; Wei, H.X.; Wong, F.L.; Tong, Q.X.; Lee, C.S. A Meta-Molecular Tailoring Strategy towards an Efficient Violet-Blue Organic Electroluminescent Material. *RSC Adv* **2015**, *5*, 18067–18074, doi:10.1039/C4RA16954A.

67. Chen, Z.; Liu, X.; Zheng, C.; Ye, J.; Li, X.; Li, F. Emitting Diode Enabled by a New Blue Fluorophor †. **2015**, 4283–4289, doi:10.1039/c5tc00285k.

68. Liu, B.; Yuan, Y.; He, D.; Huang, D.; Luo, C.; Zhu, Z. High-Performance Blue OLEDs Based on Phenanthroimidazole Emitters via Substitutions at the C6- and C9-Positions for Improving Exciton Utilization. **2016**, 12130–12137, doi:10.1002/chem.201602122.

69. Heng, C.L.I.; Hang, M.I.N.Z.; Hen, X.U.E.C.; Ing, Q.L.I. Host Material for Highly Efficient Blue Fluorescent OLED. **2016**, *6*, 2545–2553.

70. Kim, C.; Park, S.N.; Lee, S.B.; Kim, Y.S.; Lee, H.W.; Kim, Y.K.; Yoon, S.S. Derivatives for Blue Fluorescent Organic Light-Emitting Diodes. **2016**, *16*, 1859–1862, doi:10.1166/jnn.2016.12013.

71. Kim, K.H.; Baek, J.Y.; Cheon, C.W.; Moon, C.K.; Sim, B.; Choi, M.Y.; Kim, J.J.; Kim, Y.H. Highly Efficient Non-Doped Deep Blue Fluorescent Emitters with Horizontal Emitting Dipoles Using Interconnecting Units between Chromophores. *Chemical Communications* **2016**, *52*, 10956–10959, doi:10.1039/C6CC05076J.

72. Ruan, Z.; Li, L.; Wang, C.; Xie, Y.; Hu, Q.; Peng, Q.; Ye, S. Tetraphenylcyclopentadiene Derivatives : Aggregation- Induced Emission , Adjustable Luminescence from Green to Blue , Efficient Undoped OLED Performance and Good Mechanochromic Properties. **2016**, 6623–6632, doi:10.1002/smll.201602285.

73. Jhulki, S.; Mishra, A.K.; Chow, T.J.; Moorthy, J.N. Carbo[5]Helicene versus Planar Phenanthrene as a Scaffold for Organic Materials in OLEDs: The Electroluminescence of Anthracene-Functionalized Emissive Materials. **2017**, 34–36, doi:10.1039/c7nj02233f.

74. Sun, W.; Zhou, N.; Xiao, Y.; Wang, S.; Li, X. A Novel Spiro [Acridine-9 , 9 ' -Fluorene] Derivatives Containing Phenanthroimidazole Moiety for Deep-Blue OLED Application. **2017**, 3069–3076, doi:10.1002/asia.201701292.

75. Qiu, X.; Shi, J.; Xu, X.; Lu, Y.; Sun, Q.; Xue, S.; Yang, W. Dyes and Pigments Tuning the Optoelectronic Properties of Phenothiazine-Based D – A- Type Emitters through Changing Acceptor Pattern. *Dyes and Pigments* **2017**, *147*, 6–15, doi:10.1016/j.dyepig.2017.07.064.

76. Chen, D.; Liu, K.; Li, X.; Li, B.; Liu, M.; Cai, X. Engineering the Excited-State Properties of Purely Organic Intramolecular and Intermolecular Charge Transfer Emitters towards High-Performance. **2017**, 10991–11000, doi:10.1039/c7tc04014h.

77. Kim, D.Y.; Kang, J.; Lee, S.E.; Kim, Y.K.; Yoon, S.S. Arylamine-Substituted 2-(9,9-Diethyl-2-Vinyl-9H-Fluoren-7-Yl)-1,4-Diphenyltriphenylene Derivatives for Blue Organic Light-Emitting Diodes. *J Nanosci Nanotechnol* **2017**, *17*, 7286–7291, doi:10.1166/JNN.2017.14746.

78. Jeong, S.; Hyun, S.; Young, D.; Kim, C.; Won, H.; Eun, S.; Kwan, Y.; Soo, S. Blue Organic Light-Emitting Diodes Based on Diphenylamino Dibenzo [g , p] Chrysene Derivatives. *Thin Solid Films* **2017**, *636*, 8–14, doi:10.1016/j.tsf.2017.05.011.

79. Li, K.; Liu, M.; Yang, S.; Chen, Y.; He, Y.; Murtaza, I. Dyes and Pigments Substitution Effect of Super Hydrophobic Units : A New Strategy to Design Deep Blue Fl Uorescent Emitters. **2017**, *139*, 747–755, doi:10.1016/j.dyepig.2016.12.034.

80. Thanikachalam, V.; Sarojpurani, E.; Jayabharathi, J.; Jeeva, P. Efficient Phenanthroimidazole-Styryl-Triphenylamine Derivatives for Blue OLEDs: A Combined Experimental and Theoretical Study. *New Journal of Chemistry* **2017**, *41*, 2443–2457, doi:10.1039/C6NJ03801H.

81. Fan, C.; Wang, X.; Luo, J. Blue Organic Light-Emitting Diodes Based on Terpyridine-Substituted Triphenylamine Chromophores. *Opt Mater (Amst)* **2017**, *64*, 489–495, doi:10.1016/j.optmat.2017.01.018.

82. Thanikachalam, V.; Jeeva, P.; Jayabharathi, J. Highly Efficient Non-Doped Blue Organic Light Emitting Diodes Based on a D–π–A Chromophore with Different Donor Moieties. *RSC Adv* **2017**, *7*, 13604–13614, doi:10.1039/C6RA28303A.

83. Li, C.; Wei, B.; Wang, S.; Qiao, M.; Ye, Z.; Dou, D.; Chen, M.; Peng, Y.; Shi, Y. Efficient Deep-Blue Electrofluorescence with an External Quantum Efficiency Beyond 10 % Efficient Deep-Blue Electrofluorescence with an External Quantum Efficiency Beyond 10 %. *iScience* **2018**, *9*, 532–541, doi:10.1016/j.isci.2018.10.026.

84. Zhang, G.; Xing, G.; Li, J.; Zhang, X.; Lang, J.; Zhao, L.; Wang, J.; Jiang, W. Tailored Deep Blue OLEDs Characteristics with Doping Engineering in Fluorescent Dyes Based on DMPPP: Towards Manufacturable Solid-State Lighting Building Blocks. *Journal of Materials Science: Materials in Electronics* **2018**, *29*, 18852–18858, doi:10.1007/S10854-018-0010-4.

85. Hou, M.; Wang, H.; Miao, Y.; Xu, H.; Guo, Z.; Chen, Z.; Liao, X.; Li, L.; Li, J.; Guo, K. Highly Efficient Deep-Blue Electroluminescence from a A-π-D-π-A Structure Based Fluoresence Material with Exciton Utilizing Efficiency above 25%. *ACS Appl Energy Mater* **2018**, *1*, 3243–3254, doi:10.1021/ACSAEM.8B00461/SUPPL_FILE/AE8B00461_SI_001.PDF.

86. Woo, S.J.; Kim, Y.; Kim, M.J.; Baek, J.Y.; Kwon, S.K.; Kim, Y.H.; Kim, J.J. Strategies for the Molecular Design of Donor-Acceptor-Type Fluorescent Emitters for Efficient Deep Blue Organic Light Emitting Diodes. *Chemistry of Materials* **2018**, *30*, 857–863, doi:10.1021/ACS.CHEMMATER.7B04437/ASSET/IMAGES/LARGE/CM-2017-04437C_0004.JPEG.

87. Takita, Y. Highly Efficient Deep-Blue Fluorescent Dopant for Achieving Low-Power OLED Display Satisfying BT . 2020 Chromaticity., doi:10.1002/jsid.634.

88. Cho, S.; Kim, C.; Lee, S.E.; Kim, Y.K.; Yoon, S.S. Naphthalene Derivatives End-Capped with 2-(Diphenylamino)-9,9-Diethylfluorenes for Blue Organic Light-Emitting Diodes. *J Nanosci Nanotechnol* **2017**, *18*, 1251–1255, doi:10.1166/JNN.2018.14890.

89. Tang, X.; Bai, Q.; Shan, T.; Li, J.; Gao, Y.; Liu, F.; Liu, H.; Peng, Q.; Yang, B.; Li, F.; et al. Efficient Nondoped Blue Fluorescent Organic Light-Emitting Diodes (OLEDs) with a High External Quantum Efficiency of 9.4% @ 1000 Cd M−2 Based on Phenanthroimidazole−Anthracene Derivative. *Adv Funct Mater* **2018**, *28*, 1705813, doi:10.1002/ADFM.201705813.

90. Jeong, S.; Kim, C.; Kim, S.H.; Kim, D.Y.; Lee, S.E.; Kim, Y.K.; Yoon, S.S.; Jeong, S.; Kim, C.; Kim, S.H.; et al. Blue Organic Light-Emitting Diodes Based on Spiro [Fluorene-Indeno] Pyridine Derivatives. *Molecular Crystals and Liquid Crystals* **2018**, *660*, 24–32, doi:10.1080/15421406.2018.1452867.

91. Park, J.; Jang, B.; Moon, Y.J.; Lee, H.; Kim, Y.K.; Yoon, S.S. Efficient Deep Blue Organic Light Emitting Diodes Based on [2,7,7,13,13-Pentamethyl-9-(10-Phenylanthracen-9-Yl)-7,13-Dihydrobenzo[5,6]-s-Indaceno[1,2-g]Quinoline] Derivatives. *https://doi.org/10.1080/15421406.2020.1743428* **2020**, *705*, 120–126, doi:10.1080/15421406.2020.1743428.

92. Xu, Y.; Liang, X.; Liang, Y.; Guo, X.; Hanif, M.; Zhou, J.; Zhou, X.; Wang, C.; Yao, J.; Zhao, R.; et al. Efficient Deep-Blue Fluorescent OLEDs with a High Exciton Utilization Efficiency from a Fully Twisted Phenanthroimidazole-Anthracene Emitter. *ACS Appl Mater Interfaces* **2019**, *11*, 31139–31146, doi:10.1021/ACSAMI.9B10823/SUPPL_FILE/AM9B10823_SI_003.CIF.

93. Peng, S.; Zhao, Y.; Fu, C.; Pu, X.; Zhou, L.; Huang, Y.; Lu, Z. Acquiring High-Performance Deep-Blue OLED Emitters through an Unexpected Blueshift Color-Tuning Effect Induced by Electron-Donating -OMe Substituents. *Chemistry* **2018**, *24*, 8056–8060, doi:10.1002/CHEM.201800974.

94. Park, J. Highly Efficient Dual-Core Derivatives with EQEs as High as 8.38% at High Brightness for OLED Blue Emitters. **2019**, 14709–14716, doi:10.1039/c9tc04603h.

95. Sheng, R.; Song, J.; Li, A.; Zhang, F.; Zhang, H.; Duan, Y.; Zheng, J.; Wang, Z.; Chen, P. High-Efficiency and Low Efficiency Roll-off in White Organic Light-Emitting Diodes Employing a Novel Blue Emitter. *Org Electron* **2019**, *75*, 105375, doi:10.1016/j.orgel.2019.105375.

96. Sohn, S.; Wu, X.; Hun, K.; Ahn, H.; Jung, S.; Kwon, S.; Kim, Y. Journal of Industrial and Engineering Chemistry Highly-Twisted Pyrene Derivative for Pure-Blue Organic Light Emitting Diodes. *Journal of Industrial and Engineering Chemistry* **2019**, *78*, 239–245, doi:10.1016/j.jiec.2019.06.007.

97. Liu, H.; Kang, L.; Li, J.; Liu, F.; He, X.; Ren, S.; Tang, X.; Lv, C.; Lu, P. Highly Efficient Deep-Blue Organic Light-Emitting Diodes Based on Pyreno[4,5-d]Imidazole-Anthracene Structural Isomers. **2019**, 10273–10280, doi:10.1039/c9tc02990g.

98. Bae, H.W.; Kim, G.W.; Lampande, R.; Park, J.H.; Ko, I.J.; Yu, H.J.; Lee, C.Y.; Kwon, J.H. Efficiency Enhancement in Fluorescent Deep-Blue OLEDs by Boosting Singlet Exciton Generation through Triplet Fusion and Charge Recombination Rate. *Org Electron* **2019**, *70*, 1–6, doi:10.1016/J.ORGEL.2019.03.018.

99. Manuscript, A. Materials Chemistry. **2019**, doi:10.1039/C8QM00678D.

100. Tan, Y.; Wang, Z.; Wei, C.; Liu, Z.; Bian, Z.; Huang, C. Nondoped Deep-Blue Fluorescent Organic Electroluminescent Device with CIEy = 0.06 and Low Efficiency Roll-off Based on Carbazole/Oxadiazole Derivatives. *Org Electron* **2019**, *69*, 77–84, doi:10.1016/J.ORGEL.2019.02.028.

101. Gusev, A.N.; Kiskin, M.A.; Braga, E. V.; Chapran, M.; Wiosna-Salyga, G.; Baryshnikov, G. V.; Minaeva, V.A.; Minaev, B.F.; Ivaniuk, K.; Stakhira, P.; et al. Novel Zinc Complex with an Ethylenediamine Schiff Base for High-Luminance Blue Fluorescent OLED Applications. *Journal of Physical Chemistry C* **2019**, *123*, 11850–11859, doi:10.1021/ACS.JPCC.9B02171/ASSET/IMAGES/LARGE/JP-2019-02171N_0002.JPEG.

102. Liu, F.; Tao, Y.; Li, J.; Liu, H.; He, X.; Du, C.; Tang, X. Efficient Non-Doped Blue Fluorescent Organic Light-Emitting Diodes Based on Anthracene – Triphenylethylene Derivatives. **2019**, 1004–1012, doi:10.1002/asia.201801867.

103. Li, Z.; Gan, G.; Ling, Z.; Guo, K.; Si, C.; Lv, X.; Wang, H.; Wei, B.; Hao, Y. Easily Available, Low-Cost 9,9′-Bianthracene Derivatives as Efficient Blue Hosts and Deep-Blue Emitters in OLEDs. *Org Electron* **2019**, *66*, 24–31, doi:10.1016/J.ORGEL.2018.12.010.

104. Kang, S.K.; Kim, C.; Lee, S.E.; Kim, Y.K.; Yoon, S.S. Diphenylaminoarylvinyl-Substituted Diphenylanthracenes for Organic Light-Emitting Diodes. *J Nanosci Nanotechnol* **2018**, *19*, 979–983, doi:10.1166/JNN.2019.15930.

105. Huang, C.C.; Xue, M.M.; Wu, F.P.; Yuan, Y.; Liao, L.S.; Fung, M.K. Deep-Blue and Hybrid-White Organic Light Emitting Diodes Based on a Twisting Carbazole-Benzofuro[2,3-b]Pyrazine Fluorescent Emitter. *Molecules* **2019**, *24*, doi:10.3390/MOLECULES24020353.

106. Jia, Y.; Zhang, Y.; Fan, S.; Wu, S.; Zhao, X.; Wang, S.; Li, X. A Novel Bipolar Carbazole/ Phenanthroimidazole Derivative for High Efficiency Nondoped Deep-Blue Organic Light-Emitting Diodes. *Org Electron* **2019**, *64*, 259–265, doi:10.1016/J.ORGEL.2018.10.036.

107. Wang, Y.; Yan, D. On Carbazole / Phenanthroimidazole Derivatives †. **2020**, 10185–10190, doi:10.1039/d0tc02594a.

108. Patil, V.V.; Lee, K.H.; Lee, J.Y. For Blue Fluorescent Organic Light-Emitting Diodes †. **2020**, 8320–8327, doi:10.1039/d0tc01268h.

109. Zheng, X.; Zhao, J.; Chen, X.; Cai, R.; Yang, G.; Zhu, J. Imidazo [1 , 2- a] Pyridine as an Electron Acceptor to Construct High-Performance Deep-Blue Organic Light-Emitting Diodes with Negligible Efficiency Roll-Off. **2020**, 8588–8596, doi:10.1002/chem.202000518.

110. Zhang, T.; Ye, J.; Luo, A.; Liu, D. Efficient Deep Blue Emitter Based on Carbazole-Pyrene Hybrid for Non-Doped Electroluminescent Device. *Opt Mater (Amst)* **2020**, *100*, 109632, doi:10.1016/j.optmat.2019.109632.

111. Liu, F.; Liu, H.; Tang, X.; Ren, S.; He, X.; Li, J.; Du, C.; Feng, Z.; Lu, P. Nano Energy Novel Blue Fluorescent Materials for High-Performance Nondoped Blue OLEDs and Hybrid Pure White OLEDs with Ultrahigh Color Rendering Index. *Nano Energy* **2020**, *68*, 104325, doi:10.1016/j.nanoen.2019.104325.

112. Liu, X.; Liu, W.; Dongyu, W.; Wei, X.; Wang, L.; Wang, H.; Miao, Y.; Xu, H.; Yu, J.; Xu, B. Deep-Blue Fluorescent Emitter Based on a 9,9-Dioctylfluorene Bridge with a Hybridized Local and Charge-Transfer Excited State for Organic Light-Emitting Devices with EQE Exceeding 8%. **2020**, 14117–14124, doi:10.1039/d0tc02941f.

113. Huang, C.; Zhen, C.G.; Siew, P.S.; Kian, P.L.; Chen, Z.K. Solution-Processable Polyphenylphenyl Dendron Bearing Molecules for Highly Efficient Blue Light-Emitting Diodes. *Org Lett* **2005**, *7*, 391–394, doi:10.1021/OL0478180/SUPPL_FILE/OL0478180SI20041025_024322.PDF.

114. Lai, W.Y.; Zhu, R.; Fan, Q.L.; Hou, L.T.; Cao, Y.; Huang, W. Monodisperse Six-Armed Triazatruxenes: Microwave-Enhanced Synthesis and Highly Efficient Pure-Deep-Blue Electroluminescence. *Macromolecules* **2006**, *39*, 3707–3709, doi:10.1021/MA060154K.

115. Lai, W.Y.; He, Q.Y.; Zhu, R.; Chen, Q.Q.; Huang, W. Kinked Star-Shaped Fluorene/ Triazatruxene Co-Oligomer Hybrids with Enhanced Functional Properties for High-Performance, Solution-Processed, Blue Organic Light-Emitting Diodes. *Adv Funct Mater* **2008**, *18*, 265–276, doi:10.1002/ADFM.200700224.

116. Wang, L.; Jiang, Y.; Luo, J.; Zhou, Y.; Zhou, J.; Wang, J.; Pei, J.; Cao, Y. Highly Efficient and Color-Stable Deep-Blue Organic Light-Emitting Diodes Based on a Solution-Processible Dendrimer. *Adv Mater* **2009**, *21*, 4854–4858, doi:10.1002/ADMA.200901039.

117. Zhang, M.; Xue, S.; Dong, W.; Wang, Q.; Fei, T.; Gu, C.; Ma, Y. Highly-Efficient Solution-Processed OLEDs Based on New Bipolar Emitters. *Chemical Communications* **2010**, *46*, 3923–3925, doi:10.1039/C001170C.

118. Zhu, M.; Ye, T.; Li, C.G.; Cao, X.; Zhong, C.; Ma, D.; Qin, J.; Yang, C. Efficient Solution-Processed Nondoped Deep-Blue Organic Light-Emitting Diodes Based on Fluorene-Bridged Anthracene Derivatives Appended with Charge Transport Moieties. *Journal of Physical Chemistry C* **2011**, *115*, 17965–17972, doi:10.1021/JP203892Q/ASSET/IMAGES/MEDIUM/JP-2011-03892Q_0002.GIF.

119. Zhen, C.G.; Dai, Y.F.; Zeng, W.J.; Ma, Z.; Chen, Z.K.; Kieffer, J. Achieving Highly Efficient Fluorescent Blue Organic Light-Emitting Diodes through Optimizing Molecular Structures and Device Configuration. *Adv Funct Mater* **2011**, *21*, 699–707, doi:10.1002/ADFM.201002165.

120. Huang, H.; Fu, Q.; Zhuang, S.; Liu, Y.; Wang, L.; Chen, J.; Ma, D.; Yang, C. Novel Deep Blue OLED Emitters with 1,3,5-Tri(Anthracen-10-Yl)Benzene-Centered Starburst Oligofluorenes. *Journal of Physical Chemistry C* **2011**, *115*, 4872–4878, doi:10.1021/JP110652Y/SUPPL_FILE/JP110652Y_SI_003.PDF.

121. Zhang, T.; Wang, R.; Ren, H.; Chen, Z.; Li, J. Deep Blue Light-Emitting Polymers with Fluorinated Backbone for Enhanced Color Purity and Efficiency. *Polymer (Guildf)* **2012**, *7*, 1529–1534, doi:10.1016/J.POLYMER.2012.02.010.

122. Liu, C.; Li, Y.; Zhang, Y.; Yang, C.; Wu, H.; Qin, J.; Cao, Y. Solution-Processed, Undoped, Deep-Blue Organic Light-Emitting Diodes Based on Starburst Oligofluorenes with a Planar Triphenylamine Core. *Chemistry* **2012**, *18*, 6928–6934, doi:10.1002/CHEM.201200062.

123. Cho, I.; Kim, S.H.; Kim, J.H.; Park, S.; Park, S.Y. Highly Efficient and Stable Deep-Blue Emitting Anthracene-Derived Molecular Glass for Versatile Types of Non-Doped OLED Applications. *J Mater Chem* **2011**, *22*, 123–129, doi:10.1039/C1JM14482K.

124. Liu, C.; Gu, Y.; Fu, Q.; Sun, N.; Zhong, C.; Ma, D.; Qin, J.; Yang, C. Nondoped Deep-Blue Organic Light-Emitting Diodes with Color Stability and Very Low Efficiency Roll-off: Solution-Processable Small-Molecule Fluorophores by Phosphine Oxide Linkage. *Chemistry - A European Journal* **2012**, *18*, 13828–13835, doi:10.1002/CHEM.201201512.

125. Meunmart, D.; Prachumrak, N.; Keawin, T.; Jungsuttiwong, S.; Sudyoadsuk, T.; Promark, V. Bis(4-Diphenylaminophenyl)Carbazole End-Capped Fluorene as Solution-Processed Deep-Blue Light-Emitting and Hole-Transporting Materials for Electroluminescent Devices. *Tetrahedron Lett* **2012**, *28*, 3615–3618, doi:10.1016/J.TETLET.2012.05.030.

126. Jou, J.H.; Chen, Y.L.; Tseng, J.R.; Wu, R.Z.; Shyue, J.J.; Justin Thomas, K.R.; Kapoor, N.; Chen, C.T.; Lin, Y.P.; Wang, P.H.; et al. The Use of a Polarity Matching and High-Energy Exciton Generating Host in Fabricating Efficient Purplish-Blue OLEDs from a Sky-Blue Emitter. *J Mater Chem* **2012**, *22*, 15500–15506, doi:10.1039/C2JM32216A.

127. Zou, Y.; Zou, J.; Ye, T.; Li, H.; Yang, C.; Wu, H.; Ma, D.; Qin, J.; Cao, Y. Unexpected Propeller-Like Hexakis(Fluoren-2-Yl)Benzene Cores for Six-Arm Star-Shaped Oligofluorenes: Highly Efficient Deep-Blue Fluorescent Emitters and Good Hole-Transporting Materials. *Adv Funct Mater* **2013**, *23*, 1781–1788, doi:10.1002/ADFM.201202286.

128. Liu, C.; Li, Y.; Li, Y.; Yang, C.; Wu, H.; Qin, J.; Cao, Y. Efficient Solution-Processed Deep-Blue Organic Light-Emitting Diodes Based on Multibranched Oligofluorenes with a Phosphine Oxide Center. *Chemistry of Materials* **2013**, *25*, 3320–3327, doi:10.1021/CM401640V/ASSET/IMAGES/MEDIUM/CM-2013-01640V_0008.GIF.

129. Giovanella, U.; Botta, C.; Galeotti, F.; Vercelli, B.; Battiato, S.; Pasini, M. Perfluorinated Polymer with Unexpectedly Efficient Deep Blue Electroluminescence for Full-Colour OLED Displays and Light Therapy Applications. *J Mater Chem C Mater* **2013**, *1*, 5322–5329, doi:10.1039/C3TC31139B.

130. Hellerich, E.S.; Intemann, J.J.; Cai, M.; Liu, R.; Ewan, M.D.; Tlach, B.C.; Jeffries-El, M.; Shinar, R.; Shinar, J. Fluorescent Polymer Guest:Small Molecule Host Solution-Processed OLEDs. *J Mater Chem C Mater* **2013**, *1*, 5191–5199, doi:10.1039/C3TC31019A.

131. Kumar, D.; Thomas, K.R.J.; Lin, C.C.; Jou, J.H. Pyrenoimidazole-Based Deep-Blue-Emitting Materials: Optical, Electrochemical, and Electroluminescent Characteristics. *Chem Asian J* **2013**, *8*, 2111–2124, doi:10.1002/ASIA.201300271.

132. McDowell, J.J.; Maier-Flaig, F.; Wolf, T.J.A.; Unterreiner, A.N.; Lemmer, U.; Ozin, G. Synthesis and Application of Photolithographically Patternable Deep Blue Emitting Poly(3,6-Dimethoxy-9,9-Dialkylsilafluorene)s. *ACS Appl Mater Interfaces* **2014**, *6*, 83, doi:10.1021/AM4025406.

133. Gao, Z.; Wang, Z.; Shan, T.; Liu, Y.; Shen, F.; Pan, Y.; Zhang, H.; He, X.; Lu, P.; Yang, B.; et al. High-Efficiency Deep Blue Fluorescent Emitters Based on Phenanthro[9,10-d]Imidazole Substituted Carbazole and Their Applications in Organic Light Emitting Diodes. *Org Electron* **2014**, *15*, 2667–2676, doi:10.1016/J.ORGEL.2014.07.019.

134. Zhang, S.; Yao, L.; Peng, Q.; Li, W.; Pan, Y.; Xiao, R.; Gao, Y.; Gu, C.; Wang, Z.; Lu, P.; et al. Achieving a Significantly Increased Efficiency in Nondoped Pure Blue Fluorescent OLED: A Quasi-Equivalent Hybridized Excited State. *Adv Funct Mater* **2015**, *25*, 1755–1762, doi:10.1002/ADFM.201404260.

135. Chen, S.F.; Tian, Y.; Peng, J.; Zhang, H.; Feng, X.J.; Zhang, H.; Xu, X.; Li, L.; Gao, J. Synthesis and Characterization of Arylamino End-Capped Silafluorenes for Blue to Deep-Blue Organic Light-Emitting Diodes (OLEDs). *J Mater Chem C Mater* **2015**, *3*, 6822–6830, doi:10.1039/C5TC00382B.

136. Zhang, Z.; Jiang, W.; Ban, X.; Yang, M.; Ye, S.; Huang, B.; Sun, Y. Solution-Processed Efficient Deep-Blue Fluorescent Organic Light-Emitting Diodes Based on Novel 9,10-Diphenyl-Anthracene Derivatives. *RSC Adv* **2015**, *5*, 29708–29717, doi:10.1039/C5RA00627A.

137. Chen, H.; Liang, W.; Chen, Y.; Tian, G.; Dong, Q.; Huang, J.; Su, J. Efficient Blue Fluorescent Organic Light-Emitting Diodes Based on Novel 9,10-Diphenyl-Anthracene Derivatives. *RSC Adv* **2015**, *5*, 70211–70219, doi:10.1039/C5RA11715A.

138. Reddy, S.S.; Cho, W.; Sree, V.G.; Jin, S.H. Multi-Functional Highly Efficient Bipolar 9,9-Dimethyl-9,10-Dihydroacridine/Imidazole-Based Materials for Solution-Processed Organic Light-Emitting Diode Applications. *Dyes and Pigments* **2016**, *134*, 315–324, doi:10.1016/J.DYEPIG.2016.07.034.

139. Sahoo, S.; Dubey, D.K.; Singh, M.; Joseph, V.; Thomas, K.R.J.; Jou, J.H. Highly Efficient Deep-Blue Organic Light Emitting Diode with a Carbazole Based Fluorescent Emitter. *Jpn J Appl Phys* **2018**, *57*, 04FL08, doi:10.7567/JJAP.57.04FL08/XML.

140. Jou, J.H.; Li, J.L.; Sahoo, S.; Dubey, D.K.; Kumar Yadav, R.A.; Joseph, V.; Thomas, K.R.J.; Wang, C.W.; Jayakumar, J.; Cheng, C.H. Enabling a 6.5% External Quantum Efficiency Deep-Blue Organic Light-Emitting Diode with a Solution-Processable Carbazole-Based Emitter. *Journal of Physical Chemistry C* **2018**, *122*, 24295–24303, doi:10.1021/ACS.JPCC.8B07641/ASSET/IMAGES/LARGE/JP-2018-076412_0002.JPEG.

141. Li, W.; Zhang, X.; Zhang, Y.; Xu, K.; Xu, J.; Wang, H.; Li, H.; Guo, J.; Mo, J.; Yang, P. Achieving Ultra-High Efficiency by Tuning Hole Transport and Carrier Balance in Fluorescent Blue Organic Light-Emitting Diode with Extremely Simple Structure. *Synth Met* **2018**, *245*, 111–115, doi:10.1016/J.SYNTHMET.2018.08.016.

142. Wang, Y.; Huang, H.; Wang, Y.; Zhu, Q.; Qin, J. Simple Oxadiazole Derivatives as Deep Blue Fluorescent Emitter and Bipolar Host for Organic Light-Emitting Diodes. *OptMa* **2018**, *84*, 278–283, doi:10.1016/J.OPTMAT.2018.07.015.

143. Li, W.; Zhang, Y.; Zheng, Q.; Xu, K.; Zhang, X.; Liu, L.; Wei, B.; Wang, L.; Xu, J.; Zhang, X. Solution-Processed WOx Hole Injection Layer for Efficient Fluorescent Blue Organic Light-Emitting Diode. *Current Applied Physics* **2018**, *18*, 583–589, doi:10.1016/J.CAP.2018.02.011.

144. Wu, Q.; Braveenth, R.; Zhang, H.Q.; Bae, I.J.; Kim, M.; Chai, K.Y. Oxadiazole-Based Highly Efficient Bipolar Fluorescent Emitters for Organic Light-Emitting Diodes. *Molecules* **2018**, *23*, doi:10.3390/MOLECULES23040843.

145. Therdkatanyuphong, P.; Kaiyasuan, C.; Chasing, P.; Kaewpuang, T.; Chawanpunyawat, T.; Wongkaew, P.; Sudyoadsuk, T.; Promarak, V. Highly Efficient All Solution-Processed Non-Doped Deep-Blue Electroluminescent Devices from Oligocarbazole-End-Capped Spirobifluorenes. *Mater Chem Front* **2020**, *4*, 2943–2953, doi:10.1039/D0QM00531B.

146. Yang, J.; Liu, X.; Liu, Z.; Wang, L.; Sun, J.; Guo, Z.; Xu, H.; Wang, H.; Zhao, B.; Xie, G. Protonation-Induced Dual Fluorescence of a Blue Fluorescent Material with Twisted A–π–D–π–A Configuration. *J Mater Chem C Mater* **2020**, *8*, 2442–2450, doi:10.1039/C9TC06425G.

147. Hu, L.; Liu, S.; Xie, G.; Yang, W.; Zhang, B. Bis(Benzothiophene-S,S-Dioxide) Fused Small Molecules Realize Solution-Processible, High-Performance and Non-Doped Blue Organic Light-Emitting Diodes. *J Mater Chem C Mater* **2020**, *8*, 1002–1009, doi:10.1039/C9TC05019A.

148. Adachi, C.; Kwong, R.C.; Djurovich, P.; Adamovich, V.; Baldo, M.A.; Thompson, M.E.; Forrest, S.R. Endothermic Energy Transfer: A Mechanism for Generating Very Efficient High-Energy Phosphorescent Emission in Organic Materials. *Appl Phys Lett* **2001**, *79*, 2082, doi:10.1063/1.1400076.

149. Holmes, R.J.; D'Andrade, B.W.; Forrest, S.R.; Ren, X.; Li, J.; Thompson, M.E. Efficient, Deep-Blue Organic Electrophosphorescence by Guest Charge Trapping. *Appl Phys Lett* **2003**, *83*, 3818, doi:10.1063/1.1624639.

150. Tokito, S.; Iijima, T.; Suzuri, Y.; Kita, H.; Tsuzuki, T.; Sato, F. Confinement of Triplet Energy on Phosphorescent Molecules for Highly-Efficient Organic Blue-Light-Emitting Devices. *Appl Phys Lett* **2003**, *83*, 569, doi:10.1063/1.1594834.

151. Lei, G.; Wang, L.; Qiu, Y. Improved Performance of Electrophosphorescent Organic Light-Emitting Diode by Graded Doped Emissive Layer. *Japanese Journal of Applied Physics, Part 2: Letters* **2004**, *43*, L1226, doi:10.1143/JJAP.43.L1226/XML.

152. Lei, G.T.; Wang, L.D.; Duan, L.; Wang, J.H.; Qiu, Y. Highly Efficient Blue Electrophosphorescent Devices with a Novel Host Material. *Synth Met* **2004**, *144*, 249–252, doi:10.1016/J.SYNTHMET.2004.03.010.

153. Holmes, R.J.; Forrest, S.R.; Sajoto, T.; Tamayo, A.; Djurovich, P.I.; Thompson, M.E.; Brooks, J.; Tung, Y.J.; D'Andrade, B.W.; Weaver, M.S.; et al. Saturated Deep Blue Organic Electrophosphorescence Using a Fluorine-Free Emitter. *Appl Phys Lett* **2005**, *87*, 243507, doi:10.1063/1.2143128.

154. Weaver, M.S.; Tung, Y.-J.; D'Andrade, B.; Esler, J.; Brown, J.J.; Lin, C.; Mackenzie, P.B.; Walters, R.W.; Tsai, J.-Y.; Brown, C.S.; et al. 11.1: Invited Paper: Advances in Blue Phosphorescent Organic Light-Emitting Devices. *SID Symposium Digest of Technical Papers* **2006**, *37*, 127, doi:10.1889/1.2433213.

155. Xiaohui Yang, B.; Wang, Z.; Madakuni, S.; Li, J.; Jabbour, G.E.; Jabbour, G.E.; Yang, X.; Li, J.; Wang, Z.; Madakuni, S. Efficient Blue- and White-Emitting Electrophosphorescent Devices Based on Platinum(II) [1,3-Difluoro-4,6-Di(2-Pyridinyl)Benzene] Chloride. *Advanced Materials* **2008**, *20*, 2405–2409, doi:10.1002/ADMA.200702940.

156. Chang, C.F.; Cheng, Y.M.; Chi, Y.; Chiu, Y.C.; Lin, C.C.; Lee, G.H.; Chou, P.T.; Chen, C.C.; Chang, C.H.; Wu, C.C. Highly Efficient Blue-Emitting Iridium(III) Carbene Complexes and Phosphorescent OLEDs. *Angewandte Chemie International Edition* **2008**, *47*, 4542–4545, doi:10.1002/ANIE.200800748.

157. Eom, S.H.; Zheng, Y.; Chopra, N.; Lee, J.; So, F.; Xue, J. Low Voltage and Very High Efficiency Deep-Blue Phosphorescent Organic Light-Emitting Devices. *Appl Phys Lett* **2008**, *93*, 133309, doi:10.1063/1.2996274.

158. Newman, R.H.; Zhang, J.; Nakano, S.; Wu, L.; Oka, H.; Tateishi Karimata, H.; Kirihata, T.; Sato, Y.; Fujii, S.; Sakai, H.; et al. Efficient Blue Phosphorescent Host through Nonbonded Conformational Locking Interactions. *New Journal of Chemistry* **2008**, *32*, 1368–1372, doi:10.1039/B714231E.

159. Zheng, Y.; Eom, S.H.; Chopra, N.; Lee, J.; So, F.; Xue, J. Efficient Deep-Blue Phosphorescent Organic Light-Emitting Device with Improved Electron and Exciton Confinement. *Appl Phys Lett* **2008**, *92*, 223301, doi:10.1063/1.2937403.

160. Chopra, N.; Lee, J.; Zheng, Y.; Eom, S.H.; Xue, J.; So, F. Effect of the Charge Balance on High-Efficiency Blue-Phosphorescent Organic Light-Emitting Diodes. *ACS Appl Mater Interfaces* **2009**, *1*, 1169–1172, doi:10.1021/AM900228B.

161. Jeon, S.O.; Yook, K.S.; Joo, C.W.; Lee, J.Y. Phenylcarbazole-Based Phosphine Oxide Host Materials For High Efficiency In Deep Blue Phosphorescent Organic Light-Emitting Diodes. *Adv Funct Mater* **2009**, *19*, 3644–3649, doi:10.1002/ADFM.200901274.

162. Yook, K.S.; Jeon, S.O.; Joo, C.W.; Lee, J.Y. High Efficiency Deep Blue Phosphorescent Organic Light-Emitting Diodes. *Org Electron* **2009**, *10*, 170–173, doi:10.1016/J.ORGEL.2008.10.018.

163. Bhansali, U.S.; Polikarpov, E.; Swensen, J.S.; Chen, W.H.; Jia, H.; Gaspar, D.J.; Gnade, B.E.; Padmaperuma, A.B.; Omary, M.A. High-Efficiency Turquoise-Blue Electrophosphorescence from a Pt(II)-Pyridyltriazolate Complex in a Phosphine Oxide Host. *Appl Phys Lett* **2009**, *95*, 233304, doi:10.1063/1.3268434.

164. Hu, D.; Lu, P.; Wang, C.; Liu, H.; Wang, H.; Wang, Z.; Fei, T.; Gu, X.; Ma, Y. Silane Coupling Di-Carbazoles with High Triplet Energy as Host Materials for Highly Efficient Blue Phosphorescent Devices. *J Mater Chem* **2009**, *19*, 6143–6148, doi:10.1039/B906782E.

165. Wee, K.R.; Han, W.S.; Son, H.J.; Kwon, S.; Kang, S.O. Efficiency and Colour Optimization of Carbazole Based Deep Blue Phosphorescent Organic Light Emitting Devices. *J Phys D Appl Phys* **2009**, *42*, doi:10.1088/0022-3727/42/23/235107.

166. Han, W.S.; Son, H.J.; Wee, K.R.; Min, K.T.; Kwon, S.; Suh, I.H.; Choi, S.H.; Jung, D.H.; Ook, S. Silicon-Based Blue Phosphorescence Host Materials: Structure and Photophysical Property Relationship on Methyl/Phenylsilanes Adorned with 4-(N-Carbazolyl)Phenyl Groups and Optimization of Their Electroluminescence by Peripheral 4-(n-Carbazolyl)Phenyl Numbers. *Journal of Physical Chemistry C* **2009**, *113*, 19686–19693, doi:10.1021/JP907104J/SUPPL_FILE/JP907104J_SI_002.CIF.

167. Hsu, F.M.; Chien, C.H.; Shu, C.F.; Lai, C.H.; Hsieh, C.C.; Wang, K.W.; Chou, P.T. A Bipolar Host Material Containing Triphenylamine and Diphenylphosphoryl-Substituted Fluorene Units for Highly Efficient Blue Electrophosphorescence. *Adv Funct Mater* **2009**, *19*, 2834–2843, doi:10.1002/ADFM.200900703.

168. Eom, S.H.; Zheng, Y.; Wrzesniewski, E.; Lee, J.; Chopra, N.; So, F.; Xue, J. Effect of Electron Injection and Transport Materials on Efficiency of Deep-Blue Phosphorescent Organic Light-Emitting Devices. *Org Electron* **2009**, *4*, 686–691, doi:10.1016/J.ORGEL.2009.03.002.

169. Hsu, F.M.; Chien, C.H.; Shih, P.I.; Shu, C.F. Phosphine-Oxide-Containing Bipolar Host Material for Blue Electrophosphorescent Devices. *Chemistry of Materials* **2009**, *21*, 1017–1022, doi:10.1021/CM802199D/SUPPL_FILE/CM802199D_SI_001.PDF.

170. Jiang, Z.; Xu, X.; Zhang, Z.; Yang, C.; Liu, Z.; Tao, Y.; Qin, J.; Ma, D. Diarylmethylene-Bridged 4,4′-(Bis(9-Carbazolyl))Biphenyl: Morphological Stable Host Material for Highly Efficient Electrophosphorescence. *J Mater Chem* **2009**, *19*, 7661–7665, doi:10.1039/B910247G.

171. Lee, J.; Lee, J.I.; Lee, J.Y.; Chu, H.Y. Improved Performance of Blue Phosphorescent Organic Light-Emitting Diodes with a Mixed Host System. *Appl Phys Lett* **2009**, *95*, 253304, doi:10.1063/1.3276075.

172. Sasabe, H.; Takamatsu, J.I.; Motoyama, T.; Watanabe, S.; Wagenblast, G.; Langer, N.; Molt, O.; Fuchs, E.; Lennartz, C.; Kido, J. High-Efficiency Blue and White Organic Light-Emitting Devices Incorporating a Blue Iridium Carbene Complex. *Advanced Materials* **2010**, *22*, 5003–5007, doi:10.1002/ADMA.201002254.

173. Lee, J.; Lee, J.I.; Lee, J.W.; Chu, H.Y. Effects of Charge Balance on Device Performances in Deep Blue Phosphorescent Organic Light-Emitting Diodes. *Org Electron* **2010**, *11*, 1159–1164, doi:10.1016/J.ORGEL.2010.04.014.

174. Gong, S.; Zhao, Y.; Wang, M.; Yang, C.; Zhong, C.; Qin, J.; Ma, D. Versatile Benzimidazole/Triphenylamine Hybrids: Efficient Nondoped Deep-Blue Electroluminescence and Good Host Materials for Phosphorescent Emitters. *Chem Asian J* **2010**, *5*, 2093–2099, doi:10.1002/ASIA.201000206.

175. Kwon, S.; Wee, K.R.; Kim, A.L.; Kang, S.O. Bis(4-(4,5-Diphenyl-4H-1,2,4-Triazol-3-Yl)Phenyl)-Dimethylsilane as Electron-Transport Material for Deep Blue Phosphorescent OLEDs. *Journal of Physical Chemistry Letters* **2010**, *1*, 295–299, doi:10.1021/JZ900238H/SUPPL_FILE/JZ900238H_SI_001.PDF.

176. Schrögel, P.; Hoping, M.; Kowalsky, W.; Hunze, A.; Wagenblast, G.; Lennartz, C.; Strohriegl, P. Phosphazene-Based Host Materials for the Use in Blue Phosphorescent Organic Light-Emitting Diodes. *Chemistry of Materials* **2011**, *23*, 4947–4953, doi:10.1021/CM201940F/ASSET/IMAGES/MEDIUM/CM-2011-01940F_0004.GIF.

177. Fukagawa, H.; Watanabe, K.; Tsuzuki, T.; Tokito, S. Highly Efficient, Deep-Blue Phosphorescent Organic Light Emitting Diodes with a Double-Emitting Layer Structure. *Appl Phys Lett* **6335**, *93*, doi:10.1063/1.2996572.

178. Schrögel, P.; Langer, N.; Schildknecht, C.; Wagenblast, G.; Lennartz, C.; Strohriegl, P. Meta-Linked CBP-Derivatives as Host Materials for a Blue Iridium Carbene Complex. *Org Electron* **2011**, *12*, 2047–2055, doi:10.1016/J.ORGEL.2011.08.012.

179. Hsieh, C.H.; Wu, F.I.; Fan, C.H.; Huang, M.J.; Lu, K.Y.; Chou, P.Y.; Yang, Y.H.O.; Wu, S.H.; Chen, I.C.; Chou, S.H.; et al. Design and Synthesis of Iridium Bis(Carbene) Complexes for Efficient Blue Electrophosphorescence. *Chemistry – A European Journal* **2011**, *17*, 9180–9187, doi:10.1002/CHEM.201100317.

180. Lu, K.Y.; Chou, H.H.; Hsieh, C.H.; Yang, Y.H.O.; Tsai, H.R.; Tsai, H.Y.; Hsu, L.C.; Chen, C.Y.; Chen, I.C.; Cheng, C.H. Wide-Range Color Tuning of Iridium Biscarbene Complexes from Blue to Red by Different N∡N Ligands: An Alternative Route for Adjusting the Emission Colors. *Advanced Materials* **2011**, *23*, 4933–4937, doi:10.1002/ADMA.201102886.

181. Park, M.S.; Choi, D.H.; Lee, B.S.; Lee, J.Y. Fused Indole Derivatives as High Triplet Energy Hole Transport Materials for Deep Blue Phosphorescent Organic Light-Emitting Diodes. *J Mater Chem* **2012**, *22*, 3099–3104, doi:10.1039/C2JM15204E.

182. Ok Jeon, S.; Eok Jang, S.; Suk Son, H.; Yeob Lee, J.; Jeon, S.O.; Jang, S.E.; Son, H.S.; Lee, J.Y. External Quantum Efficiency Above 20% in Deep Blue Phosphorescent Organic Light-Emitting Diodes. *Advanced Materials* **2011**, *23*, 1436–1441, doi:10.1002/ADMA.201004372.

183. Jeong, S.H.; Seo, C.W.; Lee, J.Y.; Cho, N.S.; Kim, J.K.; Yang, J.H. Comparison of Bipolar Hosts and Mixed-Hosts as Host Structures for Deep-Blue Phosphorescent Organic Light Emitting Diodes. *Chem Asian J* **2011**, *6*, 2895–2898, doi:10.1002/ASIA.201100596.

184. Fukagawa, H.; Irisa, S.; Hanashima, H.; Shimizu, T.; Tokito, S.; Yokoyama, N.; Fujikake, H. Simply Structured, Deep-Blue Phosphorescent Organic Light-Emitting Diode with Bipolar Host Material. *Org Electron* **2011**, *12*, 1638–1643, doi:10.1016/J.ORGEL.2011.06.012.

185. Park, J.S.; Seo, J.H.; Koo, J.R.; Seo, B.M.; Lee, K.H.; Yoon, S.S.; Kim, Y.K. High Efficiency Phosphorescent Blue Organic Light-Emitting Diodes Using Double Emitting Layer. *J Nanosci Nanotechnol* **2011**, *11*, 4488–4491, doi:10.1166/JNN.2011.3704.

186. Gong, S.; Chen, Y.; Zhang, X.; Cai, P.; Zhong, C.; Ma, D.; Qin, J.; Yang, C. High-Performance Blue and Green Electrophosphorescence Achieved by Using Carbazole-Containing Bipolar Tetraarylsilanes as Host Materials. *J Mater Chem* **2011**, *21*, 11197–11204, doi:10.1039/C1JM11208B.

187. Chen, Y.M.; Hung, W.Y.; You, H.W.; Chaskar, A.; Ting, H.C.; Chen, H.F.; Wong, K.T.; Liu, Y.H. Carbazole–Benzimidazole Hybrid Bipolar Host Materials for Highly Efficient Green and Blue Phosphorescent OLEDs. *J Mater Chem* **2011**, *21*, 14971–14978, doi:10.1039/C1JM12598B.

188. Jeong, S.H.; Lee, J.Y. Dibenzothiophene Derivatives as Host Materials for High Efficiency in Deep Blue Phosphorescent Organic Light Emitting Diodes. *J Mater Chem* **2011**, *21*, 14604–14609, doi:10.1039/C1JM12421H.

189. Park, M.S.; Lee, J.Y. Indolo Acridine-Based Hole-Transport Materials for Phosphorescent OLEDs with over 20% External Quantum Efficiency in Deep Blue and Green. *Chemistry of Materials* **2011**, *23*, 4338–4343, doi:10.1021/CM201634W/ASSET/IMAGES/LARGE/CM-2011-01634W_0010.JPEG.

190. Yu, E.S.; Jung, S.H.; Chae, M.Y.; Jeon, W.S.; Kulshreshtha, C.; Park, J.S.; Kwon, J.H. Phosphorescent Blue Organic Light-Emitting Diodes with New Bipolar Host Materials. *J Nanosci Nanotechnol* **2012**, *12*, 1361–1364, doi:10.1166/JNN.2012.4586.

191. Piao, X.; Yin, Y.; Liu, J.; Li, Y.; Xu, K.; Wang, Y.; Xie, W. High-Efficiency Blue and White Organic Light-Emitting Devices by Combining Fluorescent and Phosphorescent Blue Emitters. *Org Electron* **2012**, *13*, 2412–2416, doi:10.1016/J.ORGEL.2012.07.006.

192. Hwang, J.; Kyw Choi, H.; Moon, J.; Yong Kim, T.; Shin, J.W.; Woong Joo, C.; Han, J.H.; Cho, D.H.; Woo Huh, J.; Choi, S.Y.; et al. Multilayered Graphene Anode for Blue Phosphorescent Organic Light Emitting Diodes. *Appl Phys Lett* **2012**, *100*, 133304, doi:10.1063/1.3697639.

193. Yang, C.H.; Mauro, M.; Polo, F.; Watanabe, S.; Muenster, I.; Fröhlich, R.; De Cola, L. Deep-Blue-Emitting Heteroleptic Iridium(III) Complexes Suited for Highly Efficient Phosphorescent OLEDs. *Chemistry of Materials* **2012**, *24*, 3684–3695, doi:10.1021/CM3010453/SUPPL_FILE/CM3010453_SI_001.PDF.

194. Moon, B.J.; Yook, K.S.; Lee, J.Y.; Shin, S.; Hwang, S.H. Synthesis, Photophysical and Electro-Optical Properties of Bis-Carbazolyl Methane Based Host Material for Pure-Blue Phosphorescent OLED. *J Lumin* **2012**, *132*, 2557–2560, doi:10.1016/J.JLUMIN.2012.05.011.

195. Jeong, S.H.; Lee, J.Y. Dibenzofuran Derivative as High Triplet Energy Host Material for High Efficiency in Deep Blue Phosphorescent Organic Light-Emitting Diodes. *Org Electron* **2012**, *13*, 1141–1145, doi:10.1016/J.ORGEL.2012.03.024.

196. Seo, C.W.; Lee, J.Y. High External Quantum Efficiency in Deep Blue Phosphorescent Organic Light Emitting Diodes Using a Simple Device Structure. *Thin Solid Films* **2012**, *520*, 7022–7025, doi:10.1016/J.TSF.2012.07.015.

197. Kim, O.Y.; Lee, J.Y. High Efficiency Deep Blue Phosphorescent Organic Light-Emitting Diodes Using a Tetraphenylsilane Based Phosphine Oxide Host Material. *Journal of Industrial and Engineering Chemistry* **2012**, *18*, 1029–1032, doi:10.1016/J.JIEC.2011.11.148.

198. Jeon, S.O.; Lee, J.Y. Comparison of Symmetric and Asymmetric Bipolar Type High Triplet Energy Host Materials for Deep Blue Phosphorescent Organic Light-Emitting Diodes. *J Mater Chem* **2012**, *22*, 7239–7244, doi:10.1039/C2JM30742A.

199. Wee, K.R.; Cho, Y.J.; Jeong, S.; Kwon, S.; Lee, J.D.; Suh, I.H.; Kang, S.O. Carborane-Based Optoelectronically Active Organic Molecules: Wide Band Gap Host Materials for Blue Phosphorescence. *J Am Chem Soc* **2012**, *134*, 17982–17990, doi:10.1021/JA3066623/SUPPL_FILE/JA3066623_SI_002.CIF.

200. Bin, J.K.; Yang, J.; Hong, J.I. New Sulfur-Containing Host Materials for Blue Phosphorescent Organic Light-Emitting Diodes. *J Mater Chem* **2012**, *22*, 21720–21726, doi:10.1039/C2JM34165D.

201. Yang, X.; Huang, H.; Pan, B.; Aldred, M.P.; Zhuang, S.; Wang, L.; Chen, J.; Ma, D. Modified 4,4′,4″-Tri(N -Carbazolyl)Triphenylamine as a Versatile Bipolar Host for Highly Efficient Blue, Orange, and White Organic Light-Emitting Diodes. *Journal of Physical Chemistry C* **2012**, *116*, 15041–15047, doi:10.1021/JP3034566.

202. Chen, C.-T.; Yeh, S.-J.; Wu, M.-F.; Chi, Y.; Song, Y.-H.; Ho, M.-H.; Hsu, S.-F.; Chen, C.H. 61.3: Blue Dopants and New Host Materials for Phosphorescent Organic Light-Emitting Diodes. *SID Symposium Digest of Technical Papers* **2005**, *36*, 1756, doi:10.1889/1.2036356.

203. Lee, M.-T.; Lin, J.-S.; Chu, M.-T.; Tseng, M.-R. P-215: Improved Carrier Transport into Wide-Bandgap Host for Low-Voltage High-Efficiency Blue PHOLEDs. *SID Symposium Digest of Technical Papers* **2008**, *39*, 2014–2017, doi:10.1889/1.3069597.

204. Huang, H.; Wang, Y.; Pan, B.; Yang, X.; Wang, L.; Chen, J.; Ma, D.; Yang, C. Simple Bipolar Hosts with High Glass Transition Temperatures Based on 1,8-Disubstituted Carbazole for Efficient Blue and Green Electrophosphorescent Devices with "Ideal" Turn-on Voltage., doi:10.1002/chem.201202329.

205. Kang, Y.; Chang, Y.L.; Lu, J.S.; Ko, S.B.; Rao, Y.; Varlan, M.; Lu, Z.H.; Wang, S. Highly Efficient Blue Phosphorescent and Electroluminescent Ir(III) Compounds. *J Mater Chem C Mater* **2012**, *1*, 441–450, doi:10.1039/C2TC00270A.

206. Lee, S.; Kim, S.O.; Shin, H.; Yun, H.J.; Yang, K.; Kwon, S.K.; Kim, J.J.; Kim, Y.H. Deep-Blue Phosphorescence from Perfluoro Carbonyl-Substituted Iridium Complexes. *J Am Chem Soc* **2013**, *135*, 14321–14328, doi:10.1021/JA4065188/SUPPL_FILE/JA4065188_SI_001.PDF.

207. Kessler, F.; Watanabe, Y.; Sasabe, H.; Katagiri, H.; Nazeeruddin, M.K.; Grätzel, M.; Kido, J. High-Performance Pure Blue Phosphorescent OLED Using a Novel Bis-Heteroleptic Iridium(III) Complex with Fluorinated Bipyridyl Ligands. *J Mater Chem C Mater* **2013**, *1*, 1070–1075, doi:10.1039/C2TC00836J.

208. Park, J.; Oh, H.; Oh, S.; Kim, J.; Park, H.J.; Kim, O.Y.; Lee, J.Y.; Kang, Y. Deep Blue Phosphorescent Organic Light-Emitting Diodes with Excellent External Quantum Efficiency. *Org Electron* **2013**, *14*, 3228–3233, doi:10.1016/J.ORGEL.2013.09.017.

209. Hang, X.-C.; Fleetham, T.; Turner, E.; Brooks, J.; Li, J.; Hang, X.; Fleetham, T.; Turner, E.; Li, J.; Brooks, J. Highly Efficient Blue-Emitting Cyclometalated Platinum(II) Complexes by Judicious Molecular Design. *Angewandte Chemie International Edition* **2013**, *52*, 6753–6756, doi:10.1002/ANIE.201302541.

210. Kim, M.; Lee, J.Y. Synthesis of 3-Substituted Carbazole Derivative as a Host Material for Deep Blue Phosphorescent Organic Light-Emitting Diodes. *Synth Met* **2013**, *Complete*, 18–22, doi:10.1016/J.SYNTHMET.2013.08.003.

211. Park, M.S.; Lee, J.Y. An Indole Derivative as a High Triplet Energy Hole Transport Material for Blue Phosphorescent Organic Light-Emitting Diodes. *Thin Solid Films* **2013**, *548*, 603–607, doi:10.1016/J.TSF.2013.09.084.

212. Lee, C.W.; Im, Y.; Seo, J.A.; Lee, J.Y. Carboline Derivatives with an Ortho-Linked Terphenyl Core for High Quantum Efficiency in Blue Phosphorescent Organic Light-Emitting Diodes. *Chemical Communications* **2013**, *49*, 9860–9862, doi:10.1039/C3CC44944K.

213. Kim, S.J.; Kim, Y.J.; Son, Y.H.; Hur, J.A.; Um, H.A.; Shin, J.; Lee, T.W.; Cho, M.J.; Kim, J.K.; Joo, S.; et al. High-Efficiency Blue Phosphorescent Organic Light-Emitting Diodes Using a Carbazole and Carboline-Based Host Material. *Chemical Communications* **2013**, *49*, 6788–6790, doi:10.1039/C3CC42569J.

214. Chen, H.; Jiang, Z.Q.; Gao, C.H.; Xu, M.F.; Dong, S.C.; Cui, L.S.; Ji, S.J.; Liao, L.S. Silicon-Based Material with Spiro-Annulated Fluorene/Triphenylamine as Host and Exciton-Blocking Layer for Blue Electrophosphorescent Devices. *Chemistry – A European Journal* **2013**, *19*, 11791–11797, doi:10.1002/CHEM.201301106.

215. Cui, L.S.; Dong, S.C.; Liu, Y.; Li, Q.; Jiang, Z.Q.; Liao, L.S. A Simple Systematic Design of Phenylcarbazole Derivatives for Host Materials to High-Efficiency Phosphorescent Organic Light-Emitting Diodes. *J Mater Chem C Mater* **2013**, *1*, 3967–3975, doi:10.1039/C3TC30410H.

216. Son, Y.H.; Kim, Y.J.; Park, M.J.; Oh, H.Y.; Park, J.S.; Yang, J.H.; Suh, M.C.; Kwon, J.H. Small Single–Triplet Energy Gap Bipolar Host Materials for Phosphorescent Blue and White Organic Light Emitting Diodes. *J Mater Chem C Mater* **2013**, *1*, 5008–5014, doi:10.1039/C3TC30671B.

217. Fan, C.; Chen, Y.; Liu, Z.; Jiang, Z.; Zhong, C.; Ma, D.; Qin, J.; Yang, C. Tetraphenylsilane Derivatives Spiro-Annulated by Triphenylamine/Carbazole with Enhanced HOMO Energy Levels and Glass Transition Temperatures without Lowering Triplet Energy: Host Materials for Efficient Blue Phosphorescent OLEDs. 中科院 **2013**, *1*, 463–469, doi:10.1039/C2TC00082B.

218. Lee, C.W.; Lee, J.Y. High Quantum Efficiency in Blue Phosphorescent Organic Light Emitting Diodes Using Ortho-Substituted High Triplet Energy Host Materials. *Org Electron* **2013**, *14*, 1602–1607, doi:10.1016/J.ORGEL.2013.03.033.

219. Huang, H.; Yang, X.; Wang, Y.; Pan, B.; Wang, L.; Chen, J.; Ma, D.; Yang, C. Optimizing the Conjugation between N,N0-Dicarbazolyl-3, 5-Benzene and Triphenylphosphine Oxide as Bipolar Hybrids for Highly Efficient Blue and Single Emissive Layer White Phosphorescent OLEDs. *Org Electron* **2013**, *14*, 2573–2581, doi:10.1016/J.ORGEL.2013.06.011.

220. Fan, C.; Zhu, L.; Jiang, B.; Zhong, C.; Ma, D.; Qin, J.; Yang, C. Efficient Blue and Bluish-Green Iridium Phosphors: Fine-Tuning Emissions of FIrpic by Halogen Substitution on Pyridine-Containing Ligands. *Org Electron* **2013**, *14*, 3163–3171, doi:10.1016/J.ORGEL.2013.09.026.

221. Peter Strohriegl, al; Wagner, D.; Schrögel, P.; Hoffmann, S.T.; Köhler, A.; Heinemeyer, U.; Münster, I.; Strohriegl, P. Novel Host Materials for Blue Phosphorescent OLEDs. *https://doi.org/10.1117/12.2023305* **2013**, *8829*, 9–20, doi:10.1117/12.2023305.

222. Ding, L.; Dong, S.C.; Jiang, Z.Q.; Chen, H.; Liao, L.S. Orthogonal Molecular Structure for Better Host Material in Blue Phosphorescence and Larger OLED White Lighting Panel. *Adv Funct Mater* **2015**, *25*, 645–650, doi:10.1002/ADFM.201403402.

223. Sun, M.L.; Yue, S.Z.; Lin, J.R.; Ou, C.J.; Qian, Y.; Zhang, Y.; Li, Y.; Wei, Q.; Zhao, Y.; Xie, L.H.; et al. Dimeric SFX Host Materials for Red, Green and Blue Phosphorescent Organic Light-Emitting Devices. *Synth Met* **2014**, *195*, 321–327, doi:10.1016/J.SYNTHMET.2014.06.022.

224. Liu, T.; Sun, H.; Fan, C.; Ma, D.; Zhong, C.; Yang, C. High Efficiency Blue PhOLEDs Using Spiro-Annulated Triphenylamine/Fluorene Hybrids as Host Materials with High Triplet Energy, High HOMO Level and High Tg. *Org Electron* **2014**, *15*, 3568–3576, doi:10.1016/J.ORGEL.2014.10.009.

225. Yuan, X.D.; Liang, J.; He, Y.C.; Li, Q.; Zhong, C.; Jiang, Z.Q.; Liao, L.S. A Rational Design of Carbazole-Based Host Materials with Suitable Spacer Group towards Highly-Efficient Blue Phosphorescence. *J Mater Chem C Mater* **2014**, *2*, 6387–6394, doi:10.1039/C4TC00867G.

226. Fan, C.; Zhu, L.; Liu, T.; Jiang, B.; Ma, D.; Qin, J.; Yang, C. Using an Organic Molecule with Low Triplet Energy as a Host in a Highly Efficient Blue Electrophosphorescent Device. *Angew Chem Int Ed Engl* **2014**, *53*, 2147–2151, doi:10.1002/ANIE.201308046.

227. Gong, S.; Chang, Y.L.; Wu, K.; White, R.; Lu, Z.H.; Song, D.; Yang, C. High-Power-Efficiency Blue Electrophosphorescence Enabled by the Synergistic Combination of Phosphine-Oxide-Based Host and Electron-Transporting Materials. *Chemistry of Materials* **2014**, *26*, 1463–1470, doi:10.1021/CM4037555/SUPPL_FILE/CM4037555_SI_001.PDF.

228. Liao, K.Y.; Hsu, C.W.; Chi, Y.; Hsu, M.K.; Wu, S.W.; Chang, C.H.; Liu, S.H.; Lee, G.H.; Chou, P.T.; Hu, Y.; et al. Pt(II) Metal Complexes Tailored with a Newly Designed Spiro-Arranged Tetradentate Ligand; Harnessing of Charge-Transfer Phosphorescence and Fabrication of Sky Blue and White OLEDs. *Inorg Chem* **2015**, *54*, 4029–4038, doi:10.1021/ACS.INORGCHEM.5B00281/SUPPL_FILE/IC5B00281_SI_002.CIF.

229. Seino, Y.; Sasabe, H.; Kimura, M.; Inomata, S.; Nakao, K.; Kido, J. Novel Blue Exciplex Comprising Acridine and Sulfone Derivatives as a Host Material for High-Efficiency Blue Phosphorescent OLEDs. *https://doi.org/10.1246/cl.151094* **2015**, *45*, 283–285, doi:10.1246/CL.151094.

230. Jou, J.H.; Kumar, S.; Fang, P.H.; Venkateswararao, A.; Thomas, K.R.J.; Shyue, J.J.; Wang, Y.C.; Li, T.H.; Yu, H.H. Highly Efficient Ultra-Deep Blue Organic Light-Emitting Diodes with a Wet- and Dry-Process Feasible Cyanofluorene Acetylene Based Emitter. *J Mater Chem C Mater* **2015**, *3*, 2182–2194, doi:10.1039/C4TC02547D.

231. Wu, F.P.; Xie, Y.M.; Cui, L.S.; Liu, X.Y.; Li, Q.; Jiang, Z.Q.; Liao, L.S. A Host Material Consisting of Phosphinic Amide for Efficient Sky-Blue Phosphorescent Organic Light-Emitting Diodes. *Synth Met* **2015**, *205*, 11–17, doi:10.1016/J.SYNTHMET.2015.03.023.

232. Hung, Y.H.; Chiu, T.L. Using Carbazole-Triazole Derives Host in Blue Phosphorescent OLEDs. *Proceedings of AM-FPD 2015 - 22nd International Workshop on Active-Matrix Flatpanel Displays and Devices: TFT Technologies and FPD Materials* **2015**, 85–88, doi:10.1109/AM-FPD.2015.7173203.

233. Im, Y.; Lee, J.Y. Effect of Nitrogen Position of Carboline on the Device Performances of Blue Phosphorescent Organic Light-Emitting Diodes. *Synth Met* **2015**, *209*, 24–28, doi:10.1016/J.SYNTHMET.2015.06.014.

234. Yang, X.; Du, X.; Tao, S.; Huang, Y.; Ding, X.; Xue, R. Efficient Hole-Transporter for Phosphorescent Organic Light Emitting Diodes with a Simple Molecular Structure. *Org Electron* **2015**, *26*, 481–486, doi:10.1016/J.ORGEL.2015.08.011.

235. Wang, L.; Pan, B.; Zhu, L.; Wang, B.; Wang, Y.; Liu, Y.; Jin, J.; Chen, J.; Ma, D. Construction of Thermally Stable 3,6-Disubstituted Spiro-Fluorene Derivatives as Host Materials for Blue Phosphorescent Organic Light-Emitting Diodes. *Dyes and Pigments* **2015**, *114*, 222–230, doi:10.1016/J.DYEPIG.2014.11.011.

236. Li, Y.; Wang, Z.; Li, X.; Xie, G.; Chen, D.; Wang, Y.F.; Lo, C.C.; Lien, A.; Peng, J.; Cao, Y.; et al. Highly Efficient Spiro[Fluorene-9,9-Thioxanthene] Core Derived Blue Emitters and Fluorescent/Phosphorescent Hybrid White Organic Light-Emitting Diodes. *Chemistry of Materials* **2015**, *27*, 1100–1109, doi:10.1021/CM504441V/SUPPL_FILE/CM504441V_SI_001.PDF.

237. Song, J.Y.; Lee, S.B.; Lee, S.J.; Kim, Y.K.; Yoon, S.S. Organic Light-Emitting Diodes Based on 9-(2-Naphthyl)Anthracene Derivatives with a Triphenylsilane Unit as the Deep-Blue Emitting Layer. *Thin Solid Films* **2015**, *577*, 42–48, doi:10.1016/J.TSF.2015.01.050.

238. Wang, C.; Yuan, Y.; Li, S.Y.; Sun, Z.B.; Jiang, Z.Q.; Zhao, C.H. A Highly Twisted Triarylborane-Based Biphenyl as an Efficient Host for Blue and Green Phosphorescent OLEDs. *J Mater Chem C Mater* **2016**, *4*, 7607–7613, doi:10.1039/C6TC02451C.

239. Cho, H.; Lee, J.; Lee, J.I.; Cho, N.S.; Park, J.H.; Lee, J.Y.; Kang, Y. Phenylimidazole-Based Homoleptic Iridium(III) Compounds for Blue Phosphorescent Organic Light-Emitting Diodes with High Efficiency and Long Lifetime. *Org Electron* **2016**, *34*, 91–96, doi:10.1016/J.ORGEL.2016.04.026.

240. Kuei, C.-Y.; Tsai, W.-L.; Tong, B.; Jiao, M.; Lee, W.-K.; Chi, Y.; Wu, C.-C.; Liu, S.-H.; Lee, G.-H.; Chou, P.-T.; et al. Bis-Tridentate Ir(III) Complexes with Nearly Unitary RGB Phosphorescence and Organic Light-Emitting Diodes with External Quantum Efficiency Exceeding 31%. *Advanced Materials* **2016**, *28*, 2795–2800, doi:10.1002/ADMA.201505790.

241. Lv, X.; Wang, B.; Pan, B.; Huang, Z.; Xiang, S.; Tan, J.; Yi, W.; Huang, H.; Wang, L. Influence of the D/A Ratio of 1,3,5-Triphenylbenzene Based Starburst Host Materials on Blue Electrophosphorescent Devices: A Comparative Study. *RSC Adv* **2016**, *6*, 46775–46784, doi:10.1039/C6RA07952K.

242. Wu, Z.G.; Jing, Y.M.; Lu, G.Z.; Zhou, J.; Zheng, Y.X.; Zhou, L.; Wang, Y.; Pan, Y. Novel Design of Iridium Phosphors with Pyridinylphosphinate Ligands for High-Efficiency Blue Organic Light-Emitting Diodes. *Scientific Reports 2016 6:1* **2016**, *6*, 1–9, doi:10.1038/srep38478.

243. Lim, H.; Shin, H.; Kim, K.H.; Yoo, S.J.; Huh, J.S.; Kim, J.J. An Exciplex Host for Deep-Blue Phosphorescent Organic Light-Emitting Diodes. *ACS Appl Mater Interfaces* **2017**, *9*, 37883–37887, doi:10.1021/ACSAMI.7B10914/ASSET/IMAGES/LARGE/AM-2017-109142_0003.JPEG.

244. Wang, F.; Liu, D.; Li, J.; Ma, M. Modulation of N-Type Units in Bipolar Host Materials toward High-Performance Phosphorescent OLEDs. *ACS Appl Mater Interfaces* **2017**, *9*, 37888–37897, doi:10.1021/ACSAMI.7B11667.

245. Hu, J.; Zhao, C.; Zhang, T.; Zhang, X.; Cao, X.; Wu, Q.; Chen, Y.; Zhang, D.; Tao, Y.; Huang, W.; et al. Isomeric N-Linked Benzoimidazole Containing New Electron Acceptors for Exciplex Forming Hosts in Highly Efficient Blue Phosphorescent OLEDs. *Adv Opt Mater* **2017**, *5*, 1700036, doi:10.1002/ADOM.201700036.

246. Sarma, M.; Tsai, W.L.; Lee, W.K.; Chi, Y.; Wu, C.C.; Liu, S.H.; Chou, P.T.; Wong, K.T. Anomalously Long-Lasting Blue PhOLED Featuring Phenyl-Pyrimidine Cyclometalated Iridium Emitter. *Chem* **2017**, *3*, 461–476, doi:10.1016/J.CHEMPR.2017.08.001.

247. Cho, Y.J.; Kim, S.Y.; Kim, J.H.; Lee, J.; Cho, D.W.; Yi, S.; Son, H.J.; Han, W.S.; Kang, S.O. Probing Photophysical Properties of Isomeric N-Heterocyclic Carbene Ir(III) Complexes and Their Applications to Deep-Blue Phosphorescent Organic Light-Emitting Diodes. *J Mater Chem C Mater* **2017**, *5*, 1651–1659, doi:10.1039/C6TC04757B.

248. Choi, H.J.; Hyun, M.H.; Park, H.J.; Yoon, U.C. Synthesis of Efficient Blue Phosphorescent Heteroleptic Ir(III) Complexes Containing 4-Alkoxy- or 4-Alkylaminopicolinates as Ancillary Ligands. *J Lumin* **2017**, *188*, 323–330, doi:10.1016/J.JLUMIN.2017.04.043.

249. Kim, G.W.; Yang, D.R.; Kim, Y.C.; Yang, H.I.; Fan, J.G.; Lee, C.H.; Chai, K.Y.; Kwon, J.H. Di(Biphenyl)Silane and Carbazole Based Bipolar Host Materials for Highly Efficient Blue Phosphorescent OLEDs. *Dyes and Pigments* **2017**, *136*, 8–16, doi:10.1016/J.DYEPIG.2016.08.024.

250. Yong Byeon, S.; Hyun Han, S.; Yeob Lee, J.; Byeon, S.Y.; Han, S.H.; Lee, J.Y. Molecular Design of Blue Phosphorescent Host Materials for Phenylimidazole-Type Blue Triplet Emitters to Extend Operational Lifetime. *Adv Opt Mater* **2018**, *6*, 1701263, doi:10.1002/ADOM.201701263.

251. Liu, X.Y.; Tian, Q.S.; Zhao, D.; Fan, J.; Liao, L.S. Novel O-D-π-A Arylamine/Arylphosphine Oxide Hybrid Hosts for Efficient Phosphorescent Organic Light-Emitting Diodes. *Org Electron* **2018**, *56*, 186–191, doi:10.1016/J.ORGEL.2018.02.021.

252. Wang, F.; Liu, D.; Li, J. High-Triplet-Energy Host Materials Containing Saturated Carbon Atom for Blue and Green Phosphorescent OLEDs. *J Photochem Photobiol A Chem* **2018**, *355*, 152–157, doi:10.1016/J.JPHOTOCHEM.2017.09.059.

253. Chen, Z.; Wang, L.; Ho, C.L.; Chen, S.; Suramitr, S.; Plucksacholatarn, A.; Zhu, N.; Hannongbua, S.; Wong, W.Y. Smart Design on the Cyclometalated Ligands of Iridium(III) Complexes for Facile Tuning of Phosphorescence Color Spanning from Deep-Blue to Near-Infrared. *Adv Opt Mater* **2018**, *6*, 1800824, doi:10.1002/ADOM.201800824.

254. Pal, A.K.; Krotkus, S.; Fontani, M.; R Mackenzie, C.F.; Cordes, D.B.; Z Slawin, A.M.; W Samuel, I.D.; Zysman-Colman, E.; Pal, A.K.; Fontani, M.; et al. High-Efficiency Deep-Blue-Emitting Organic Light-Emitting Diodes Based on Iridium(III) Carbene Complexes. *Advanced Materials* **2018**, *30*, 1804231, doi:10.1002/ADMA.201804231.

255. Li, X.; Zhang, J.; Zhao, Z.; Wang, L.; Yang, H.; Chang, Q.; Jiang, N.; Liu, Z.; Bian, Z.; Liu, W.; et al. Deep Blue Phosphorescent Organic Light-Emitting Diodes with CIEy Value of 0.11 and External Quantum Efficiency up to 22.5%. *Advanced Materials* **2018**, *30*, 1705005, doi:10.1002/ADMA.201705005.

256. Xia, J.C.; Liang, X.; Yan, Z.P.; Wu, Z.G.; Zhao, Y.; Zheng, Y.X.; Zhang, W.W. Iridium(III) Phosphors with Bis(Diphenylphorothioyl)Amide Ligand for Efficient Green and Sky-Blue OLEDs with EQE of Nearly 28%. *J Mater Chem C Mater* **2018**, *6*, 9010–9016, doi:10.1039/C8TC02265H.

257. Liu, D.; Wang, F.; Yao, R. Molecular Evolution of Host Materials by Regular Tuning of n/p Ratio for High-Performance Phosphorescent Organic Light-Emitting Diodes. *J Mater Chem C Mater* **2018**, *6*, 7839–7846, doi:10.1039/C8TC02432D.

258. Chen, W.C.; Yuan, Y.; Zhu, Z.L.; Ni, S.F.; Jiang, Z.Q.; Liao, L.S.; Wong, F.L.; Lee, C.S. A Novel Spiro-Annulated Benzimidazole Host for Highly Efficient Blue Phosphorescent Organic Light-Emitting Devices. *Chemical Communications* **2018**, *54*, 4541–4544, doi:10.1039/C8CC00903A.

259. Su, N.; Shen, C.Z.; Zheng, Y.X. Efficient Electroluminescence of Sky-Blue Iridium(III) Complexes for Organic Light-Emitting Diodes. *Dyes and Pigments* **2018**, *159*, 100–106, doi:10.1016/J.DYEPIG.2018.06.011.

260. Yang, R.; Li, D.; Bai, Y.; Zhang, L.; Liu, Z.; Hao, J.; Wei, Q.; Liao, L.; Peng, R.; Ge, Z. Novel Tetraarylsilane-Based Hosts for Blue Phosphorescent Organic Light-Emitting Diodes. *Org Electron* **2018**, *55*, 117–125, doi:10.1016/J.ORGEL.2018.01.026.

261. Hu, W.K.; Li, S.H.; Ma, X.F.; Zhou, S.X.; Zhang, Q. feng; Xu, J.Y.; Shi, P.; Tong, B.H.; Fung, M.K.; Fu, L. Blue-to-Green Electrophosphorescence from Iridium(III) Complexes with Cyclometalated Pyrimidine Ligands. *Dyes and Pigments* **2018**, *150*, 284–292, doi:10.1016/J.DYEPIG.2017.12.020.

262. Hindenberg, P.; Zimmermann, J.; Hernandez-Sosa, G.; Romero-Nieto, C. Lighting with Organophosphorus Materials: Solution-Processed Blue/Cyan Light-Emitting Devices Based on Phosphaphenalenes. *Dalton Transactions* **2019**, *48*, 7503–7508, doi:10.1039/C9DT00380K.

263. Hsu, L.Y.; Liang, Q.; Wang, Z.; Kuo, H.H.; Tai, W.S.; Su, S.J.; Zhou, X.; Yuan, Y.; Chi, Y. Bis-Tridentate Ir III Phosphors Bearing Two Fused Five-Six-Membered Metallacycles: A Strategy to Improved Photostability of Blue Emitters. *Chemistry* **2019**, *25*, 15375–15386, doi:10.1002/CHEM.201903707.

264. Lee, S.E.; Oh, J.H.; Baek, H.J.; Kim, S.; Do, Y.R.; Kim, Y.K. Realization of High-Color-Quality White-by-Blue Organic Light-Emitting Diodes with Yellow and Red Phosphor Films. *J Lumin* **2019**, *207*, 195–200, doi:10.1016/J.JLUMIN.2018.10.109.

265. Ying, S.; Pang, P.; Zhang, S.; Sun, Q.; Dai, Y.; Qiao, X.; Yang, D.; Chen, J.; Ma, D. Superior Efficiency and Low-Efficiency Roll-Off White Organic Light-Emitting Diodes Based on Multiple Exciplexes as Hosts Matched to Phosphor Emitters. *ACS Appl Mater Interfaces* **2019**, *11*, 31078–31086, doi:10.1021/ACSAMI.9B09429/ASSET/IMAGES/LARGE/AM9B09429_0006.JPEG.

266. Li, G.; Zhao, X.; Fleetham, T.; Chen, Q.; Zhan, F.; Zheng, J.; Yang, Y.F.; Lou, W.; Yang, Y.; Fang, K.; et al. Tetradentate Platinum(II) Complexes for Highly Efficient Phosphorescent Emitters and Sky Blue OLEDs. *Chemistry of Materials* **2020**, *32*, 537–548, doi:10.1021/ACS.CHEMMATER.9B04263/SUPPL_FILE/CM9B04263_SI_007.PDF.

267. Zhu, X.D.; Zhang, Y.L.; Yuan, Y.; Zheng, Q.; Yu, Y.J.; Li, Y.; Jiang, Z.Q.; Liao, L.S. Incorporating a Tercarbazole Donor in a Spiro-Type Host Material for Efficient RGB Phosphorescent Organic Light-Emitting Diodes. *J Mater Chem C Mater* **2019**, *7*, 6714–6720, doi:10.1039/C9TC01426H.

268. Jang, H.J.; Lee, J.Y. Triplet Energy Boosting Ternary Exciplex Host for Improved Efficiency in Deep Blue Phosphorescent Organic Light Emitting Diode. *Org Electron* **2019**, *75*, 105441, doi:10.1016/J.ORGEL.2019.105441.

269. Klimes, K.; Zhu, Z.Q.; Li, J. Efficient Blue Phosphorescent OLEDs with Improved Stability and Color Purity through Judicious Triplet Exciton Management. *Adv Funct Mater* **2019**, *29*, doi:10.1002/adfm.201903068.

270. Wu, Y.; Tan, X.; Lv, A.; Yu, F.; Ma, H.; Shen, K.; Sun, Z.; Chen, F.; Chen, Z.K.; Hang, X.C. Triplet Excited-State Engineering of Phosphorescent Pt(II) Complexes. *J Phys Chem Lett* **2019**, *10*, 5105–5110, doi:10.1021/ACS.JPCLETT.9B01685.

271. Yang, C.Y.; Jang, H.J.; Lee, K.H.; Byeon, S.Y.; Lee, J.Y.; Jeong, H. 12-1: Analysis of Key Factors Affecting the Lifetime of Blue Phosphorescent OLED Using CN Modified Blue Host Materials. *SID Symposium Digest of Technical Papers* **2019**, *50*, 141–144, doi:10.1002/SDTP.12875.

272. Zheng, X.-H.; Zhao, J.-W.; Huang, T.-T.; Chen, X.; Cao, C.; Yang, G.-X.; Lin, Z.-H.; Tong, Q.-X.; Tao, S.-L.; Liu, D. Cover Feature: Versatile Host Materials for Highly-Efficient Green, Red Phosphorescent and White Organic Light-Emitting Diodes (ChemElectroChem 23/2019). *ChemElectroChem* **2019**, *6*, 5766–5766, doi:10.1002/CELC.201901834.

273. Yu, Z.; Zhang, J.; Liu, S.; Zhang, L.; Zhao, Y.; Zhao, H.; Xie, W. High-Efficiency Blue Phosphorescent Organic Light-Emitting Devices with Low Efficiency Roll-Off at Ultrahigh Luminance by the Reduction of Triplet-Polaron Quenching. *ACS Appl Mater Interfaces* **2019**, doi:10.1021/ACSAMI.8B19280/ASSET/IMAGES/LARGE/AM-2018-192803_0002.JPEG.

274. Han, S.H.; Lee, K.H.; Lee, J.Y. Spatial Separation of Two Blue Triplet Emitters for Improved Lifetime in Blue Phosphorescent Organic Light-Emitting Diodes by Confining Excitons at the Interface between Two Emitting Layers. *Org Electron* **2019**, *69*, 227–231, doi:10.1016/J.ORGEL.2019.03.042.

275. Ma, H.; Shen, K.; Wu, Y.; Xia, F.; Yu, F.; Sun, Z.; Qian, C.; Peng, Q.; Zhang, H.H.; You, C.; et al. High-Color-Purity and Efficient Solution-Processable Blue Phosphorescent Light-Emitting Diodes with Pt(II) Complexes Featuring 3ππ* Transitions. *Mater Chem Front* **2019**, *3*, 2448–2454, doi:10.1039/C9QM00347A.

276. Liu, X.Y.; Zhang, Y.J.; Fei, X.; Ran, Q.; Fung, M.K.; Fan, J. Diazaspirocycles: Novel Platforms for Efficient Phosphorescent Organic Light-Emitting Diodes. *J Mater Chem C Mater* **2019**, *7*, 1370–1378, doi:10.1039/C8TC05462B.

277. Park, H.-Y.; Maheshwaran, A.; Moon, C.-K.; Lee, H.; Sudhaker Reddy, S.; Gopalan Sree, V.; Yoon, J.; Won Kim, J.; Hyuk Kwon, J.; Kim, J.-J.; et al. External Quantum Efficiency Exceeding 24% with CIEy Value of 0.08 Using a Novel Carbene-Based Iridium Complex in Deep-Blue Phosphorescent Organic Light-Emitting Diodes. *Advanced Materials* **2020**, *32*, 2002120, doi:10.1002/ADMA.202002120.

278. Zhu, Z.L.; Chen, W.C.; Ni, S.F.; Yan, J.; Wang, S.F.; Fu, L.W.; Tsai, H.Y.; Chi, Y.; Lee, C.S. Constructing Deep-Blue Bis-Tridentate Ir(III) Phosphors with Fluorene-Based Dianionic Chelates. *J Mater Chem C Mater* **2021**, *9*, 1318–1325, doi:10.1039/D0TC05218C.

279. Bullock, J.D.; Xu, Z.; Valandro, S.; Younus, M.; Xue, J.; Schanze, K.S. Trans- N-(Heterocyclic Carbene) Platinum(II) Acetylide Chromophores as Phosphors for OLED Applications. *ACS Appl Electron Mater* **2020**, *2*, 1026–1034, doi:10.1021/ACSAELM.0C00064/ASSET/IMAGES/MEDIUM/EL0C00064_0004.GIF.

280. Han, P.; Xu, Z.; Lin, C.; Ma, D.; Qin, A.; Tang, B.Z. Tetraphenylbenzene-Based AIEgens: Horizontally Oriented Emitters for Highly Efficient Non-Doped Deep Blue OLEDs and Hosts for High-Performance Hybrid WOLEDs. *J Mater Chem C Mater* **2020**, *8*, 7012–7018, doi:10.1039/D0TC00920B.

281. Jung, M.; Lee, K.H.; Lee, J.Y.; Kim, T. A Bipolar Host Based High Triplet Energy Electroplex for an over 10 000 h Lifetime in Pure Blue Phosphorescent Organic Light-Emitting Diodes. *Mater Horiz* **2020**, *7*, 559–565, doi:10.1039/C9MH01268K.

282. Qiu, X.; Ying, S.; Yao, J.; Zhou, J.; Wang, C.; Wang, B.; Li, Y.; Xu, Y.; Jiang, Q.; Zhao, R.; et al. Universal Host Materials Based on Carbazole-Formate Derivatives for Blue, Green and Red Phosphorescent Organic Light-Emitting Diodes. *Dyes and Pigments* **2020**, *174*, doi:10.1016/J.DYEPIG.2019.108045.

283. Ma, H.; Liu, D.; Li, J.; Mei, Y.; Li, D.; Ding, Y.; Wei, W. Sky-Blue Iridium Complexes with Pyrimidine Ligands for Highly Efficient Phosphorescent Organic Light-Emitting Diodes. *New Journal of Chemistry* **2020**, *44*, 8743–8750, doi:10.1039/D0NJ01262A.

284. Cheng, W.; Wang, L.D.; Zhou, Y.Y.; Bian, Z.Q.; Tong, B.H.; Liu, Z.W.; Wang, S. Blue Iridium(III) Complexes with High Internal Quantum Efficiency Based on 4-(Pyridin-3-Yl)Pyrimidine Derivative and Their Electroluminescent Properties. *Dyes and Pigments* **2020**, *177*, 108257, doi:10.1016/J.DYEPIG.2020.108257.

285. Chen, Z.; Suramitr, S.; Zhu, N.; Ho, C.L.; Hannongbua, S.; Chen, S.; Wong, W.Y. Tetrafluorinated Phenylpyridine Based Heteroleptic Iridium(III) Complexes for Efficient Sky Blue Phosphorescent Organic Light-Emitting Diodes. *J Mater Chem C Mater* **2020**, *8*, 2551–2557, doi:10.1039/C9TC05779J.

286. Yao, J.; Xiao, S.; Zhang, S.; Sun, Q.; Dai, Y.; Qiao, X.; Yang, D.; Chen, J.; Ma, D. High Efficiency, Low Efficiency Roll-off and Long Lifetime Fluorescent White Organic Light-Emitting Diodes Based on Strategic Management of Triplet Excitons via Triplet–Triplet Annihilation up-Conversion and Phosphor Sensitization. *J Mater Chem C Mater* **2020**, *8*, 8077–8084, doi:10.1039/D0TC01622E.

287. Lucas, F.; Quinton, C.; Fall, S.; Heiser, T.; Tondelier, D.; Geffroy, B.; Leclerc, N.; Rault-Berthelot, J.; Poriel, C. Universal Host Materials for Red, Green and Blue High-Efficiency Single-Layer Phosphorescent Organic Light-Emitting Diodes. *J Mater Chem C Mater* **2020**, *8*, 16354–16367, doi:10.1039/D0TC04650G.

288. Kang, J.; Zaen, R.; Park, K.M.; Lee, K.H.; Lee, J.Y.; Kang, Y. Cyclometalated Platinum(II) β-Diketonate Complexes as Single Dopants for High-Efficiency White OLEDs: The Relationship between Intermolecular Interactions in the Solid State and Electroluminescent Efficiency. *Cryst Growth Des* **2020**, *20*, 6129–6138, doi:10.1021/ACS.CGD.0C00838/ASSET/IMAGES/LARGE/CG0C00838_0009.JPEG.

289. Chen, Y.; Yang, D.; Qiao, X.; Dai, Y.; Sun, Q.; Ma, D. Novel Strategy to Improve the Efficiency Roll-off at High Luminance and Operational Lifetime of Hybrid White OLEDs via Employing an Assistant Layer with Triplet–Triplet Annihilation up-Conversion Characteristics. *J Mater Chem C Mater* **2020**, *8*, 6577–6586, doi:10.1039/D0TC00867B.

290. Kumar Konidena, R.; Jae Chung, W.; Yeob Lee, J.; Konidena, R.K.; Chung, W.J.; Lee, J.Y. Molecular Engineering of Cyano-Substituted Carbazole-Based Host Materials for Simultaneous Achievement of High Efficiency and Long Lifetime in Blue Phosphorescent Organic Light-Emitting Diodes. *Adv Electron Mater* **2020**, *6*, 2000132, doi:10.1002/AELM.202000132.

291. Wang, L.; Zhao, Z.; Zhan, G.; Fang, H.; Yang, H.; Huang, T.; Zhang, Y.; Jiang, N.; Duan, L.; Liu, Z.; et al. Deep-Blue Organic Light-Emitting Diodes Based on a Doublet d–f Transition Cerium(III) Complex with 100% Exciton Utilization Efficiency. *Light: Science & Applications 2020 9:1* **2020**, *9*, 1–9, doi:10.1038/s41377-020-00395-4.

292. Zhang, Y.L.; Ran, Q.; Wang, Q.; Tian, Q.S.; Kong, F.C.; Fan, J.; Liao, L.S. High-Performance Sky-Blue Phosphorescent Organic Light-Emitting Diodes Employing Wide-Bandgap Bipolar Host Materials with Thermally Activated Delayed Fluorescence Characteristics. *Org Electron* **2020**, *81*, 105660, doi:10.1016/J.ORGEL.2020.105660.

293. Zhao, Z.; Wang, L.; Zhan, G.; Liu, Z.; Bian, Z.; Huang, C. Efficient Rare Earth Cerium(III) Complex with Nanosecond D−f Emission for Blue Organic Light-Emitting Diodes. *Natl Sci Rev* **2021**, *8*, 2021, doi:10.1093/NSR/NWAA193.

294. Tai, W.S.; Hsu, L.Y.; Hung, W.Y.; Chen, Y.Y.; Ko, C.L.; Zhou, X.; Yuan, Y.; Jen, A.K.Y.; Chi, Y. Methoxy-Substituted Bis-Tridentate Iridium(III) Phosphors and Fabrication of Blue Organic Light Emitting Diodes. *J Mater Chem C Mater* **2020**, *8*, 13590–13602, doi:10.1039/D0TC03453C.

295. You, A.; Be, M.A.Y.; In, I. Electrophosphorescent Device with Conjugated Polymers as the Host. **2006**, *051116*, 24–27, doi:10.1063/1.2167788.

296. Erk, P.; Bold, M.; Egen, M.; Fuchs, E.; Geßner, T.; Kahle, K.; Lennartz, C.; Molt, O.; Nord, S.; Reichelt, H.; et al. 11 . 2 : Efficient Deep Blue Triplet Emitters for OLEDs Design of Carbene Triplet Emitters Results and Discussion Properties of Iridium Carbene Emitters. **2006**, 131–134.

297. Song, Y.; Chiu, Y.; Chi, Y.; Cheng, Y.; Lai, C.; Chou, P.; Wong, K.; Tsai, M.; Wu, C. Phosphorescent Iridium ACHTUNG RE (III) Complexes with Nonconjugated Cyclometalated. **2008**, 5423–5434, doi:10.1002/chem.200800050.

298. Diode, L.; Jou, J.; Wang, W.; Hsu, M.; Liu, C.; Chen, C. Small Nano-Dot Incorporated High-Efficiency Phosphorescent Blue Organic Small Nano-Dot Incorporated High-Efficiency Phosphorescent Blue Organic Light-Emitting Diode. **2008**, doi:10.2529/PIERS070827062209.

299. Wee, K.R.; Kim, A.L.; Jeong, S.Y.; Kwon, S.; Kang, S.O. The Relationship between the Device Performance and Hole Mobility of Host Materials in Mixed Host System for Deep Blue Phosphorescent Organic Light Emitting Devices. *Org Electron* **2011**, *12*, 1973–1979, doi:10.1016/J.ORGEL.2011.08.021.

300. Ahmed, E.; Earmme, T.; Jenekhe, S.A. New Solution-Processable Electron Transport Materials for Highly Effi Cient Blue Phosphorescent OLEDs. **2011**, 3889–3899, doi:10.1002/adfm.201100848.

301. Gong, S.; Fu, Q.; Wang, Q.; Yang, C.; Zhong, C.; Qin, J.; Ma, D. Highly Efficient Deep-Blue Electrophosphorescence Enabled by Solution-Processed Bipolar Tetraarylsilane Host with Both a High Triplet Energy and a High-Lying HOMO Level. *Advanced Materials* **2011**, *23*, 4956–4959, doi:10.1002/ADMA.201102758.

302. Liu, S.-W.; Yuan, C.-H.; Yeh, S.-J.; Wu, M.-F.; Chen, C.-T.; Lee, C.-C. Efficiency Enhancement of Solution-Processed Single-Layer Blue-Phosphorescence Organic Light-Emitting Devices Having Co-Host Materials of Polymer (PVK) and Small-Molecule (SimCP2). *J Soc Inf Disp* **2011**, *19*, 346, doi:10.1889/JSID19.4.346.

303. Park, J.H.; Koh, T.W.; Do, Y.; Lee, M.H.; Yoo, S. Soluble Polynorbornenes with Pendant Carbazole Derivatives as Host Materials for Highly Efficient Blue Phosphorescent Organic Light-Emitting Diodes. *J Polym Sci A Polym Chem* **2012**, *50*, 2356–2365, doi:10.1002/POLA.26005.

304. Gong, S.; Fu, Q.; Zeng, W.; Zhong, C.; Yang, C.; Qin, J. Solution-Processed Double-Silicon-Bridged Oxadiazole/Arylamine Hosts for High-E Ffi Ciency Blue Electrophosphorescence. **2012**.

305. Jiang, W.; Duan, L.; Qiao, J.; Dong, G.; Zhang, D.; Wang, L.; Qiu, Y. Dyes and Pigments Novel Carbazole / Pyridine-Based Host Material for Solution-Processed Blue Phosphorescent Organic Light-Emitting Devices. *Dyes and Pigments* **2012**, *92*, 891–896, doi:10.1016/j.dyepig.2011.08.009.

306. Jiang, W.; Tang, J.; Yang, W.; Ban, X.; Huang, B.; Dai, Y.; Sun, Y.; Duan, L.; Qiao, J.; Wang, L.; et al. Synthesis of Carbazole-Based Dendrimer: Host Material for Highly Efficient Solution-Processed Blue Organic Electrophosphorescent Diodes. *Tetrahedron* **2012**, *68*, 5800–5805, doi:10.1016/J.TET.2012.05.025.

307. Sciencedirect, S.; All, E.B. V; The, D.; Scientific, F.; Gemini, V.; Technology, A.; Electrospray, S.; Trap, I. Synthetic Metals. **2012**, *162*, 1828–1833, doi:10.1016/j.synthmet.2012.09.001.

308. Jiang, W.; Yang, W.; Ban, X.; Huang, B.; Dai, Y.; Sun, Y.; Duan, L.; Qiu, Y. Synthesis of New Bipolar Materials Based on Diphenylphosphine Oxide and Triphenylamine Units: Efficient Host for Deep-Blue Phosphorescent Organic Light-Emitting Diodes. *Tetrahedron* **2012**, *68*, 9672–9678, doi:10.1016/J.TET.2012.09.051.

309. Yook, K.S.; Lee, J.Y. Solution Processed Multilayer Deep Blue and White Phosphorescent Organic Light-Emitting Diodes Using an Alcohol Soluble Bipolar Host and Phosphorescent Dopant Materials. *J Mater Chem* **2012**, *22*, 14546–14550, doi:10.1039/C2JM31814H.

310. Fu, Q.; Chen, J.; Shi, C.; Ma, D. Solution-Processed Small Molecules as Mixed Host for Highly Efficient Blue and White Phosphorescent Organic Light-Emitting Diodes. *ACS Appl Mater Interfaces* **2012**, *4*, 6579–6586, doi:10.1021/AM301703A/ASSET/IMAGES/MEDIUM/AM-2012-01703A_0010.GIF.

311. Lee, N.J.; Lee, D.H.; Kim, D.W.; Lee, J.H.; Cho, S.H.; Jeon, W.S.; Kwon, J.H.; Suh, M.C. Highly Efficient Soluble Materials for Blue Phosphorescent Organic Light–Emitting Diode. *Dyes and Pigments* **2012**, *95*, 221–228, doi:10.1016/J.DYEPIG.2012.04.011.

312. Gong, S.; Zhong, C.; Fu, Q.; Ma, D.; Qin, J.; Yang, C. Extension of Molecular Structure toward Solution-Processable Hosts for Efficient Blue Phosphorescent Organic Light-Emitting Diodes. *Journal of Physical Chemistry C* **2013**, *117*, 549–555, doi:10.1021/JP309100E/ASSET/IMAGES/LARGE/JP-2012-09100E_0007.JPEG.

313. Qian, Y.; Cao, F.; Guo, W. High Thermal Stability 3, 6-Fluorene-Carbazole-Dendrimers as Host Materials for Efficient Solution-Processed Blue Phosphorescent Devices. *Tetrahedron* **2013**, *69*, 4169–4175, doi:10.1016/J.TET.2013.04.004.

314. Yun, S.J.; Seo, H.J.; Song, M.; Jin, S.H.; Kang, S.K.; Kim, Y.I. Sky-Blue Phosphorescent Iridium(III) Complexes with Two Substituted 2-Phenylpyridine Derivatives and One Picolinic Acid for Organic Light-Emitting Diodes. *J Organomet Chem* **2013**, *724*, 244–250, doi:10.1016/J.JORGANCHEM.2012.11.016.

315. Kozhevnikov, V.N.; Zheng, Y.; Clough, M.; Al-attar, H.A.; Gri, G.C.; Abdullah, K.; Raisys, S.; Jankus, V.; Bryce, M.R.; Monkman, A.P. Cyclometalated Ir(III) Complexes for High-E Ffi Ciency Solution- Processable Blue PhOLEDs. **2013**, 2–8.

316. Yeoh, K.H.; Yi, C.; Ng, B.; Chua, C.L.; Azrinatalik, N.; Woon, K.L. High Power Efficiency Phosphorescent Organic Light-Emitting Diode by Controlling Charge Transport at the Emissive Layer and Heterojunction. **2013**, *424*, 421–424, doi:10.1002/pssr.201307089.

317. Kim, C.; Park, H.; Won, H.; Jin, W.; Young, S. All-in-One-Type Organic Light-Emitting Diodes for Color Tuning Using Phosphor in Glasses with Pb-Free Silicate Powders. *Current Applied Physics* **2014**, *14*, 1677–1681, doi:10.1016/j.cap.2014.09.018.

318. Liu, L.; Liu, X.; Wu, K.; Ding, J.; Zhang, B.; Xie, Z.; Wang, L. Efficient Solution-Processed Blue Phosphorescent Organic Light-Emitting Diodes with Halogen-Free Solvent to Optimize the Emissive Layer Morphology. *Org Electron* **2014**, *15*, 1401–1406, doi:10.1016/J.ORGEL.2014.04.005.

319. Lee, Y.T.; Chang, Y.T.; Lee, M.T.; Chiang, P.H.; Chen, C.T.; Chen, C.T. Solution-Processed Bipolar Small Molecular Host Materials for Single-Layer Blue Phosphorescent Organic Light-Emitting Diodes. *J Mater Chem C Mater* **2013**, *2*, 382–391, doi:10.1039/C3TC31641F.

320. Tian, G.; Liang, W.; Chen, Y.; Xiang, N.; Dong, Q.; Huang, J.; Su, J.H. A Novel Spiro-Annulated Host Based on Carbazole with Good Thermal Stability and High Triplet Energy for Efficient Blue and Green Phosphorescent Organic Light-Emitting Diodes. *Dyes and Pigments* **2016**, *126*, 296–302, doi:10.1016/J.DYEPIG.2015.11.017.

321. Li, L.; Hu, T.; Yin, C.; Xie, L.; Yang, Y.; Wang, C.; Lin, J.; Yi, M.; Ye, S.; Huang, W. Polymer Chemistry. **2015**, 983–988, doi:10.1039/c4py01016g.

322. Dumur, F.; Beouch, L.; Peralta, S.; Wantz, G.; Goubard, F. Solution-Processed Blue Phosphorescent OLEDs with Carbazole-Based Polymeric Host Materials. **2015**, *25*, 21–30, doi:10.1016/j.orgel.2015.06.013.

323. Ban, X.; Jiang, W.; Sun, K.; Xie, X.; Peng, L.; Dong, H.; Sun, Y.; Huang, B.; Duan, L.; Qiu, Y. Bipolar Host with Multielectron Transport Benzimidazole Units for Low Operating Voltage and High Power E Ffi Ciency Solution- Processed Phosphorescent OLEDs. **2015**, doi:10.1021/acsami.5b00510.

324. Ban, X.; Sun, K.; Sun, Y.; Huang, B.; Ye, S.; Yang, M.; Jiang, W. High Power Efficiency Solution-Processed Blue Phosphorescent Organic Light-Emitting Diodes Using Exciplex-Type Host with a Turn-on Voltage Approaching the Theoretical Limit. *ACS Appl Mater Interfaces* **2015**, *7*, 25129–25138, doi:10.1021/ACSAMI.5B06424/SUPPL_FILE/AM5B06424_SI_001.PDF.

325. Sun, D.; Zhou, X.; Li, H.; Sun, X.; Ren, Z.; Yan, S. Multi-3,3 ′ -Bicarbazole-Substituted Arylsilane Host Materials with Balanced Charge Transport for Highly E Ffi Cient Solution-Processed Blue Phosphorescent Organic Light-Emitting Diodes. **2015**, doi:10.1021/acsami.5b04112.

326. Jiang, W.; Ye, M.; Ban, X.; Sun, Y. Phenylcarbazole/Diphenylphosphine Oxide-Based Alcohol Soluble Host Materials for Efficient Solution-Processed Multilayer Blue Electrophosphorescent OLEDs. *Dyes and Pigments* **2015**, *122*, 192–198, doi:10.1016/J.DYEPIG.2015.06.028.

327. Zhang, F.; Li, W.; Wei, D.; Dong, X.; Li, S.; Li, Z.; Zhang, F. Inorganica Chimica Acta Solution-Processed Organic Light-Emitting Diodes Based on a Blue-Emitting Cationic Iridium (III) Complex Using 2- (1H-Pyrazol-1-Yl) Pyridine as Ancillary Ligand. *Inorganica Chim Acta* **2016**, *453*, 115–121, doi:10.1016/j.ica.2016.07.052.

328. Byeon, S.Y.; Choi, J.M.; Lee, J.Y. Dyes and Pigments Pyridoindole Based Intramolecular Charge Transfer Type Host Material for Blue Phosphorescent Organic Light-Emitting Diodes. *Dyes and Pigments* **2016**, *134*, 285–290, doi:10.1016/j.dyepig.2016.07.021.

329. Ban, X.; Sun, K.; Sun, Y.; Huang, B.; Jiang, W. Enhanced Electron Affinity and Exciton Confinement in Exciplex-Type Host: Power Efficient Solution-Processed Blue Phosphorescent OLEDs with Low Turn-on Voltage. *ACS Appl Mater Interfaces* **2016**, *8*, 2010–2016, doi:10.1021/ACSAMI.5B10335/SUPPL_FILE/AM5B10335_SI_001.PDF.

330. Oh, C.S.; Lee, Y.; Noh, H.; Han, S. Molecular Design of Host Materials for High Power Efficiency in Blue Phosphorescent Organic Light-Emitting Diodes Doped with an Imidazole Ligand Based Triplet Emitter. **2016**, 3792–3797, doi:10.1039/c5tc02595h.

331. Okamura, N.; Nakamura, T.; Yagi, S.; Maeda, T.; Nakazumi, H.; Fujiwara, H.; Koseki, S. Novel Bis- and Tris-Cyclometalated Iridium(III) Complexes Bearing a Benzoyl Group on Each Fluorinated 2-Phenylpyridinate Ligand Aimed at Development of Blue Phosphorescent Materials for OLED. *RSC Adv* **2016**, *6*, 51435–51445, doi:10.1039/C6RA09385J.

332. Benjamin, H.; Zheng, Y.; Batsanov, A.S.; Fox, M.A.; Al-Attar, H.A.; Monkman, A.P.; Bryce, M.R. Sulfonyl-Substituted Heteroleptic Cyclometalated Iridium(III) Complexes as Blue Emitters for Solution-Processable Phosphorescent Organic Light-Emitting Diodes. *Inorg Chem* **2016**, *55*, 8612–8627, doi:10.1021/ACS.INORGCHEM.6B01179/SUPPL_FILE/IC6B01179_SI_002.ZIP.

333. Li, W.; Li, J.; Liu, D.; Jin, Q. Simple Bipolar Host Materials for High-E Ffi Ciency Blue, Green, and White Phosphorescence OLEDs. **2016**, doi:10.1021/acsami.6b05355.

334. Feng, Y.; Zhuang, X.; Zhu, D.; Liu, Y.; Bryce, M.R. Heteroleptic Phosphorescent Iridium (III). **2016**, 10246–10252, doi:10.1039/c6tc04119a.

335. Jhulki, S.; Seth, S.; Ghosh, A.; Chow, T.J.; Moorthy, J.N. Benzophenones as Generic Host Materials for Phosphorescent Organic Light-Emitting Diodes. **2016**, doi:10.1021/acsami.5b11232.

336. Park, S.R.; Kim, S.M.; Kang, J.H.; Lee, J.H.; Suh, M.C. Bipolar Host Materials with Carbazole and Dipyridylamine Groups Showing High Triplet Energy for Blue Phosphorescent Organic Light Emitting Diodes. *Dyes and Pigments* **2017**, *141*, 217–224, doi:10.1016/J.DYEPIG.2017.02.014.

337. Kim, M.; Lee, I.H.; Lee, J.; Hyun, S.; Park, K.; Kang, Y. Journal of Industrial and Engineering Chemistry Phenanthridine Based Ir (III) Complex for Blue Phosphorescent Organic Light-Emitting Diodes with Long-Term Operational Stability. *Journal of Industrial and Engineering Chemistry* **2018**, *58*, 386–390, doi:10.1016/j.jiec.2017.09.052.

338. Liu, Y.; Hao, Z.; Jiang, H.; Liu, Y.; Wang, Y.; Tan, H.; Xie, G. High-Efficiency Blue OLEDs Based on Dendritic Dinuclear Iridium (III) Complexes Grafted with Fluorene Core and Blue Fluorescence Chromospheres. *Tetrahedron* **2018**, *74*, 425–432, doi:10.1016/J.TET.2017.12.018.

339. Kwon, Y.; Han, S.H.; Yu, S.; Lee, J.Y.; Lee, K.M. Functionalized Phenylimidazole-Based Facial-Homoleptic Iridium(Iii) Complexes and Their Excellent Performance in Blue Phosphorescent Organic Light-Emitting Diodes. **2018**, 4565–4572, doi:10.1039/c8tc00568k.

340. Lee, C.; Zaen, R.; Park, K.; Lee, K.H.; Lee, J.Y.; Kang, Y. Blue Phosphorescent Platinum Complexes Based on Tetradentate Bipyridine Ligands and Their Application to Organic Light-Emitting Diodes (OLEDs). **2018**, doi:10.1021/acs.organomet.8b00659.

341. Bail, R.; Hong, J.Y.; Chin, B.D. Inkjet Printing of Blue Phosphorescent Light-Emitting Layer Based on Bis(3,5-Di(9H-Carbazol-9-Yl))Diphenylsilane. *RSC Adv* **2018**, *8*, 11191–11197, doi:10.1039/C8RA00582F.

342. Ye, H.; Wu, H.; Chen, L.; Ma, S.; Zhou, K.; Yan, G.; Shen, J.; Chen, D.; Su, S.J. Synthesis, Properties, Calculations and Applications of Small Molecular Host Materials Containing Oxadiazole Units with Different Nitrogen and Oxygen Atom Orientations for Solution-Processable Blue Phosphorescent OLEDs. *EML* **2018**, *14*, 89–100, doi:10.1007/S13391-018-0011-8.

343. Choi, H.J.; Kim, B.S.; Kim, O.; Chin, B.D.; Lee, C.W. The Correlation between Structure and Physical Properties of Soluble Host for Blue Phosphorescent OLED. **2019**, *74*, 1176–1183, doi:10.3938/jkps.74.1176.

344. Kim, J.H.; Kim, S.Y.; Jang, S.; Yi, S.; Cho, D.W.; Son, H.J.; Kang, S.O. Blue Phosphorescence with High Quantum Efficiency Engaging the Trifluoromethylsulfonyl Group to Iridium Phenylpyridine Complexes. *Inorg Chem* **2019**, *58*, 16112–16125, doi:10.1021/ACS.INORGCHEM.9B02672/SUPPL_FILE/IC9B02672_SI_001.PDF.

345. Manuscript, A. Ac Ce Pte d M Us. **2020**.

346. Manuscript, A. Materials Chemistry C. **2020**, doi:10.1039/D0TC05150K.

347. Sun, J.W.; Baek, J.Y.; Kim, K.H.; Moon, C.K.; Lee, J.H.; Kwon, S.K.; Kim, Y.H.; Kim, J.J. Thermally Activated Delayed Fluorescence from Azasiline Based Intramolecular Charge-Transfer Emitter (DTPDDA) and a Highly Efficient Blue Light Emitting Diode. *Chemistry of Materials* **2015**, *27*, 6675–6681, doi:10.1021/ACS.CHEMMATER.5B02515/ASSET/IMAGES/LARGE/CM-2015-02515X_0006.JPEG.

348. Hiraga, Y.; Nishide, J.I.; Nakanotani, H.; Aonuma, M.; Adachi, C. High-Efficiency Sky-Blue Organic Light-Emitting Diodes Utilizing Thermally-Activated Delayed Fluorescence. *IEICE Transactions on Electronics* **2015**, *E98C*, 971–976, doi:10.1587/TRANSELE.E98.C.971.

349. Mallesham, G.; Swetha, C.; Niveditha, S.; Mohanty, M.E.; Babu, N.J.; Kumar, A.; Bhanuprakash, K.; Rao, V.J. Phosphine Oxide Functionalized Pyrenes as Efficient Blue Light Emitting Multifunctional Materials for Organic Light Emitting Diodes. *J Mater Chem C Mater* **2015**, *3*, 1208–1224, doi:10.1039/C4TC01753F.

350. Rajamalli, P.; Senthilkumar, N.; Gandeepan, P.; Ren-Wu, C.C.; Lin, H.W.; Cheng, C.H. A Method for Reducing the Singlet-Triplet Energy Gaps of TADF Materials for Improving the Blue OLED Efficiency. *ACS Appl Mater Interfaces* **2016**, *8*, 27026–27034, doi:10.1021/ACSAMI.6B10678/ASSET/IMAGES/LARGE/AM-2016-106784_0007.JPEG.

351. Zhang, J.; Li, J.; Chen, W.; Zheng, D.; Yu, J.; Wang, H.; Xu, B. An Efficient Blue Thermally Activated Delayed Fluorescence Material Based on 4-Fluorocyanobenzene Derivative for Organic Light-Emitting Diodes. *Tetrahedron Lett* **2016**, *19*, 2044–2048, doi:10.1016/J.TETLET.2016.03.097.

352. Sun, J.W.; Kim, K.H.; Moon, C.K.; Lee, J.H.; Kim, J.J. Highly Efficient Sky-Blue Fluorescent Organic Light Emitting Diode Based on Mixed Cohost System for Thermally Activated Delayed Fluorescence Emitter (2CzPN). *ACS Appl Mater Interfaces* **2016**, *8*, 9806–9810, doi:10.1021/ACSAMI.6B00286/ASSET/IMAGES/LARGE/AM-2016-00286H_0005.JPEG.

353. Seob Park, I.; Youn Lee, S.; Adachi, C.; Yasuda, T.; Park, I.S.; Yasuda, T.; Lee, S.Y.; Adachi, C. Full-Color Delayed Fluorescence Materials Based on Wedge-Shaped Phthalonitriles and Dicyanopyrazines: Systematic Design, Tunable Photophysical Properties, and OLED Performance. *Adv Funct Mater* **2016**, *26*, 1813–1821, doi:10.1002/ADFM.201505106.

354. Park, I.S.; Lee, J.; Yasuda, T. High-Performance Blue Organic Light-Emitting Diodes with 20% External Electroluminescence Quantum Efficiency Based on Pyrimidine-Containing Thermally Activated Delayed Fluorescence Emitters. *J Mater Chem C Mater* **2016**, *4*, 7911–7916, doi:10.1039/C6TC02027E.

355. Kukhta, N.A.; Matulaitis, T.; Volyniuk, D.; Ivaniuk, K.; Turyk, P.; Stakhira, P.; Grazulevicius, J. V.; Monkman, A.P. Deep-Blue High-Efficiency TTA OLED Using Para- and Meta-Conjugated Cyanotriphenylbenzene and Carbazole Derivatives as Emitter and Host. *Journal of Physical Chemistry Letters* **2017**, *8*, 6199–6205, doi:10.1021/ACS.JPCLETT.7B02867/SUPPL_FILE/JZ7B02867_SI_001.PDF.

356. Pei, J.; Du, X.; Li, C.; Wang, C.; Fan, C.; Tan, H.; Cao, B.; Huang, F.; Tao, S.; Li, J. Highly Twisted Organic Molecules with Ortho Linkage as the Efficient Bipolar Hosts for Sky-Blue Thermally Activated Delayed Fluorescence Emitter in OLEDs. *Org Electron* **2017**, *50*, 153–160, doi:10.1016/J.ORGEL.2017.07.038.

357. Lee, J.; Aizawa, N.; Yasuda, T. Isobenzofuranone- and Chromone-Based Blue Delayed Fluorescence Emitters with Low Efficiency Roll-Off in Organic Light-Emitting Diodes. *Chemistry of Materials* **2017**, *29*, 8012–8020, doi:10.1021/ACS.CHEMMATER.7B03371/SUPPL_FILE/CM7B03371_SI_001.PDF.

358. Wu, S.-F.; Li, S.-H.; Wang, Y.-K.; Huang, C.-C.; Sun, Q.; Liang, J.-J.; Liao, L.-S.; Fung, M.-K.; Wu, S.-F.; Li, S.-H.; et al. White Organic LED with a Luminous Efficacy Exceeding 100 Lm W−1 without Light Out-Coupling Enhancement Techniques. *Adv Funct Mater* **2017**, *27*, 1701314, doi:10.1002/ADFM.201701314.

359. Ihn, S.G.; Lee, N.; Jeon, S.O.; Sim, M.; Kang, H.; Jung, Y.; Huh, D.H.; Son, Y.M.; Lee, S.Y.; Numata, M.; et al. An Alternative Host Material for Long-Lifespan Blue Organic Light-Emitting Diodes Using Thermally Activated Delayed Fluorescence. *Advanced Science* **2017**, *4*, 1600502, doi:10.1002/ADVS.201600502.

360. Cui, Y.; Liu, J. An Efficient Material via Meta-Position Connection as Thermally Activated Delayed Fluorescence Emitter for Organic Light-Emitting Diodes. *Tetrahedron Lett* **2017**, *16*, 1579–1584, doi:10.1016/J.TETLET.2017.03.018.

361. Nakao, K.; Sasabe, H.; Komatsu, R.; Hayasaka, Y.; Ohsawa, T.; Kido, J.; Nakao, K.; Sasabe, H.; Komatsu, R.; Hayasaka, Y.; et al. Significant Enhancement of Blue OLED Performances through Molecular Engineering of Pyrimidine-Based Emitter. *Adv Opt Mater* **2017**, *5*, 1600843, doi:10.1002/ADOM.201600843.

362. Miwa, T.; Kubo, S.; Shizu, K.; Komino, T.; Adachi, C.; Kaji, H. Blue Organic Light-Emitting Diodes Realizing External Quantum Efficiency over 25% Using Thermally Activated Delayed Fluorescence Emitters. *Scientific Reports 2017 7:1* **2017**, *7*, 1–8, doi:10.1038/s41598-017-00368-5.

363. Hladka, I.; Volyniuk, D.; Bezvikonnyi, O.; Kinzhybalo, V.; Bednarchuk, T.J.; Danyliv, Y.; Lytvyn, R.; Lazauskas, A.; Grazulevicius, J. V. Polymorphism of Derivatives of Tert-Butyl Substituted Acridan and Perfluorobiphenyl as Sky-Blue OLED Emitters Exhibiting Aggregation Induced Thermally Activated Delayed Fluorescence. *J Mater Chem C Mater* **2018**, *6*, 13179–13189, doi:10.1039/C8TC04867C.

364. Oh, C.S.; Pereira, D.D.S.; Han, S.H.; Park, H.J.; Higginbotham, H.F.; Monkman, A.P.; Lee, J.Y. Dihedral Angle Control of Blue Thermally Activated Delayed Fluorescent Emitters through Donor Substitution Position for Efficient Reverse Intersystem Crossing. *ACS Appl Mater Interfaces* **2018**, *10*, 35420–35429, doi:10.1021/ACSAMI.8B10595/SUPPL_FILE/AM8B10595_SI_005.CIF.

365. Ganesan, P.; Chen, D.G.; Liao, J.L.; Li, W.C.; Lai, Y.N.; Luo, Di.; Chang, C.H.; Ko, C.L.; Hung, W.Y.; Liu, S.W.; et al. Isomeric Spiro-[Acridine-9,9'-Fluorene]-2,6-Dipyridylpyrimidine Based TADF Emitters: Insights into Photophysical Behaviors and OLED Performances. *J Mater Chem C Mater* **2018**, *6*, 10088–10100, doi:10.1039/C8TC03645D.

366. Wong, M.Y.; Krotkus, S.; Copley, G.; Li, W.; Murawski, C.; Hall, D.; Hedley, G.J.; Jaricot, M.; Cordes, D.B.; Slawin, A.M.Z.; et al. Deep-Blue Oxadiazole-Containing Thermally Activated Delayed Fluorescence Emitters for Organic Light-Emitting Diodes. *ACS Appl Mater Interfaces* **2018**, *10*, 33360–33372, doi:10.1021/ACSAMI.8B11136/SUPPL_FILE/AM8B11136_SI_002.CIF.

367. Liang, X.; Yan, Z.-P.; Han, H.-B.; Wu, Z.-G.; ou-Xuan Zheng, Y.; Meng, H.; Zuo, J.-L.; uang, W.; Liang, X.; Yan, Z.; et al. Peripheral Amplification of Multi-Resonance Induced Thermally Activated Delayed Fluorescence for Highly Efficient OLEDs. *Angewandte Chemie International Edition* **2018**, *57*, 11316–11320, doi:10.1002/ANIE.201806323.

368. Zhang, Q.; Xiang, S.; Huang, Z.; Sun, S.; Ye, S.; Lv, X.; Liu, W.; Guo, R.; Wang, L. Molecular Engineering of Pyrimidine-Containing Thermally Activated Delayed Fluorescence Emitters for Highly Efficient Deep-Blue (CIEy < 0.06) Organic Light-Emitting Diodes. *Dyes and Pigments* **2018**, *155*, 51–58, doi:10.1016/J.DYEPIG.2018.03.004.

369. Ahn, D.H.; Moon, J.S.; Kim, S.W.; Lee, S.Y.; Karthik, D.; Lee, J.Y.; Kwon, J.H. Effect of Various Host Characteristics on Blue Thermally Activated Delayed Fluorescent Devices. *Org Electron* **2018**, *59*, 39–44, doi:10.1016/J.ORGEL.2018.04.034.

370. Zhang, W.; Zhang, Y.X.; Zhang, X.Q.; Liu, X.Y.; Fan, J.; Liao, L.S. Blue Thermally Activated Delayed Fluorescence Materials Based on Bi/Tri-Carbazole Derivatives. *Org Electron* **2018**, *58*, 238–244, doi:10.1016/J.ORGEL.2018.04.022.

371. Xiang, S.; Lv, X.; Sun, S.; Zhang, Q.; Huang, Z.; Guo, R.; Gu, H.; Liu, S.; Wang, L. To Improve the Efficiency of Thermally Activated Delayed Fluorescence OLEDs by Controlling the Horizontal Orientation through Optimizing Stereoscopic and Linear Structures of Indolocarbazole Isomers. *J Mater Chem C Mater* **2018**, *6*, 5812–5820, doi:10.1039/C8TC01419A.

372. The Role of Fluorine-Substitution on the π-Bridge in Constructing Effective Thermally Activated Delayed Fluorescence Molecules | Request PDF Available online: https://www.researchgate.net/publication/324713980_The_role_of_fluorine-substitution_on_the_p-bridge_in_constructing_effective_thermally_activated_delayed_fluorescence_molecules (accessed on 12 May 2022).

373. Yang, R.; Guan, Q.; Liu, Z.; Song, W.; Hong, L.; Lei, T.; Wei, Q.; Peng, R.; Fan, X.; Ge, Z. A Methodological Study on Tuning the Thermally Activated Delayed Fluorescent Performance by Molecular Constitution in Acridine-Benzophenone Derivatives. *Chem Asian J* **2018**, *13*, 1187–1191, doi:10.1002/ASIA.201800173.

374. Liang, J.J.; Li, Y.; Yuan, Y.; Li, S.H.; Zhu, X.D.; Barlow, S.; Fung, M.K.; Jiang, Z.Q.; Marder, S.R.; Liao, L.S. A Blue Thermally Activated Delayed Fluorescence Emitter Developed by Appending a Fluorene Moiety to a Carbazole Donor with Meta-Linkage for High-Efficiency OLEDs. *Mater Chem Front* **2018**, *2*, 917–922, doi:10.1039/C7QM00605E.

375. Liang, J.; Li, C.; Zhuang, X.; Ye, K.; Liu, Y.; Wang, Y.; Liang, J.; Li, C.; Zhuang, X.; Ye, K.; et al. Novel Blue Bipolar Thermally Activated Delayed Fluorescence Material as Host Emitter for High-Efficiency Hybrid Warm-White OLEDs with Stable High Color-Rendering Index. *Adv Funct Mater* **2018**, *28*, 1707002, doi:10.1002/ADFM.201707002.

376. Youn, K.M.; Ahn, D.H.; Kim, G.H.; Karthik, D.; Lee, J.Y.; Kwon, J.H. Blue Thermally Activated Delayed Fluorescence Emitters with a δ-Pyridoindole Donor Moiety. *New Journal of Chemistry* **2018**, *42*, 5532–5539, doi:10.1039/C8NJ00767E.

377. Liang, X.; Han, H.B.; Yan, Z.P.; Liu, L.; Zheng, Y.X.; Meng, H.; Huang, W. Versatile Functionalization of Trifluoromethyl Based Deep Blue Thermally Activated Delayed Fluorescence Materials for Organic Light Emitting Diodes. *New Journal of Chemistry* **2018**, *42*, 4317–4323, doi:10.1039/C7NJ04482H.

378. Chan, C.-Y.; Cui, L.-S.; Uk Kim, J.; Nakanotani, H.; Adachi, C.; Chan, C.; Cui, L.; Kim, J.U.; Nakanotani, H.; Adachi, C. Rational Molecular Design for Deep-Blue Thermally Activated Delayed Fluorescence Emitters. *Adv Funct Mater* **2018**, *28*, 1706023, doi:10.1002/ADFM.201706023.

379. Li, B.; Li, Z.; Hu, T.; Zhang, Y.; Wang, Y.; Yi, Y.; Guo, F.; Zhao, L. Highly Efficient Blue Organic Light-Emitting Diodes from Pyrimidine-Based Thermally Activated Delayed Fluorescence Emitters. *J Mater Chem C Mater* **2018**, *6*, 2351–2359, doi:10.1039/C7TC05746F.

380. Moon, J.S.; Ahn, D.H.; Kim, S.W.; Lee, S.Y.; Lee, J.Y.; Kwon, J.H. δ-Carboline-Based Bipolar Host Materials for Deep Blue Thermally Activated Delayed Fluorescence OLEDs with High Efficiency and Low Roll-off Characteristic. *RSC Adv* **2018**, *8*, 17025–17033, doi:10.1039/C8RA01761A.

381. Dos Santos, P.L.; Chen, D.; Rajamalli, P.; Matulaitis, T.; Cordes, D.B.; Slawin, A.M.Z.; Jacquemin, D.; Zysman-Colman, E.; Samuel, I.D.W. Use of Pyrimidine and Pyrazine Bridges as a Design Strategy to Improve the Performance of Thermally Activated Delayed Fluorescence Organic Light Emitting Diodes. *ACS Appl Mater Interfaces* **2019**, *11*, 45171–45179, doi:10.1021/ACSAMI.9B16952/SUPPL_FILE/AM9B16952_SI_004.CIF.

382. Hall, D.; Suresh, S.M.; dos Santos, P.L.; Duda, E.; Bagnich, S.; Pershin, A.; Rajamalli, P.; Cordes, D.B.; Slawin, A.M.Z.; Beljonne, D.; et al. Improving Processability and Efficiency of Resonant TADF Emitters: A Design Strategy. *Adv Opt Mater* **2020**, *8*, 1901627, doi:10.1002/ADOM.201901627.

383. Kwon, T.H.; Jeon, S.O.; Numata, M.; Lee, H.; Chung, Y.S.; Kim, J.S.; Ihn, S.G.; Sim, M.; Kim, S.; Kim, B.M. A Novel Design Strategy for Suppressing Efficiency Roll-Off of Blue Thermally Activated Delayed Fluorescence Molecules through Donor-Acceptor Interlocking by C-C Bonds. *Nanomaterials (Basel)* **2019**, *9*, doi:10.3390/NANO9121735.

384. Xu, M.; Liu, M.; Shi, M.; Ali, M.U.; Jiao, S.; Cao, W.; Wu, Y.C.; Meng, H. Efficient Thermally Activated Delayed Fluorescence Based on Carbonitrile-Substituted Pyridine and Carbazole. *J Mater Chem C Mater* **2019**, *7*, 13754–13758, doi:10.1039/C9TC03954F.

385. Huang, R.; Kukhta, N.A.; Ward, J.S.; Danos, A.; Batsanov, A.S.; Bryce, M.R.; Dias, F.B. Balancing Charge-Transfer Strength and Triplet States for Deep-Blue Thermally Activated Delayed Fluorescence with an Unconventional Electron Rich Dibenzothiophene Acceptor. *J Mater Chem C Mater* **2019**, *7*, 13224–13234, doi:10.1039/C9TC02175B.

386. Hippola, C.; Danilovic, D.; Bhattacharjee, U.; Perez-Bolivar, C.; Sachinthani, K.A.N.; Nelson, T.L.; Anzenbacher, P.; Petrich, J.W.; Shinar, R.; Shinar, J. Bright Deep Blue TADF OLEDs: The Role of Triphenylphosphine Oxide in NPB/TPBi:PPh3O Exciplex Emission. *Adv Opt Mater* **2020**, *8*, 0191282, doi:10.1002/ADOM.201901282.

387. Karthik, D.; Lee, S.Y.; Ahn, D.H.; Lee, H.; Lee, J.Y.; Kwon, J.H.; Park, J.H. Comparative Analysis of Various Indolocarbazole-Based Emitters on Thermally Activated Delayed Fluorescence Performances. *Org Electron* **2019**, *74*, 282–289, doi:10.1016/J.ORGEL.2019.07.024.

388. Du, R.; Duan, C.; Li, Y.; Zhang, J.; Han, C.; Xu, H. Spirobicyclic Host Material with Pseudo-Intramolecular Charge Transfer: Improving Color Purity of High-Performance Pure-Blue and White Thermally Activated Delayed Fluorescence Diodes. *Chemical Engineering Journal* **2019**, *374*, 471–478, doi:10.1016/J.CEJ.2019.05.192.

389. Li, G.; Lou, W.; Wang, D.; Deng, C.; Zhang, Q. Difluoroboron-Enabled Thermally Activated Delayed Fluorescence. *ACS Appl Mater Interfaces* **2019**, *11*, 32209–32217, doi:10.1021/ACSAMI.9B08107/SUPPL_FILE/AM9B08107_SI_001.PDF.

390. Cheng, Z.; Li, Z.; Xu, Y.; Liang, J.; Lin, C.; Wei, J.; Wang, Y. Achieving Efficient Blue Delayed Electrofluorescence by Shielding Acceptors with Carbazole Units. *ACS Appl Mater Interfaces* **2019**, *11*, 28096–28105, doi:10.1021/ACSAMI.9B07820/ASSET/IMAGES/LARGE/AM-2019-07820Y_0006.JPEG.

391. Stachelek, P.; Ward, J.S.; Dos Santos, P.L.; Danos, A.; Colella, M.; Haase, N.; Raynes, S.J.; Batsanov, A.S.; Bryce, M.R.; Monkman, A.P. Molecular Design Strategies for Color Tuning of Blue TADF Emitters. *ACS Appl Mater Interfaces* **2019**, *11*, 27125–27133, doi:10.1021/ACSAMI.9B06364/SUPPL_FILE/AM9B06364_SI_002.ZIP.

392. Li, W.; Li, B.; Cai, X.; Gan, L.; Xu, Z.; Li, W.; Liu, K.; Chen, D.; Su, S.J. Tri-Spiral Donor for High Efficiency and Versatile Blue Thermally Activated Delayed Fluorescence Materials. *Angewandte Chemie International Edition* **2019**, *58*, 11301–11305, doi:10.1002/ANIE.201904272.

393. Shi, Z.; Zhang, X.; Wang, H.; Huo, J.; Zhao, H.; Shi, H.; Tang, B.Z. The Synthesis, Photoluminescence and Electroluminescence Properties of a New Emitter Based on Diphenylethene, Carbazole and 9,9,10,10-Tetraoxidethianthrene. *Org Electron* **2019**, *70*, 7–13, doi:10.1016/J.ORGEL.2019.03.045.

394. Liu, J.; Zhou, K.; Wang, D.; Deng, C.; Duan, K.; Ai, Q.; Zhang, Q. Pyrazine-Based Blue Thermally Activated Delayed Fluorescence Materials: Combine Small Singlet-Triplet Splitting with Large Fluorescence Rate. *Front Chem* **2019**, *7*, 312, doi:10.3389/FCHEM.2019.00312/BIBTEX.

395. Ahn, D.H.; Lee, H.; Kim, S.W.; Karthik, D.; Lee, J.; Jeong, H.; Lee, J.Y.; Kwon, J.H. Highly Twisted Donor-Acceptor Boron Emitter and High Triplet Host Material for Highly Efficient Blue Thermally Activated Delayed Fluorescent Device. *ACS Appl Mater Interfaces* **2019**, *11*, 14909–14916, doi:10.1021/ACSAMI.9B00931/SUPPL_FILE/AM9B00931_SI_001.PDF.

396. Chapran, M.; Pander, P.; Vasylieva, M.; Wiosna-Salyga, G.; Ulanski, J.; Dias, F.B.; Data, P. Realizing 20% External Quantum Efficiency in Electroluminescence with Efficient Thermally Activated Delayed Fluorescence from an Exciplex. *ACS Appl Mater Interfaces* **2019**, *11*, 13460–13471, doi:10.1021/ACSAMI.8B18284/ASSET/IMAGES/LARGE/AM-2018-18284B_0008.JPEG.

397. Ohsawa, T.; Sasabe, H.; Watanabe, T.; Nakao, K.; Komatsu, R.; Hayashi, Y.; Hayasaka, Y.; Kido, J.; Ohsawa, T.; Sasabe, H.; et al. A Series of Imidazo[1,2-f]Phenanthridine-Based Sky-Blue TADF Emitters Realizing EQE of over 20%. *Adv Opt Mater* **2019**, *7*, 1801282, doi:10.1002/ADOM.201801282.

398. Xie, Y.; Gong, Y.; Han, M.; Zhang, F.; Peng, Q.; Xie, G.; Li, Z. Tetraphenylcyclopentadiene-Based Hyperbranched Polymers: Convenient Syntheses from One Pot "a 4 + B 2 " Polymerization and High External Quantum Yields up to 9.74% in OLED Devices. *Macromolecules* **2019**, *52*, 896–903, doi:10.1021/ACS.MACROMOL.8B02051/ASSET/IMAGES/LARGE/MA-2018-020513_0006.JPEG.

399. Bezvikonnyi, O.; Gudeika, D.; Volyniuk, D.; Mimaite, V.; Sebastine, B.R.; Grazulevicius, J. V. Effect of Donor Substituents on Thermally Activated Delayed Fluorescence of Diphenylsulfone Derivatives. *J Lumin* **2019**, *206*, 250–259, doi:10.1016/J.JLUMIN.2018.10.018.

400. Guo, R.; Leng, P.; Zhang, Q.; Wang, Y.; Lv, X.; Sun, S.; Ye, S.; Duan, Y.; Wang, L. Donor Engineering for Diphenylsulfone Derivatives with Both Thermally Activated Delayed Fluorescence and Aggregation-Induced Emission Properties. *Dyes and Pigments* **2021**, *184*, 108781, doi:10.1016/J.DYEPIG.2020.108781.

401. Aksoy, E.; Danos, A.; Varlikli, C.; Monkman, A.P. Navigating CIE Space for Efficient TADF Downconversion WOLEDs. *Dyes and Pigments* **2020**, *183*, 108707, doi:10.1016/J.DYEPIG.2020.108707.

402. Li, H.; Li, J.; Liu, D.; Mei, Y. Mechanism Evolution from Normal Fluorescence to Thermally Activated Delayed Fluorescence and Color Tuning over Visible Light Range: Effect of Intramolecular Charge Transfer Strength. *Dyes and Pigments* **2020**, *183*, 108732, doi:10.1016/J.DYEPIG.2020.108732.

403. Han, C.; Yang, W.; Xu, H. Asymmetrically Phosphorylated Carbazole Host for Highly Efficient Blue and White Thermally Activated Delayed Fluorescence Diodes. *Chemical Engineering Journal* **2020**, *401*, 126049, doi:10.1016/J.CEJ.2020.126049.

404. Yoon, J.; Kim, S.K.; Kim, H.J.; Choi, S.; Jung, S.W.; Lee, H.; Kim, J.Y.; Yoon, D.W.; Han, C.W.; Chae, W.S.; et al. Asymmetric Host Molecule Bearing Pyridine Core for Highly Efficient Blue Thermally Activated Delayed Fluorescence OLEDs. *Chemistry – A European Journal* **2020**, *26*, 16383–16391, doi:10.1002/CHEM.202002655.

405. Chen, Y.; Sun, Q.; Dai, Y.; Yang, D.; Qiao, X.; Ma, D. High Efficiency Blue and Color-Stable Hybrid Warm White Organic Light-Emitting Diodes Based on a Thermally Activated Delayed Fluorescent Material as an Assistant Host. *J Mater Chem C Mater* **2020**, *8*, 13777–13785, doi:10.1039/D0TC03644G.

406. Lim, H.; Cheon, J.; Woo, S.-J.; Kwon, S.-K.; Kim, Y.-H.; Kim, J.-J.; Lim, H.; Woo, S.-J.; Kim, J.-J.; Cheon, H.J.; et al. Highly Efficient Deep-Blue OLEDs Using a TADF Emitter with a Narrow Emission Spectrum and High Horizontal Emitting Dipole Ratio. *Advanced Materials* **2020**, *32*, 2004083, doi:10.1002/ADMA.202004083.

407. Xia, Y.; Liu, Z.; Li, J.; Fan, C.; Li, G.; Zhao, B.; Wu, Y.; Wang, H.; Guo, K. TADF Material with Non-Conjugated Rigid Donor for High-Performance Full-Color Phosphorescent OLEDs: Effects of Triplet Harvest and Charge Transport on Efficiency. *Org Electron* **2020**, *85*, 105826, doi:10.1016/J.ORGEL.2020.105826.

408. Maeng, J.H.; Ahn, D.H.; Lee, H.; Jung, Y.H.; Karthik, D.; Lee, J.Y.; Kwon, J.H. Rigid Indolocarbazole Donor Moiety for Highly Efficient Thermally Activated Delayed Fluorescent Device. *Dyes and Pigments* **2020**, *180*, 108485, doi:10.1016/J.DYEPIG.2020.108485.

409. Serevičius, T.; Dodonova, J.; Skaisgiris, R.; Banevičius, D.; Kazlauskas, K.; Juršėnas, S.; Tumkevičius, S. Optimization of the Carbazole–Pyrimidine Linking Pattern for Achieving Efficient TADF. *J Mater Chem C Mater* **2020**, *8*, 11192–11200, doi:10.1039/D0TC02194F.

410. Gudeika, D.; Bezvikonnyi, O.; Volyniuk, D.; Skuodis, E.; Lee, P.H.; Chen, C.H.; Ding, W.C.; Lee, J.H.; Chiu, T.L.; Grazulevicius, J. V. Oxygen Sensing and OLED Applications of Di-Tert-Butyl-Dimethylacridinyl Disubstituted Oxygafluorene Exhibiting Long-Lived Deep-Blue Delayed Fluorescence. *J Mater Chem C Mater* **2020**, *8*, 9632–9638, doi:10.1039/D0TC01648A.

411. Su, L.; Cao, F.; Cheng, C.; Tsuboi, T.; Zhu, Y.; Deng, C.; Zheng, X.; Wang, D.; Liu, Z.; Zhang, Q. High Fluorescence Rate of Thermally Activated Delayed Fluorescence Emitters for Efficient and Stable Blue OLEDs. *ACS Appl Mater Interfaces* **2020**, *12*, 31706–31715, doi:10.1021/ACSAMI.0C07840/ASSET/IMAGES/LARGE/AM0C07840_0006.JPEG.

412. Duan, K.; Wang, D.; Yang, M.; Liu, Z.; Wang, C.; Tsuboi, T.; Deng, C.; Zhang, Q. Weakly Conjugated Phosphine Oxide Hosts for Efficient Blue Thermally Activated Delayed Fluorescence Organic Light-Emitting Diodes. *ACS Appl Mater Interfaces* **2020**, *12*, 30591–30599, doi:10.1021/ACSAMI.0C02800/ASSET/IMAGES/LARGE/AM0C02800_0007.JPEG.

413. Kreiza, G.; Banevičius, D.; Jovaišaitė, J.; Juršėnas, S.; Javorskis, T.; Vaitkevičius, V.; Orentas, E.; Kazlauskas, K. Realization of Deep-Blue TADF in Sterically Controlled Naphthyridines for Vacuum- and Solution-Processed OLEDs. *J Mater Chem C Mater* **2020**, *8*, 8560–8566, doi:10.1039/D0TC01637C.

414. Luo, Y.; Li, S.; Zhao, Y.; Li, C.; Pang, Z.; Huang, Y.; Yang, M.; Zhou, L.; Zheng, X.; Pu, X.; et al. An Ultraviolet Thermally Activated Delayed Fluorescence OLED with Total External Quantum Efficiency over 9%. *Advanced Materials* **2020**, *32*, 2001248, doi:10.1002/ADMA.202001248.

415. Shen, Y.F.; Zhao, W.L.; Lu, H.Y.; Wang, Y.F.; Zhang, D.W.; Li, M.; Chen, C.F. Naphthyridine-Based Thermally Activated Delayed Fluorescence Emitters for Highly Efficient Blue OLEDs. *Dyes and Pigments* **2020**, *178*, 108324, doi:10.1016/J.DYEPIG.2020.108324.

416. Serdiuk, I.E.; Serdiuk, I.E.; Ryoo, C.H.; Kozakiewicz, K.; Mońka, M.; Liberek, B.; Park, S.Y. Twisted Acceptors in the Design of Deep-Blue TADF Emitters: Crucial Role of Excited-State Relaxation in the Photophysics of Methyl-Substituted s-Triphenyltriazine Derivatives. *J Mater Chem C Mater* **2020**, *8*, 6052–6062, doi:10.1039/C9TC07102D.

417. Kim, J.U.; Park, I.S.; Chan, C.Y.; Tanaka, M.; Tsuchiya, Y.; Nakanotani, H.; Adachi, C. Nanosecond-Time-Scale Delayed Fluorescence Molecule for Deep-Blue OLEDs with Small Efficiency Rolloff. *Nature Communications 2020 11:1* **2020**, *11*, 1–8, doi:10.1038/s41467-020-15558-5.

418. Ahn, D.H.; Maeng, J.H.; Lee, H.; Yoo, H.; Lampande, R.; Lee, J.Y.; Kwon, J.H. Rigid Oxygen-Bridged Boron-Based Blue Thermally Activated Delayed Fluorescence Emitter for Organic Light-Emitting Diode: Approach towards Satisfying High Efficiency and Long Lifetime Together. *Adv Opt Mater* **2020**, *8*, 2000102, doi:10.1002/ADOM.202000102.

419. Cheng, Z.; Yang, T.; Li, Z.; Liang, J.; Lin, C.; Mu, X.; Wang, Y. From Sky Blue to Orange Red: Accomplishment of Single-Emitter Full-Color Electroluminescence via Manipulating Intermolecular π-π Interactions. *Org Electron* **2020**, *78*, 105550, doi:10.1016/J.ORGEL.2019.105550.

420. Balijapalli, U.; Tanaka, M.; Auffray, M.; Chan, C.Y.; Lee, Y.T.; Tsuchiya, Y.; Nakanotani, H.; Adachi, C. Utilization of Multi-Heterodonors in Thermally Activated Delayed Fluorescence Molecules and Their High Performance Bluish-Green Organic Light-Emitting Diodes. *ACS Appl Mater Interfaces* **2020**, *12*, 9498–9506, doi:10.1021/ACSAMI.9B20020/ASSET/IMAGES/LARGE/AM9B20020_0006.JPEG.

421. Zhang, Z.; Ding, D.; Wei, Y.; Zhang, J.; Han, C.; Xu, H. Excited-State Engineering of Universal Ambipolar Hosts for Highly Efficient Blue Phosphorescence and Thermally Activated Delayed Fluorescence Organic Light-Emitting Diodes. *Chemical Engineering Journal* **2020**, *382*, 122485, doi:10.1016/J.CEJ.2019.122485.

422. Yang, C.Y.; Lee, K.H.; Lee, J.Y. Zig-Zag Type Molecular Design Strategy of N-Type Hosts for Sky-Blue Thermally-Activated Delayed Fluorescence Organic Light-Emitting Diodes. *Chemistry - A European Journal* **2020**, *26*, 2429–2435, doi:10.1002/CHEM.201904477.

423. Liu, F.; Liu, H.; Tang, X.; Ren, S.; He, X.; Li, J.; Du, C.; Feng, Z.; Lu, P. Novel Blue Fluorescent Materials for High-Performance Nondoped Blue OLEDs and Hybrid Pure White OLEDs with Ultrahigh Color Rendering Index. *Nano Energy* **2020**, *68*, 104325, doi:10.1016/J.NANOEN.2019.104325.

424. Li, W.; Li, W.; Gan, L.; Li, M.; Zheng, N.; Ning, C.; Chen, D.; Wu, Y.C.; Su, S.J. J-Aggregation Enhances the Electroluminescence Performance of a Sky-Blue Thermally Activated Delayed-Fluorescence Emitter in Nondoped Organic Light-Emitting Diodes. *ACS Appl Mater Interfaces* **2020**, *12*, 2717–2723, doi:10.1021/ACSAMI.9B17585/ASSET/IMAGES/LARGE/AM9B17585_0004.JPEG.

425. Yi, C.L.; Ko, C.L.; Yeh, T.C.; Chen, C.Y.; Chen, Y.S.; Chen, D.G.; Chou, P.T.; Hung, W.Y.; Wong, K.T. Harnessing a New Co-Host System and Low Concentration of New TADF Emitters Equipped with Trifluoromethyl- And Cyano-Substituted Benzene as Core for High-Efficiency Blue OLEDs. *ACS Appl Mater Interfaces* **2020**, *12*, 2724–2732, doi:10.1021/ACSAMI.9B18272/ASSET/IMAGES/LARGE/AM9B18272_0008.JPEG.

426. Lee, Y.; Woo, S.J.; Kim, J.J.; Hong, J.I. Blue Thermally Activated Delayed Fluorescence Emitter Using Modulated Triazines as Electron Acceptors. *Dyes and Pigments* **2020**, *172*, 107864, doi:10.1016/J.DYEPIG.2019.107864.

427. Ban, X.; Lin, B.; Jiang, W.; Sun, Y. Constructing a Novel Dendron for a Self-Host Blue Emitter with Thermally Activated Delayed Fluorescence: Solution-Processed Nondoped Organic Light-Emitting Diodes with Bipolar Charge Transfer and Stable Color Purity. *Chem Asian J* **2017**, *12*, 216–223, doi:10.1002/ASIA.201601384.

428. Luo, J.; Gong, S.; Gu, Y.; Chen, T.; Li, Y.; Zhong, C.; Xie, G.; Yang, C. Multi-Carbazole Encapsulation as a Simple Strategy for the Construction of Solution-Processed, Non-Doped Thermally Activated Delayed Fluorescence Emitters. *J Mater Chem C Mater* **2016**, *4*, 2442–2446, doi:10.1039/C6TC00418K.

429. Cha, J.R.; Lee, C.W.; Gong, M.S. Effect of Increasing Electron Donor Units for High-Efficiency Blue Thermally Activated Delayed Fluorescence. *Dyes and Pigments* **2017**, *140*, 399–406, doi:10.1016/J.DYEPIG.2017.01.053.

430. Joo, C.W.; Cho, H.; Kwon, B.H.; Cho, N.S.; Kim, Y.; Kim, Y.H.; Lee, J. Development of Solution-Processable Blue/Hybrid-White OLEDs Based on Thermally Activated Delayed Fluorescence. *Journal of Industrial and Engineering Chemistry* **2018**, *65*, 35–39, doi:10.1016/J.JIEC.2018.03.030.

431. Byeon, S.Y.; Kim, J.; Lee, D.R.; Han, S.H.; Forrest, S.R.; Lee, J.Y. Nearly 100% Horizontal Dipole Orientation and Upconversion Efficiency in Blue Thermally Activated Delayed Fluorescent Emitters. *Adv Opt Mater* **2018**, *6*, doi:10.1002/adom.201701340.

432. Wu, K.; Wang, Z.; Zhan, L.; Zhong, C.; Gong, S.; Xie, G.; Yang, C. Realizing Highly Efficient Solution-Processed Homojunction-Like Sky-Blue OLEDs by Using Thermally Activated Delayed Fluorescent Emitters Featuring an Aggregation-Induced Emission Property. *J Phys Chem Lett* **2018**, *9*, 1547–1553, doi:10.1021/ACS.JPCLETT.8B00344.

433. Weissenseel, S.; Drigo, N.A.; Kudriashova, L.G.; Schmid, M.; Morgenstern, T.; Lin, K.H.; Prlj, A.; Corminboeuf, C.; Sperlich, A.; Brütting, W.; et al. Getting the Right Twist: Influence of Donor-Acceptor Dihedral Angle on Exciton Kinetics and Singlet-Triplet Gap in Deep Blue Thermally Activated Delayed Fluorescence Emitter. *Journal of Physical Chemistry C* **2019**, doi:10.1021/ACS.JPCC.9B08269/SUPPL_FILE/JP9B08269_SI_001.PDF.

434. Joo, C.W.; Huseynova, G.; Yifei, J.; Yoo, J.M.; Kim, Y.H.; Cho, N.S.; Lee, J.H.; Kim, Y.H.; Lee, J. Highly Efficient Solution-Processed Blue Organic Light-Emitting Diodes Based on Thermally Activated Delayed Fluorescence Emitters with Spiroacridine Donor. *Journal of Industrial and Engineering Chemistry* **2019**, *78*, 265–270, doi:10.1016/J.JIEC.2019.06.003.

435. Wang, R.; Liu, Y.; Hu, T.; Wei, X.; Liu, J.; Li, Z.; Hu, X.; Yi, Y.; Wang, P.; Wang, Y. Solution-Processed White Organic Light-Emitting Diodes with Bi-Component Emitting Layer Based on Symmetry Blue Spiro-Sulfone Derivative. *Org Electron* **2019**, *71*, 24–30, doi:10.1016/J.ORGEL.2019.04.034.

436. Ha, T.H.; Cho, E.H.; Lee, C.W.; Choi, H.J.; Kim, O.Y.; Chin, B.D. Improvement of Quantum Efficiency and Efficiency Roll-off in OLED Devices Using Blue Thermally Activated Delayed Fluorescent Materials. *Journal of the Korean Physical Society 2019 74:11* **2019**, *74*, 1059–1066, doi:10.3938/JKPS.74.1059.

437. Peng, C.-C.; Yang, S.-Y.; Li, H.-C.; Xie, G.-H.; Cui, L.-S.; Zou, S.-N.; Poriel, C.; Jiang, Z.-Q.; Liao, L.-S.; Peng, C.-C.; et al. Highly Efficient Thermally Activated Delayed Fluorescence via an Unconjugated Donor–Acceptor System Realizing EQE of Over 30%. *Advanced Materials* **2020**, *32*, 2003885, doi:10.1002/ADMA.202003885.

438. Khan, F.; Urbonas, E.; Volyniuk, D.; Grazulevicius, J. V.; Mobin, S.M.; Misra, R. White Hyperelectrofluorescence from Solution-Processable OLEDs Based on Phenothiazine Substituted Tetraphenylethylene Derivatives. *J Mater Chem C Mater* **2020**, *8*, 13375–13388, doi:10.1039/D0TC03136D.

439. Duan, J.; Liu, D.; Tian, W.; Jiang, L.; Jiang, W.; Sun, Y. Elevating the Triplet Level of Carbazolyl Benzonitrile-Based Dendritic Hosts by Suppressing Intramolecular Charge Transfer for Solution-Processed Blue Thermally Activated Delayed Fluorescence OLEDs. *Opt Mater (Amst)* **2020**, *104*, 109941, doi:10.1016/J.OPTMAT.2020.109941.

440. Kim, H.J.; Godumala, M.; Kim, S.K.; Yoon, J.; Kim, C.Y.; Park, H.; Kwon, J.H.; Cho, M.J.; Choi, D.H. Color-Tunable Boron-Based Emitters Exhibiting Aggregation-Induced Emission and Thermally Activated Delayed Fluorescence for Efficient Solution-Processable Nondoped Deep-Blue to Sky-Blue OLEDs. *Adv Opt Mater* **2020**, *8*, 1902175, doi:10.1002/ADOM.201902175.

441. Xie, F.M.; An, Z.D.; Xie, M.; Li, Y.Q.; Zhang, G.H.; Zou, S.J.; Chen, L.; Chen, J. De; Cheng, T.; Tang, J.X. Tert-Butyl Substituted Hetero-Donor TADF Compounds for Efficient Solution-Processed Non-Doped Blue OLEDs. *J Mater Chem C Mater* **2020**, *8*, 5769–5776, doi:10.1039/D0TC00718H.

442. Ban, X.; Chen, F.; Pan, J.; Liu, Y.; Zhu, A.; Jiang, W.; Sun, Y. Exciplex Formation and Electromer Blocking for Highly Efficient Blue Thermally Activated Delayed Fluorescence OLEDs with All-Solution-Processed Organic Layers. *Chemistry – A European Journal* **2020**, *26*, 3090–3102, doi:10.1002/CHEM.201904415.

443. Zheng, X.; Huang, R.; Zhong, C.; Xie, G.; Ning, W.; Huang, M.; Ni, F.; Dias, F.B.; Yang, C.; Zheng, X.; et al. Achieving 21% External Quantum Efficiency for Nondoped Solution-Processed Sky-Blue Thermally Activated Delayed Fluorescence OLEDs by Means of Multi-(Donor/Acceptor) Emitter with Through-Space/-Bond Charge Transfer. *Advanced Science* **2020**, *7*, 1902087, doi:10.1002/ADVS.201902087.
